# Multiple transcription factors contribute to inter-chromosomal interaction in yeast

**DOI:** 10.1186/s12918-018-0643-1

**Published:** 2018-12-21

**Authors:** Yulin Dai, Chao Li, Guangsheng Pei, Xiao Dong, Guohui Ding, Zhongming Zhao, Yixue Li, Peilin Jia

**Affiliations:** 1 Center for Precision Health, School of Biomedical Informatics, The University of Texas Health Science Center at Houston, 7000 Fannin St. Suite 820, Houston, TX 77030 USA; 2 Key Laboratory of Systems Biology, Shanghai Institutes for Biological Sciences, Chinese Academy of Sciences, 320 Yueyang Rd, Shanghai, 200031 People’s Republic of China; 3 Graduate School of Chinese Academy of Sciences, 19 Yuquan Rd, Beijing, 100049 People’s Republic of China; 4 Shanghai Center for Bioinformation Technology, 1278 Keyuan Rd, Shanghai, 201203 People’s Republic of China; 5 Human Genetics Center, School of Public Health, The University of Texas Health Science Center at Houston, Houston, TX 77030 USA; 6 Department of Biomedical Informatics, Vanderbilt University Medical Center, Nashville, TN 37203 USA

**Keywords:** High-throughput chromosome conformation capture (hi-C), Spatial disposition of chromatins, Transcription factor (TF), Set12, Dig1, Centromere

## Abstract

**Background:**

Chromatin interactions medicated by genomic elements located throughout the genome play important roles in gene regulation and can be identified with the technologies such as high-throughput chromosome conformation capture (Hi-C), followed by next-generation sequencing. These techniques were wildly used to reveal the relative spatial disposition of chromatins in human, mouse and yeast. Unlike metazoan where CTCF plays major roles in mediating chromatin interactions, in yeast, the transcription factors (TFs) involved in this biological process are poorly known.

**Results:**

Here, we presented two computational approaches to estimate the TFs enriched in the chromatin physical inter-chromosomal interactions in yeast. Through the Chi-square method, we found TFs whose binding data are differentially distributed in different interaction groups, including Cin5, Stp1 and Sut1, whose binding data are negatively correlated with the chromosome spatial distance. A multivariate linear regression model was employed to estimate the potential contribution of different transcription factors against the physical distance of chromosomes. Rlr1, Set12 and Dig1 were found to be top positively participated in these chromosomal interactions. Ste12 was highlighted to be involved in gene reposition. Overall, we found 10 TFs enriched from both computational approaches, potentially to be involved in inter-chromosomal interactions.

**Conclusions:**

No transcription factor (TF) in our study was found to have a dominant impact on the inter-chromosomal interaction as CTCF did in human or other metazoan, suggesting species without CTCF might have different regulatory systems in mediating inter-chromosomal interactions. In summary, we presented a systematic examination of TFs involved in chromatin interaction in yeast and the results provide candidate TFs for future studies.

**Electronic supplementary material:**

The online version of this article (10.1186/s12918-018-0643-1) contains supplementary material, which is available to authorized users.

## Background

The existence of eukaryotic nucleus is an important distinction between eukaryotic and prokaryotic nucleus. Nucleus is a spatial organization with critical functional importance for gene expression, repression, RNA processing, and genomic replication [1]. The chromosome conformation capture technology utilizes restriction enzyme to digest DNA followed by ligation and paired-end sequencing. The results from Hi-C presented large scale paired-end reads which are interpreted as evidence supporting the spatial interaction between pairs of genomic segments. Since its invention, the Hi-C technology has been utilized in studying three-dimensional organization of genomes and provided novel insights into the genome architecture which are not possible using linear genome data. In higher eukaryotes, genomes are organized into topologically associating domains (TADs) which are associated with a fractal globule model of polymer folding and are considered to have a scaling relationship between genomic distance and contact frequency [2, 3]. In addition, a zinc finger protein, CCCTC-binding factor (CTCF), is responsible higher-order chromatin structure, like loop. In human nucleus, CTCF binding sites displayed a relationship with chromosomal interaction [4]. Interestingly, CTCF is conserved from fly to human. However, chromatin interactions in yeast remain complicated. Whereas TADs are conserved in drosophila, mouse, and human, they were not observed in yeast until recently. Two recent studies reported TADs in 200-kb scale and self-associated domains with 2~ 10 kb in size in budding yeast [5, 6], which provided evidence for genomic distance and contact frequency in yeast. Besides regional TADs, inter-chromosomal interaction is also considered not a random activity but is likely regulated by many transcription factors, which control gene expression by binding regulatory region of relevant genes [7, 8]. In yeast, it has been reported that centromere, telomere, breakpoints, tRNA and early replication origin genes were enriched in inter-chromosomal region [8, 9].

While inter-chromosomal interactions are experimentally proved in eukaryotes (eg., mainly mediated by CTCF) and are associated with potential functions (such as the transcription factory hypothesis [10] which states that genes on different chromosomes migrates to the transcription hotspot [11]), it remains poorly understood what TFs are involved in the inter-chromosomal interactions in yeast and what their functions are. In yeast, transcriptional regulators are likely function at short distance along the linear DNA, because more than 70% transcriptional regulator binding sites lie between 100 and 500 base pair upstream of protein-coding sequence [12]. In addition, two transcription factors, Ace2 and Ams2, were found to play important roles in recruiting condensin for global chromosomal organization in fission yeast [13]. Thus, it remains elusive whether TF binding sites are related with inter-chromosomal interactions and which TFs are potentially involved, as CTCF is absent [14]. In this study, we mapped yeast TF binding sites and Hi-C data to detect the potential TFs related to order inter-chromosomal structure.

## Methods

### Data sets

#### Hi-C data

Hi-C sequencing data were downloaded from Sequence Read Archive (accession ID: SRP002120) which was generated from Duan et al.’s study [9]. The experiment does not include cell cycle arrest. Therefore, these are average results among the whole cell cycle, and we cannot avoid the limitations from the “average model” [15]. False discovery rate control of the contact frequencies was conducted following the methods from the original study [9]. We split every chromosome of yeast with a size 1 kb as the data from chromosome conformation capture was at kilo-base resolution [9], resulting in non-overlapping chromosome segments. The Hi-C sequencing data were mapped to these segments. For any pair of segments (i.e., interactions), we count the number of reads mapped to either segments and denoted the total number of reads by n. We discarded interactions (pairs of fragments) where the two fragments were located shorter than 1 kb, because such segment pairs are unlikely to represent an interaction with each other. Interactions with 5 or less reads were also discarded, as those interactions contained a lot of false positive sites and more likely to be noises.

#### Yeast TF binding data

Yeast TF binding data was obtained from the Ref [12]. The data contains prediction results from six motif discovery programs for 203 TFs using genome-wide chromatin immunoprecipitation data. The regulatory map was constructed by finding all conserved occurrences of each motif within intergenic regions bound by the corresponding TF. TF binding site conserved in at least two other yeast were chosen. As a result, we had TF binding data for 105 TFs. Each TF binding sites were mapped to the chromosome segments used for Hi-C data. Finally, all chromosome segments were labelled whether they were overlapping with a TF binding site or not.

### Correlation between inter-chromosomal interactions and TF binding sites

We defined the following rules for our model to detect the relationship between interactions and transcription factor binding site.

**Fig. 1:**
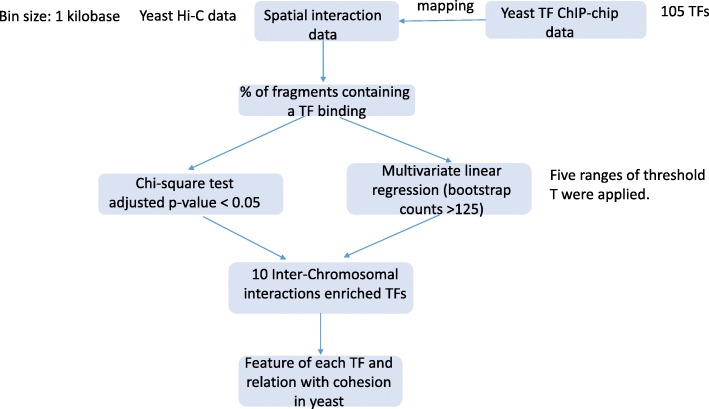
Distribution of interaction reads. **a** Distribution of interactions. The x-axis is the number of reads mapped to interactions. The y-axis is the number of interactions. Inter-chromosomal interactions (green) and all kinds of interactions (black) were plotted seperately. **b** Distribution of interactions in log10 scale

1. For each interaction, we used n to represent the number of counts obtained from Hi-C, where n measures the interaction intensity. We utilized N to represent those interaction fragments from Hi-C data that contains at least n reads.
2. Low-intensity filter: interactions with *n* ≤ 5 were removed from further analysis, as these interactions are likely noise in the Hi-C experiment data.
3. We investigated n = [6,…,100]. The interactions with *n* ≤ 100 accounted for 99.99% of all interactions.
4. For each interaction, we compared the genomic coordinates of both its interacting fragments and combine them with TF binding sites. An interaction is labelled as positive if at least one of its bins overlapped with a TFBS.
5. Lastly, for each threshold T we picked the corresponding fragments mentioned in rule 2nd and computed the percentage of those corresponding fragments that contains at least one TF binding site (Fig. 1).

### Method 1: Chi-square method to detect differential TF binding data in interactions

We utilized Chi-square test to assess whether the binding sites of a given TF showed different distribution in interactions with different intensity. Suppose T indicates the threshold that separates the interactions into two groups: one with n ≥ T (A) and the other with n < T ($\overline{A}$).

**Table:** 

	TFBS	
Interaction	∣*A* ∩ *B*∣	$\mid \overline{A}\cap B\mid$
	$\mid A\cap \overline{B}\mid$	$\mid \overline{A}\cap \overline{B}\mid$

Here, *A* denotes the set of interactions whose n ≥ T and *B* denotes the set of interactions that involve at least one segment which overlaps with a TF binding bin. The format |•| indicates the number of records referred by •. ∣A ∩ B∣ denotes the number of interactions with n ≥ T while overlapping with TF binding bins; $\mid \overline{A}\cap B\mid$ denotes the number interactions with n < T while overlapping with TF binding bins; $\mid A\cap \overline{B}\mid$ denotes the number of interactions with n ≥ T but not overlapping with any TF binding bins and $\mid \overline{A}\cap \overline{B}\mid$ represents the number of interactions with n < T and not overlapping with any TF binding bins. We used the Chi-square test to estimate whether a TF has its binding bins significantly enriched with interactions whose n was ≥ T. For each TF, we tested T from 7 and 45. Throughout this work, we defined an overlapping ratio for each TF at a given threshold T as |A ∩ B|/ ∣ *A*∣, which describes the proportion of interactions that overlapped with the binding bins of a given TF.

### Method 2: Regression model to detect differential TF binding data in interactions

As far as we know, CTCF binding pattern and inter-chromosomal interaction reads has positive relationship, we assumed the potential TFs contribute to yeast inter-chromosomal interaction might have a similar pattern [4]. Thus, the correlation relationship between interaction reads and TF binding data was modelled using a multivariate linear regression approach based on the Elastic Net algorithm implemented in the R package “glmnet” [16]. Elastic Net is a combination model of traditional Lasso and ridge regression methods, emphasizing model sparsity while appropriately balancing the contributions of correlated variables. It is ideal for building linear models in situations where the number of variables (markers) greatly outweighs the number of samples. Taking the interaction reads as the response variable, we included the overlapping ratio of all 105 TFs as the predictor in the model, aiming to select TFs that are most likely associated with the interaction reads. Optimal regularization parameters were estimated via 10-fold cross validation. We employed bootstrap analysis, sampling the data set with replacement 500 times. Because it is not known a priori at which read range the linear relationship fits the data appropriately, we tested 5 ranges, i.e., n = [6,…,40], [6,…,45], [6,…,50], [6,…,55], and [6,…,60] (similar to multiple threshold of T as we used in the Chi-square test). We selected the TFs that remained in the final Elastic Net model with more than 5% times (i.e., 25 out of 500 times for each range or 125 out of 2500 times in total) and computed the beta score of each picked TF.

## Results

### Overview of results: Distribution of interactions

**Fig. 2:**
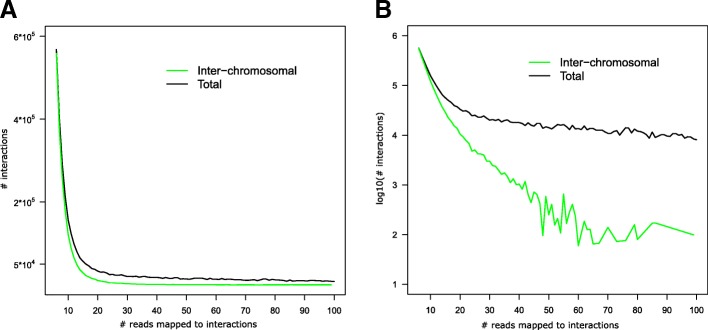
The analysis pipeline used in this work

Our analysis pipeline was presented in Fig. 2. By setting the interacting segments with size 1 kb, we found about 305,000 interactions involving 3678 unique fragments (~ 3.46 million reads in total), including about 240,000 interactions are inter-chromosome among 3480 unique fragments (~ 1.92 million reads in total). Following the rules described in methods, we plotted Fig. 1. The declining curve indicates that the distance between interacting segments is negatively correlated with the number of reads *n*, which is consistent with previous studies [4]. Around 50% of total interactions had no more than 10 reads. The number of interactions decreased dramatically when their mapped reads increased. On the very end of the distribution, there were only about 100 interactions that had 100 or more reads and 70 of them were between inter-chromosomal interactions. Previous works have defined two kinds of interactions according to their reads: the strong interactions and the weak interactions [4]. The strong interactions were referred to be the fragments contained more than 10 reads, as the binary fragments are likely to be spatially close to each other. The rest were referred as the weakly interacting fragments. In our results, we observed that at around 15, the interactions could be approximately distinguished as strong interactions and weak interactions.

### TFs that showed difference in their overlapping ratios: Results from the chi-square method

**Fig. 3:**
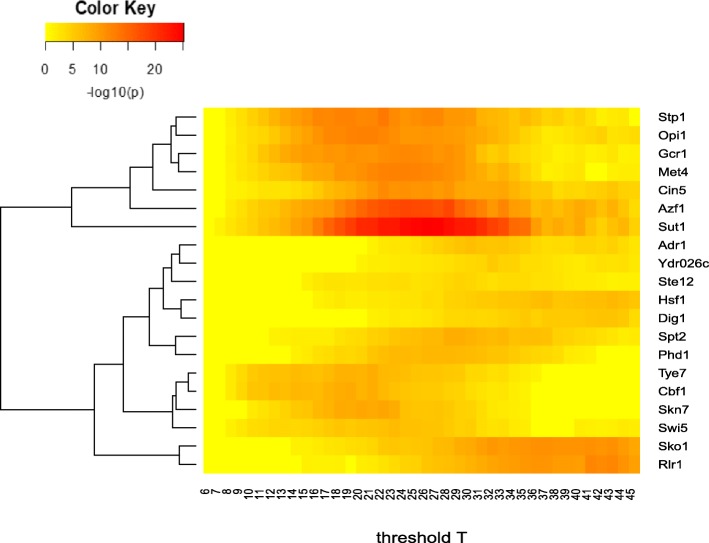
Distribution of results from the Chi-square method. We conducted the Chi-square test for each threshold T moving from 7 to 45 to reject those TFs which have unbalanced overlapping ratio before and after threshold T at least one time. The 20 TFs that showed significant differential interactions (adjusted *p*-value < 0.05, Bonferroni method) were shown in the figure. Although the adjusted p-value for Ste12 was not significant, it had a quite significant raw p-value and thus, we included it in the figure (Table 1 & Additional file 1)

To find the TFs that are significantly involved in spatial interaction, we applied Chi-square test on each of the 105 TFs with available data [12]. For each TF, we conducted Chi-square test multiple times depending on the different threshold of *T*, ranging from 7 to 45. Because *n* is proportional to spatial interaction, a TF that displayed significant differences at any threshold indicated that the TF might have significantly differentiated spatial interactions. As shown in Fig. 3, we found 20 TFs showing significant difference (adjusted *p*-value < 0.05 using the Bonferroni correction) with at least one threshold (Table 1). Among them, Sut1 had the strongest pattern at threshold 17–34, followed by Azf1 being differentiated at threshold 20–30. Interestingly, these TFs formed two groups according to the threshold ranges. Seven genes, Stp1, Opi1, Gcr1, Met4, Cin5, Azf1, and Sut1, showed significantly different distributions around the threshold range 15–35. In contrast, the other 13 TFs showed differentiation when the threshold of T tuned large, e.g., *T* > 30. Collectively, these TFs represented the candidates whose binding data were significantly different in the tested range 7–45. The result of 105 TFs Chi-square test is in Additional file 1.

**Table 1:** Significant TFs obtained using the Chi-square method and the multivariate regression model

Chi-square method			Multivariate regression model	
TF	p < 0.05	Adjusted *p* < 0.05	TF	# times selected
Sut1	39	33	Cin5^a^	2466
Azf1	38	34	Rlr1^a^	2032
Cin5^a^	38	23	Dig1^a^	1558
Opi1^a^	38	23	Ydr026c^a^	1286
Gcr1	38	22	Sok2	1235
Stp1	37	27	Spt2^a^	1037
Met4	36	23	Sko1^a^	783
Spt2^a^	34	16	Rlm1	556
Swi5	34	12	Adr1^a^	458
Sko1^a^	32	24	Ste12^a,b^	381
Ste12^a,b^	31	0	Hsf1^a^	376
Rlr1^a^	30	20	Uga3	360
Hsf1^a^	30	15	Opi1^a^	234
Tye7	29	20	Reb1	224
Cbf1	28	18	Pdr3	211
Phd1	28	14	Stb4	177
Skn7	26	15	Gat1	137
Ydr026c^a^	26	1		
Dig1^a^	25	10		
Adr1^a^	25	8		

^a^ Overlapping between two methods

^b^ Ste12 was not among the significant TF list but was manually selected because it contained more than 30 raw *p* < 0.05 and have several marginal adjusted *p* ~ 0.05

### TFs whose overlapping ratio was associated with reads *n*: Results from the regression model

To detect TFs which were associated with the number of reads, we built a predictive model to find the relationship between threshold *T* and the overlapping ratio of each TF using a penalized multivariate regression method known as Elastic Net [17], combined with a bootstrap approach. As a result, we identified 17 TFs that were selected in the final regression model for > 5% times, implying they were not selected by chance expectation (Table 1). The TF Cin5 had the strongest association with reads n, as it remained in the final model for nearly all resamples (2466 out of 2500). Other TFs with strong associations included Rlr1, Dig1, Ydr026c, Sok2 and Spt2. Bootstrap result for 105 TF is in Additional file 2**.**

### TFs related to chromatin interactions in yeast: The combined results

**Fig. 4:**
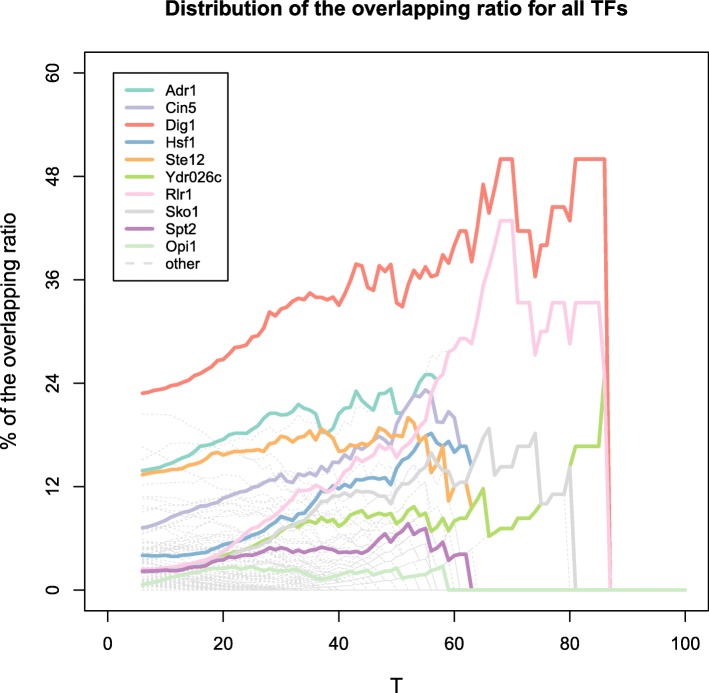
Distribution of the overlapping ratio for all 105 TFs. Y: The overlapping ratio. X: T, indicating the threshold that separates the interactions into two group. For example, when x = 40, the overlapping ratio is calculated for interactions with n > =40. Solid lines: significant TFs; different TFs were shown with different colors. Dashed lines: non-significant TFs (grey)

Comparing the result from the Chi-square test (Fig. 3) and the regression model (Table 1), we found 10 TFs that were significant according to both methods. We thus consider these 10 TFs as high-confident candidates which mediate the inter-chromosomal interactions. The overlapping ratio of each TF (i.e., |*A* ∩ *B*|/*A*) were plotted to threshold T in Fig. 4. The enriched 11 TFs have higher ratio and overall increasing curve than other non-significant TFs. The TF Dig1 displayed the strongest pattern, where its binding bins overlapped with interacting segments reached > 40%. Similarly, Rlr1 also had a quite high overlapping ratio at *n* > 60. Other TFs also showed a clear pattern that was distinct from non-significant ones, whose overlapping ratio failed to distinguish at any threshold. Overlapping ratio plot for each TF is in Additional file 3.

**Fig. 5:**
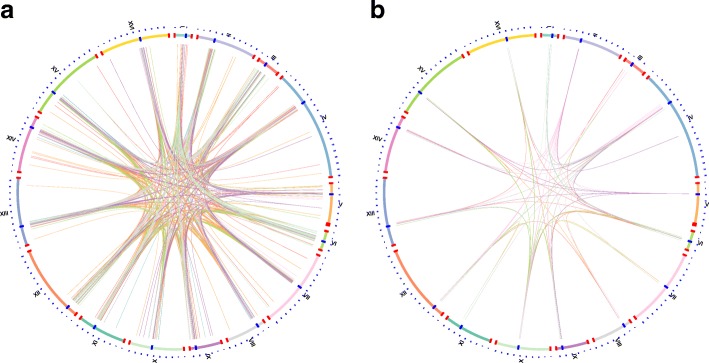
Circos diagram showing the interactions involved in the 10 significant TFs. The outside circle showed chromosomes, where red bars at both ends of each chromosome indicates centromeres and blue bars indicate telomeres. Each link indicates an interaction. The color of the link is the same as in Figure 4. **a** Circos diagram for *T* = 20. **b** Circos diagram for *T* = 40

We plotted the interactions involved in the 10 significant TFs using a circos diagram [18] (Fig. 5). To demonstrate the relationship between reads and chromosomal, we plotted interactions with *T* = 20 and *T* = 40. In T = 20 diagram (Fig. 5a), a few telomere interactions could be observed, besides the strong interactions between centromere and its periphery regions. In T = 40 diagram (Fig. 5b), the majority of these interactions were mainly distributed within the centromere region, its periphery region and telomere region. Circos diagrams for each significant TF at two conditions (T = 20, T = 40) is in Additional files 4.

**Fig. 6:**
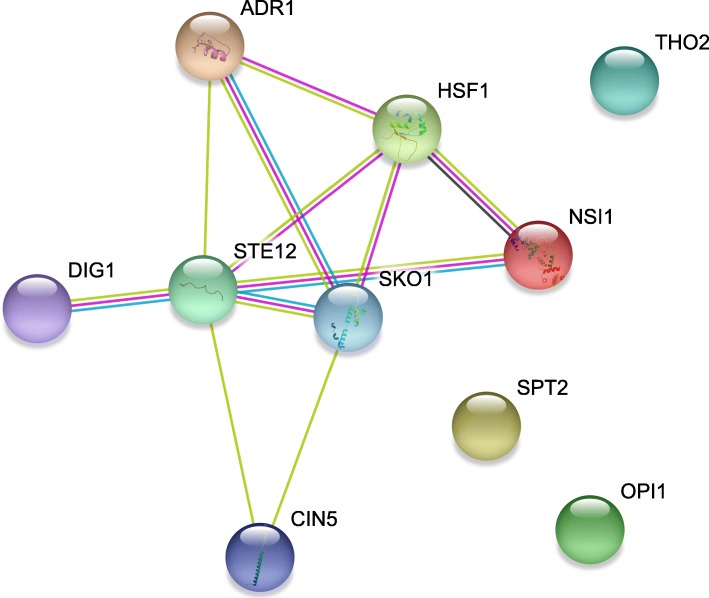
Protein-protein interaction (PPI) among the 10 significant TFs based on the STRING network. Seven out of the ten significant TFs had PPIs. THO2 is alias to Rlr1. NSI1 is alias to Ydr026c.

An investigation of the interactions among these TFs using the STRING network showed that 7 out of the 10 TFs had interactions or functional associations [19] (Fig. 6).

## Discussion

Chromatin interaction is associated with many critical biological processes in living cells, such as regulation of transcription, replication, and maintenance of chromatin structure. Identification of TFs that are involved in chromatin interactions could provide insights on the functions and mechanisms underlying cellular processes. In this work, we systematically investigated TFs that were involved in chromatin interaction and showed significant space difference in yeast. Our results highlights 10 TFs that are significantly involved in chromatin interactions. The interactions mediated by these TFs mainly occurred in centromere, telomere, and their periphery regions, implying potential functional importance.

Among the 10 significant TFs, Ste12 and Dig1 had been previously reported to be the centromere-binding proteins [20]. Ste12 is activated by a MAPK signalling cascade; activates genes involved in mating or pseudohyphal/invasive growth pathways. Dig1 (Down-regulator of Invasive Growth) is MAP kinase-responsive inhibitor of the Ste12p transcription factor; involved in the regulation of mating-specific genes and the invasive growth pathway [21–25]. Moreover, Ste12 was involved in recruiting genes from the nucleoplasm to the nuclear periphery [26]. Interestingly, we found Cbf1 (Centromere Binding Factor) significant enriched in the Chi-square method. Previous study has shown Cbf1 involved in methionine biosynthesis [27], which binding the core centromere DNA as a bona fide constitutive centromere protein [28]. Thus, Ste12 and Dig1 could regulate Cbf1 activity. Loss of Cbf1, Ste12 or Dig1 resulted in chromosomal instability. Moreover, chromosomal instability phenotype of Cbf1 mutants could be rescued by driving centromere transcription from an artificial promoter, indicating transcription at the centromere is vital to centromere function in yeast [20, 29].

Checking function of the other TFs in yeast genome database (https://www.yeastgenome.org/), we know that Ydr026c is a RNA polymerase I termination factor; binds to rDNA terminator element region between chromosomes in nucleolus [30–33]. Thus, the main function of Ydr026c is chromatin silencing at rDNA, which is an important biological process to limit rDNA transcribed to rRNA, and therefore keep the translation function stop. Spt2 has function similar to mammalian HMG1 (high-mobility group protein 1) proteins, which might act DNA chaperone involved in replication, transcription, chromatin remodeling and etc. [34–36]. Functions of Adr1, Hsf1, Rlr1, Sko1, Cin5 and Opi1 were concluded in Additional file 5.

As far as we know, there is no sophisticated method exactly designed to test the situation we encountered here. “Test For Trend In Proportions” can test whether the proportion of trends are consistent or not, which could be used to test the overlapping ratio trend. We used R function *prop.trend.test* () to conduct this test in 5 ranges defined in **Method** 2 (Additional file 6). We sorted the TFs by significance of the *p*-value. Ninety-two out of 105 TFs were significant after Bonferroni correction. Top 20 increasing TFs have 17 overlapping genes with our Chi-square method group, while 10 genes were found to be overlapped with multivariate regression model group (Additional file 7). The top significance TFs from proportion trend test have high accordance with our Chi-square method result. However, the interpretation of the whole significant TF set is very hard, since this proportion trend test cannot distinguish a stable trend, a decreasing trend or an increasing trend. Thus, a lot of false positive result will be kept in the significant set. Even we only keep those TFs which has an increasing ratio, it will still need extra methods to evaluate the importance of different TFs. Overall, our Chi-square method and multivariate regression model could greatly narrow down the candidate TF set.

We failed to detect the significant enrichment of Yap5, Dal80, and Stp23, which were discovered in previous study [5]. Even the same TF binding dataset was used, the different enrichment strategy and flanking region scale could bring difference. Ace2 and Ams2 were reported has the ability to recruit condensin to maintain topological association domain in fission yeast [13]. However, the reason that we have not found them enriched might because they were M/G1 stage specific expressed cell cycle TFs, while our Hi-C model is in an “average model” and the TF binding information we took was a mixture of different stage and environment [12].

## Conclusions

We presented an analysis framework to systematically detect TFs that are involved in chromatin interactions in yeast. Through two computational approaches, we identified 10 TFs that are significantly enriched in inter-chromosomal interactions. We highlighted two significantly enriched TFs, Ste12 and Dig1, which had been reported to be involved in centromeric transcript maintaining and spatial organization, as a proof for the rationality of our approach. No TF in our study was found to have a dominant impact on the inter-chromosomal interaction as CTCF did in human or other metazoan [4], suggesting species without CTCF might have different regulatory systems in mediating inter-chromosomal interactions. Our finding could provide candidate TFs for future studies.

## Additional files

Additional file 1: Table is for the result of 105 TFs from Chi-square approach. (XLSX 34 kb)

Additional file 2: Table is for Bootstrap result of 105 TFs from multivariate method. (XLSX 13 kb)

Additional file 3: A folder named SB-06-S3 contains 105 overlapping plot for each TF. (ZIP 624 kb)

Additional file 4: A folder named SB-06-S4 contains circos diagrams for 10 significant TFs at two conditions (*T* = 20, *T* = 40). (ZIP 2300 kb)

Additional file 5: Table contains function annotation for 10 significant TFs. (XLSX 8 kb)

Additional file 6: Table is for the result of 105 TFs conducted by “Test For Trend In Proportions” (prop.trend.test function in R). (XLSX 9 kb)

Additional file 7: Table contains the comparison between the top results of three approaches. (XLSX 18 kb)

## Abbreviations

CTCF: CCCTC-binding factor
Hi-C: High-throughput chromosome conformation capture
PPI: Protein-protein interaction
TADs: Topologically associating domains
TF: Transcription factor
TFs: Transcription factors

## References

[1] Taddei A, Gasser SM (2012). Structure and function in the budding yeast nucleus. Genetics.

[2] Lieberman-Aiden E, van Berkum NL, Williams L, Imakaev M, Ragoczy T, Telling A, Amit I, Lajoie BR, Sabo PJ, Dorschner MO (2009). Comprehensive mapping of long-range interactions reveals folding principles of the human genome. Science.

[3] Mirny LA (2011). The fractal globule as a model of chromatin architecture in the cell. Chromosom Res.

[4] Botta M, Haider S, Leung IX, Lio P, Mozziconacci J (2010). Intra- and inter-chromosomal interactions correlate with CTCF binding genome wide. Mol Syst Biol.

[5] Eser U, Chandler-Brown D, Ay F, Straight AF, Duan Z, Noble WS, Skotheim JM (2017). Form and function of topologically associating genomic domains in budding yeast. Proc Natl Acad Sci U S A.

[6] Hsieh TH, Weiner A, Lajoie B, Dekker J, Friedman N, Rando OJ (2015). Mapping nucleosome resolution chromosome folding in yeast by micro-C. Cell.

[7] Heun P, Laroche T, Shimada K, Furrer P, Gasser SM (2001). Chromosome dynamics in the yeast interphase nucleus. Science.

[8] Stone EM, Heun P, Laroche T, Pillus L, Gasser SM (2000). MAP kinase signaling induces nuclear reorganization in budding yeast. Curr Biol.

[9] Duan Z, Andronescu M, Schutz K, McIlwain S, Kim YJ, Lee C, Shendure J, Fields S, Blau CA, Noble WS (2010). A three-dimensional model of the yeast genome. Nature.

[10] Iborra FJ, Pombo A, Jackson DA, Cook PR (1996). Active RNA polymerases are localized within discrete transcription factories in human nuclei. J Cell Sci.

[11] Rieder D, Trajanoski Z, McNally JG (2012). Transcription factories. Front Genet.

[12] Harbison CT, Gordon DB, Lee TI, Rinaldi NJ, Macisaac KD, Danford TW, Hannett NM, Tagne JB, Reynolds DB, Yoo J (2004). Transcriptional regulatory code of a eukaryotic genome. Nature.

[13] Kim KD, Tanizawa H, Iwasaki O, Noma K (2016). Transcription factors mediate condensin recruitment and global chromosomal organization in fission yeast. Nat Genet.

[14] Cai M, Davis RW (1990). Yeast centromere binding protein CBF1, of the helix-loop-helix protein family, is required for chromosome stability and methionine prototrophy. Cell.

[15] Heermann DW, Jerabek H, Liu L, Li Y (2012). A model for the 3D chromatin architecture of pro and eukaryotes. Methods.

[16] Friedman J, Hastie T, Tibshirani R (2010). Regularization paths for generalized linear models via coordinate descent. J Stat Softw.

[17] Zou H, Hastie T (2003). Regularization and Variable Selection via the Elastic Net. J Royal Stat Soc Series B (Statistical Methodol).

[18] Hu Y, Yan C, Hsu CH, Chen QR, Niu K, Komatsoulis GA, Meerzaman D (2014). OmicCircos: a simple-to-use R package for the circular visualization of multidimensional omics data. Cancer Inform.

[19] Szklarczyk D, Morris JH, Cook H, Kuhn M, Wyder S, Simonovic M, Santos A, Doncheva NT, Roth A, Bork P (2017). The STRING database in 2017: quality-controlled protein-protein association networks, made broadly accessible. Nucleic Acids Res.

[20] Ohkuni K, Kitagawa K (2011). Endogenous transcription at the centromere facilitates centromere activity in budding yeast. Curr Biol.

[21] Cook JG, Bardwell L, Kron SJ, Thorner J (1996). Two novel targets of the MAP kinase Kss1 are negative regulators of invasive growth in the yeast Saccharomyces cerevisiae. Genes Dev.

[22] Bardwell L, Cook JG, Zhu-Shimoni JX, Voora D, Thorner J (1998). Differential regulation of transcription: repression by unactivated mitogen-activated protein kinase Kss1 requires the Dig1 and Dig2 proteins. Proc Natl Acad Sci U S A.

[23] Olson KA, Nelson C, Tai G, Hung W, Yong C, Astell C, Sadowski I (2000). Two regulators of Ste12p inhibit pheromone-responsive transcription by separate mechanisms. Mol Cell Biol.

[24] Gelli A (2002). Rst1 and Rst2 are required for the a/alpha diploid cell type in yeast. Mol Microbiol.

[25] McCullagh E, Seshan A, El-Samad H, Madhani HD (2010). Coordinate control of gene expression noise and interchromosomal interactions in a MAP kinase pathway. Nat Cell Biol.

[26] Randise-Hinchliff C, Brickner JH (2016). Transcription factors dynamically control the spatial organization of the yeast genome. Nucleus.

[27] O’Connell KF, Baker RE (1992). Possible cross-regulation of phosphate and sulfate metabolism in Saccharomyces cerevisiae. Genetics.

[28] Hemmerich P, Stoyan T, Wieland G, Koch M, Lechner J, Diekmann S (2000). Interaction of yeast kinetochore proteins with centromere-protein/transcription factor Cbf1. Proc Natl Acad Sci U S A.

[29] Chan FL, Wong LH (2012). Transcription in the maintenance of centromere chromatin identity. Nucleic Acids Res.

[30] Ha CW, Sung MK, Huh WK (2012). Nsi1 plays a significant role in the silencing of ribosomal DNA in Saccharomyces cerevisiae. Nucleic Acids Res.

[31] Fleischer TC, Weaver CM, McAfee KJ, Jennings JL, Link AJ (2006). Systematic identification and functional screens of uncharacterized proteins associated with eukaryotic ribosomal complexes. Genes Dev.

[32] Mohanty BK, Bastia D (2004). Binding of the replication terminator protein Fob1p to the Ter sites of yeast causes polar fork arrest. J Biol Chem.

[33] Reiter A, Hamperl S, Seitz H, Merkl P, Perez-Fernandez J, Williams L, Gerber J, Nemeth A, Leger I, Gadal O (2012). The Reb1-homologue Ydr026c/Nsi1 is required for efficient RNA polymerase I termination in yeast. EMBO J.

[34] Winston F, Chaleff DT, Valent B, Fink GR (1984). Mutations affecting ty-mediated expression of the HIS4 gene of Saccharomyces cerevisiae. Genetics.

[35] Perez-Martin J, Johnson AD (1998). The C-terminal domain of Sin1 interacts with the SWI-SNF complex in yeast. Mol Cell Biol.

[36] Hershkovits G, Bangio H, Cohen R, Katcoff DJ (2006). Recruitment of mRNA cleavage/polyadenylation machinery by the yeast chromatin protein Sin1p/Spt2p. Proc Natl Acad Sci U S A.

