# Supplementary figures and images for: Multiple transcription factors contribute to inter-chromosomal interaction in yeast (part 2 of 2)
Source: BMC Syst Biol. 2018 Dec 21;12(Suppl 8):140. doi: 10.1186/s12918-018-0643-1 (PMC6302461; doi:10.1186/s12918-018-0643-1)

# YAP7

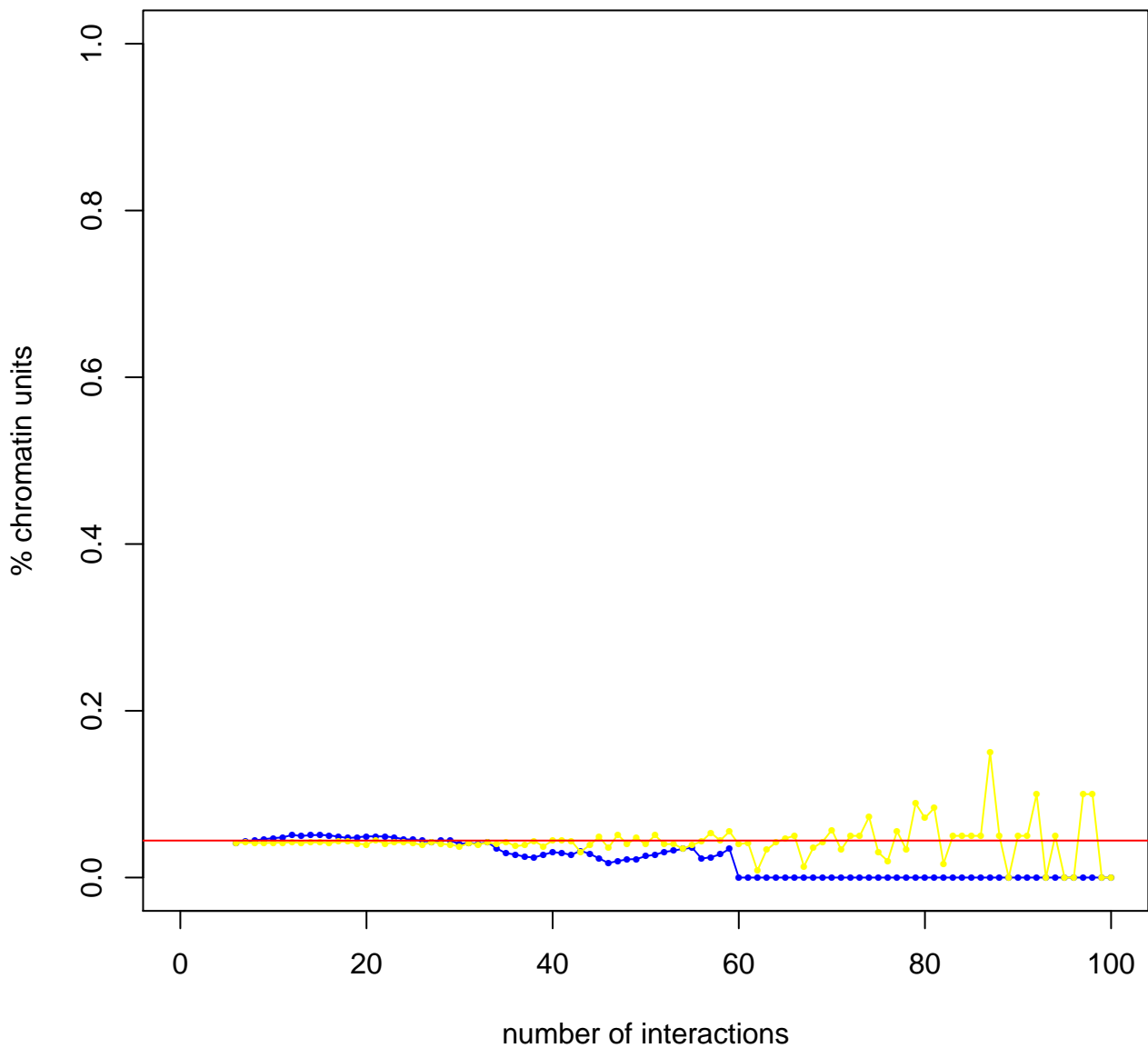

Supplement: Supplementary file 3 — A folder named SB-06-S3 contains 105 overlapping plot for each TF. (ZIP 624 kb) [file 12918_2018_643_MOESM3_ESM.zip › SB-06-S3/YAP7.pdf]

# YDR026c

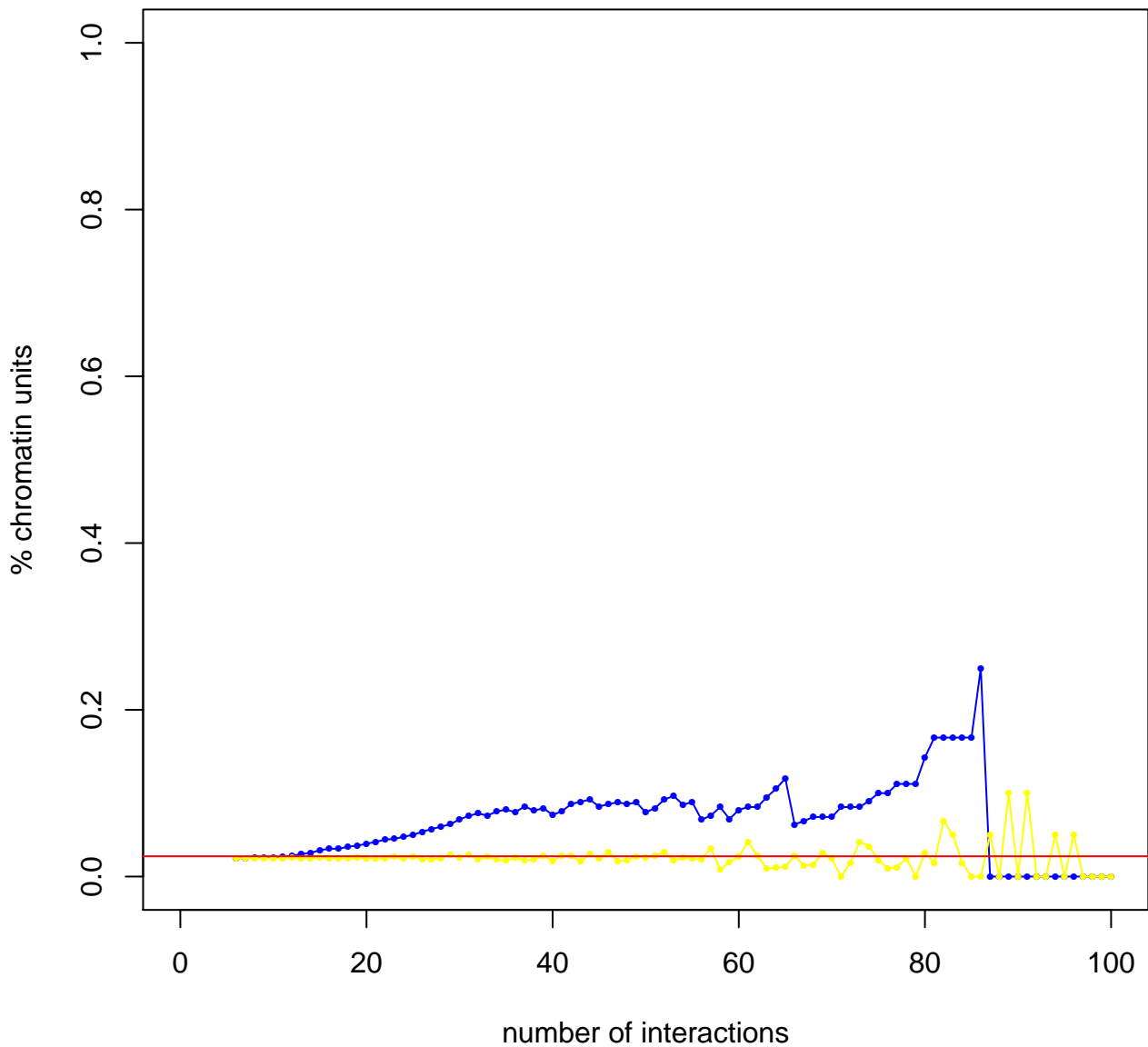

Supplement: Supplementary file 3 — A folder named SB-06-S3 contains 105 overlapping plot for each TF. (ZIP 624 kb) [file 12918_2018_643_MOESM3_ESM.zip › SB-06-S3/YDR026c.pdf]

# YHP1

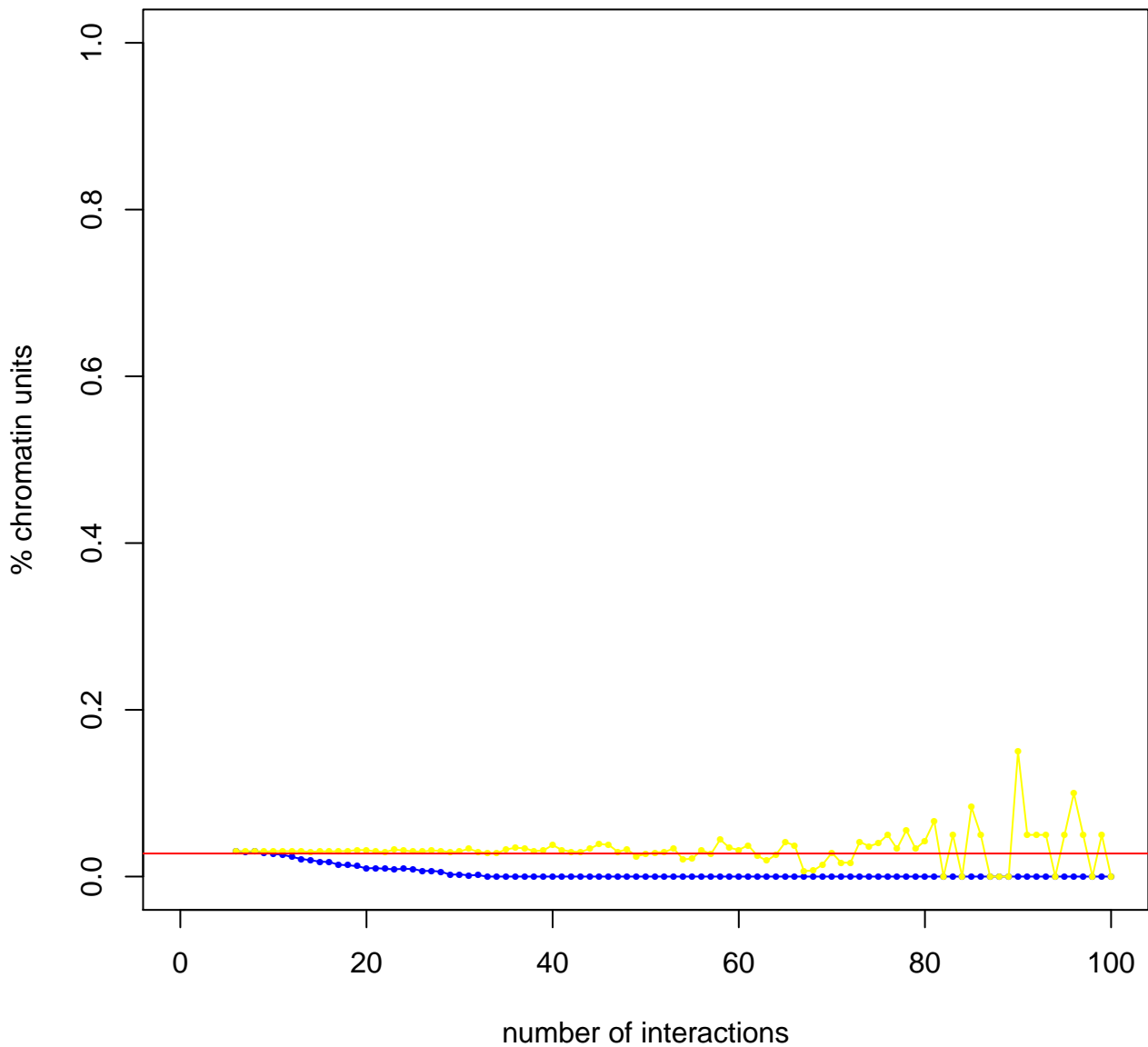

Supplement: Supplementary file 3 — A folder named SB-06-S3 contains 105 overlapping plot for each TF. (ZIP 624 kb) [file 12918_2018_643_MOESM3_ESM.zip › SB-06-S3/YHP1.pdf]

# YOX1

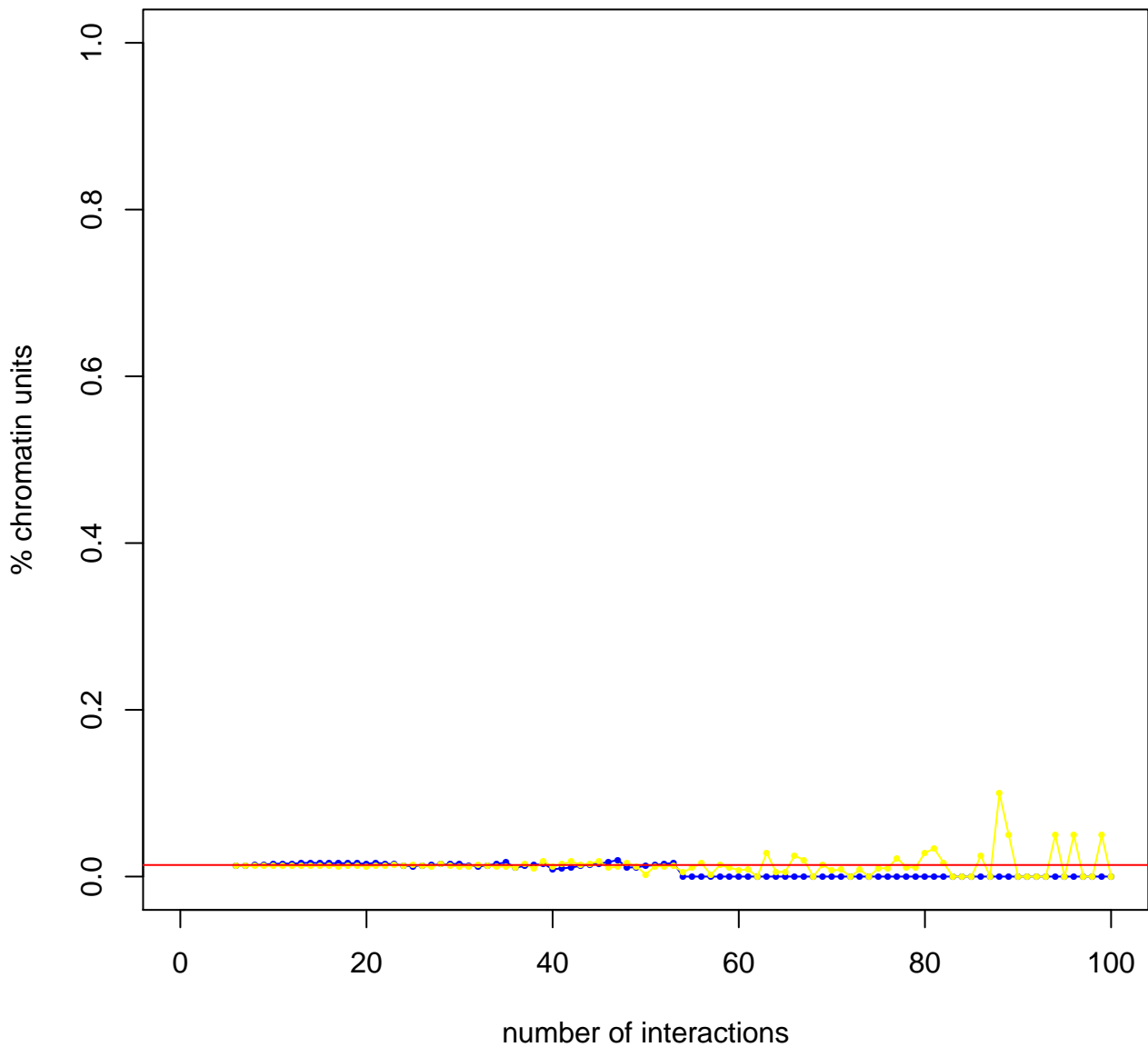

Supplement: Supplementary file 3 — A folder named SB-06-S3 contains 105 overlapping plot for each TF. (ZIP 624 kb) [file 12918_2018_643_MOESM3_ESM.zip › SB-06-S3/YOX1.pdf]

# ZAP1

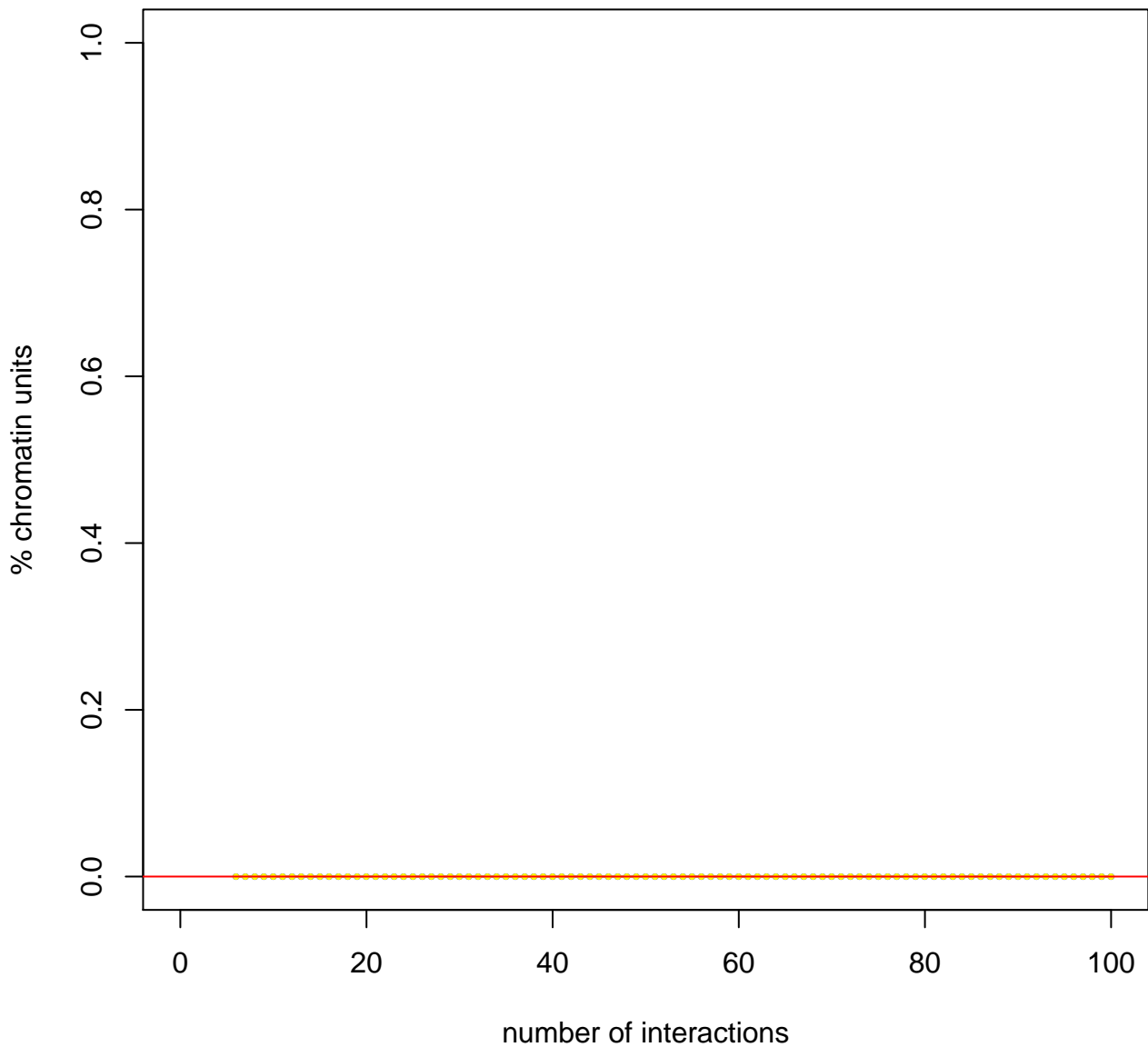

Supplement: Supplementary file 3 — A folder named SB-06-S3 contains 105 overlapping plot for each TF. (ZIP 624 kb) [file 12918_2018_643_MOESM3_ESM.zip › SB-06-S3/ZAP1.pdf]

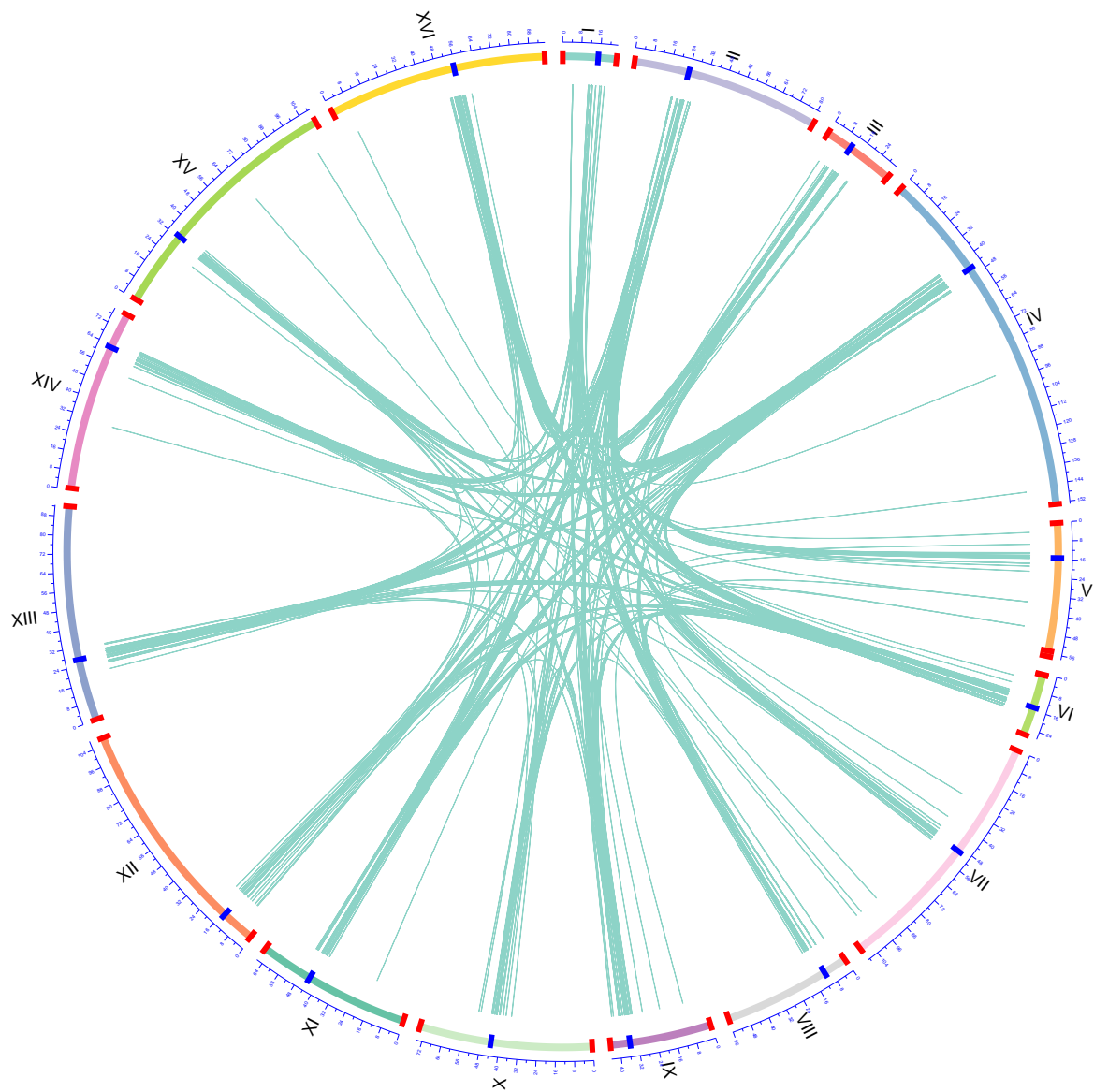

Supplement: Supplementary file 4 — A folder named SB-06-S4 contains circos diagrams for 10 significant TFs at two conditions (T = 20, T = 40). (ZIP 2300 kb) [file 12918_2018_643_MOESM4_ESM.zip › SB-06-S4/ADR1_20.pdf]

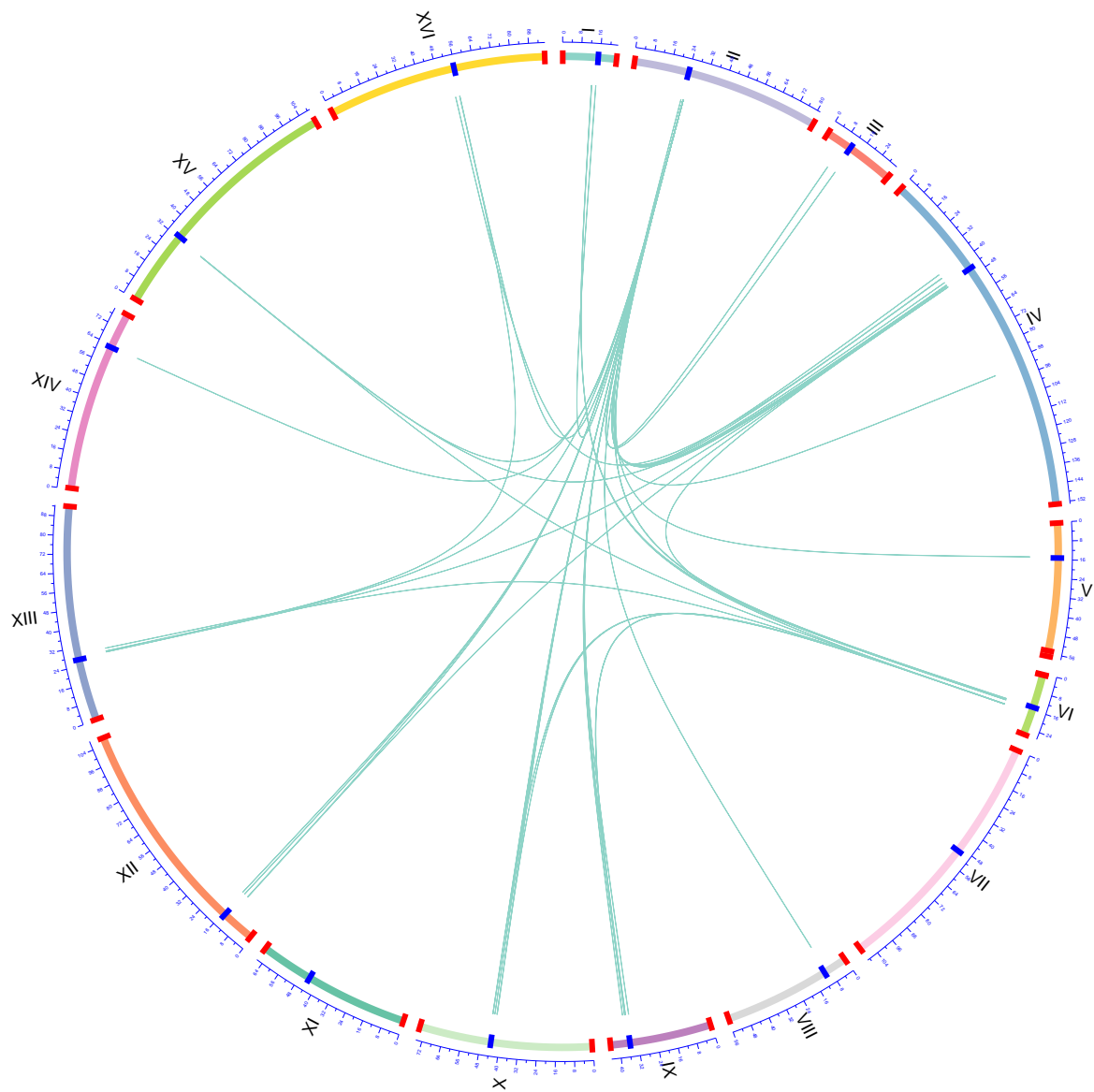

Supplement: Supplementary file 4 — A folder named SB-06-S4 contains circos diagrams for 10 significant TFs at two conditions (T = 20, T = 40). (ZIP 2300 kb) [file 12918_2018_643_MOESM4_ESM.zip › SB-06-S4/ADR1_40.pdf]

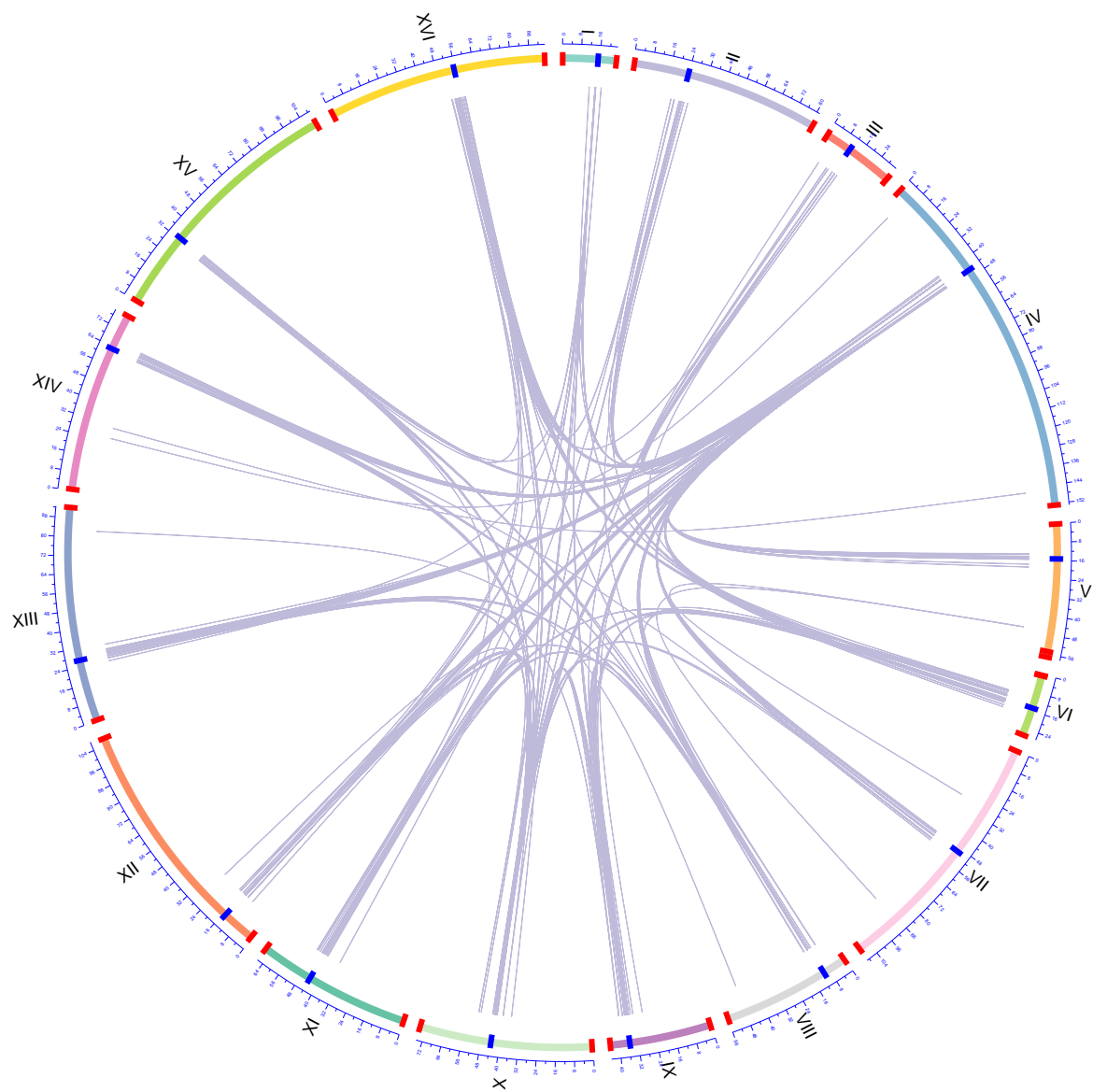

Supplement: Supplementary file 4 — A folder named SB-06-S4 contains circos diagrams for 10 significant TFs at two conditions (T = 20, T = 40). (ZIP 2300 kb) [file 12918_2018_643_MOESM4_ESM.zip › SB-06-S4/CIN5_20.pdf]

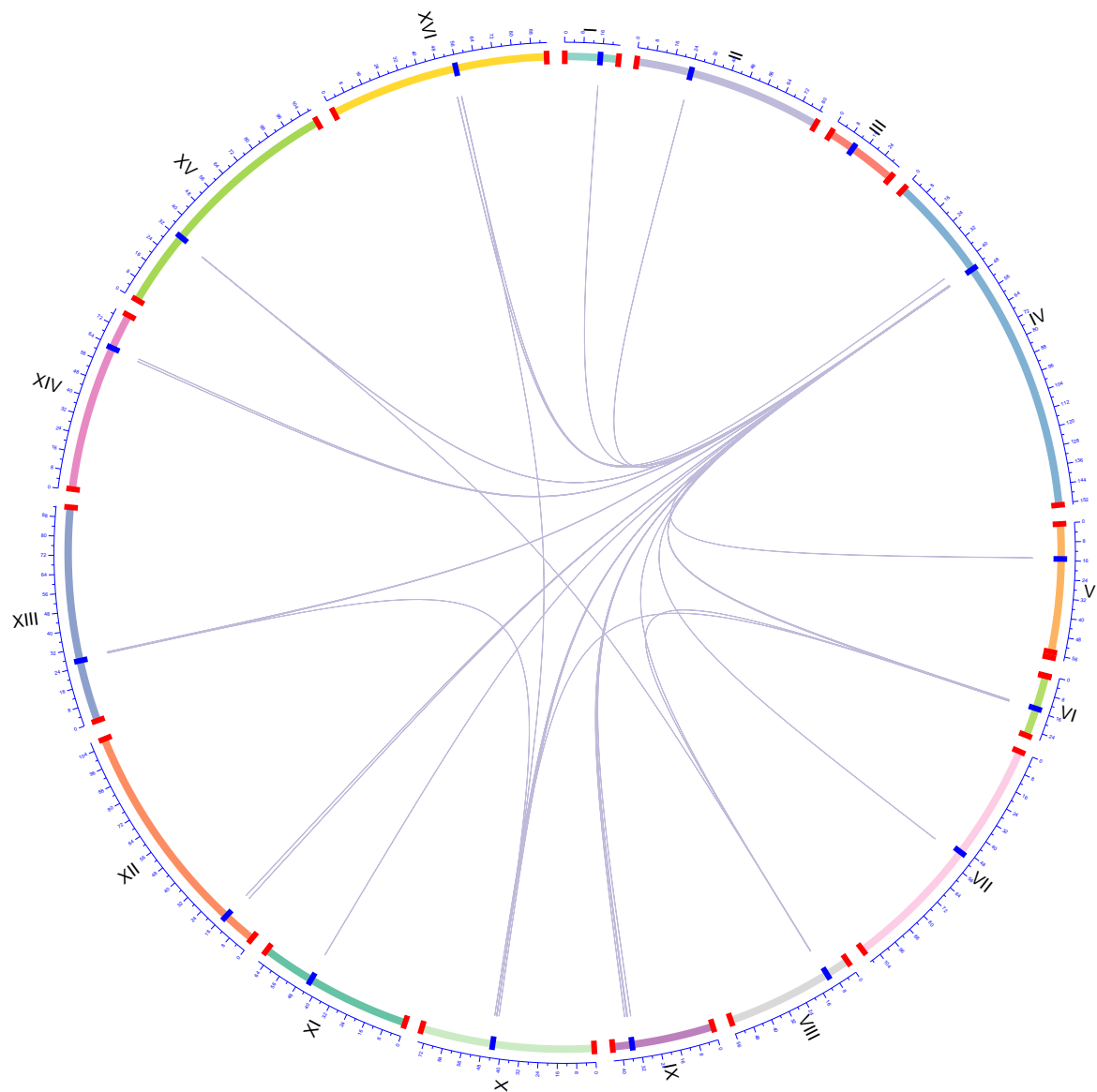

Supplement: Supplementary file 4 — A folder named SB-06-S4 contains circos diagrams for 10 significant TFs at two conditions (T = 20, T = 40). (ZIP 2300 kb) [file 12918_2018_643_MOESM4_ESM.zip › SB-06-S4/CIN5_40.pdf]

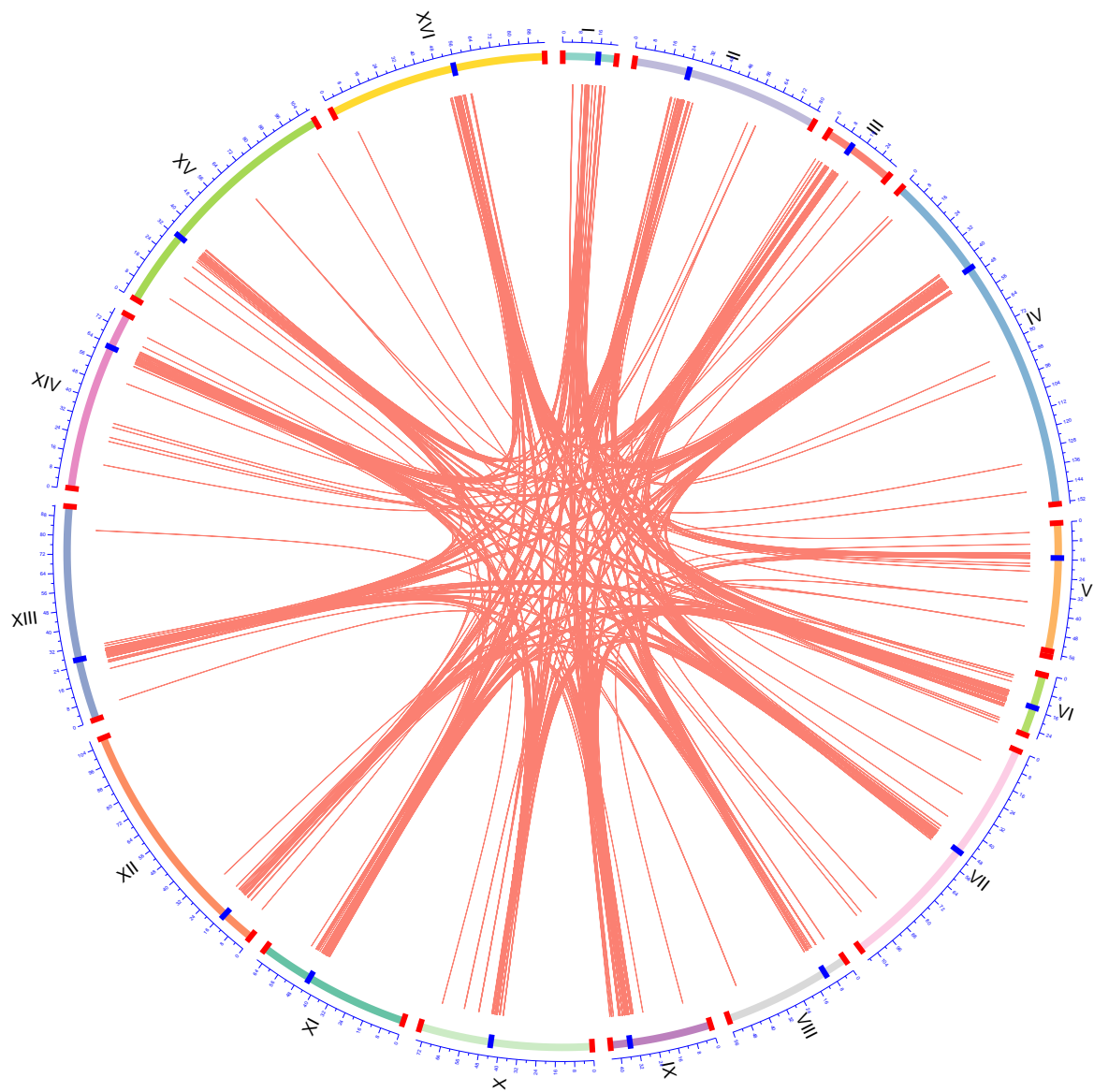

Supplement: Supplementary file 4 — A folder named SB-06-S4 contains circos diagrams for 10 significant TFs at two conditions (T = 20, T = 40). (ZIP 2300 kb) [file 12918_2018_643_MOESM4_ESM.zip › SB-06-S4/DIG1_20.pdf]

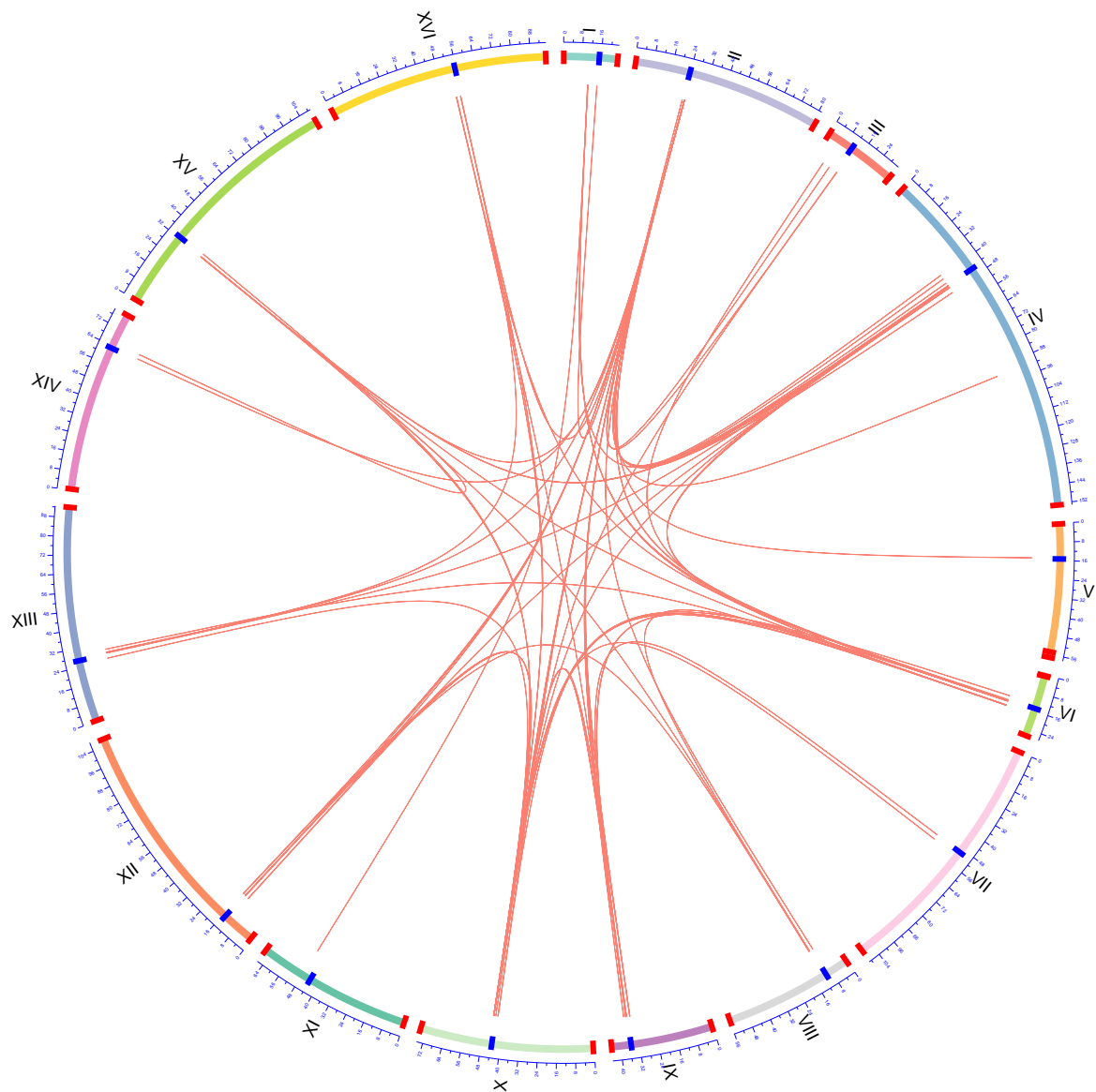

Supplement: Supplementary file 4 — A folder named SB-06-S4 contains circos diagrams for 10 significant TFs at two conditions (T = 20, T = 40). (ZIP 2300 kb) [file 12918_2018_643_MOESM4_ESM.zip › SB-06-S4/DIG1_40.pdf]

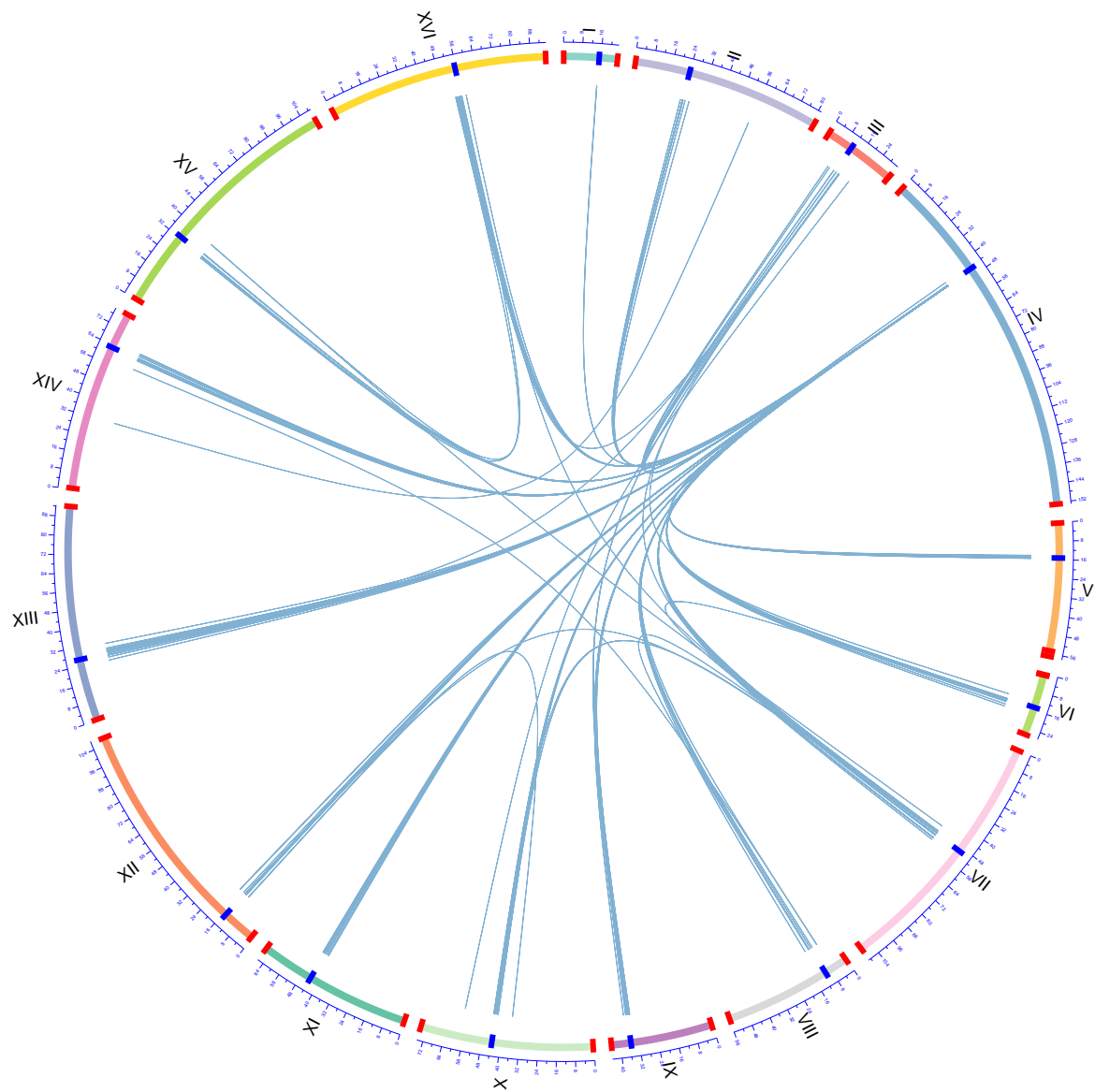

Supplement: Supplementary file 4 — A folder named SB-06-S4 contains circos diagrams for 10 significant TFs at two conditions (T = 20, T = 40). (ZIP 2300 kb) [file 12918_2018_643_MOESM4_ESM.zip › SB-06-S4/HSF1_20.pdf]

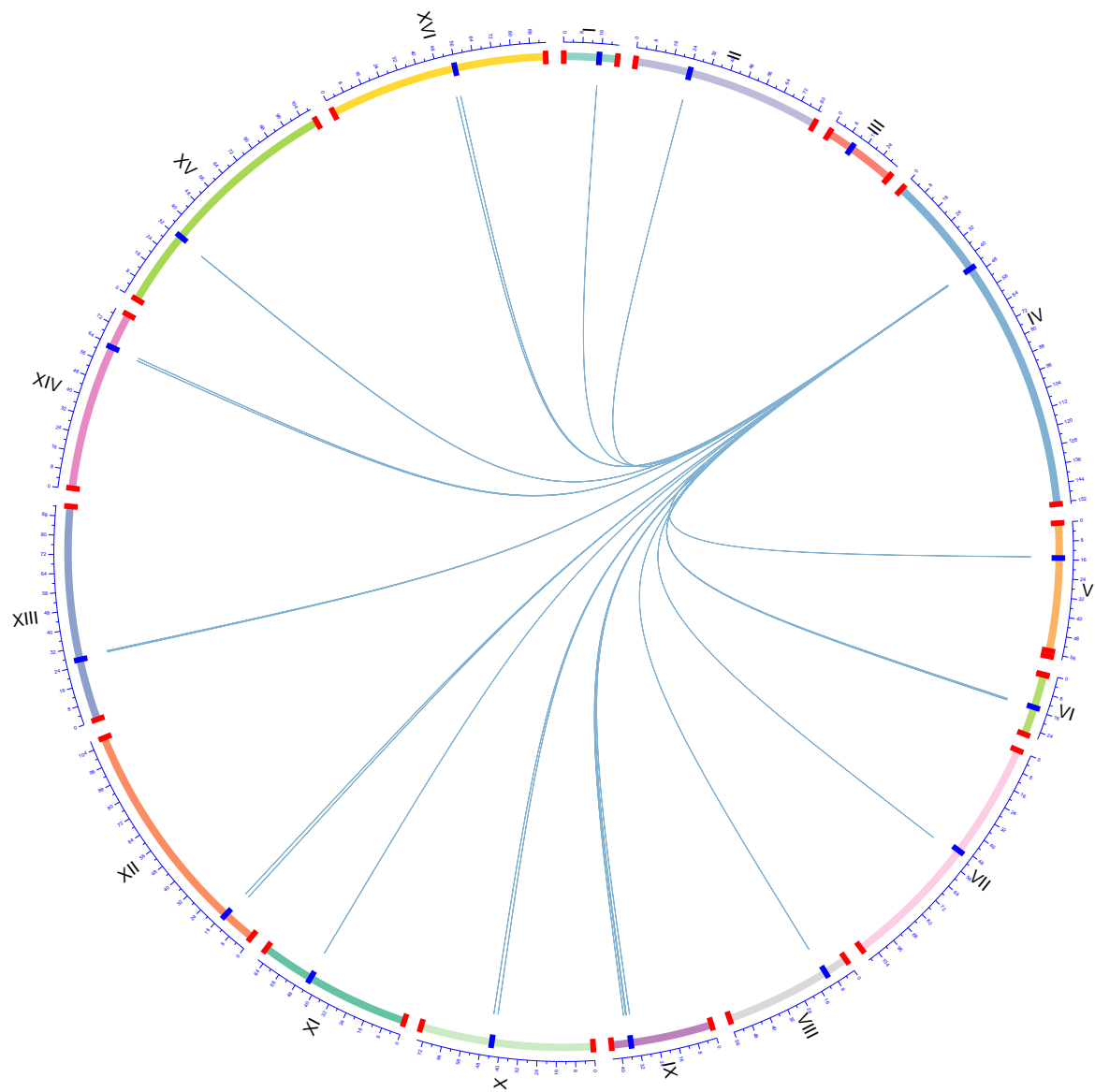

Supplement: Supplementary file 4 — A folder named SB-06-S4 contains circos diagrams for 10 significant TFs at two conditions (T = 20, T = 40). (ZIP 2300 kb) [file 12918_2018_643_MOESM4_ESM.zip › SB-06-S4/HSF1_40.pdf]

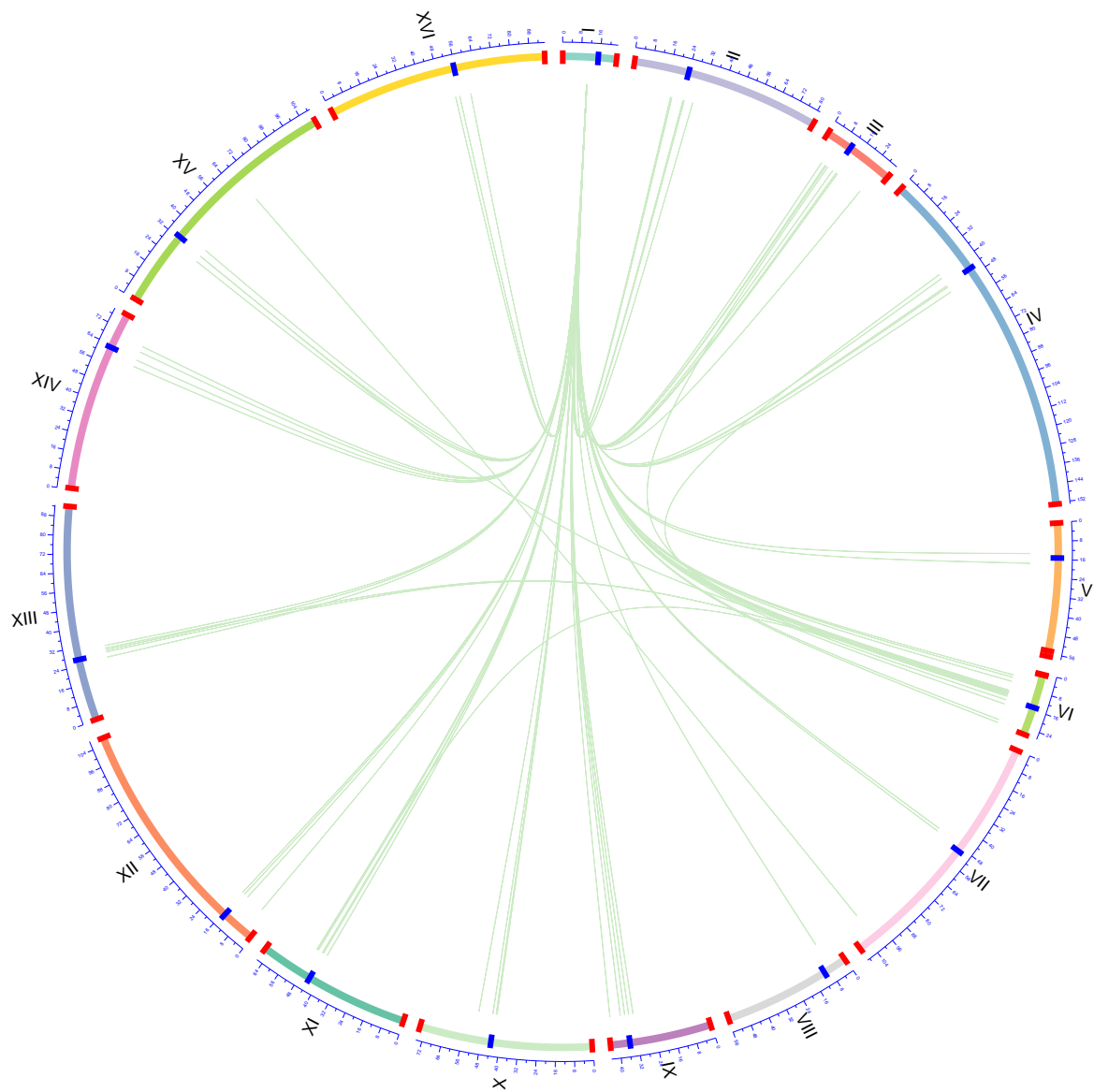

Supplement: Supplementary file 4 — A folder named SB-06-S4 contains circos diagrams for 10 significant TFs at two conditions (T = 20, T = 40). (ZIP 2300 kb) [file 12918_2018_643_MOESM4_ESM.zip › SB-06-S4/OPI1_20.pdf]

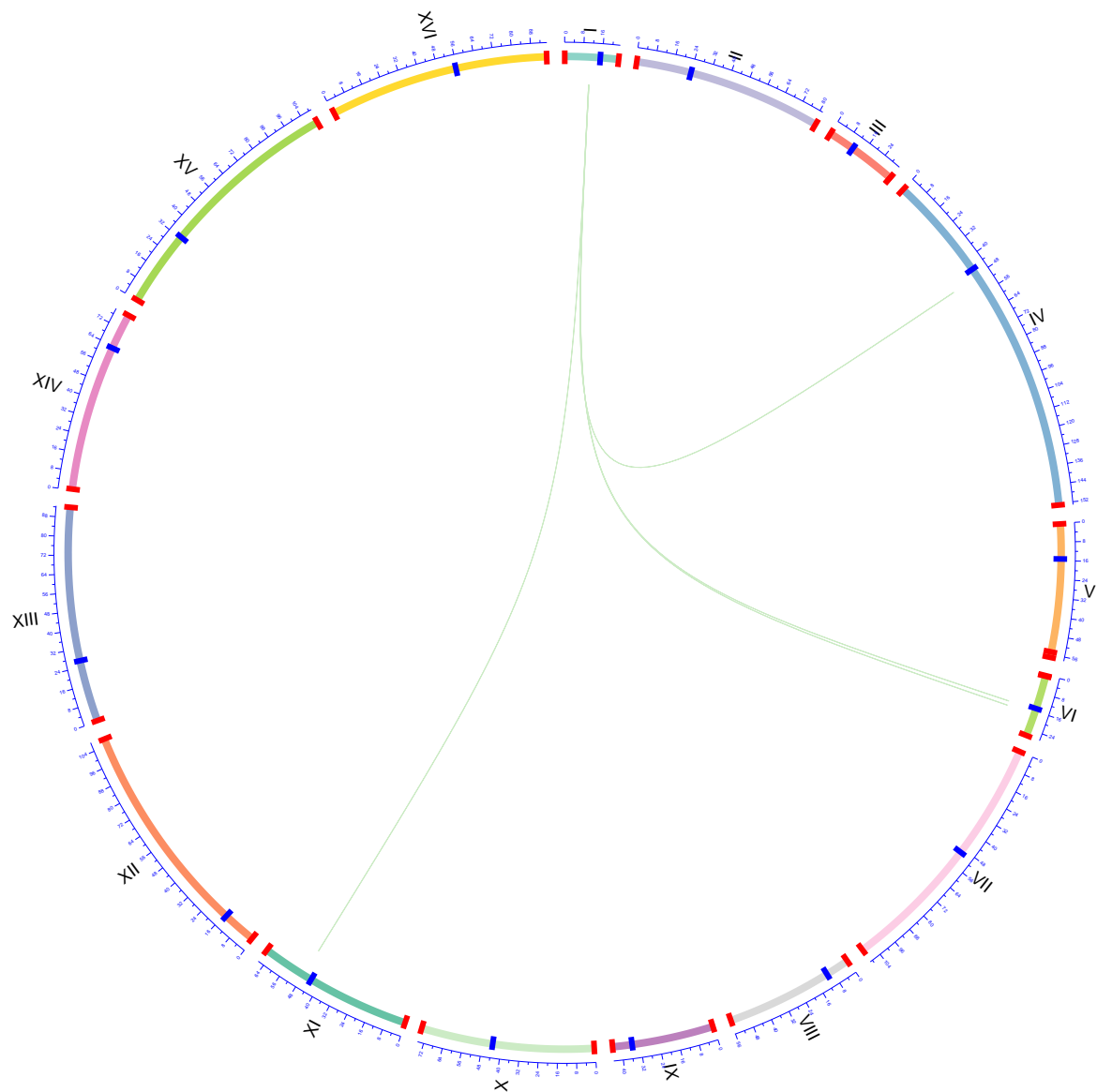

Supplement: Supplementary file 4 — A folder named SB-06-S4 contains circos diagrams for 10 significant TFs at two conditions (T = 20, T = 40). (ZIP 2300 kb) [file 12918_2018_643_MOESM4_ESM.zip › SB-06-S4/OPI1_40.pdf]

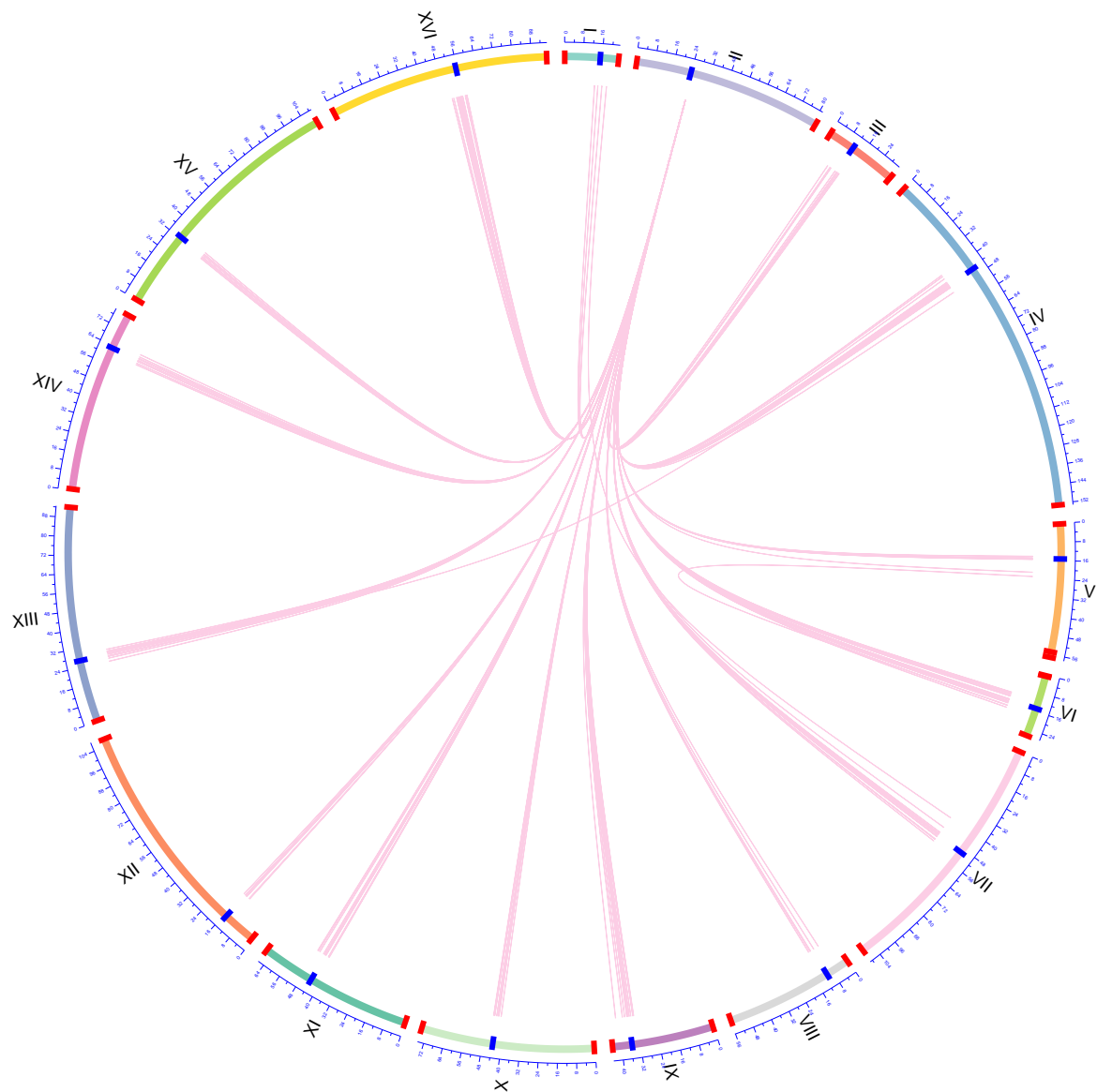

Supplement: Supplementary file 4 — A folder named SB-06-S4 contains circos diagrams for 10 significant TFs at two conditions (T = 20, T = 40). (ZIP 2300 kb) [file 12918_2018_643_MOESM4_ESM.zip › SB-06-S4/RLR1_20.pdf]

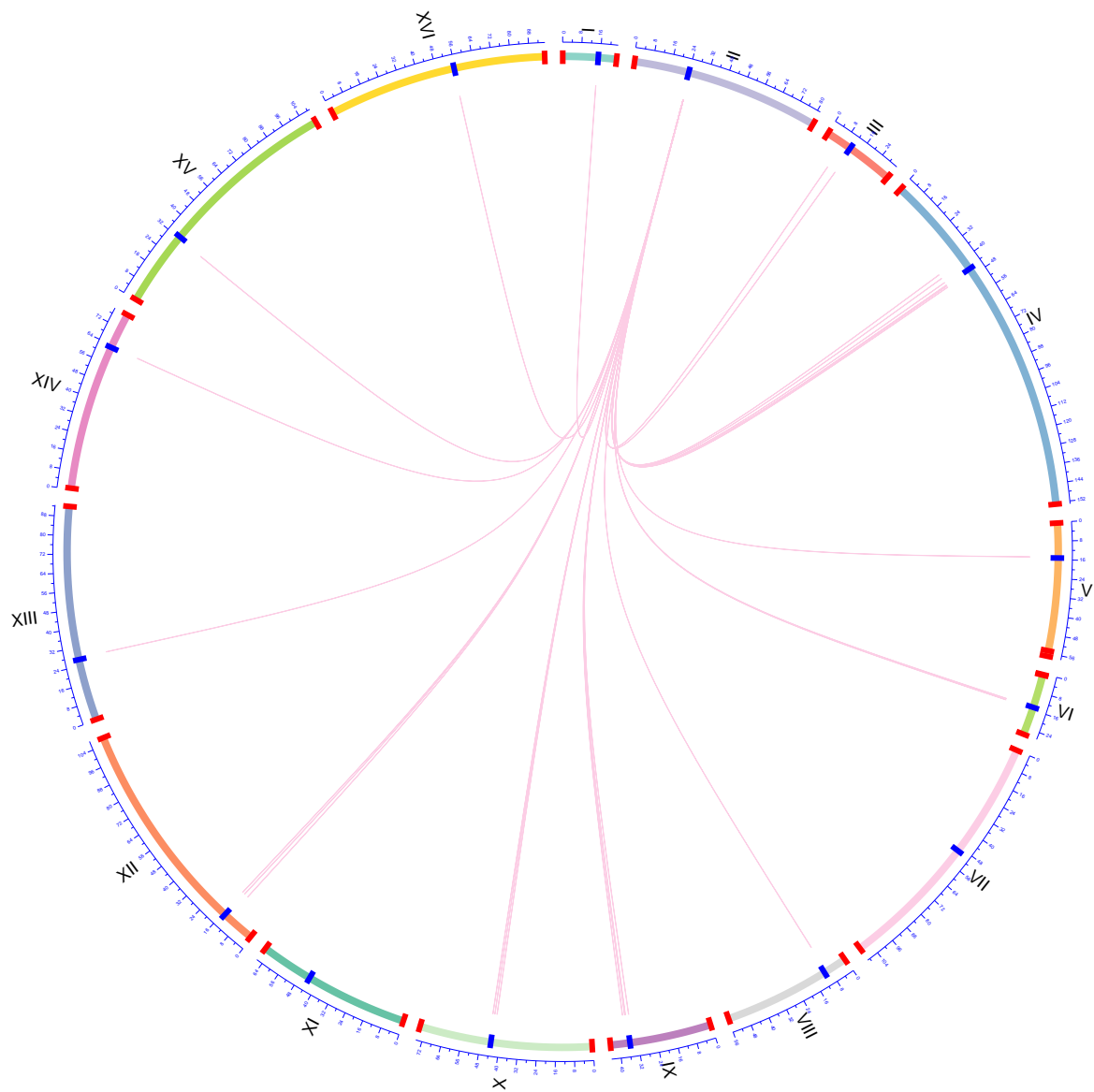

Supplement: Supplementary file 4 — A folder named SB-06-S4 contains circos diagrams for 10 significant TFs at two conditions (T = 20, T = 40). (ZIP 2300 kb) [file 12918_2018_643_MOESM4_ESM.zip › SB-06-S4/RLR1_40.pdf]

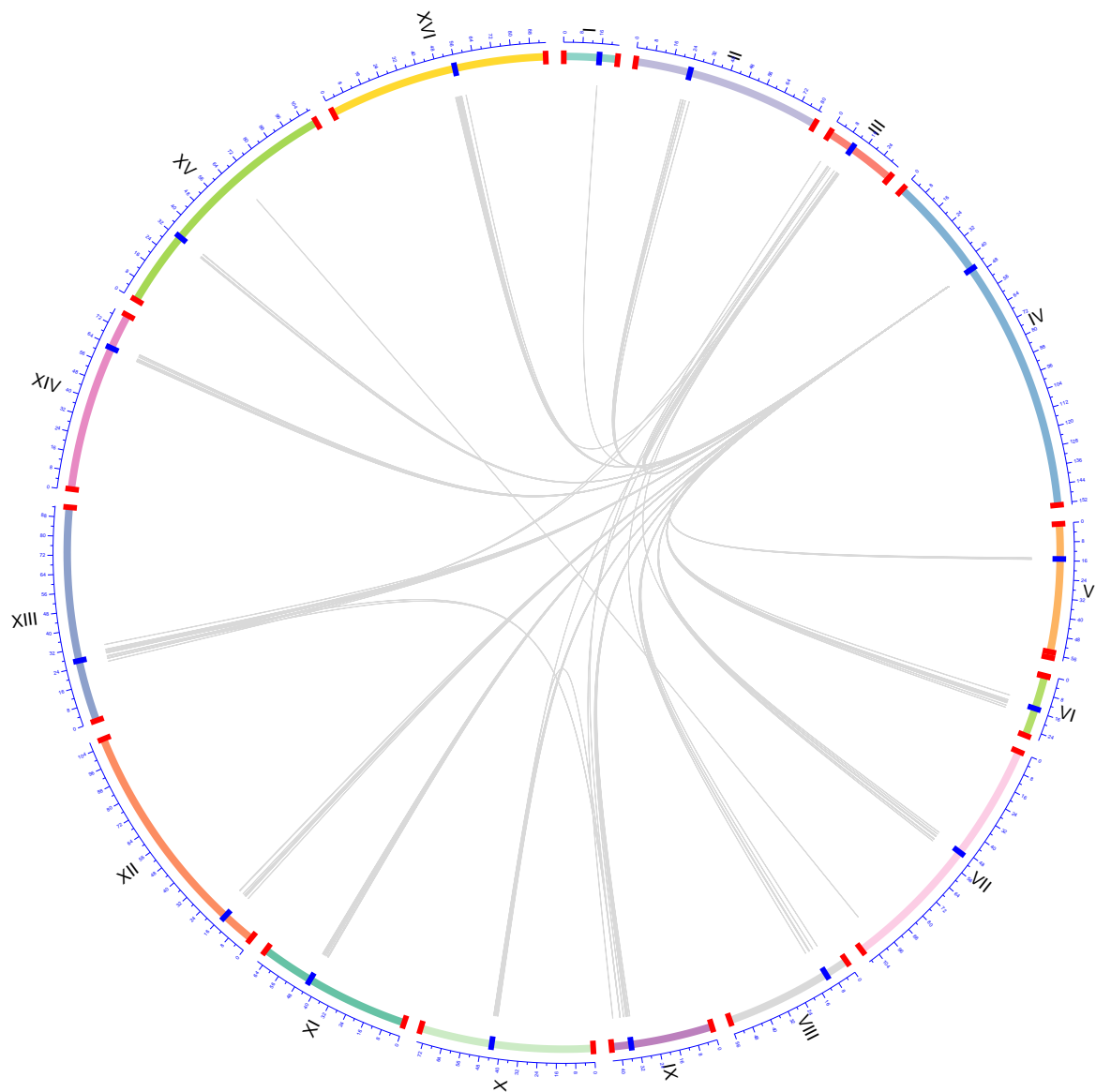

Supplement: Supplementary file 4 — A folder named SB-06-S4 contains circos diagrams for 10 significant TFs at two conditions (T = 20, T = 40). (ZIP 2300 kb) [file 12918_2018_643_MOESM4_ESM.zip › SB-06-S4/SKO1_20.pdf]

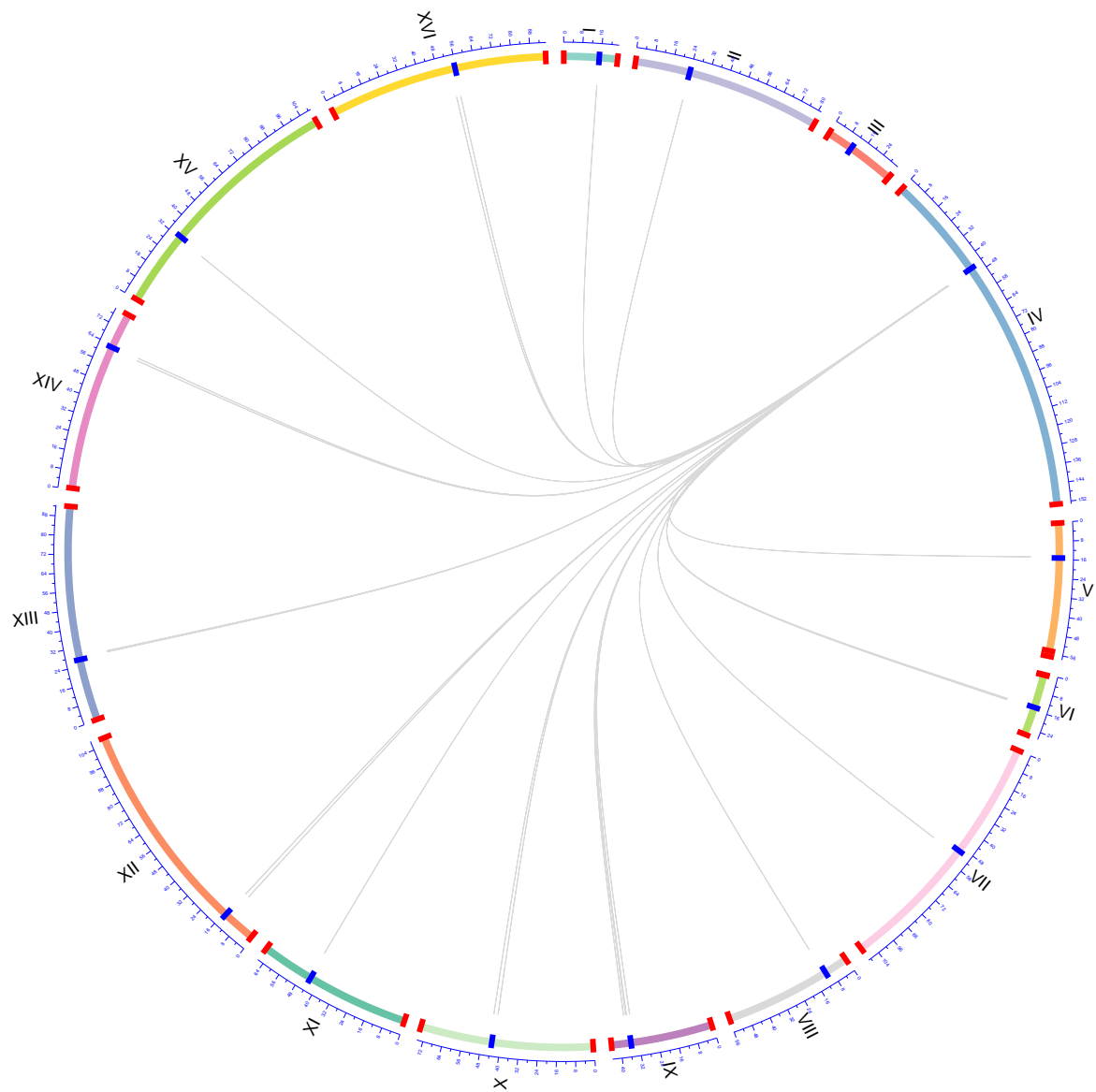

Supplement: Supplementary file 4 — A folder named SB-06-S4 contains circos diagrams for 10 significant TFs at two conditions (T = 20, T = 40). (ZIP 2300 kb) [file 12918_2018_643_MOESM4_ESM.zip › SB-06-S4/SKO1_40.pdf]

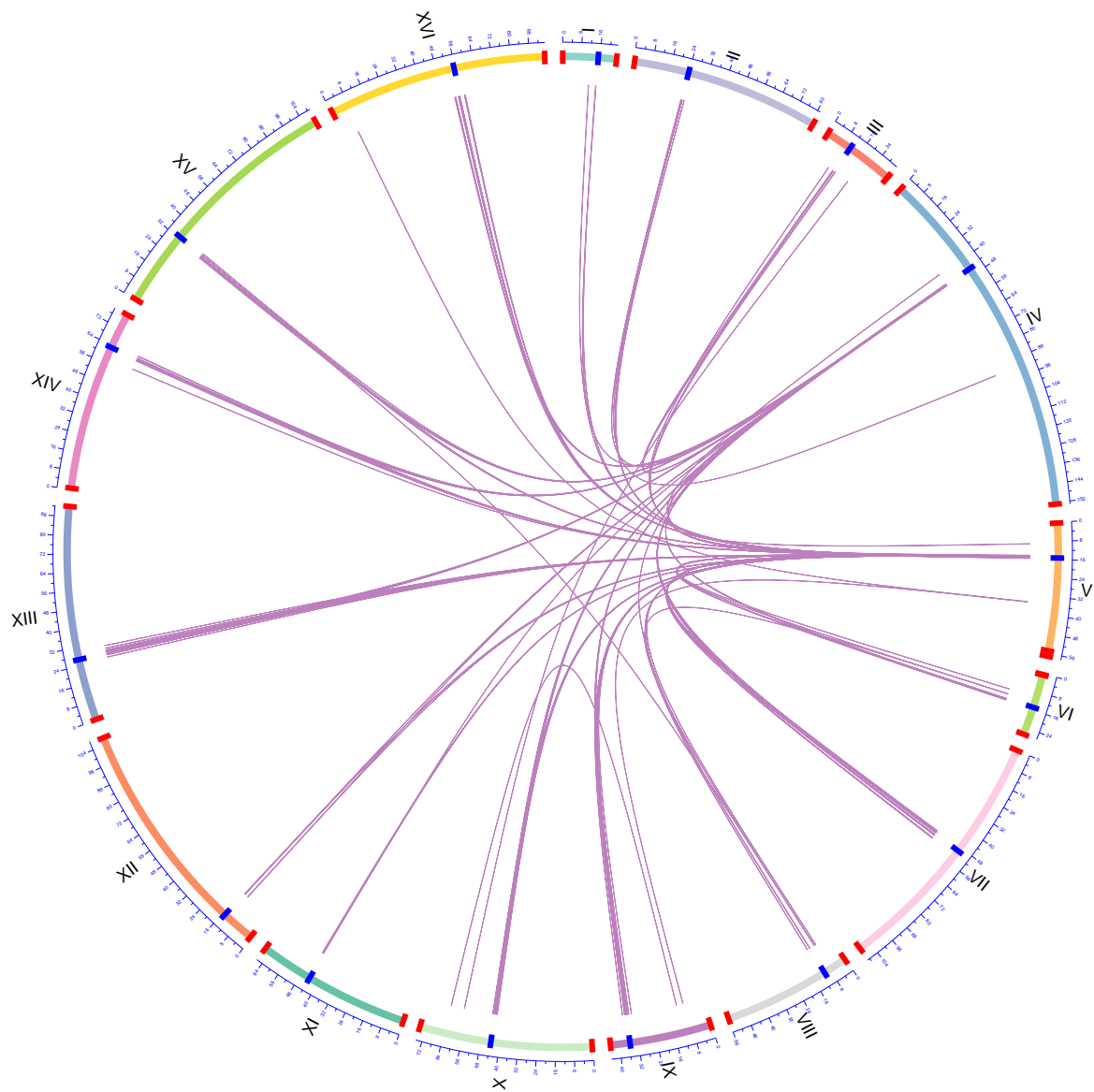

Supplement: Supplementary file 4 — A folder named SB-06-S4 contains circos diagrams for 10 significant TFs at two conditions (T = 20, T = 40). (ZIP 2300 kb) [file 12918_2018_643_MOESM4_ESM.zip › SB-06-S4/SPT2_20.pdf]

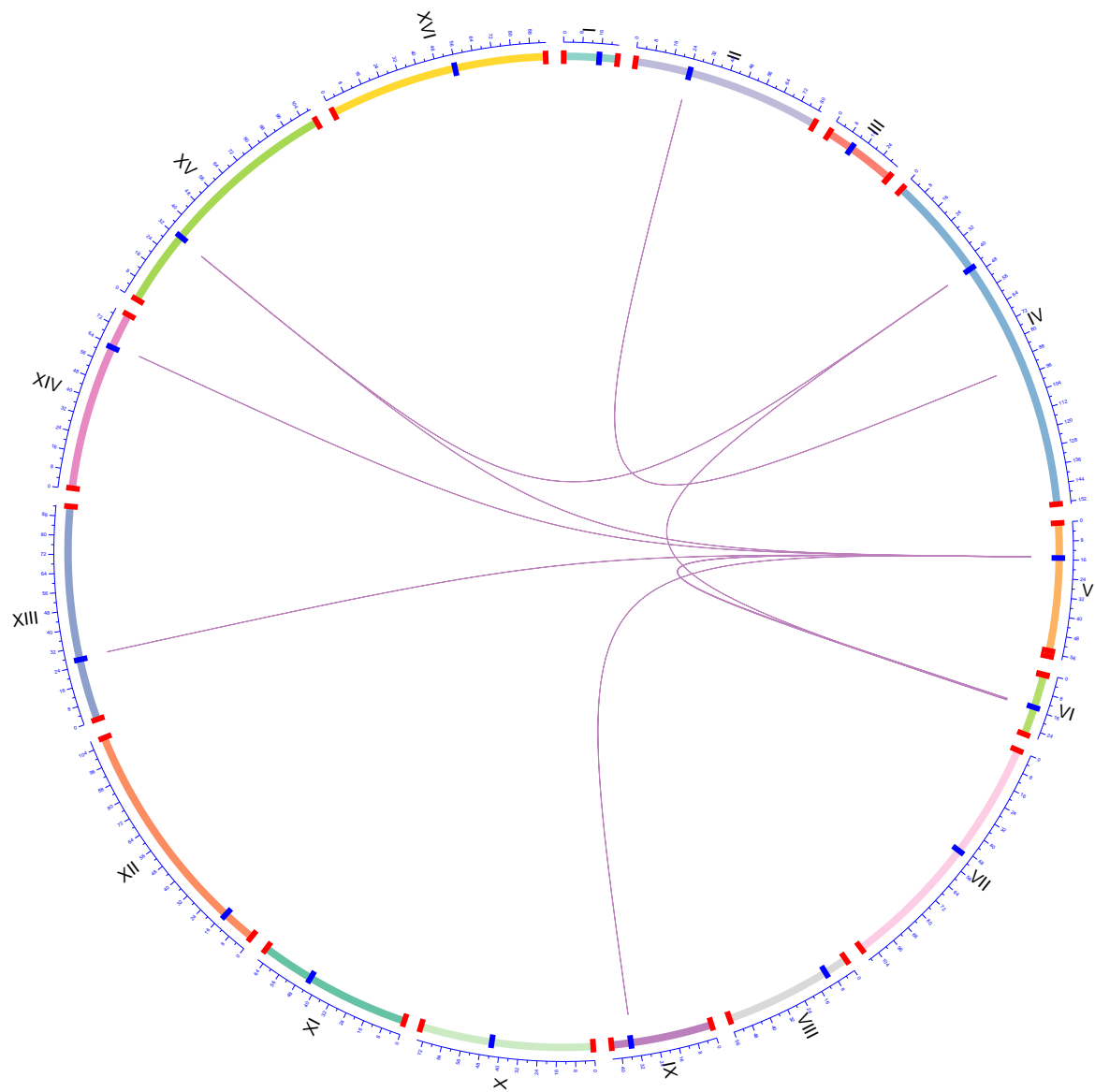

Supplement: Supplementary file 4 — A folder named SB-06-S4 contains circos diagrams for 10 significant TFs at two conditions (T = 20, T = 40). (ZIP 2300 kb) [file 12918_2018_643_MOESM4_ESM.zip › SB-06-S4/SPT2_40.pdf]

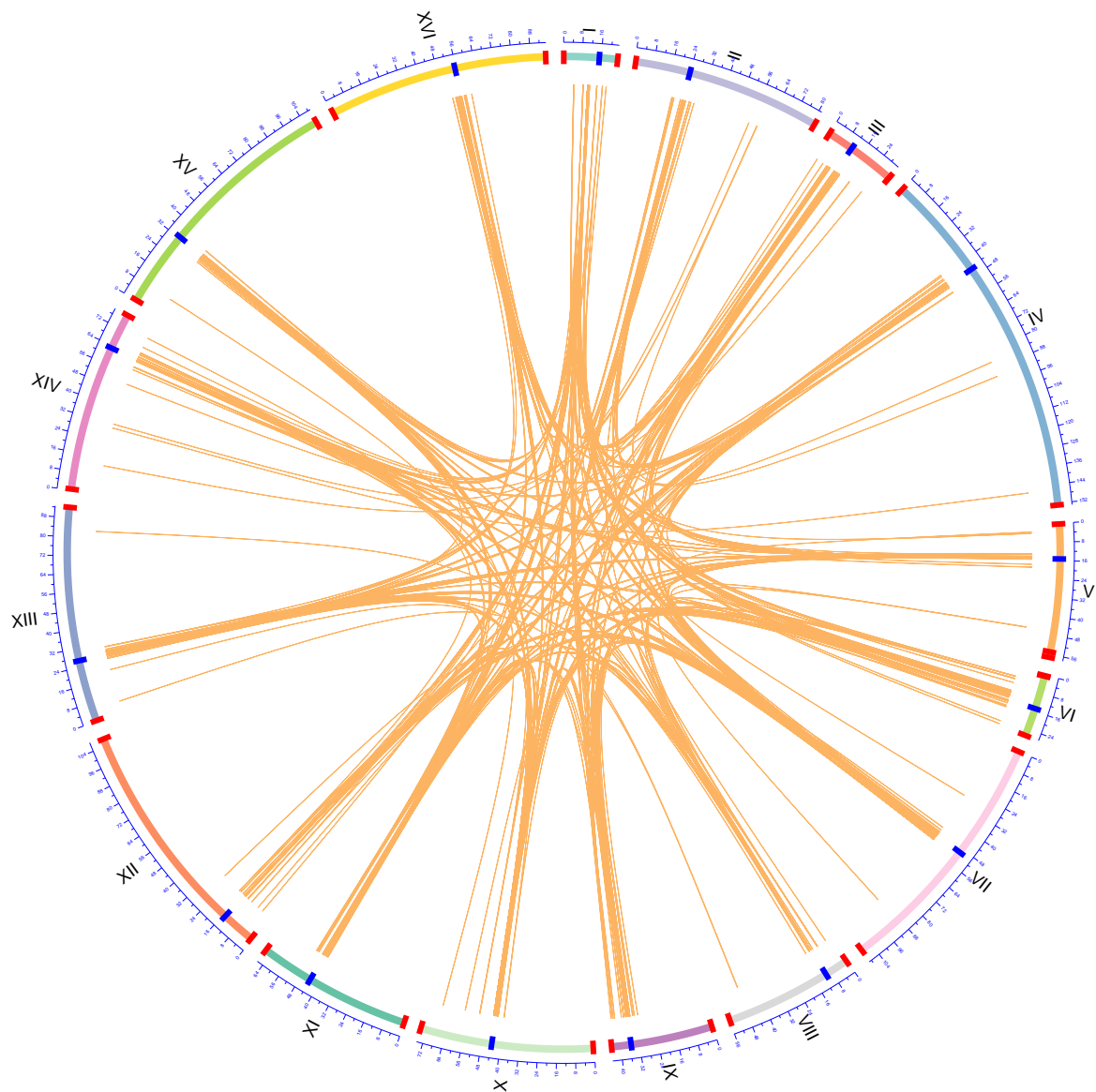

Supplement: Supplementary file 4 — A folder named SB-06-S4 contains circos diagrams for 10 significant TFs at two conditions (T = 20, T = 40). (ZIP 2300 kb) [file 12918_2018_643_MOESM4_ESM.zip › SB-06-S4/STE12_20.pdf]

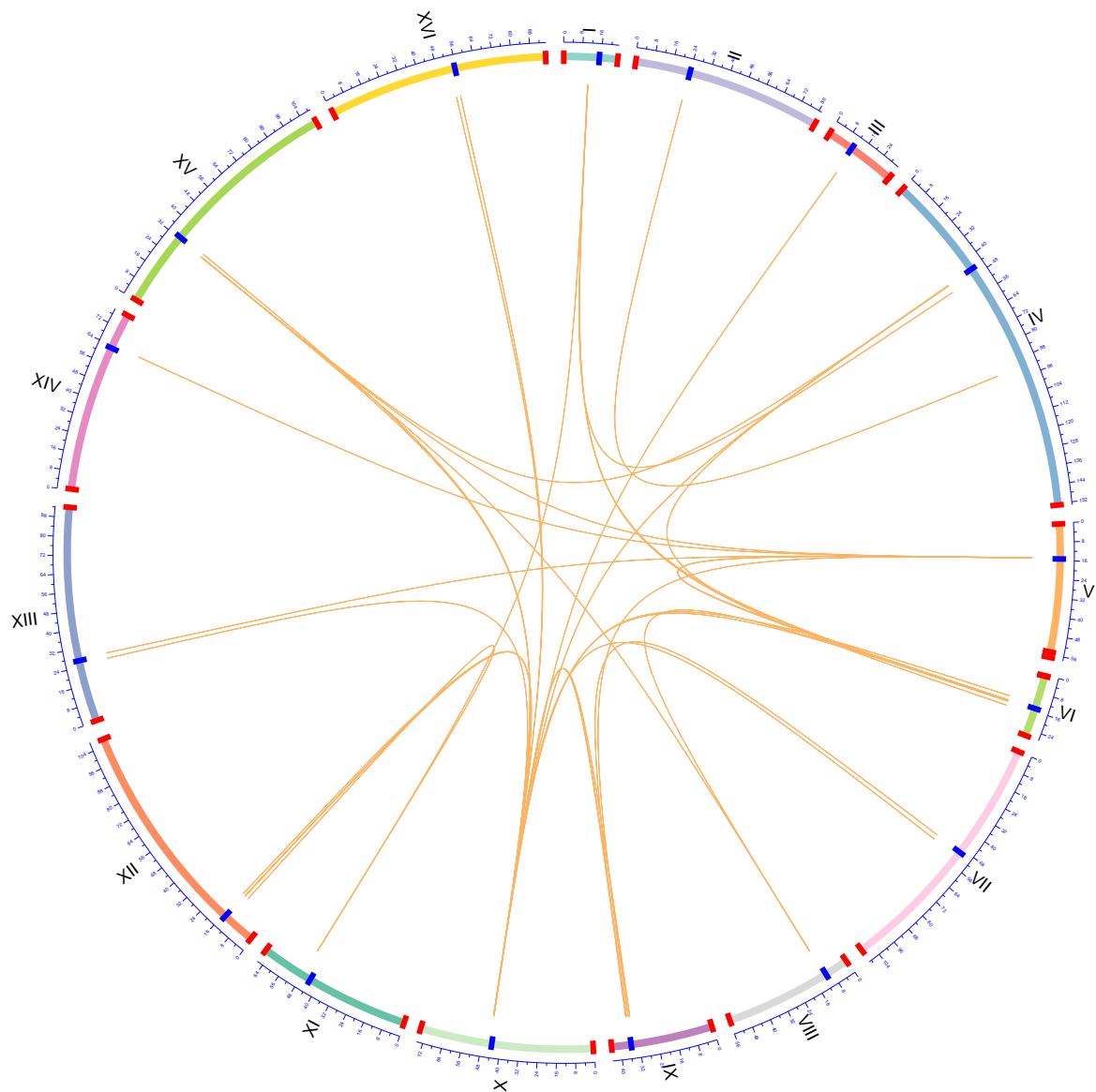

Supplement: Supplementary file 4 — A folder named SB-06-S4 contains circos diagrams for 10 significant TFs at two conditions (T = 20, T = 40). (ZIP 2300 kb) [file 12918_2018_643_MOESM4_ESM.zip › SB-06-S4/STE12_40.pdf]

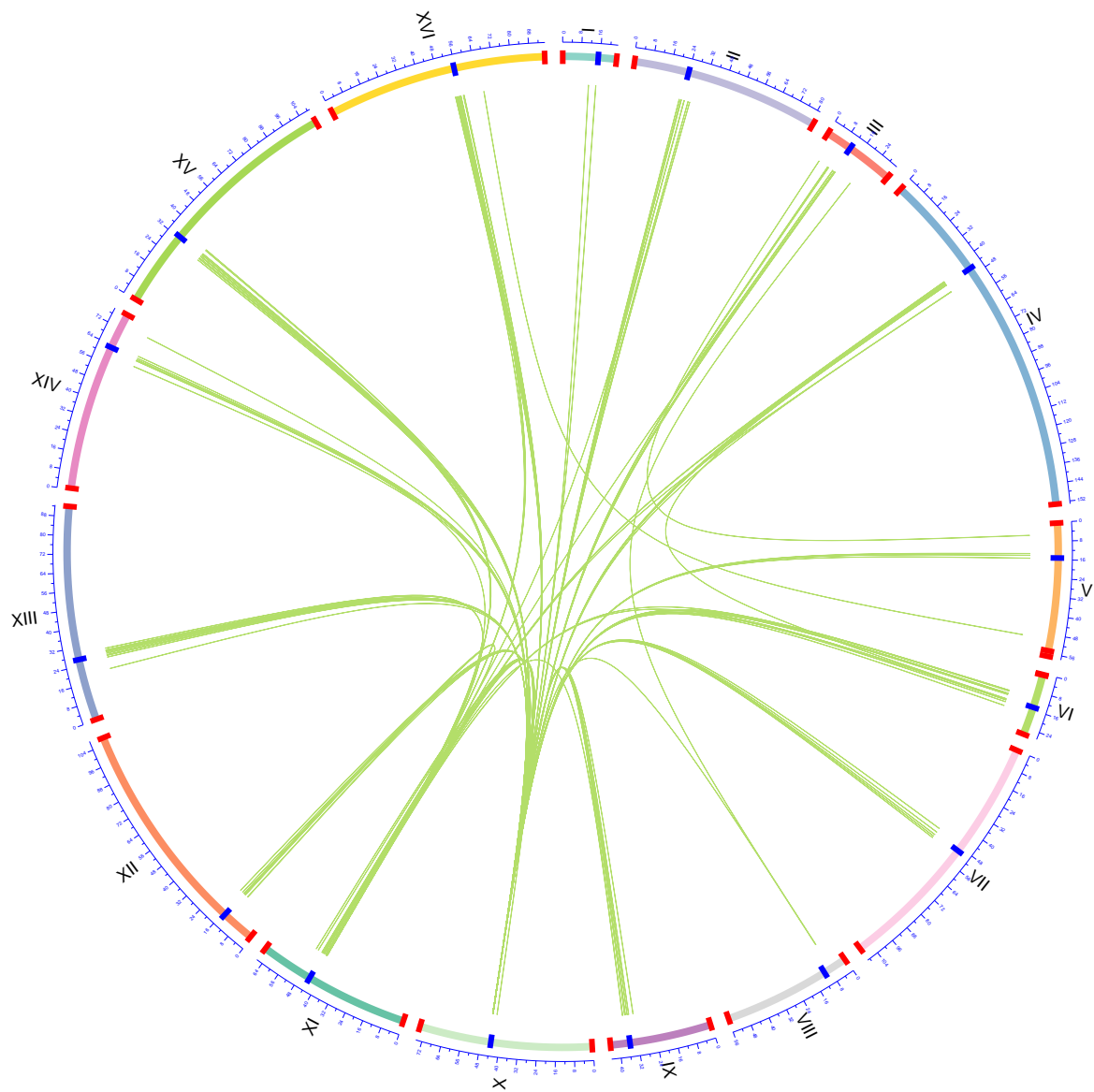

Supplement: Supplementary file 4 — A folder named SB-06-S4 contains circos diagrams for 10 significant TFs at two conditions (T = 20, T = 40). (ZIP 2300 kb) [file 12918_2018_643_MOESM4_ESM.zip › SB-06-S4/YDR026c_20.pdf]

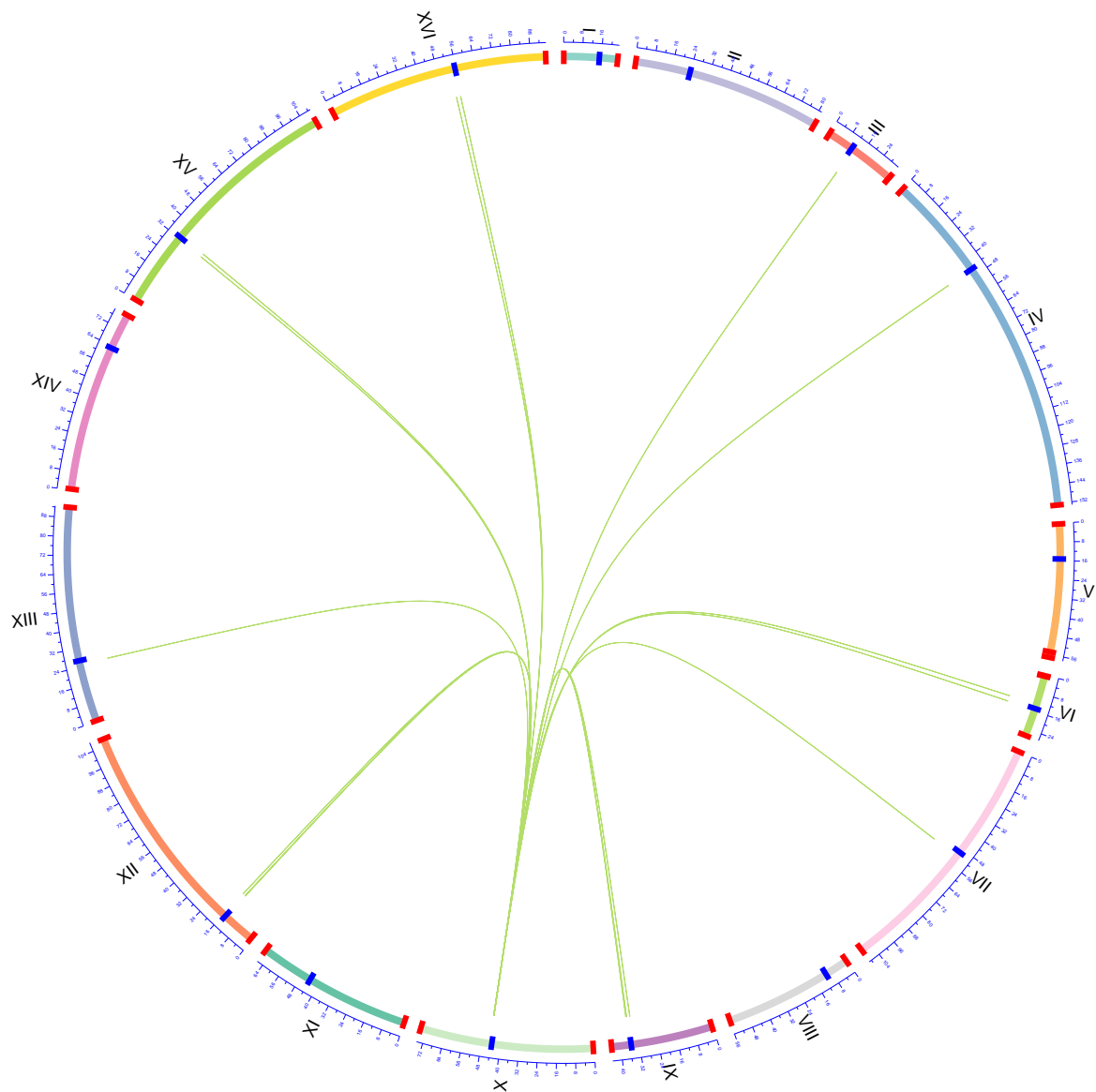

Supplement: Supplementary file 4 — A folder named SB-06-S4 contains circos diagrams for 10 significant TFs at two conditions (T = 20, T = 40). (ZIP 2300 kb) [file 12918_2018_643_MOESM4_ESM.zip › SB-06-S4/YDR026c_40.pdf]
