# Supplementary figures and images for: Multiple transcription factors contribute to inter-chromosomal interaction in yeast (part 1 of 2)
Source: BMC Syst Biol. 2018 Dec 21;12(Suppl 8):140. doi: 10.1186/s12918-018-0643-1 (PMC6302461; doi:10.1186/s12918-018-0643-1)

# ABF1

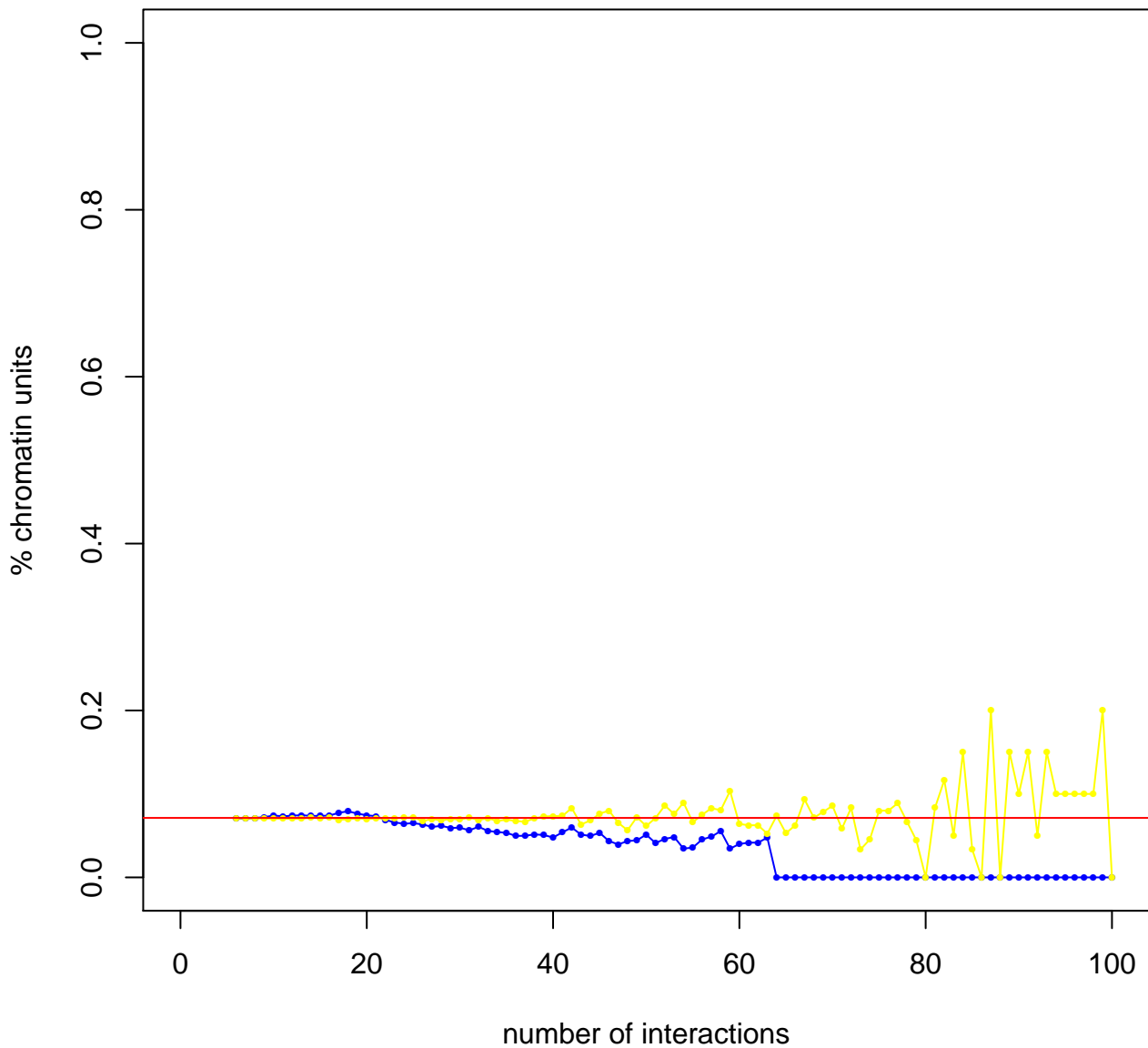

Supplement: Supplementary file 3 — A folder named SB-06-S3 contains 105 overlapping plot for each TF. (ZIP 624 kb) [file 12918_2018_643_MOESM3_ESM.zip › SB-06-S3/ABF1.pdf]

# ACE2

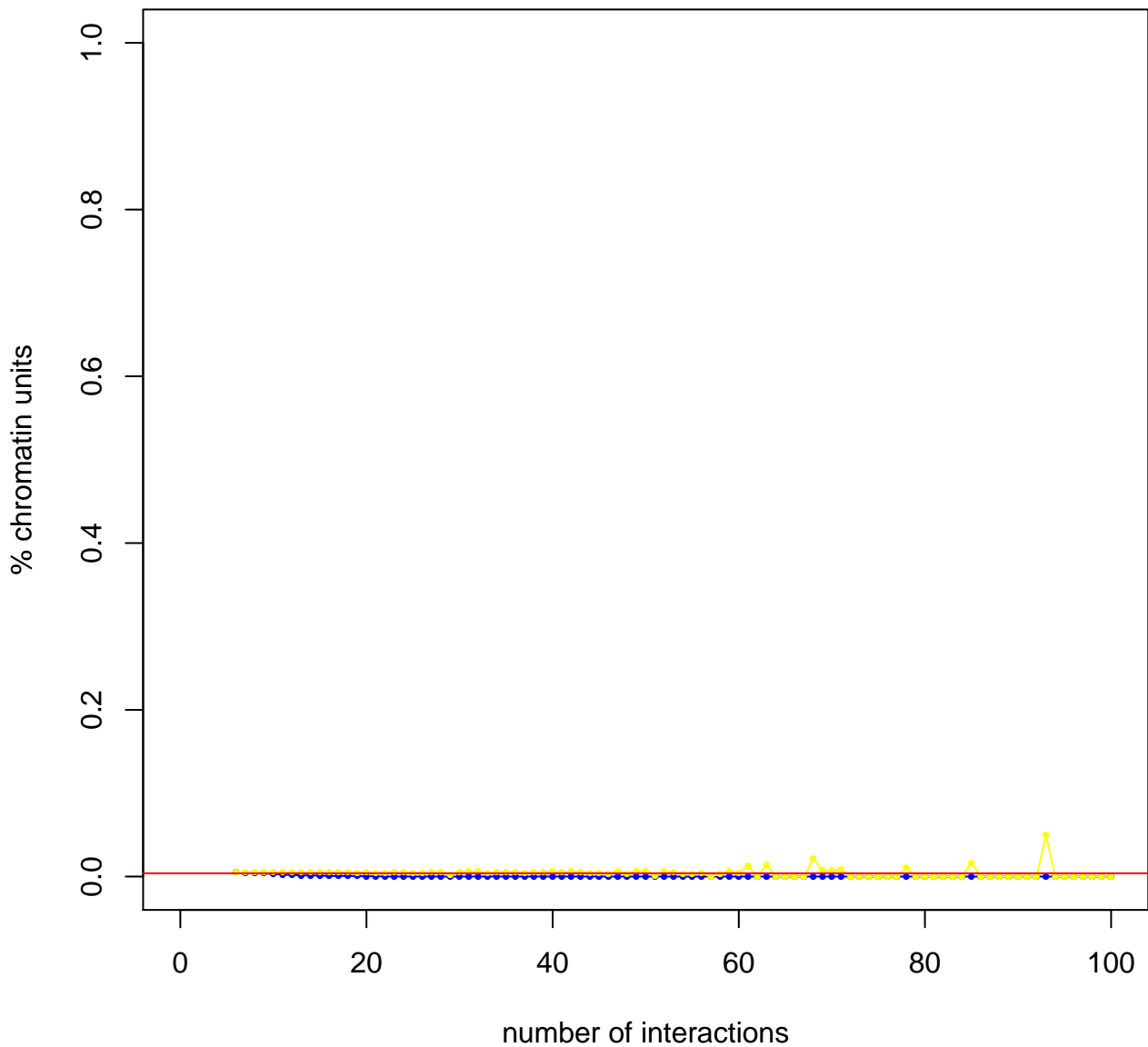

Supplement: Supplementary file 3 — A folder named SB-06-S3 contains 105 overlapping plot for each TF. (ZIP 624 kb) [file 12918_2018_643_MOESM3_ESM.zip › SB-06-S3/ACE2.pdf]

# ADR1

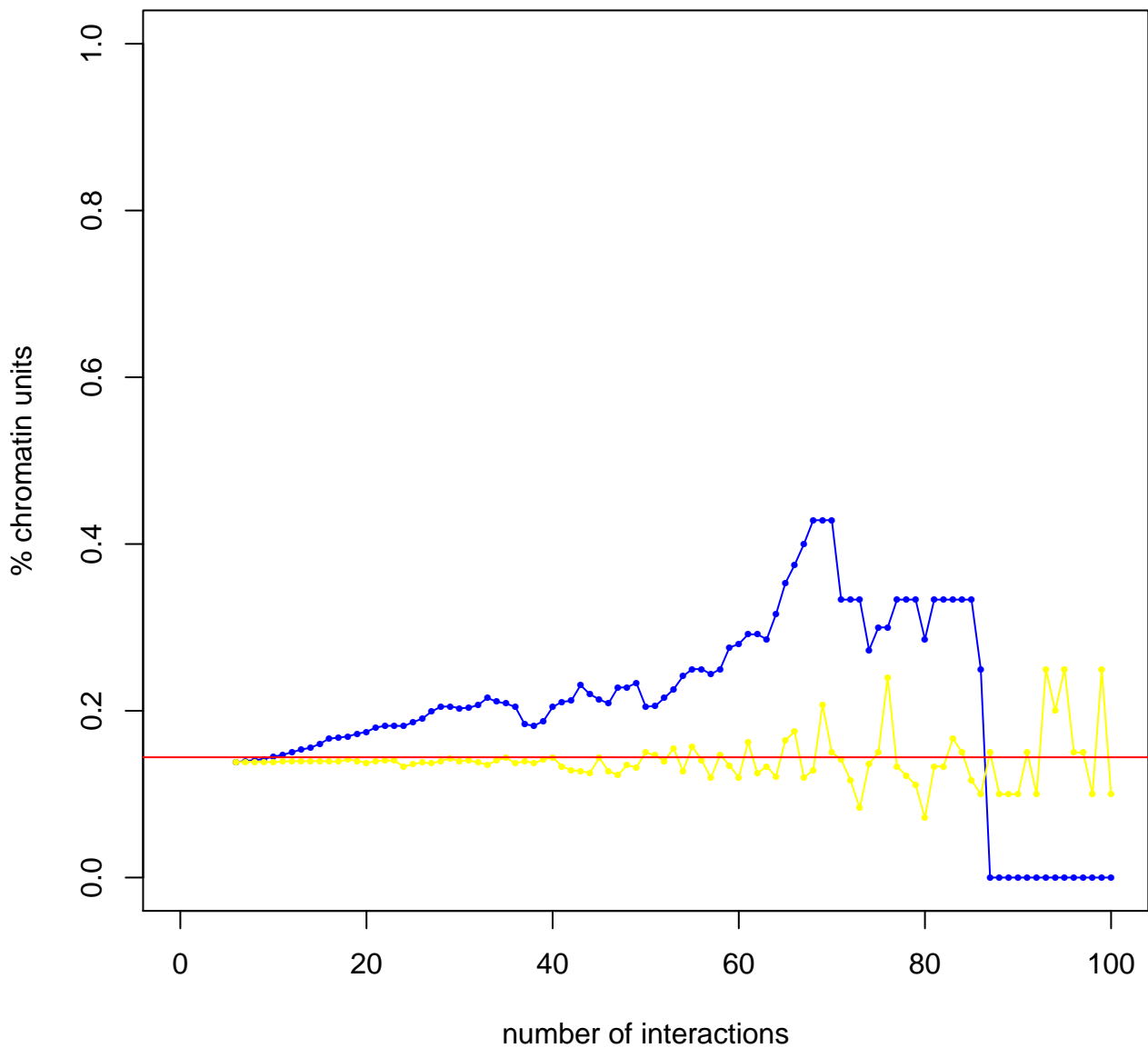

Supplement: Supplementary file 3 — A folder named SB-06-S3 contains 105 overlapping plot for each TF. (ZIP 624 kb) [file 12918_2018_643_MOESM3_ESM.zip › SB-06-S3/ADR1.pdf]

# AFT2

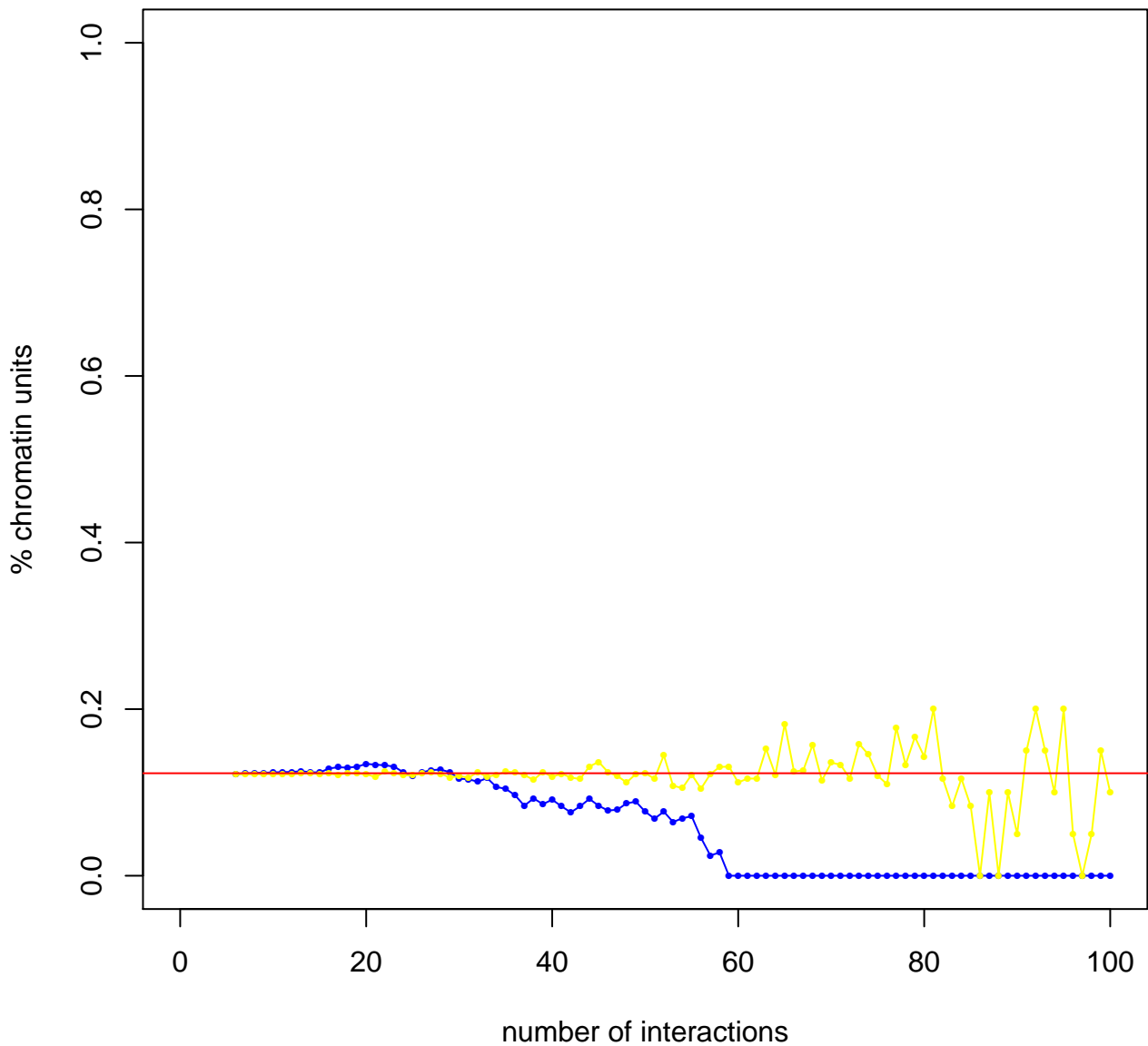

Supplement: Supplementary file 3 — A folder named SB-06-S3 contains 105 overlapping plot for each TF. (ZIP 624 kb) [file 12918_2018_643_MOESM3_ESM.zip › SB-06-S3/AFT2.pdf]

# ARR1

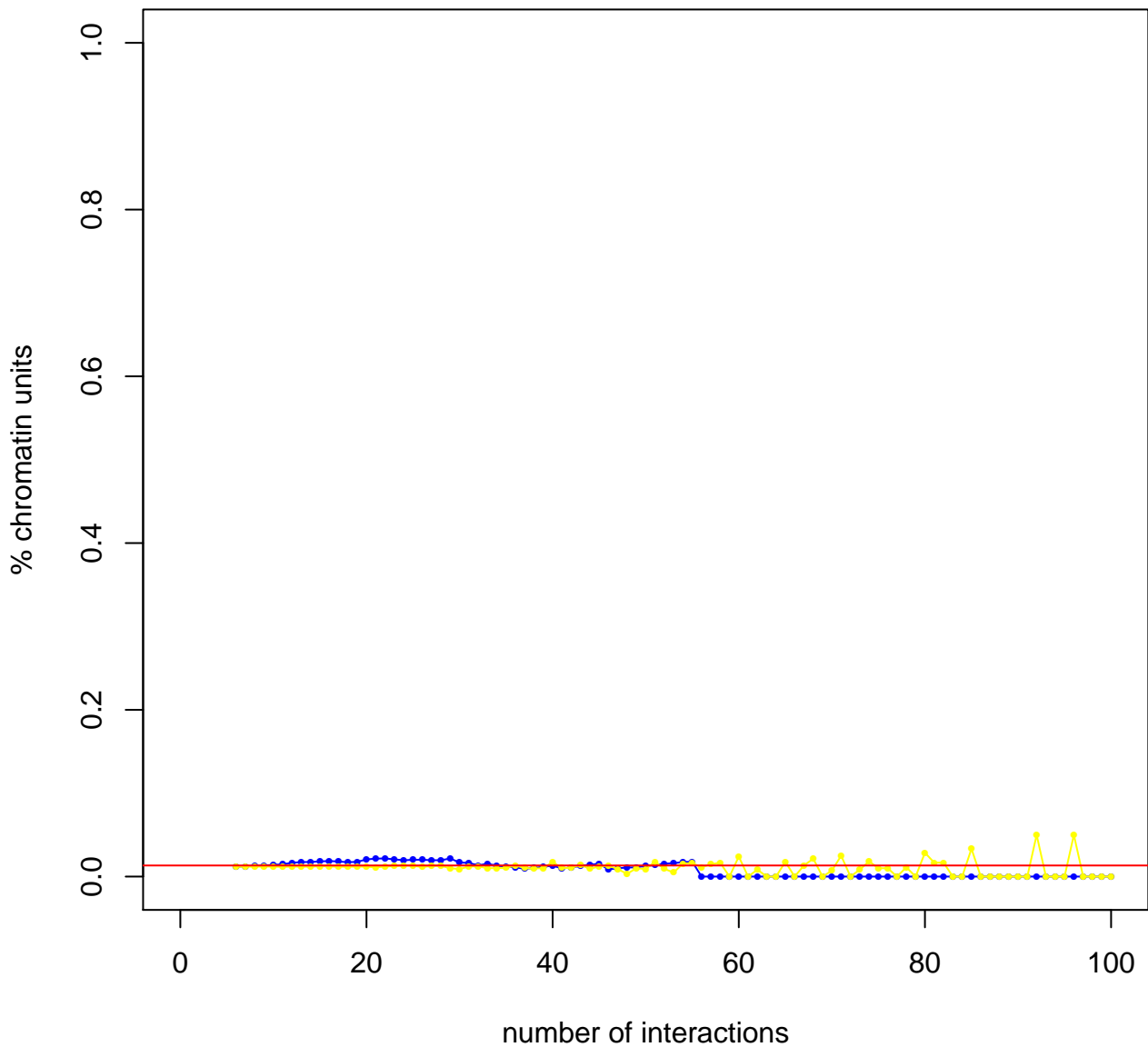

Supplement: Supplementary file 3 — A folder named SB-06-S3 contains 105 overlapping plot for each TF. (ZIP 624 kb) [file 12918_2018_643_MOESM3_ESM.zip › SB-06-S3/ARR1.pdf]

# ASH1

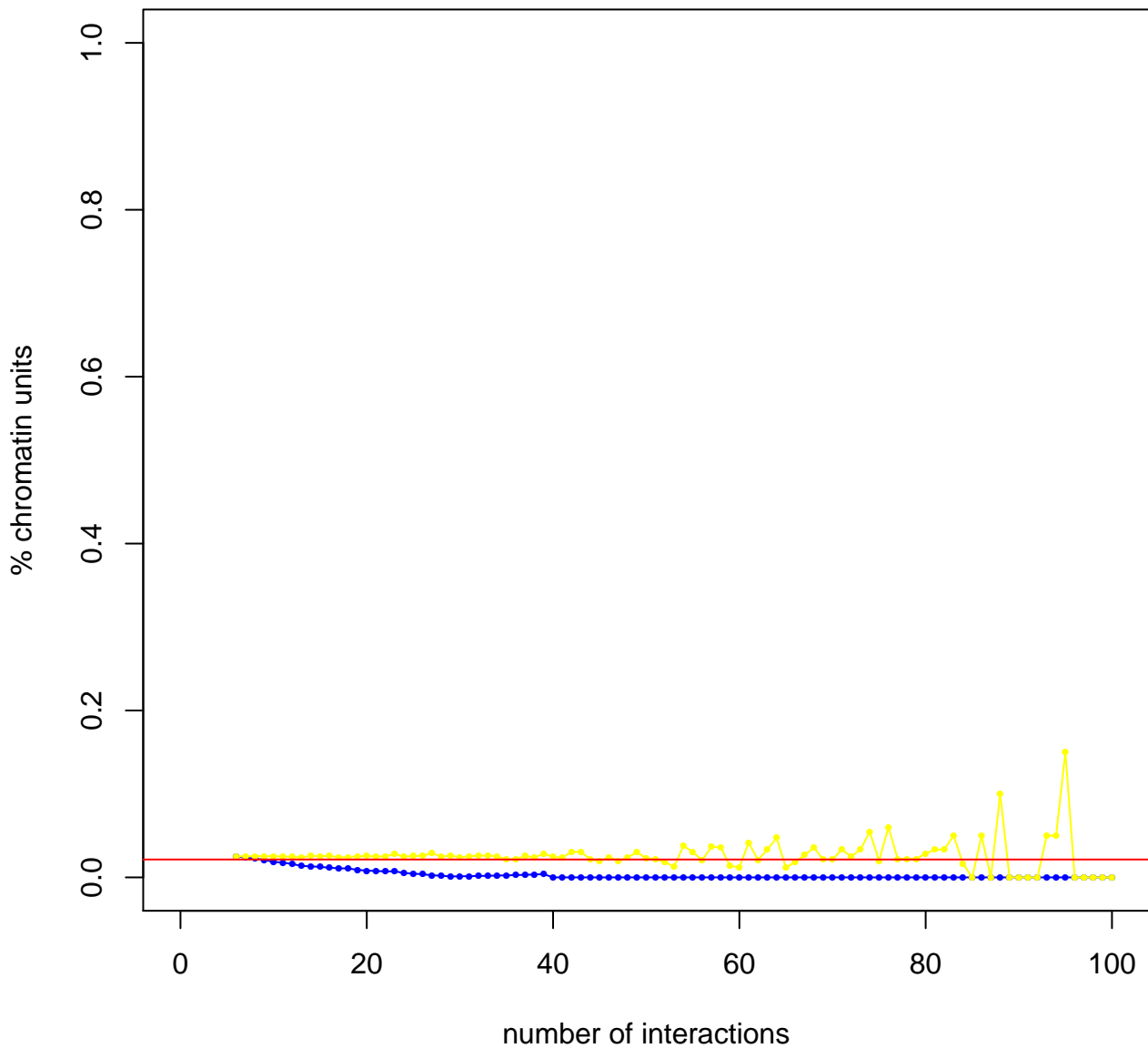

Supplement: Supplementary file 3 — A folder named SB-06-S3 contains 105 overlapping plot for each TF. (ZIP 624 kb) [file 12918_2018_643_MOESM3_ESM.zip › SB-06-S3/ASH1.pdf]

# AZF1

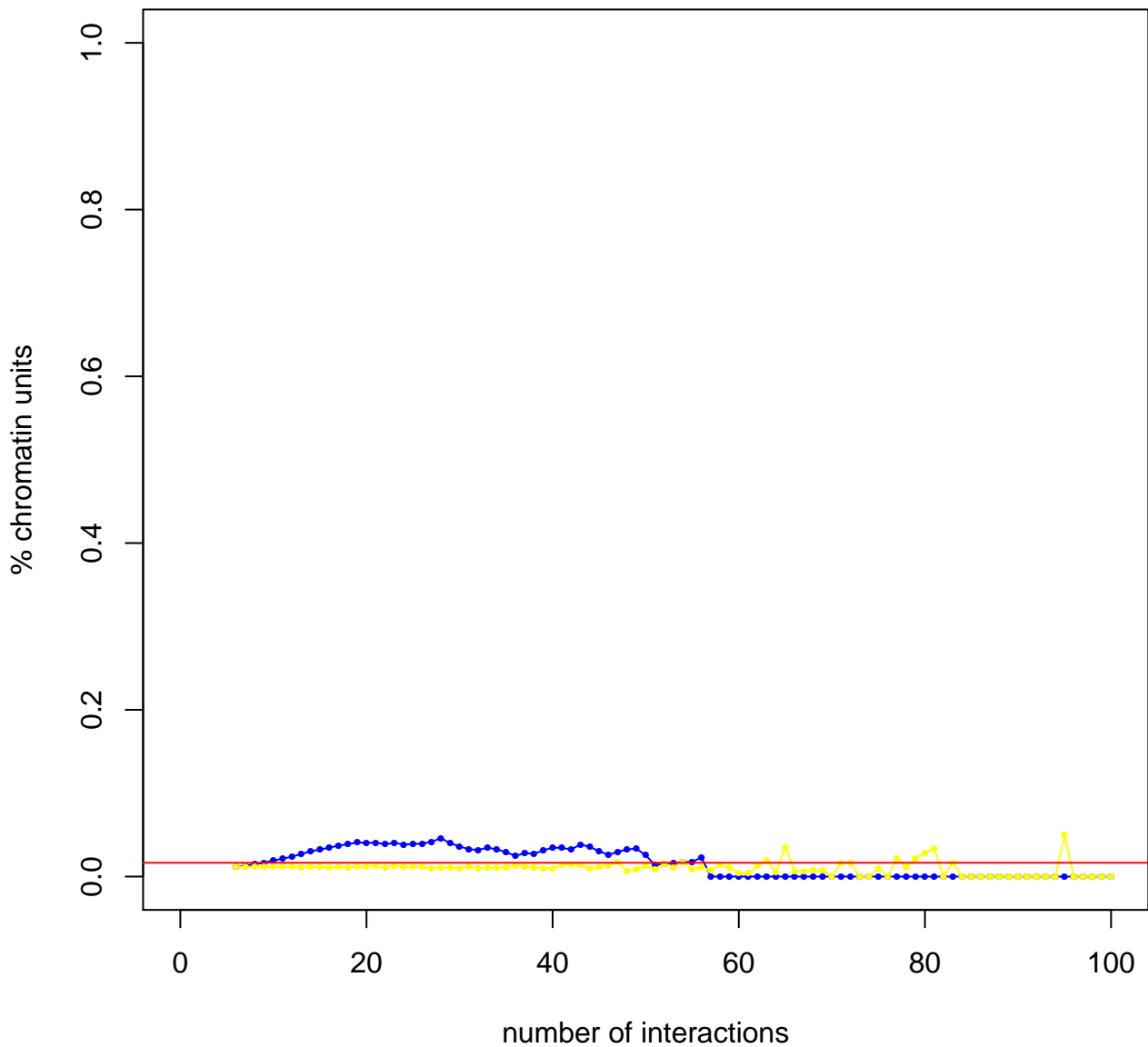

Supplement: Supplementary file 3 — A folder named SB-06-S3 contains 105 overlapping plot for each TF. (ZIP 624 kb) [file 12918_2018_643_MOESM3_ESM.zip › SB-06-S3/AZF1.pdf]

# BAS1

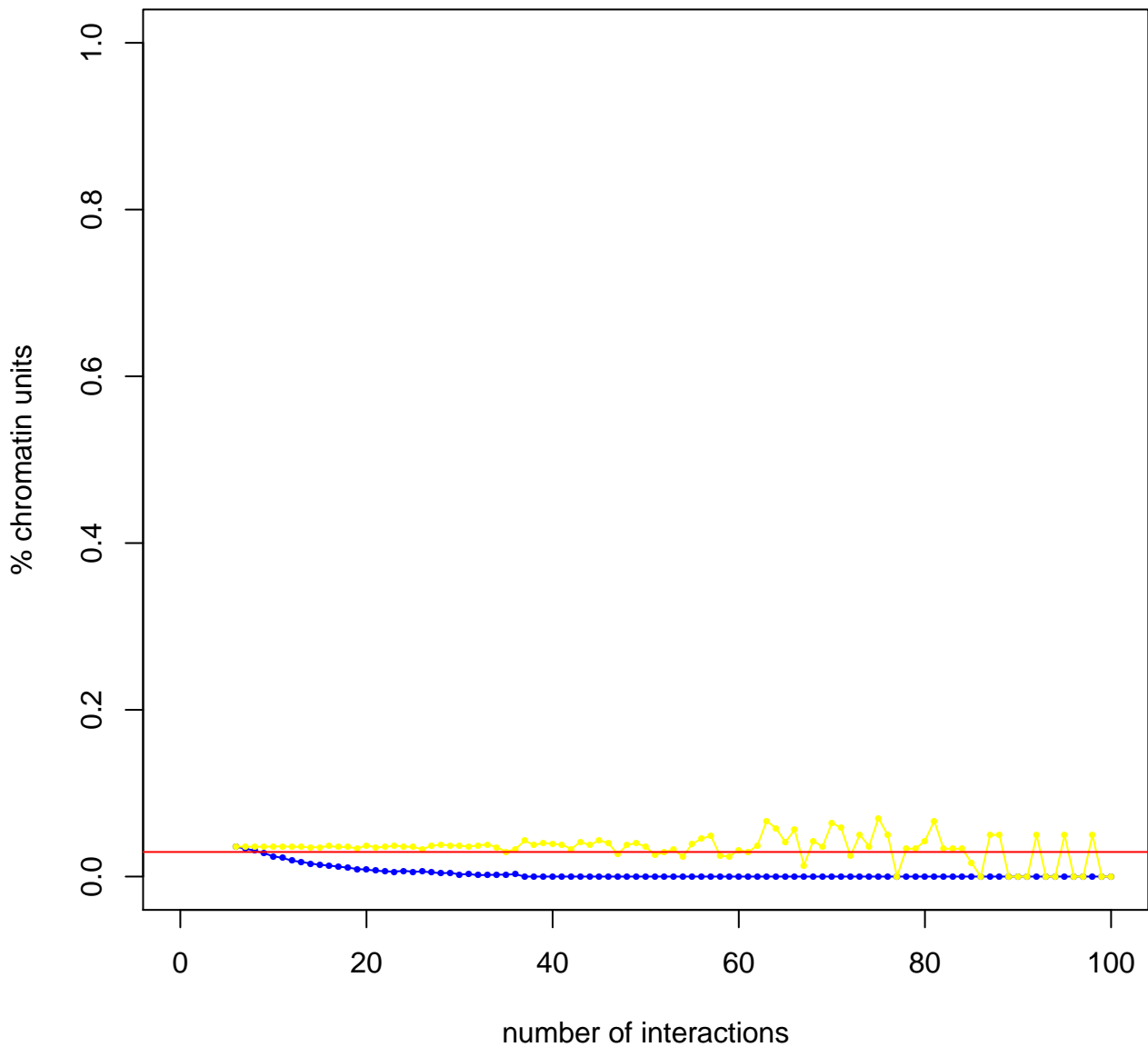

Supplement: Supplementary file 3 — A folder named SB-06-S3 contains 105 overlapping plot for each TF. (ZIP 624 kb) [file 12918_2018_643_MOESM3_ESM.zip › SB-06-S3/BAS1.pdf]

# CAD1

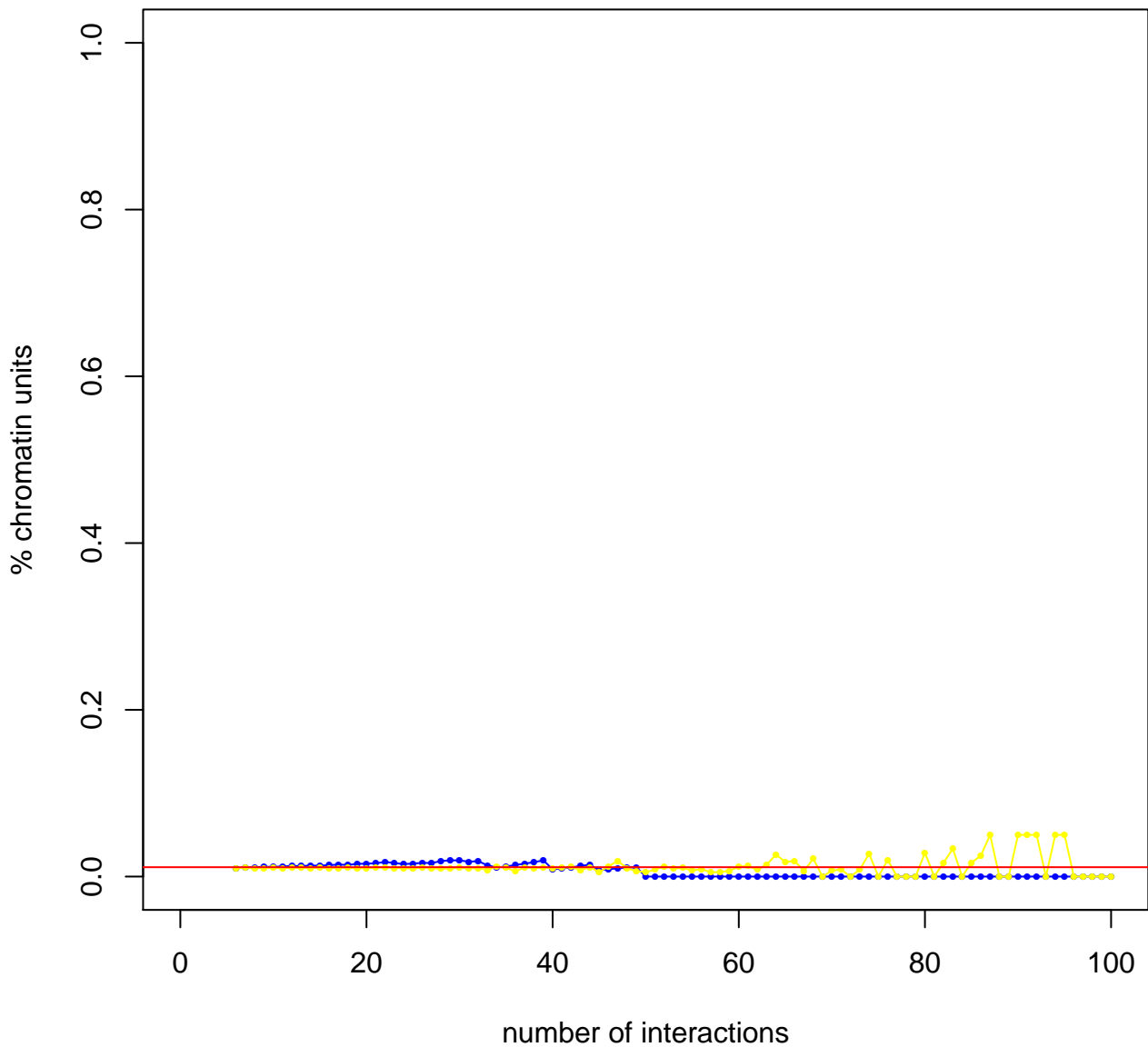

Supplement: Supplementary file 3 — A folder named SB-06-S3 contains 105 overlapping plot for each TF. (ZIP 624 kb) [file 12918_2018_643_MOESM3_ESM.zip › SB-06-S3/CAD1.pdf]

# CBF1

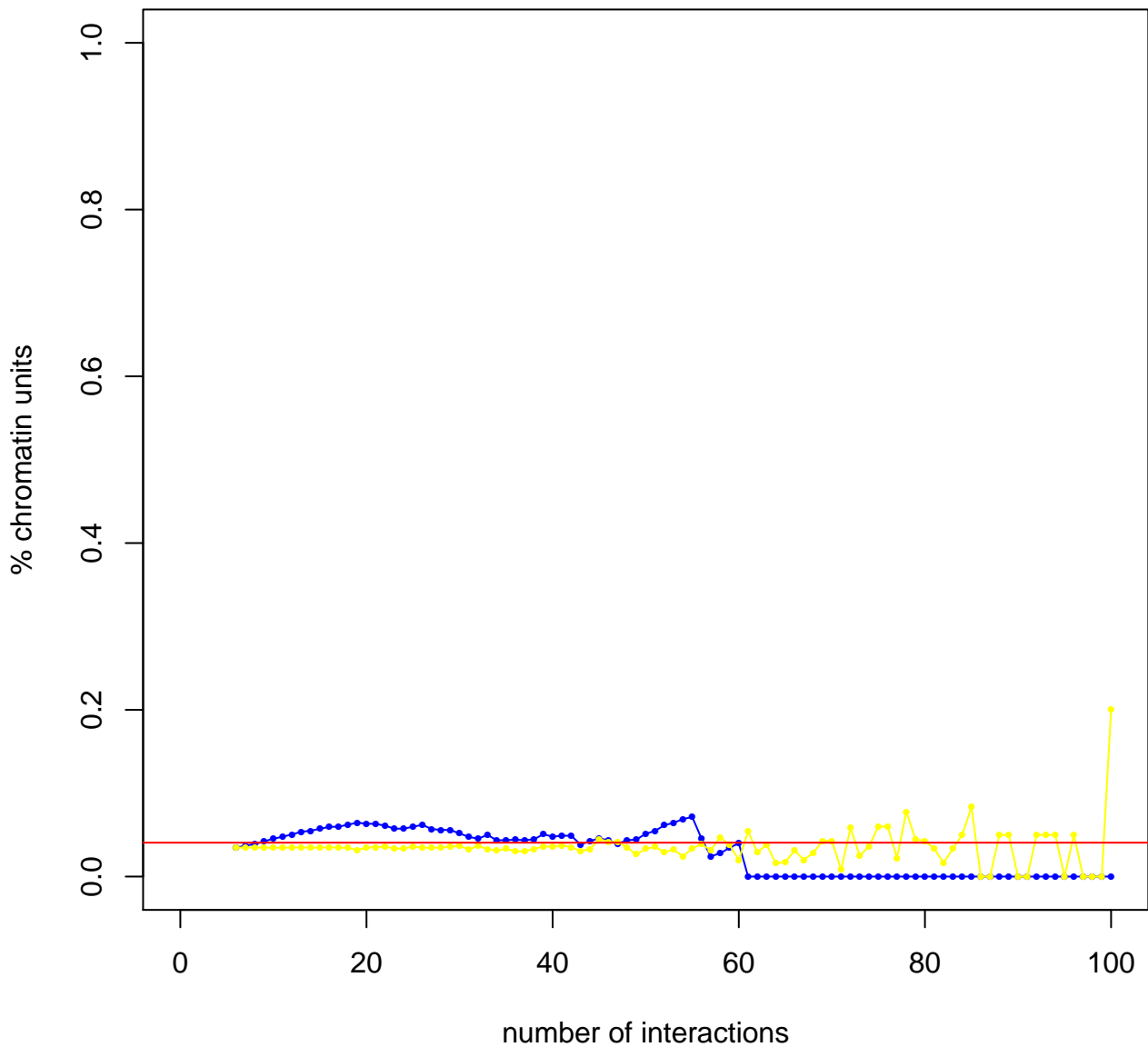

Supplement: Supplementary file 3 — A folder named SB-06-S3 contains 105 overlapping plot for each TF. (ZIP 624 kb) [file 12918_2018_643_MOESM3_ESM.zip › SB-06-S3/CBF1.pdf]

# CIN5

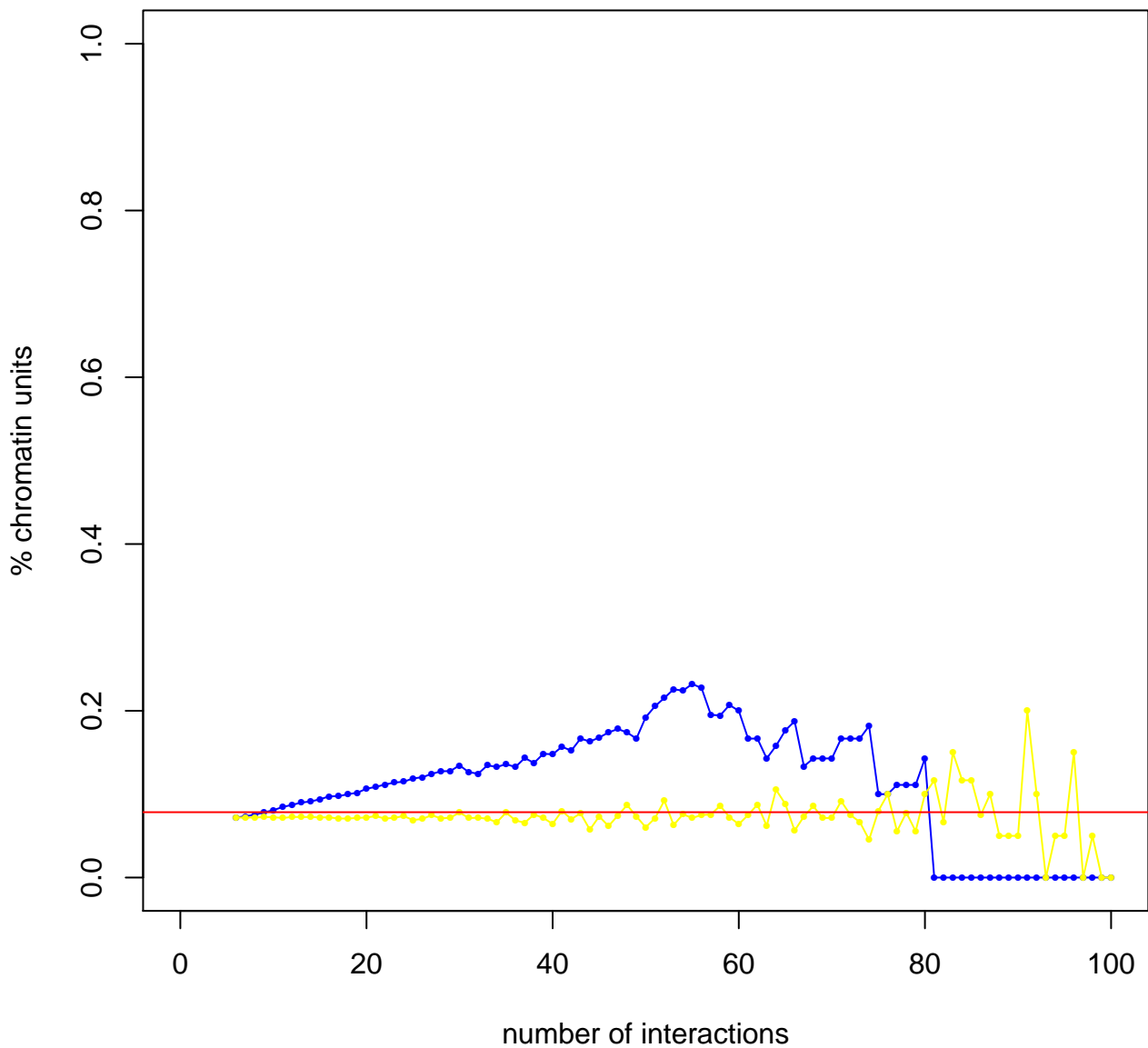

Supplement: Supplementary file 3 — A folder named SB-06-S3 contains 105 overlapping plot for each TF. (ZIP 624 kb) [file 12918_2018_643_MOESM3_ESM.zip › SB-06-S3/CIN5.pdf]

# CRZ1

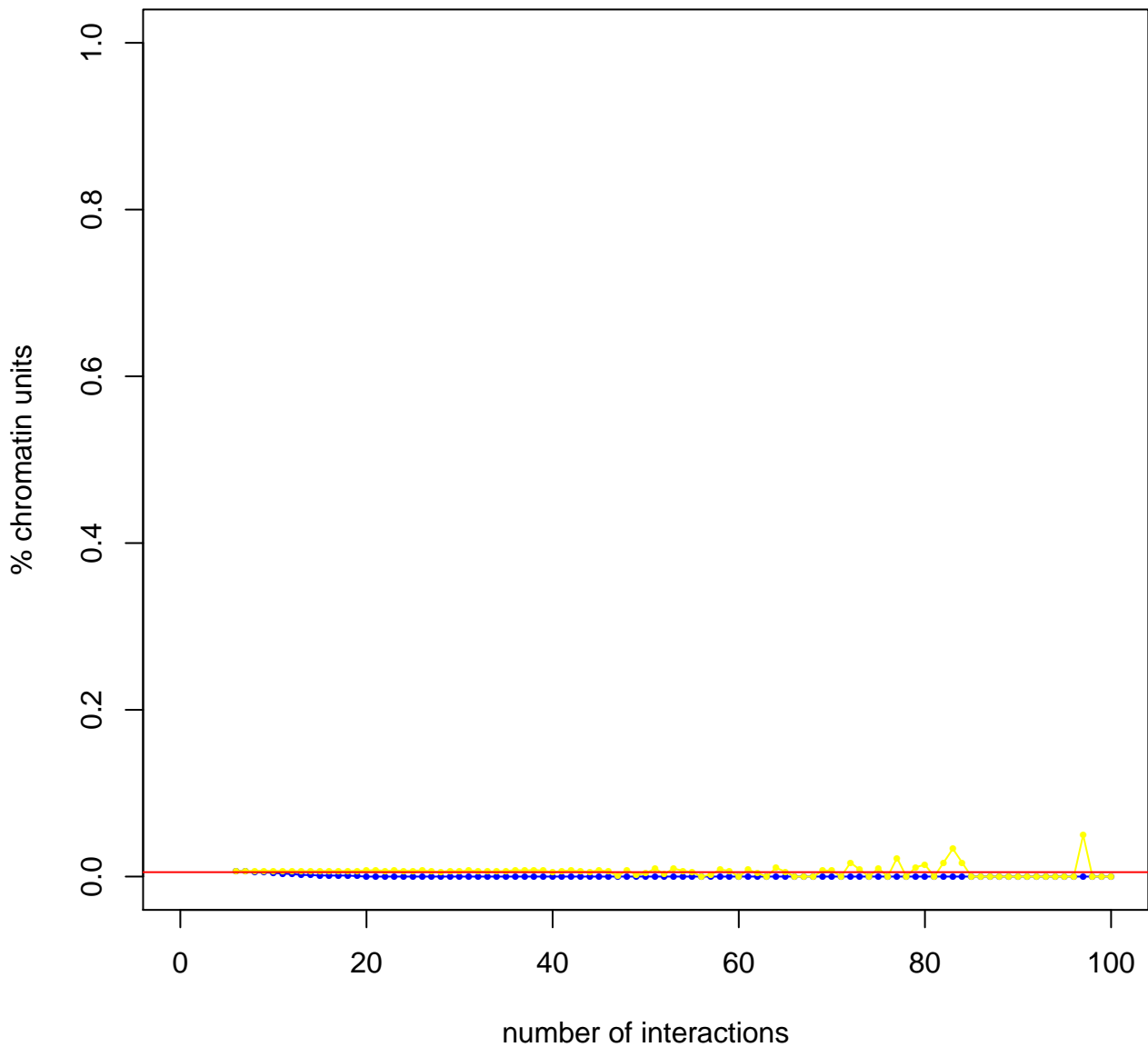

Supplement: Supplementary file 3 — A folder named SB-06-S3 contains 105 overlapping plot for each TF. (ZIP 624 kb) [file 12918_2018_643_MOESM3_ESM.zip › SB-06-S3/CRZ1.pdf]

# DAL80

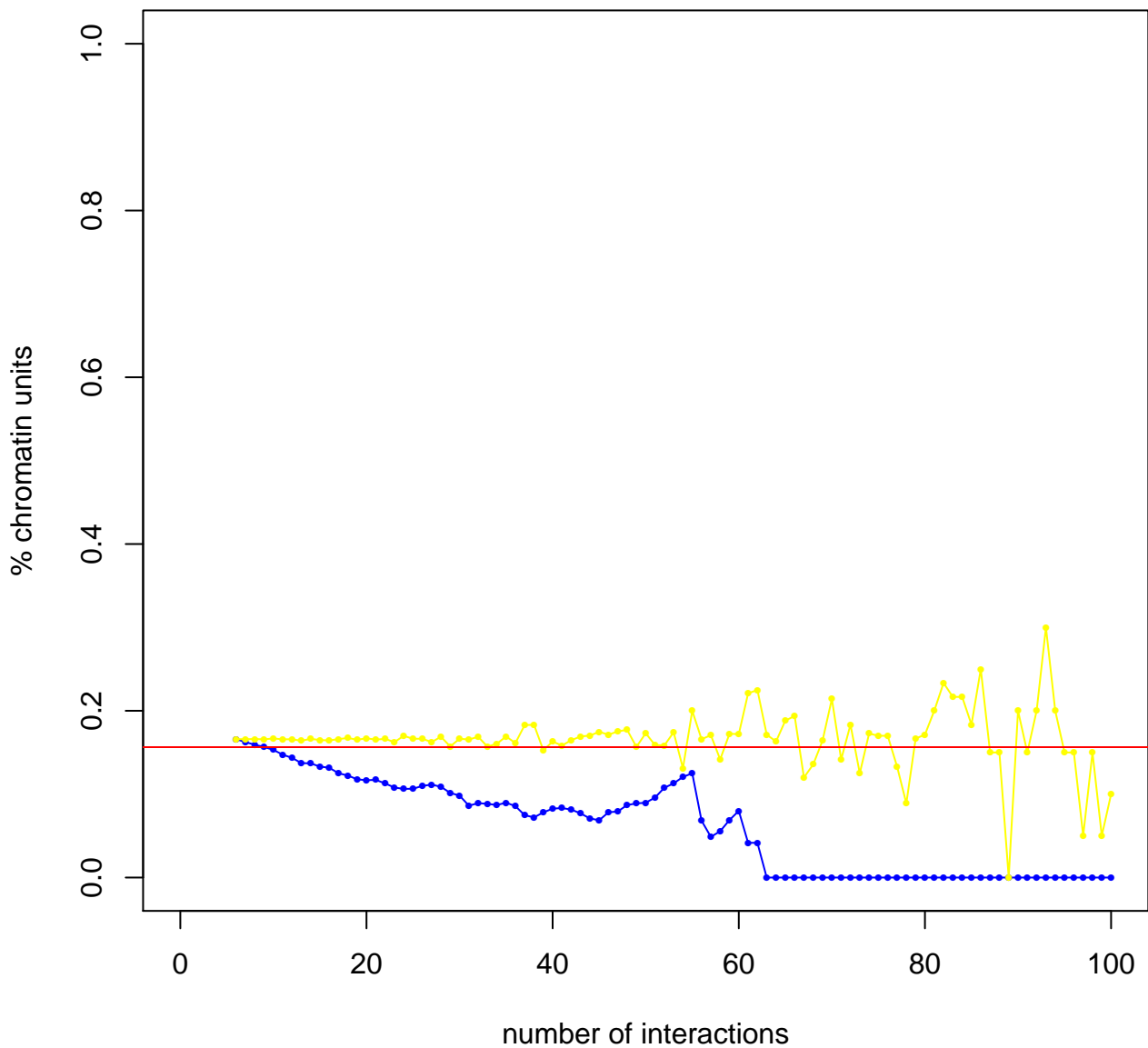

Supplement: Supplementary file 3 — A folder named SB-06-S3 contains 105 overlapping plot for each TF. (ZIP 624 kb) [file 12918_2018_643_MOESM3_ESM.zip › SB-06-S3/DAL80.pdf]

# DAL81

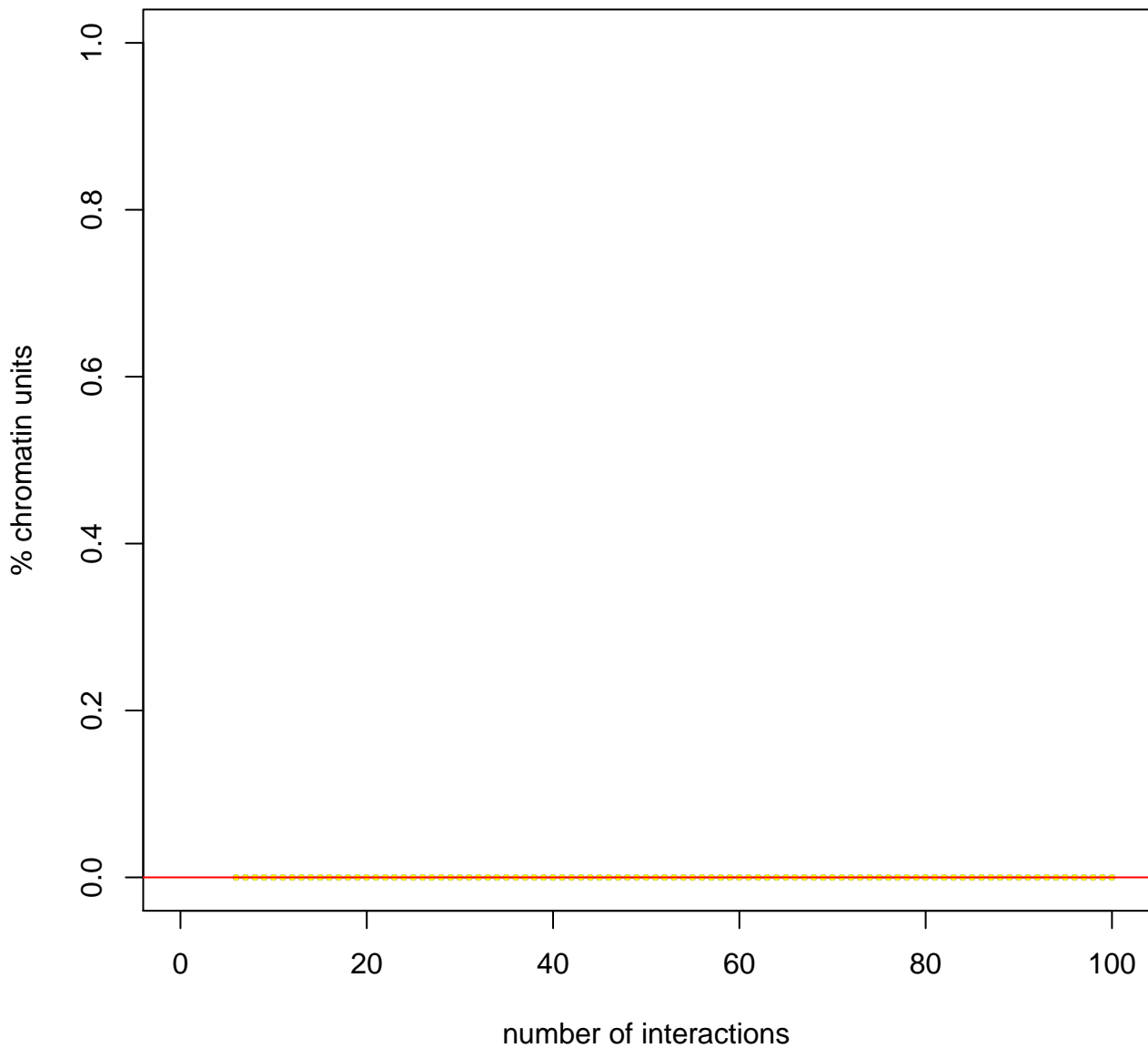

Supplement: Supplementary file 3 — A folder named SB-06-S3 contains 105 overlapping plot for each TF. (ZIP 624 kb) [file 12918_2018_643_MOESM3_ESM.zip › SB-06-S3/DAL81.pdf]

# DAL82

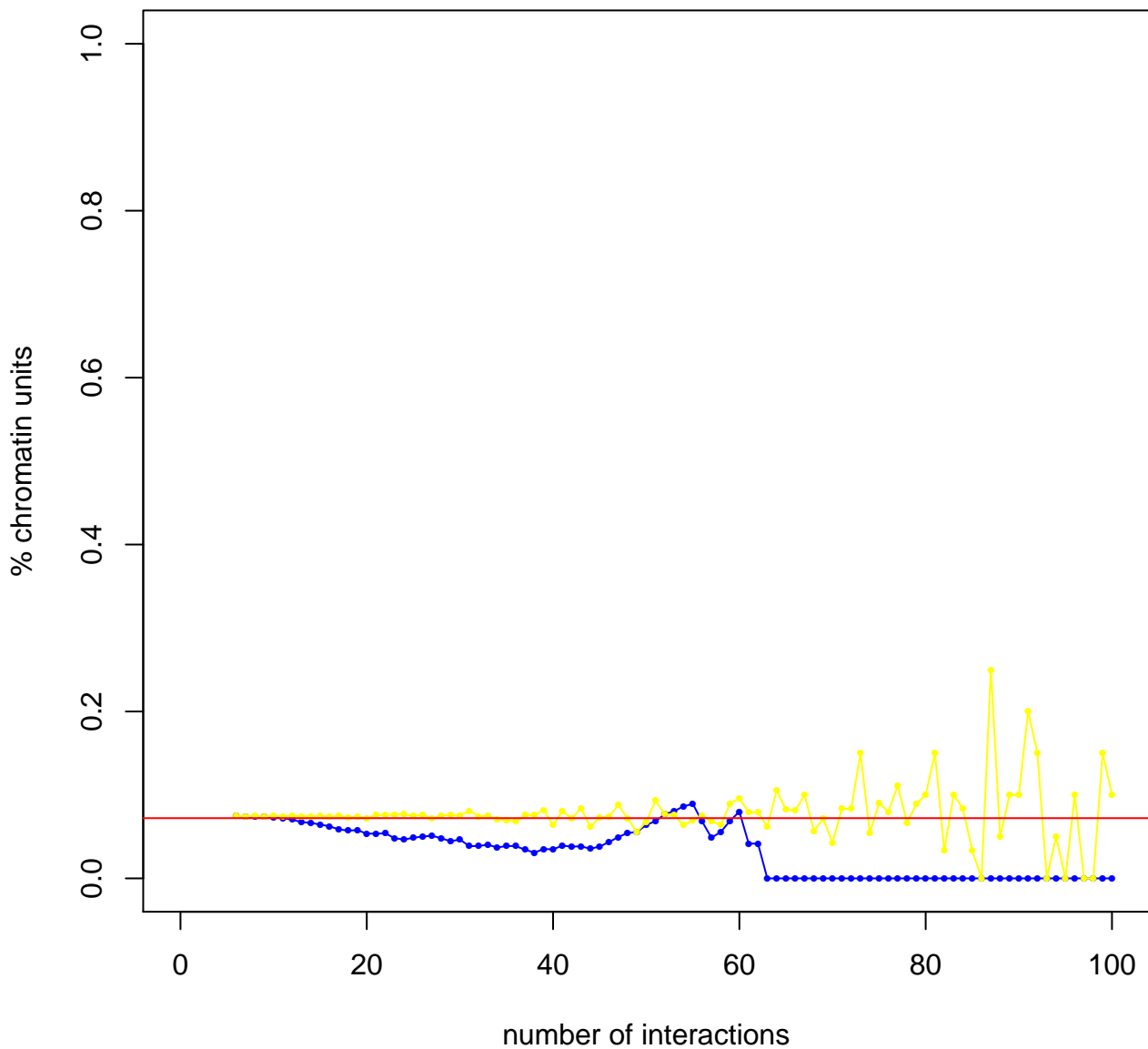

Supplement: Supplementary file 3 — A folder named SB-06-S3 contains 105 overlapping plot for each TF. (ZIP 624 kb) [file 12918_2018_643_MOESM3_ESM.zip › SB-06-S3/DAL82.pdf]

# DIG1

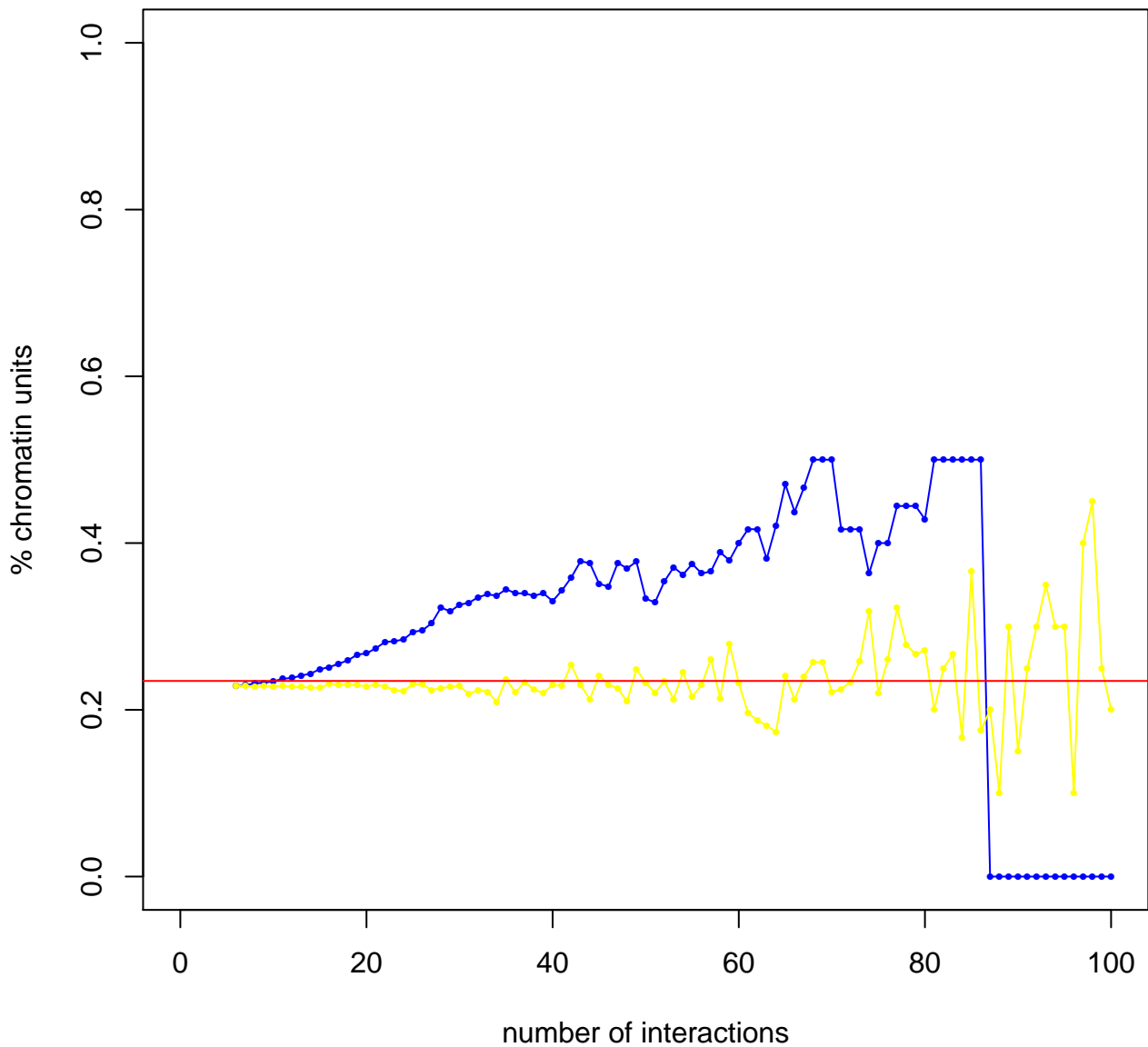

Supplement: Supplementary file 3 — A folder named SB-06-S3 contains 105 overlapping plot for each TF. (ZIP 624 kb) [file 12918_2018_643_MOESM3_ESM.zip › SB-06-S3/DIG1.pdf]

## ECM22

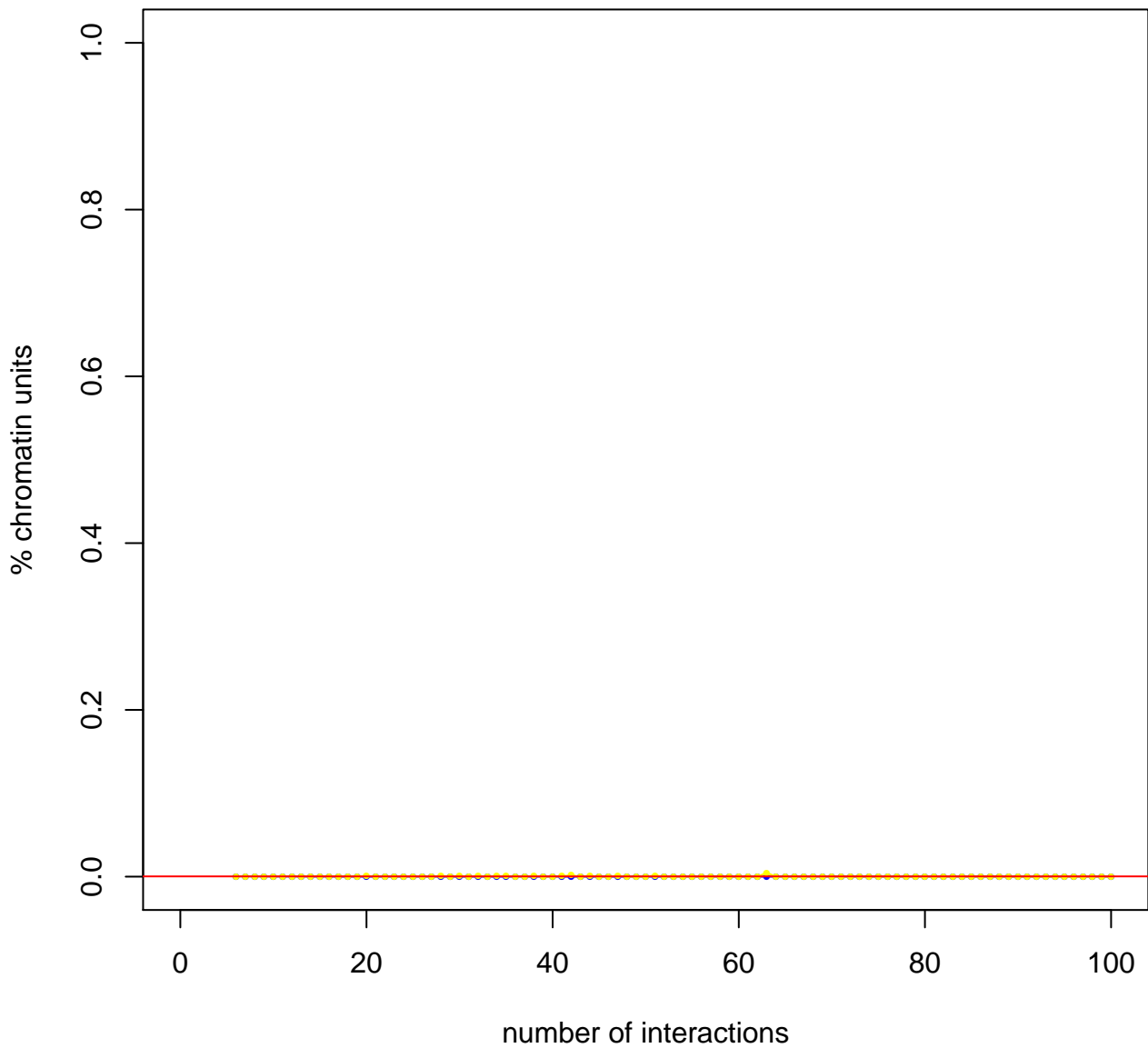

Supplement: Supplementary file 3 — A folder named SB-06-S3 contains 105 overlapping plot for each TF. (ZIP 624 kb) [file 12918_2018_643_MOESM3_ESM.zip › SB-06-S3/ECM22.pdf]

# FHL1

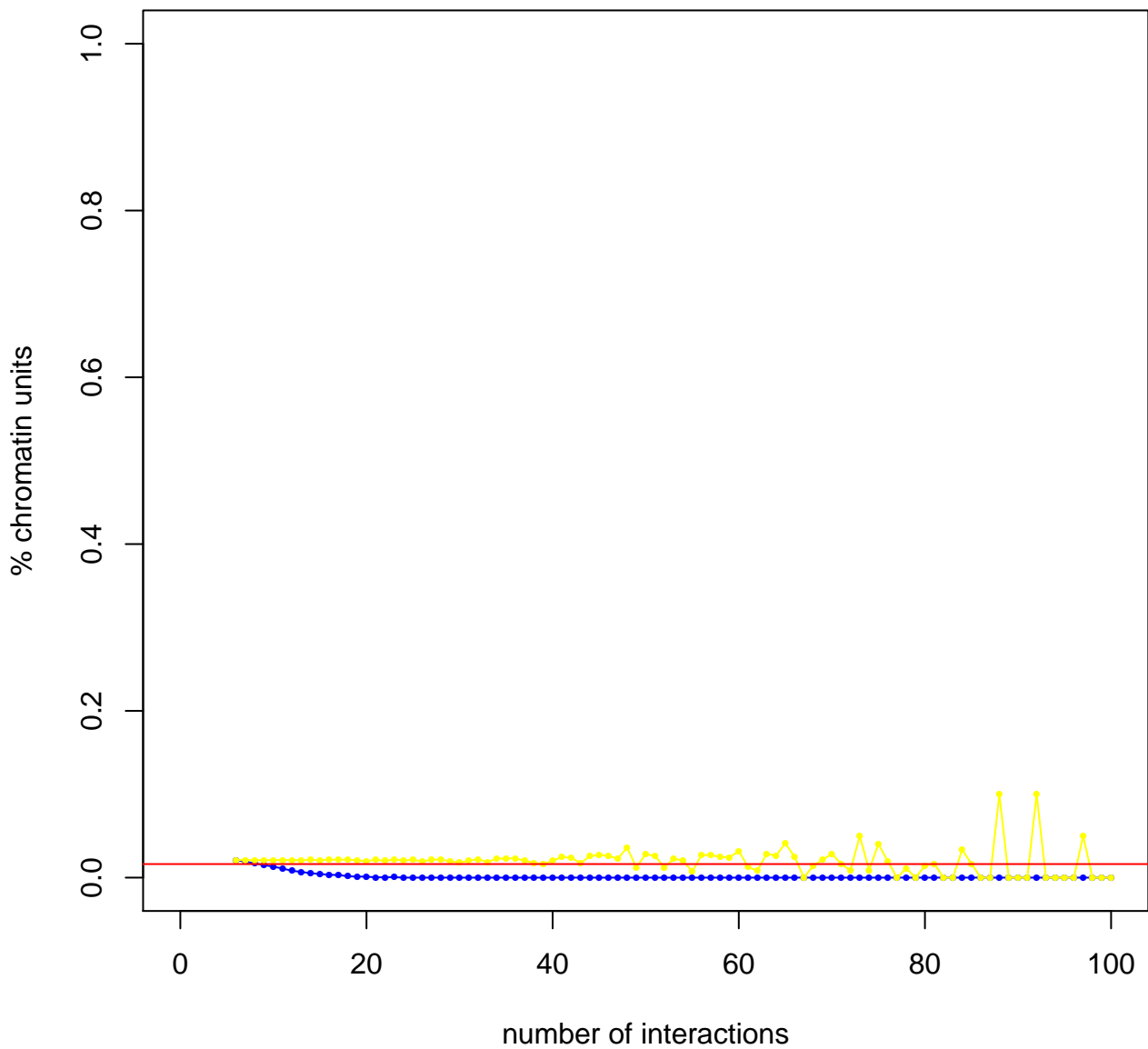

Supplement: Supplementary file 3 — A folder named SB-06-S3 contains 105 overlapping plot for each TF. (ZIP 624 kb) [file 12918_2018_643_MOESM3_ESM.zip › SB-06-S3/FHL1.pdf]

# FKH1

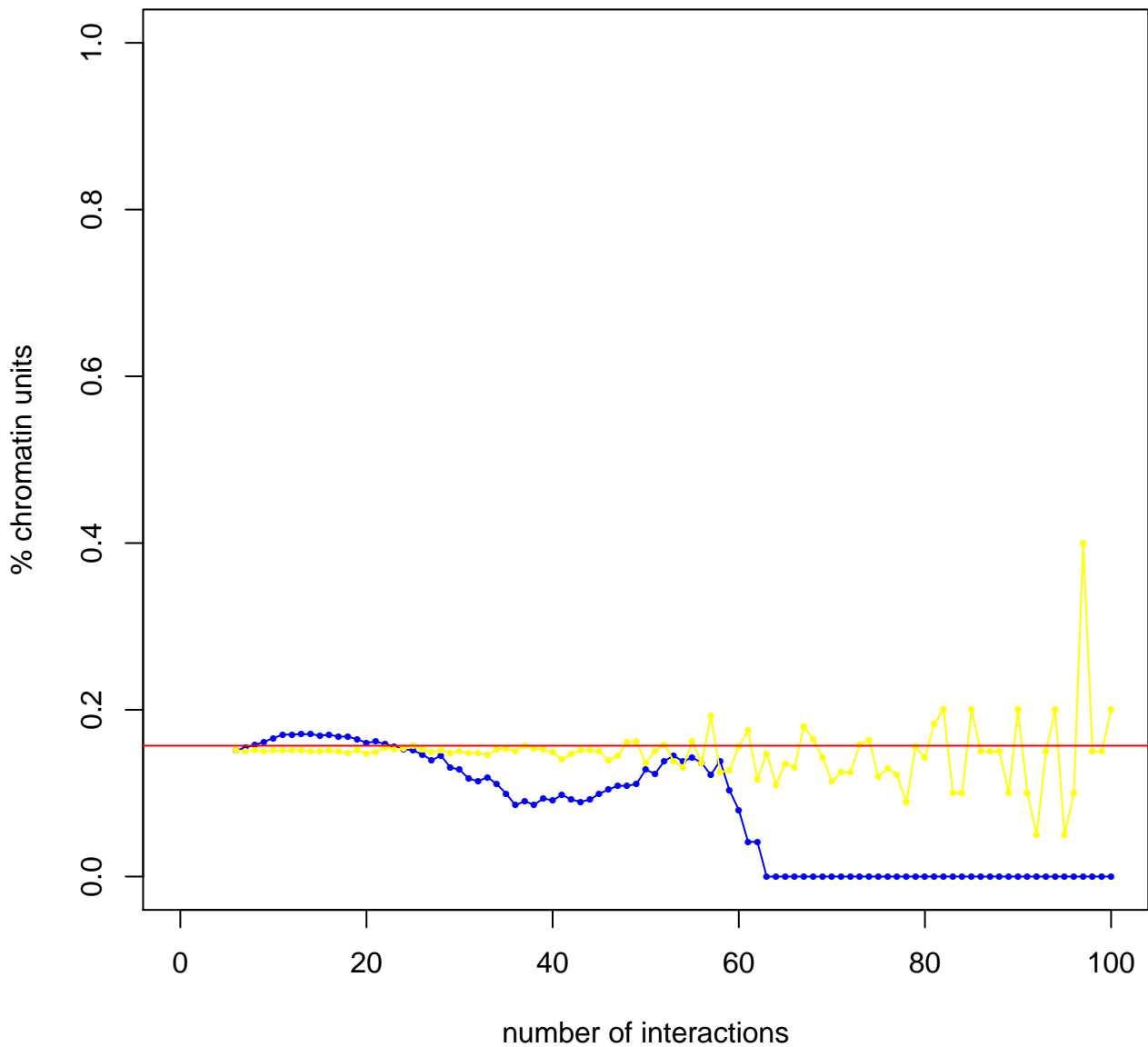

Supplement: Supplementary file 3 — A folder named SB-06-S3 contains 105 overlapping plot for each TF. (ZIP 624 kb) [file 12918_2018_643_MOESM3_ESM.zip › SB-06-S3/FKH1.pdf]

# FKH2

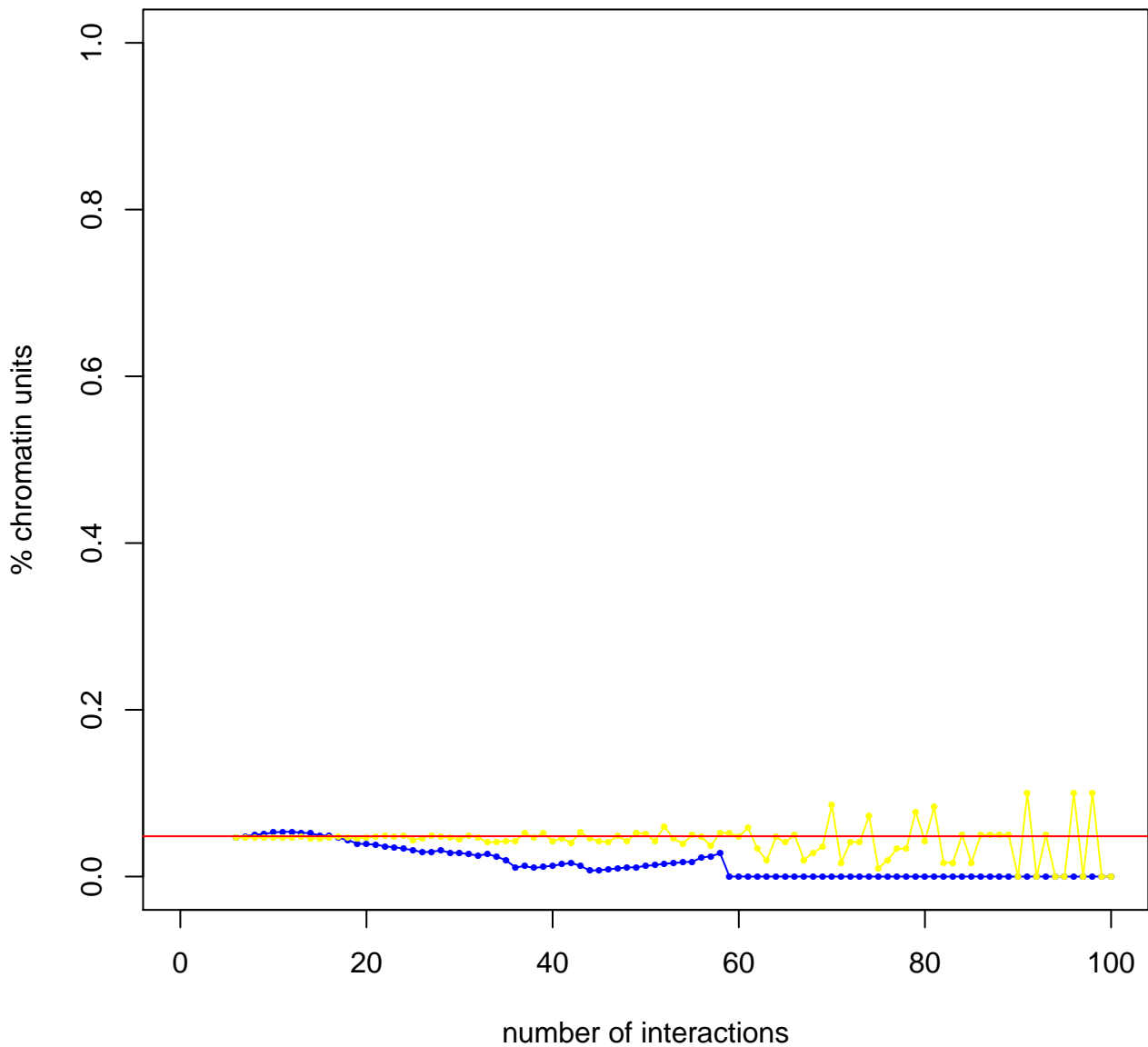

Supplement: Supplementary file 3 — A folder named SB-06-S3 contains 105 overlapping plot for each TF. (ZIP 624 kb) [file 12918_2018_643_MOESM3_ESM.zip › SB-06-S3/FKH2.pdf]

# GAL4

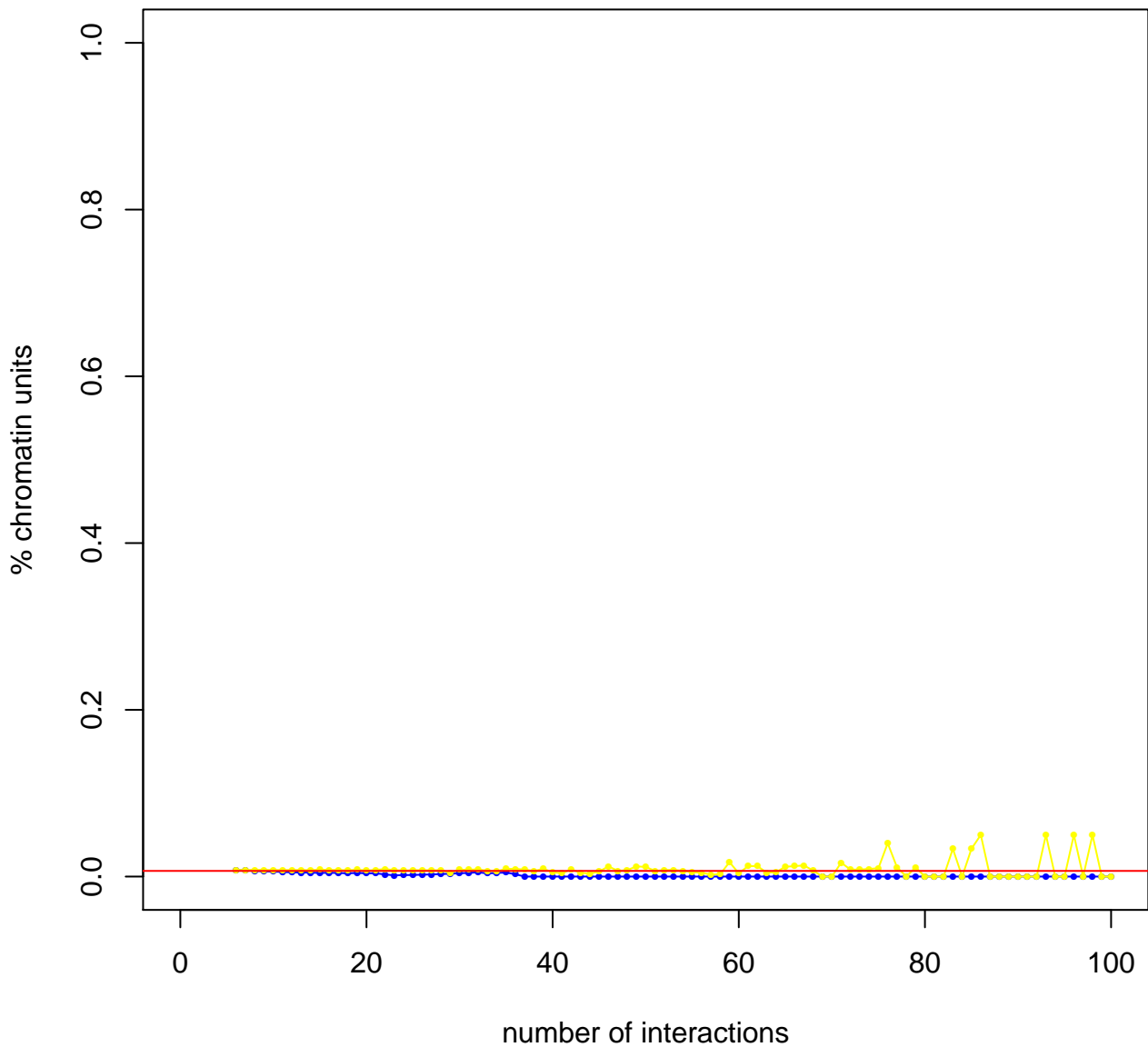

Supplement: Supplementary file 3 — A folder named SB-06-S3 contains 105 overlapping plot for each TF. (ZIP 624 kb) [file 12918_2018_643_MOESM3_ESM.zip › SB-06-S3/GAL4.pdf]

# GAL80

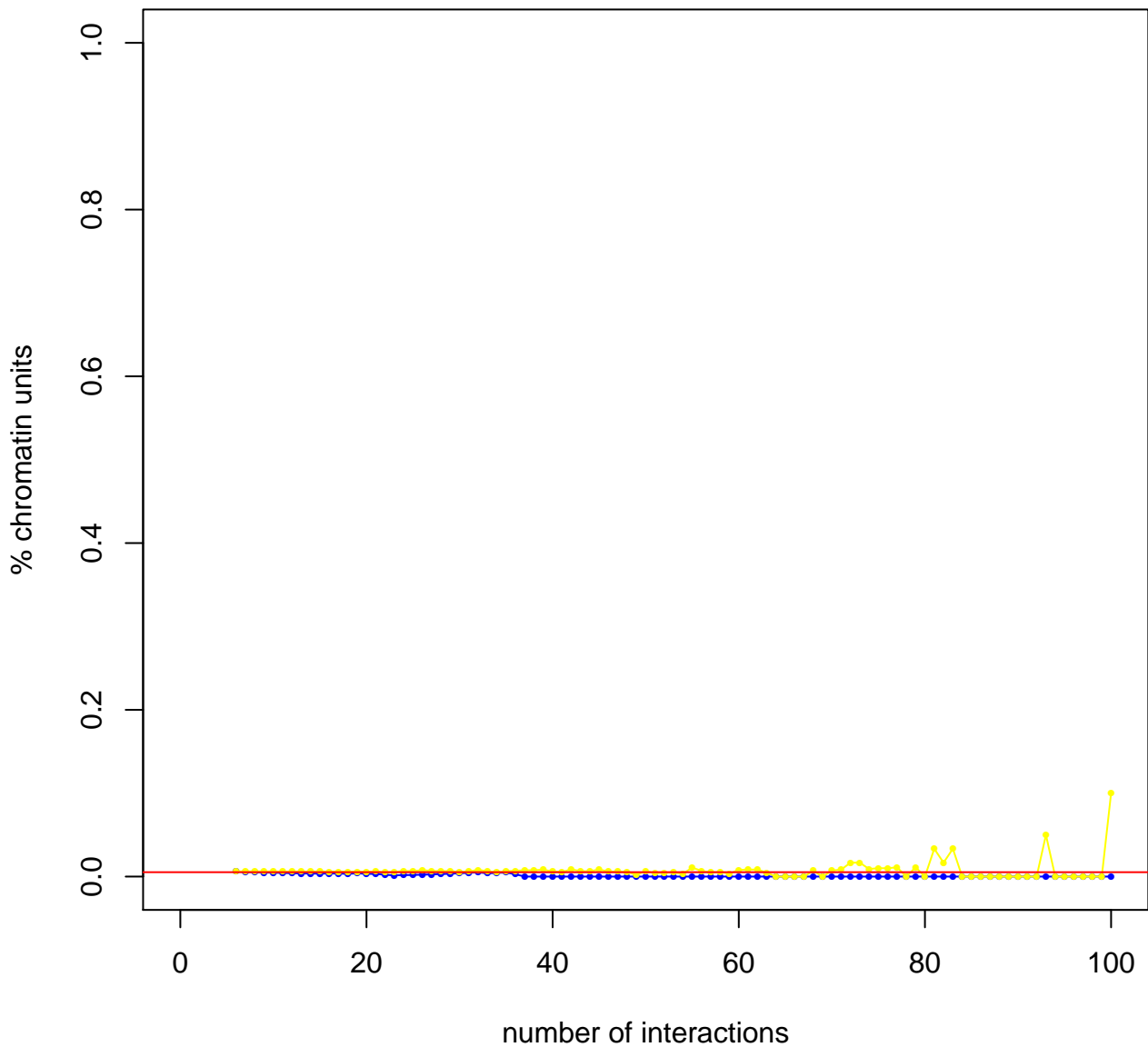

Supplement: Supplementary file 3 — A folder named SB-06-S3 contains 105 overlapping plot for each TF. (ZIP 624 kb) [file 12918_2018_643_MOESM3_ESM.zip › SB-06-S3/GAL80.pdf]

# GAT1

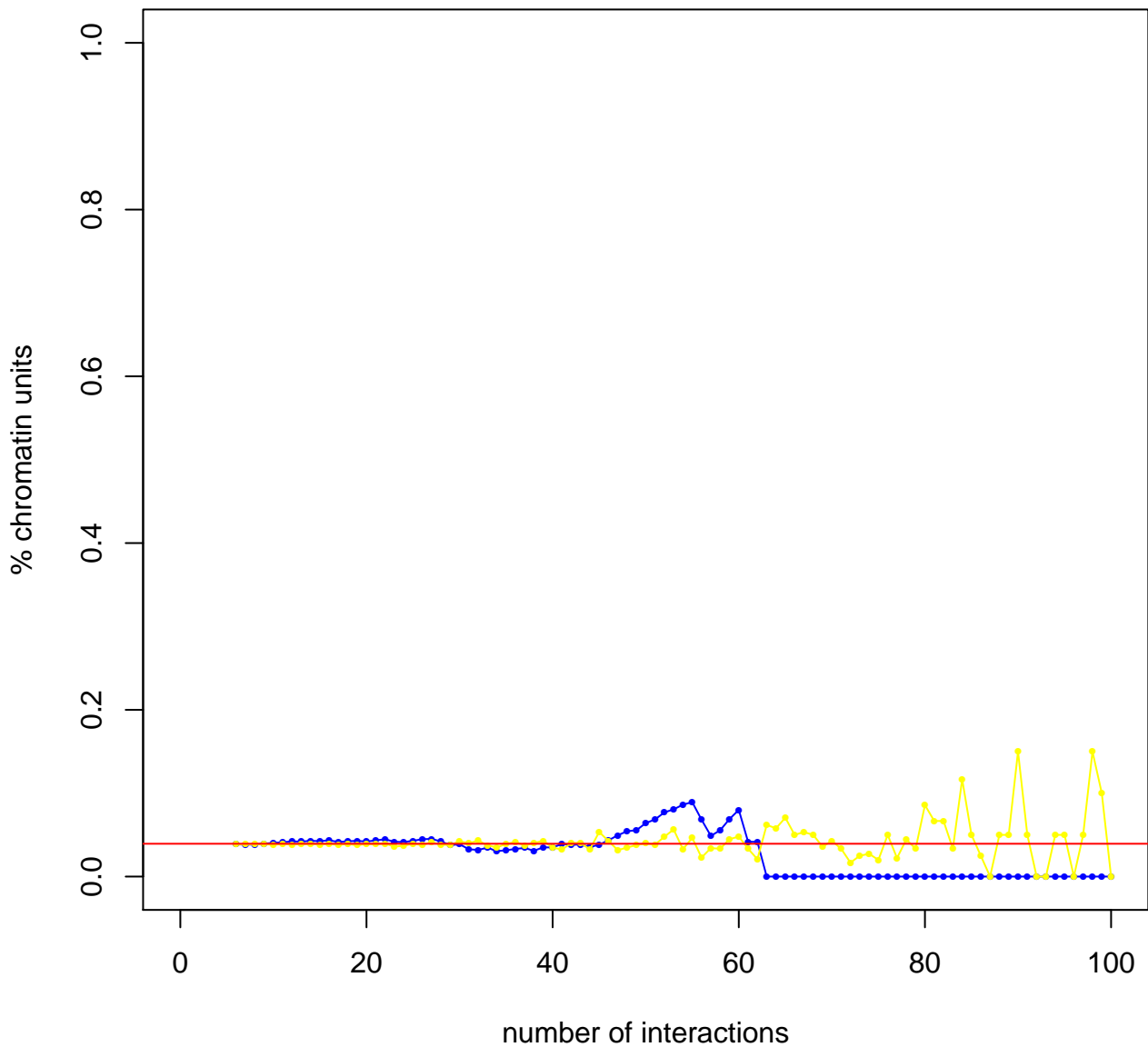

Supplement: Supplementary file 3 — A folder named SB-06-S3 contains 105 overlapping plot for each TF. (ZIP 624 kb) [file 12918_2018_643_MOESM3_ESM.zip › SB-06-S3/GAT1.pdf]

# GCN4

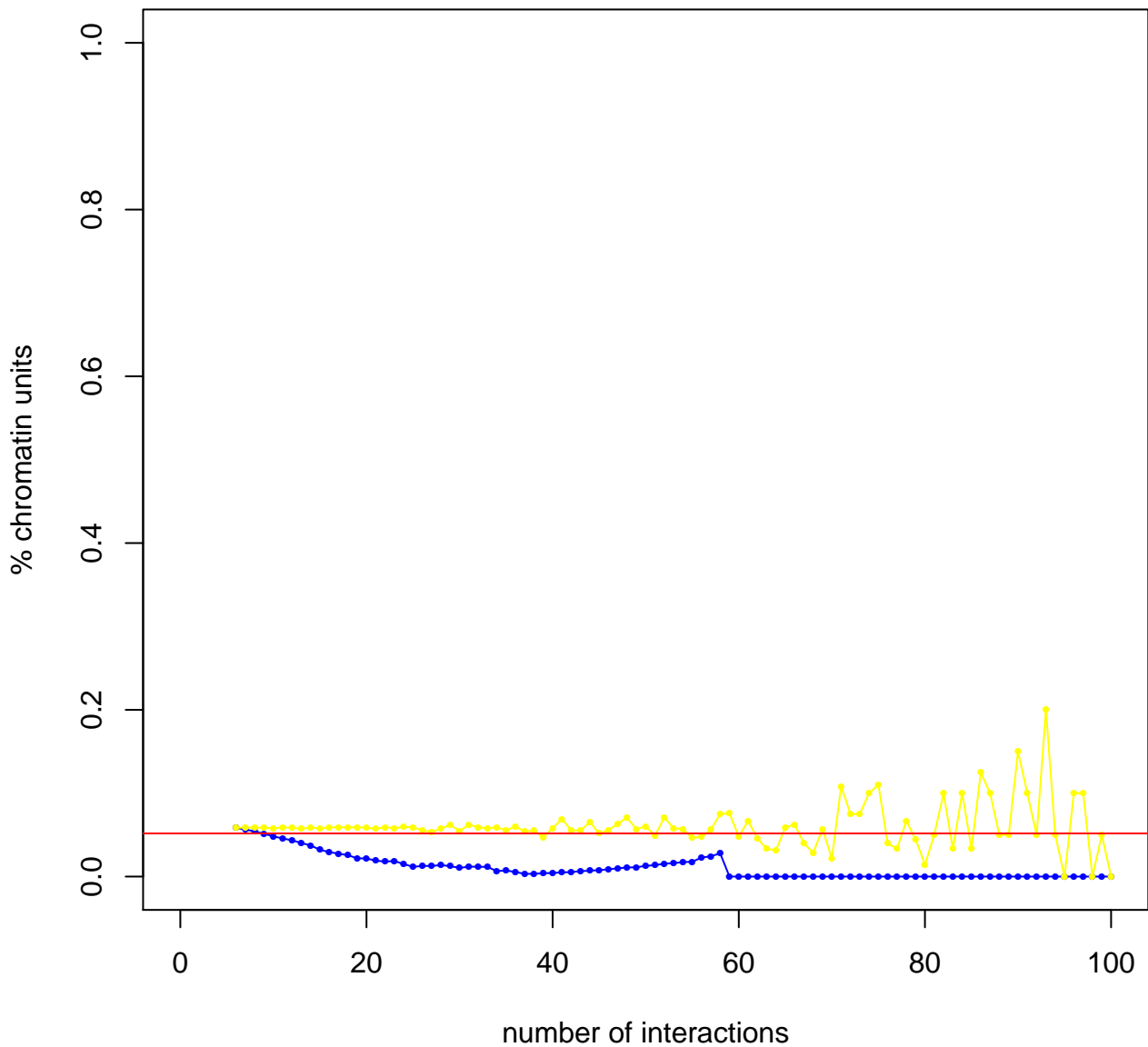

Supplement: Supplementary file 3 — A folder named SB-06-S3 contains 105 overlapping plot for each TF. (ZIP 624 kb) [file 12918_2018_643_MOESM3_ESM.zip › SB-06-S3/GCN4.pdf]

# GCR1

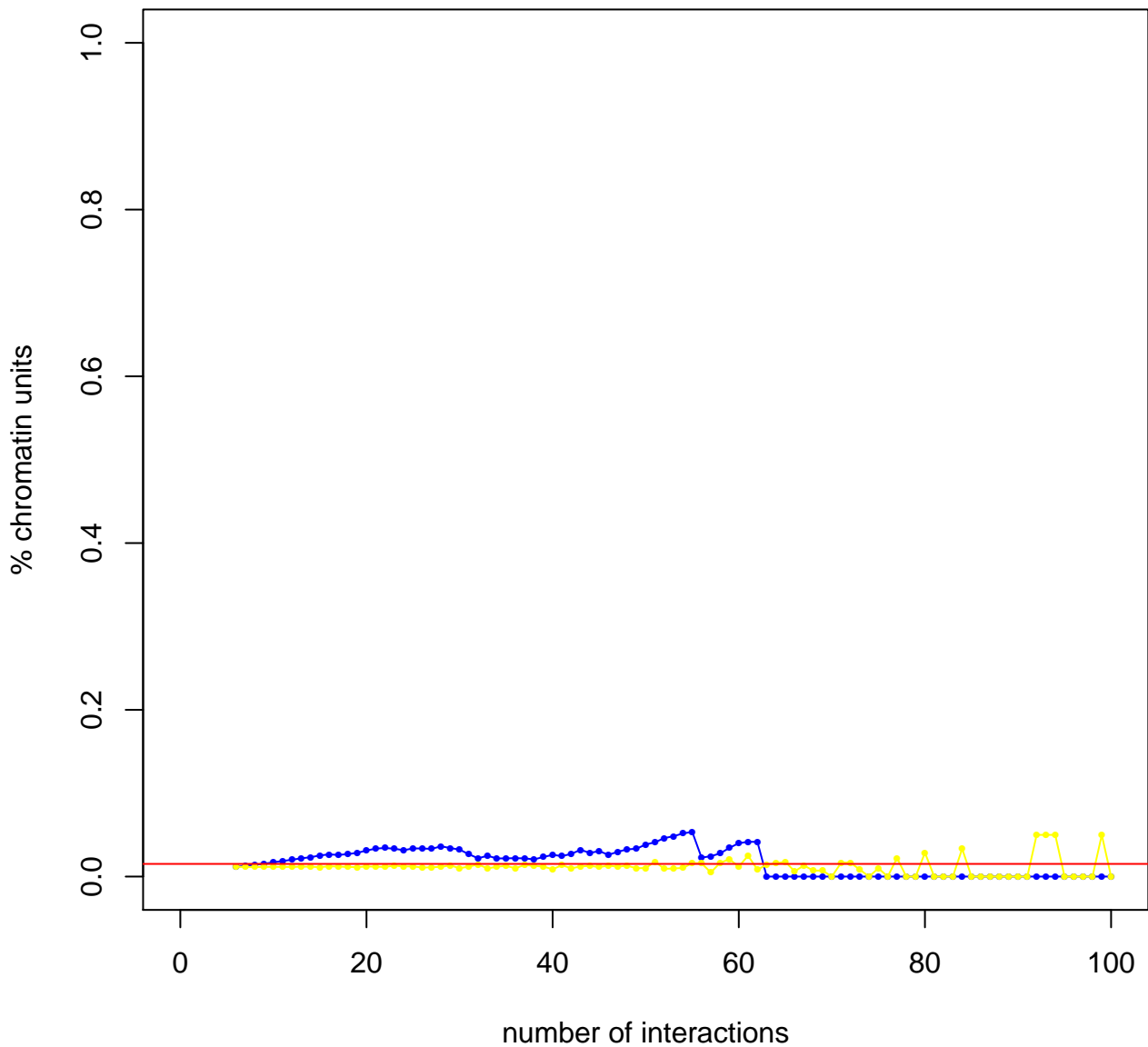

Supplement: Supplementary file 3 — A folder named SB-06-S3 contains 105 overlapping plot for each TF. (ZIP 624 kb) [file 12918_2018_643_MOESM3_ESM.zip › SB-06-S3/GCR1.pdf]

# GLN3

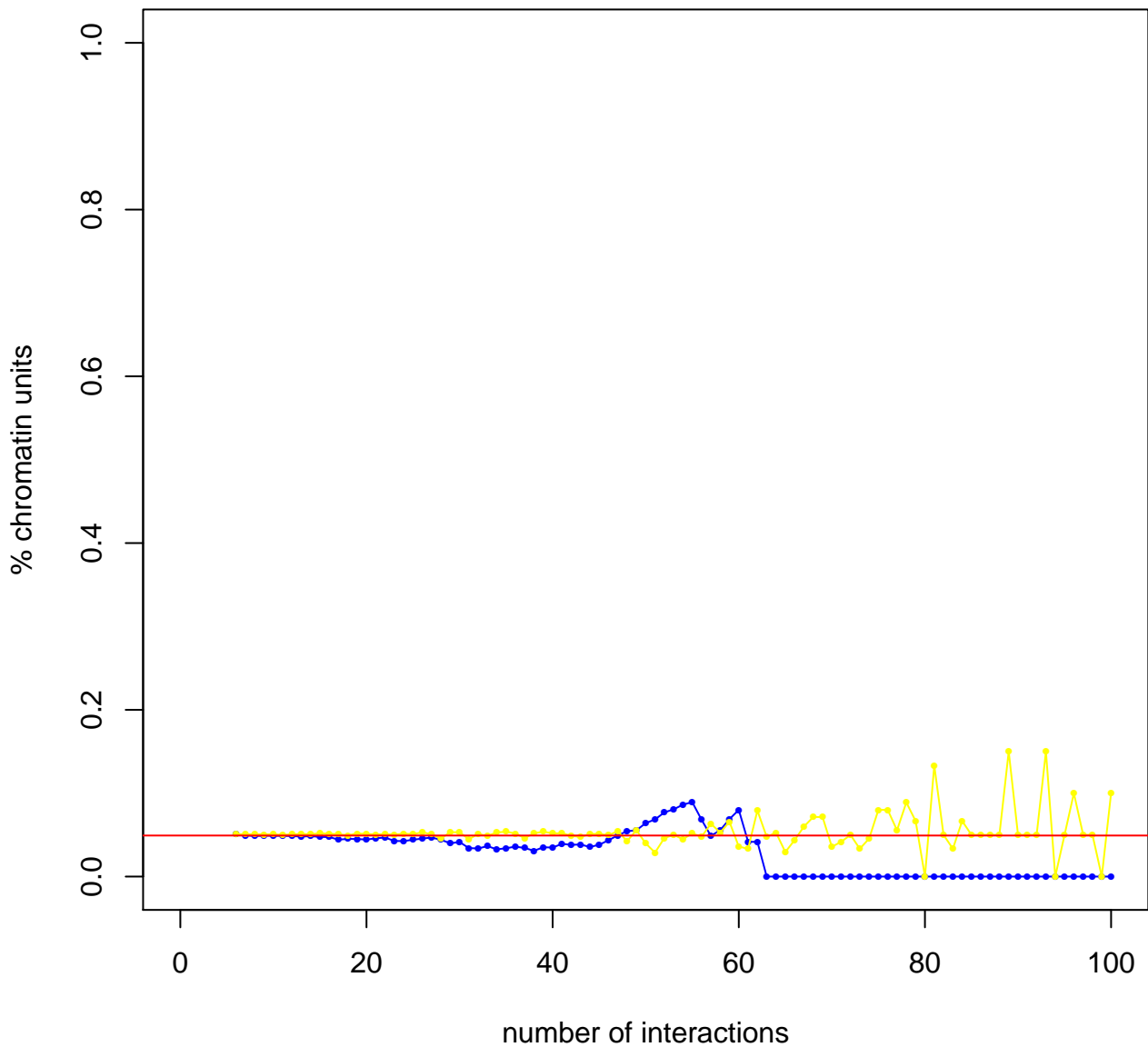

Supplement: Supplementary file 3 — A folder named SB-06-S3 contains 105 overlapping plot for each TF. (ZIP 624 kb) [file 12918_2018_643_MOESM3_ESM.zip › SB-06-S3/GLN3.pdf]

# GZF3

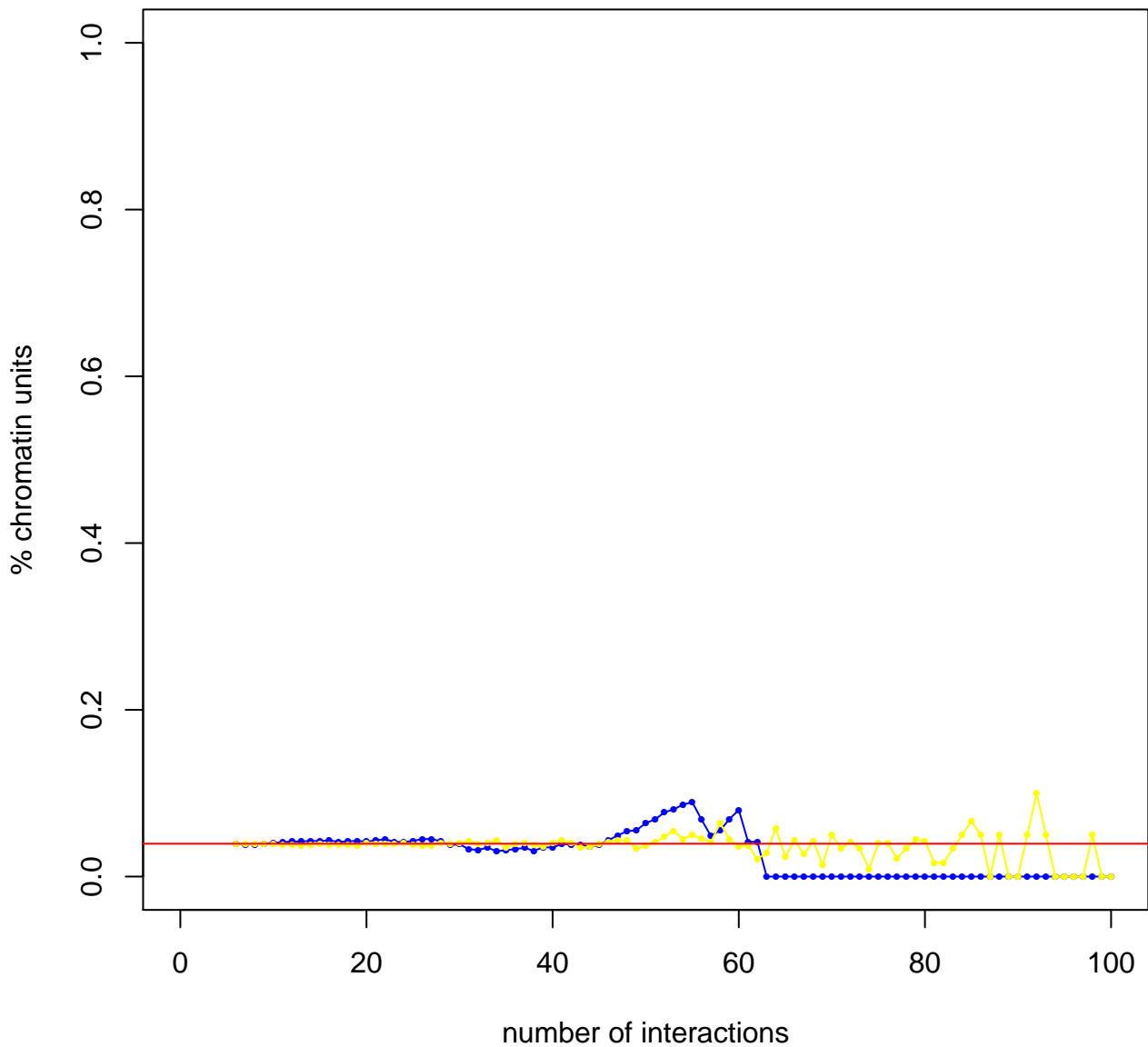

Supplement: Supplementary file 3 — A folder named SB-06-S3 contains 105 overlapping plot for each TF. (ZIP 624 kb) [file 12918_2018_643_MOESM3_ESM.zip › SB-06-S3/GZF3.pdf]

# HAC1

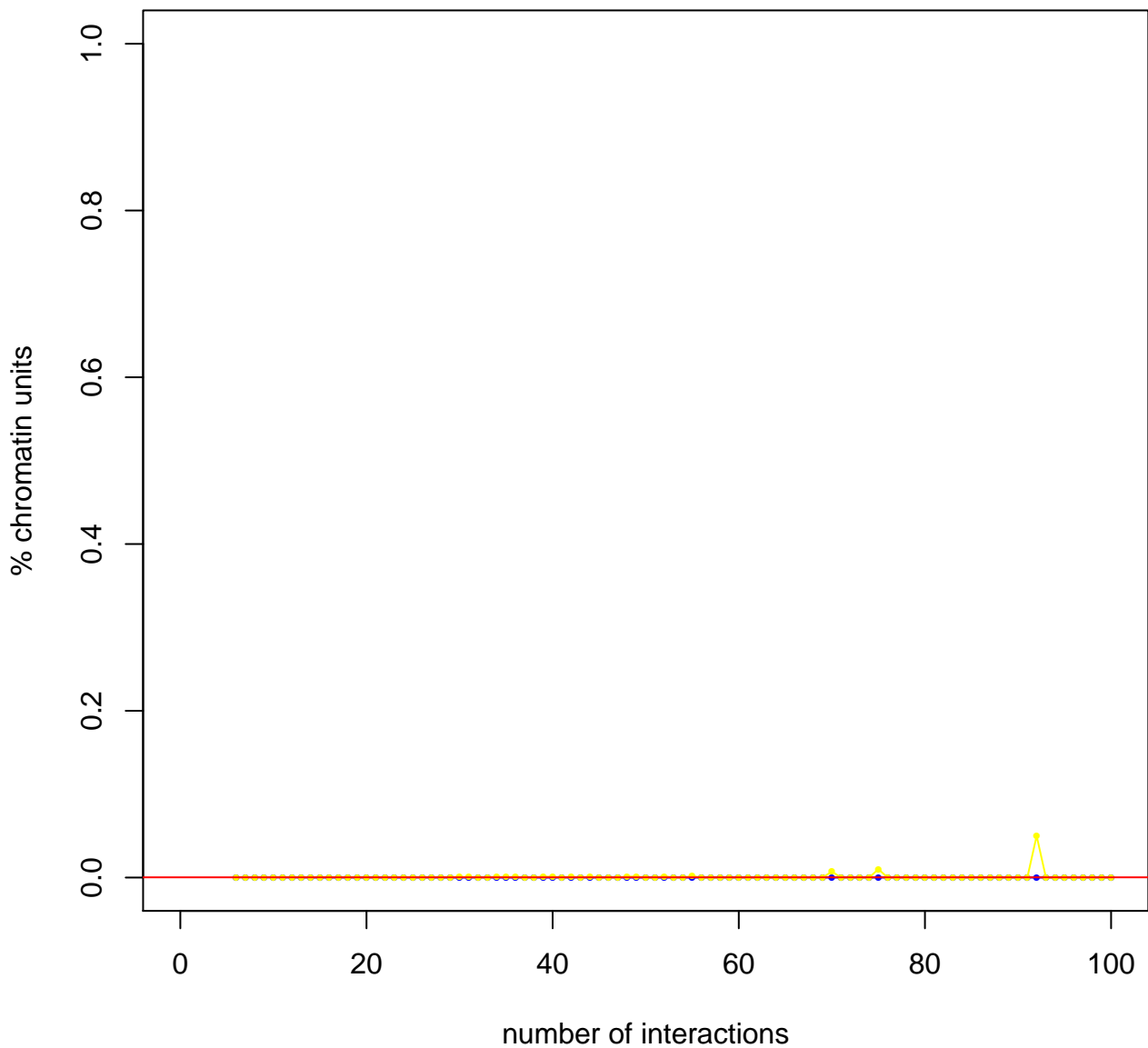

Supplement: Supplementary file 3 — A folder named SB-06-S3 contains 105 overlapping plot for each TF. (ZIP 624 kb) [file 12918_2018_643_MOESM3_ESM.zip › SB-06-S3/HAC1.pdf]

# HAP1

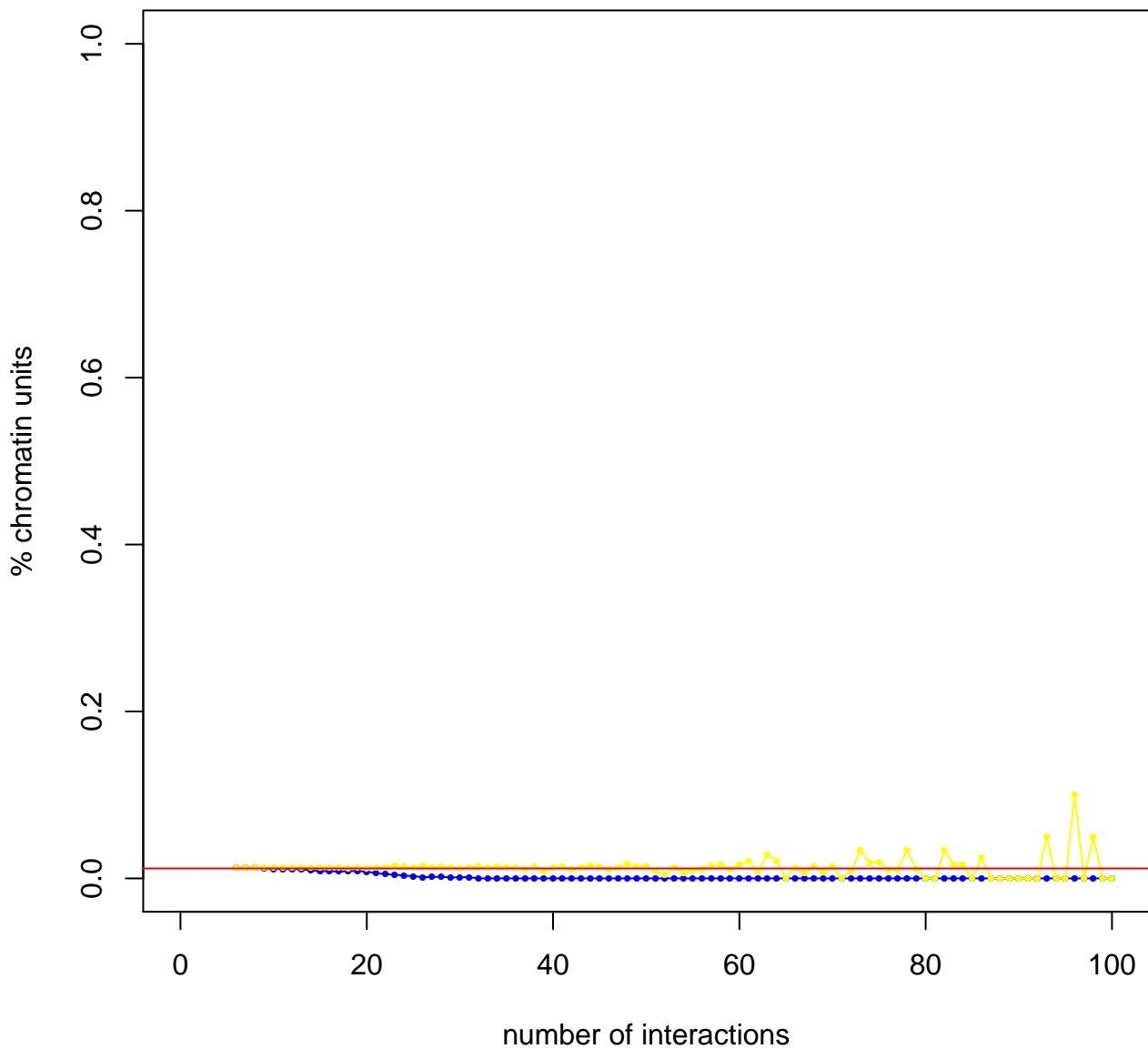

Supplement: Supplementary file 3 — A folder named SB-06-S3 contains 105 overlapping plot for each TF. (ZIP 624 kb) [file 12918_2018_643_MOESM3_ESM.zip › SB-06-S3/HAP1.pdf]

# HAP2

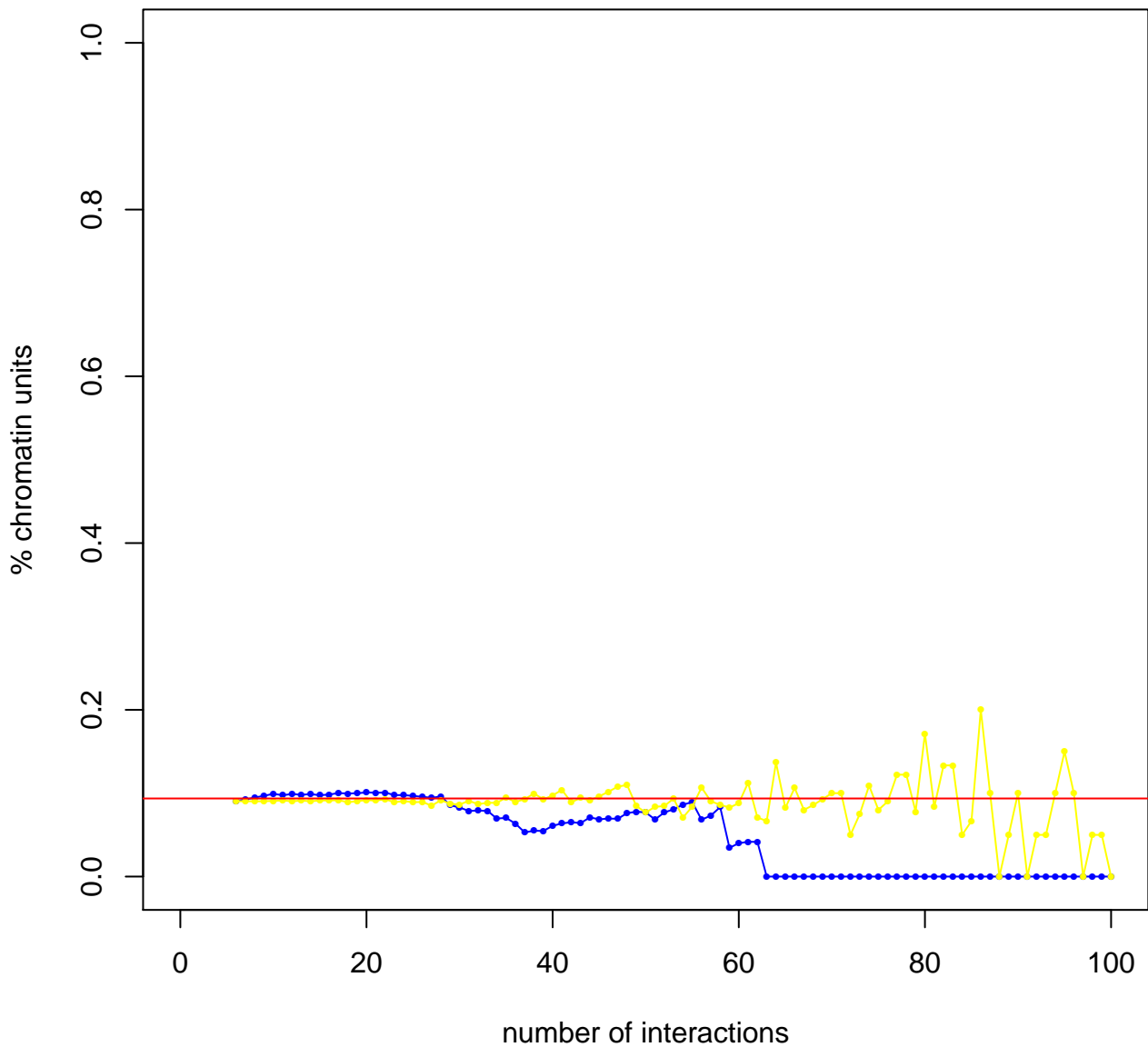

Supplement: Supplementary file 3 — A folder named SB-06-S3 contains 105 overlapping plot for each TF. (ZIP 624 kb) [file 12918_2018_643_MOESM3_ESM.zip › SB-06-S3/HAP2.pdf]

# HAP3

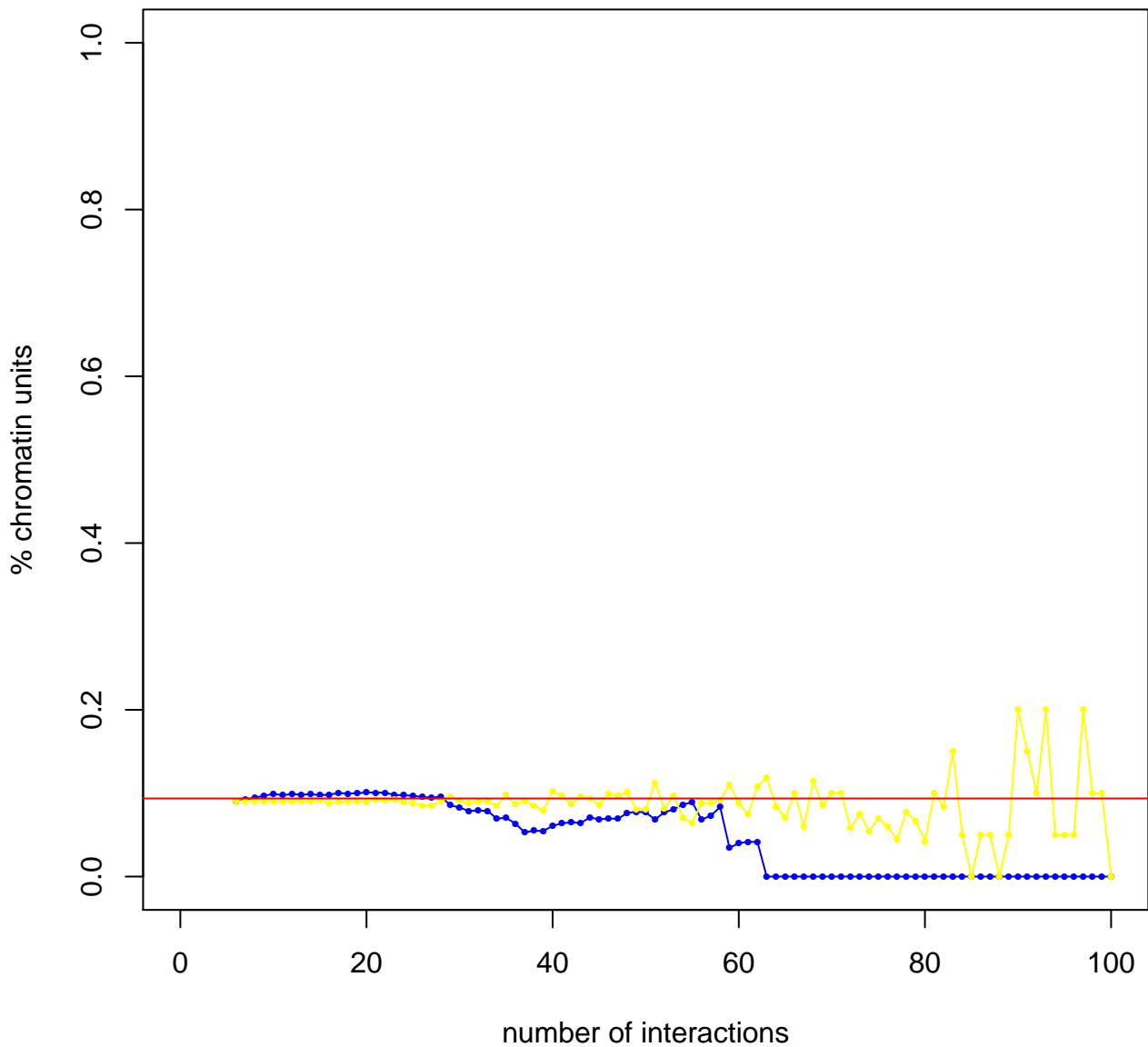

Supplement: Supplementary file 3 — A folder named SB-06-S3 contains 105 overlapping plot for each TF. (ZIP 624 kb) [file 12918_2018_643_MOESM3_ESM.zip › SB-06-S3/HAP3.pdf]

# HAP4

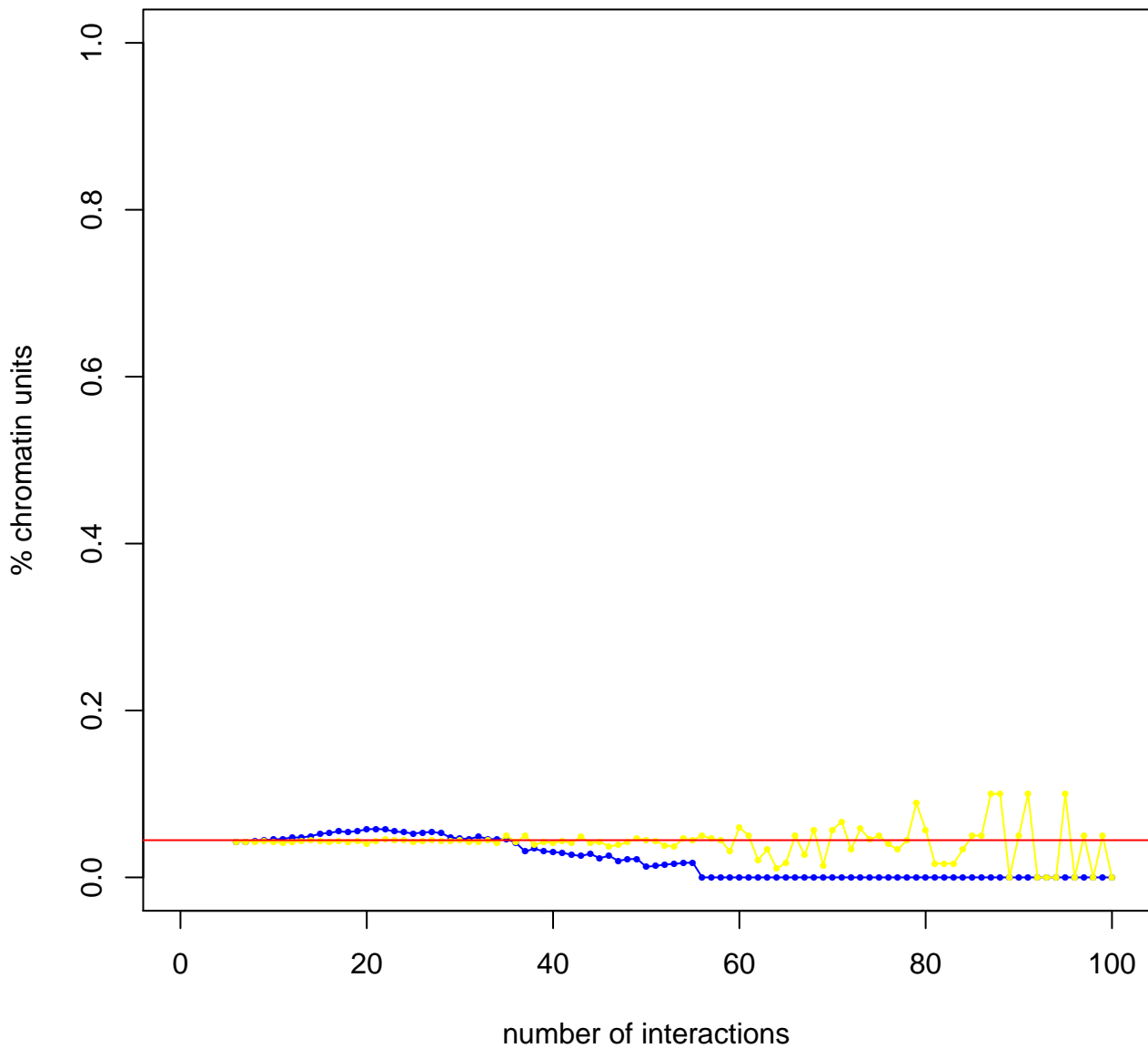

Supplement: Supplementary file 3 — A folder named SB-06-S3 contains 105 overlapping plot for each TF. (ZIP 624 kb) [file 12918_2018_643_MOESM3_ESM.zip › SB-06-S3/HAP4.pdf]

# HAP5

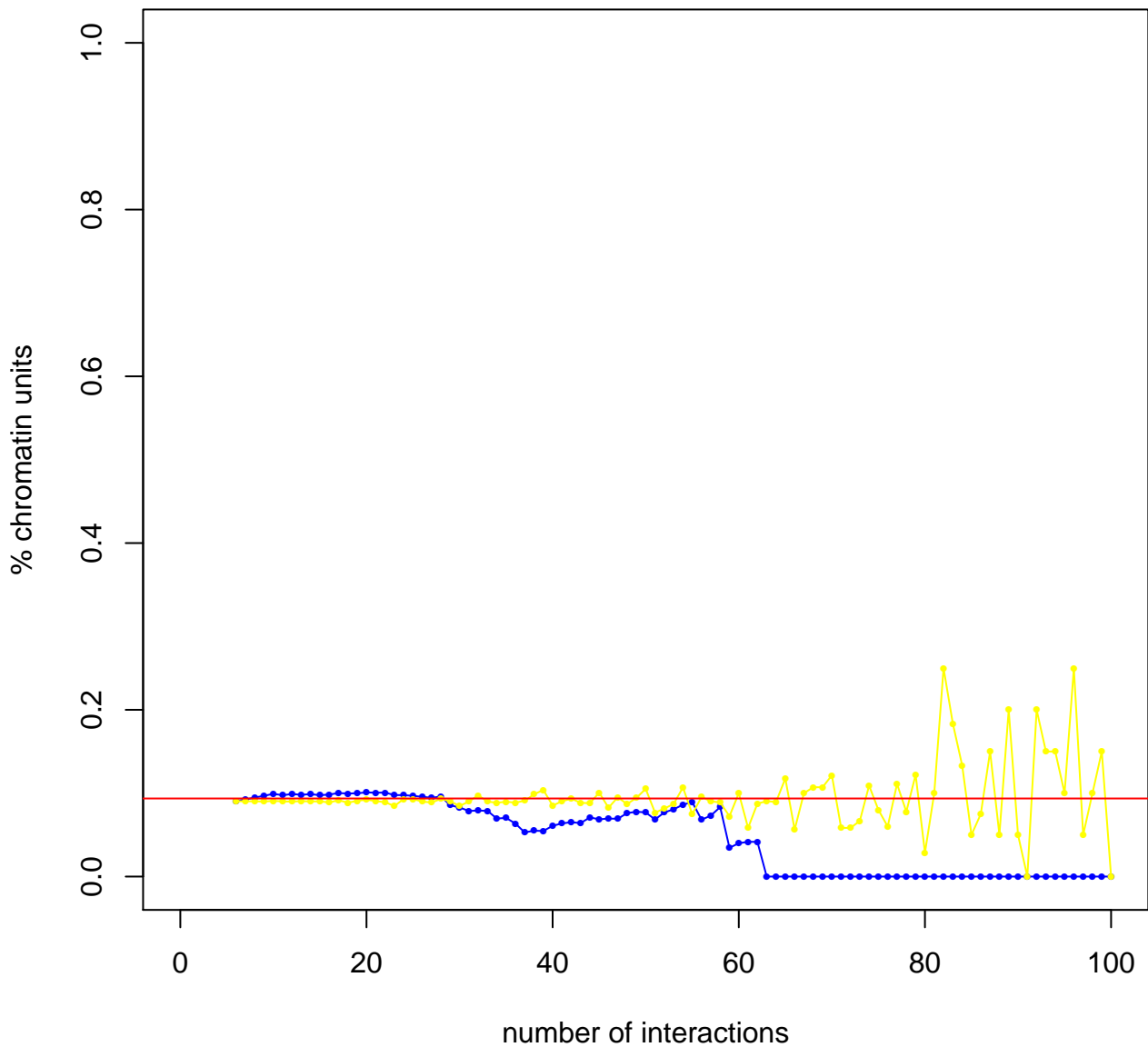

Supplement: Supplementary file 3 — A folder named SB-06-S3 contains 105 overlapping plot for each TF. (ZIP 624 kb) [file 12918_2018_643_MOESM3_ESM.zip › SB-06-S3/HAP5.pdf]

# HSF1

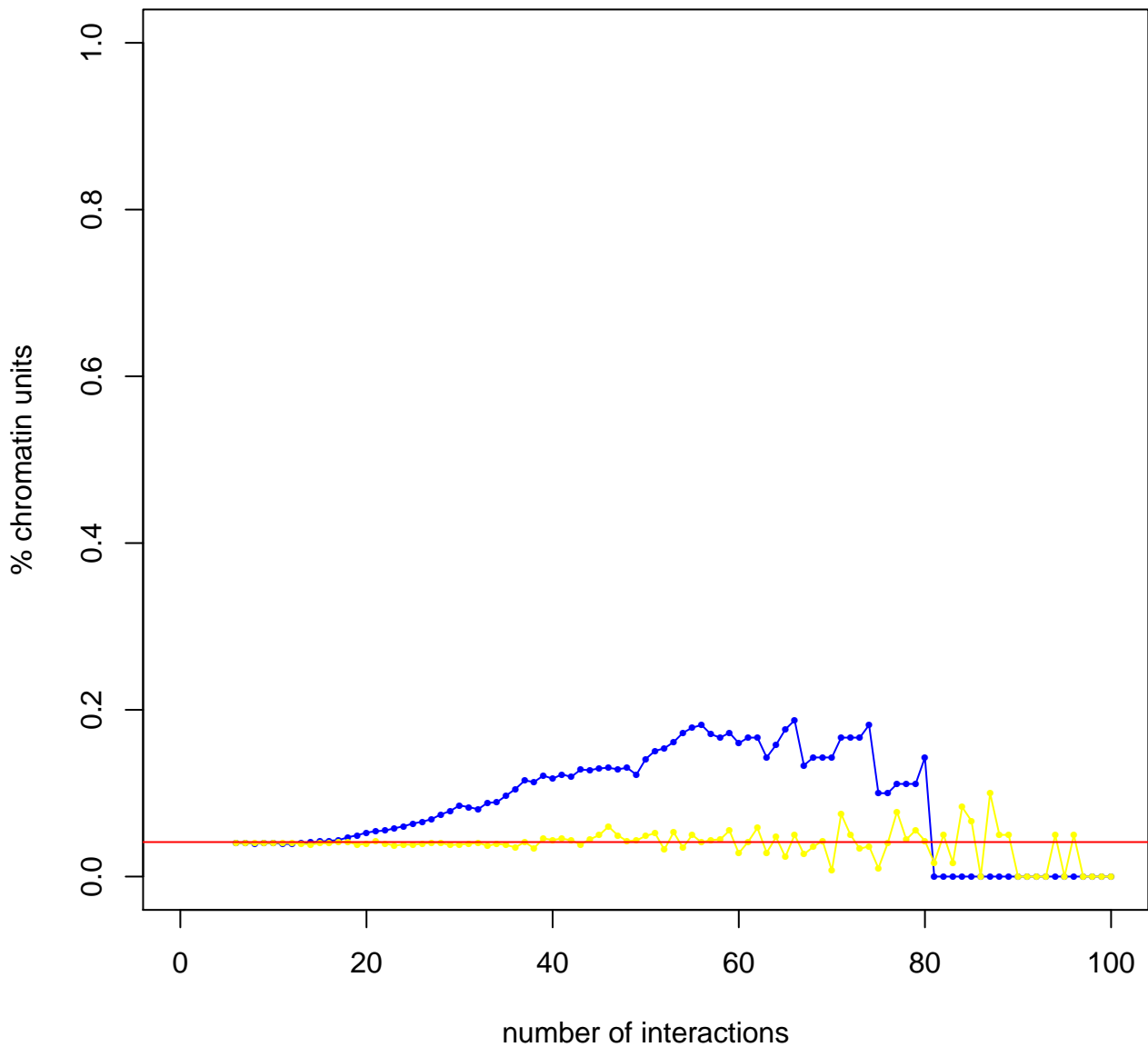

Supplement: Supplementary file 3 — A folder named SB-06-S3 contains 105 overlapping plot for each TF. (ZIP 624 kb) [file 12918_2018_643_MOESM3_ESM.zip › SB-06-S3/HSF1.pdf]

# INO2

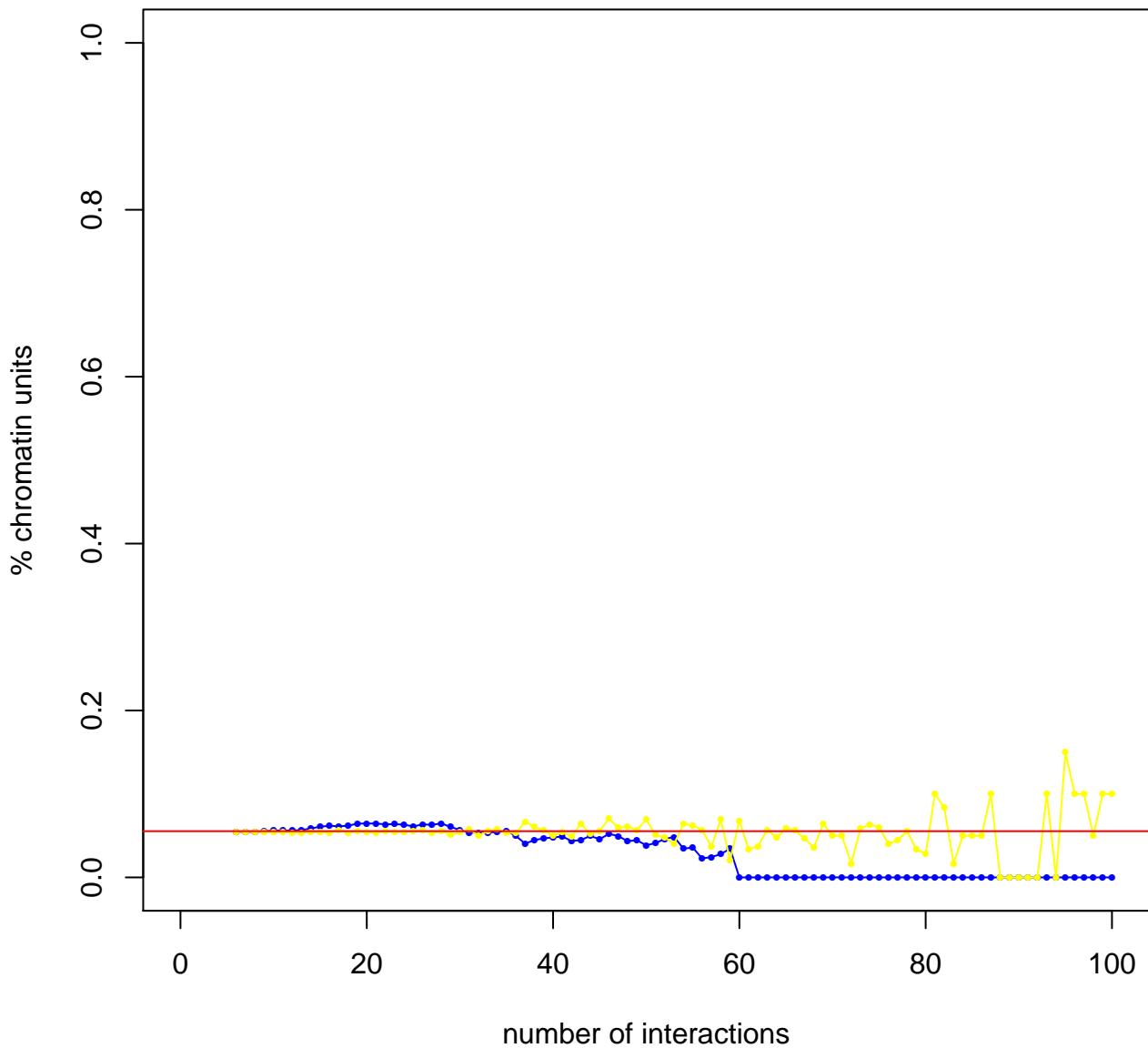

Supplement: Supplementary file 3 — A folder named SB-06-S3 contains 105 overlapping plot for each TF. (ZIP 624 kb) [file 12918_2018_643_MOESM3_ESM.zip › SB-06-S3/INO2.pdf]

# INO4

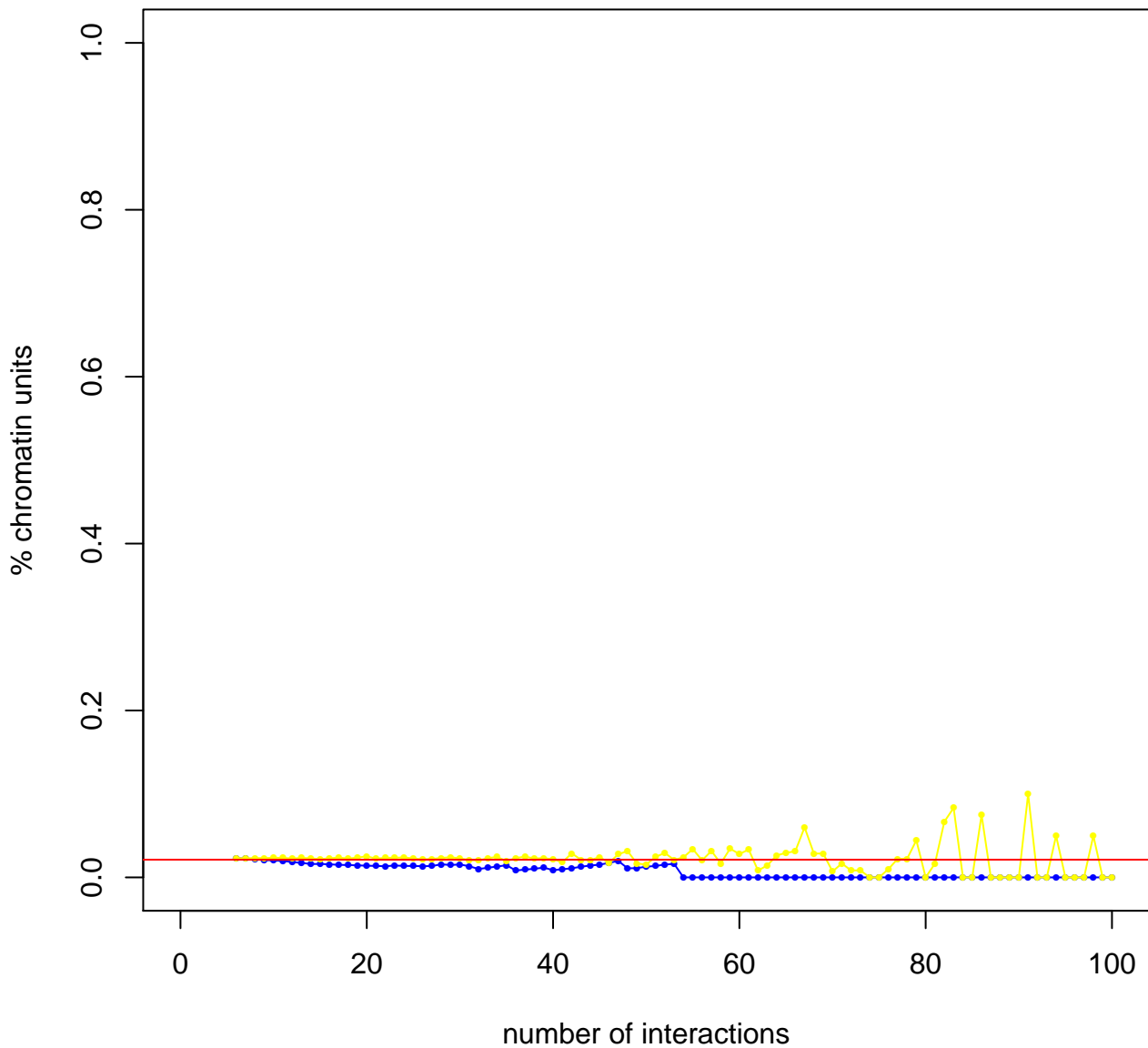

Supplement: Supplementary file 3 — A folder named SB-06-S3 contains 105 overlapping plot for each TF. (ZIP 624 kb) [file 12918_2018_643_MOESM3_ESM.zip › SB-06-S3/INO4.pdf]

# LEU3

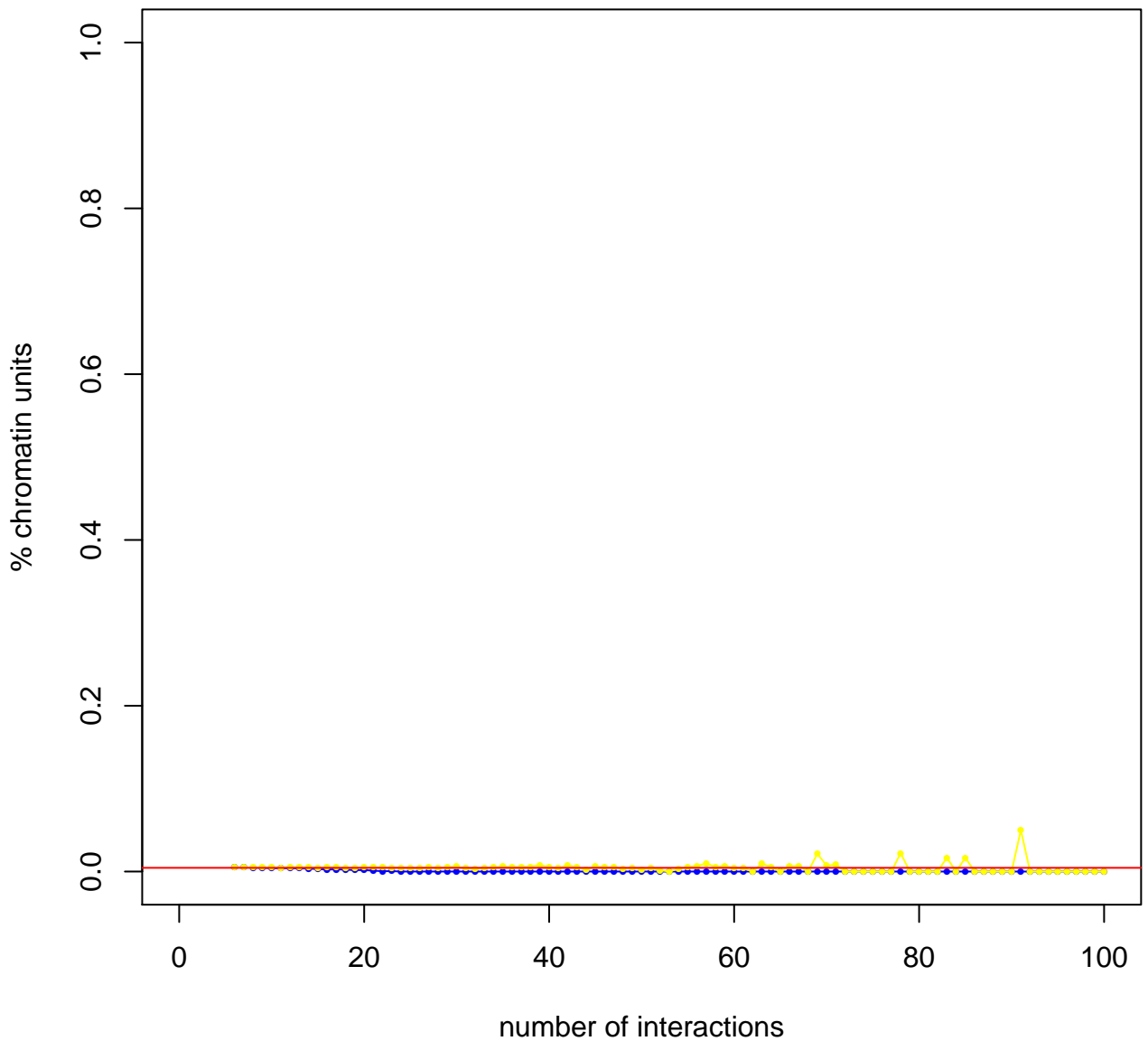

Supplement: Supplementary file 3 — A folder named SB-06-S3 contains 105 overlapping plot for each TF. (ZIP 624 kb) [file 12918_2018_643_MOESM3_ESM.zip › SB-06-S3/LEU3.pdf]

# MAC1

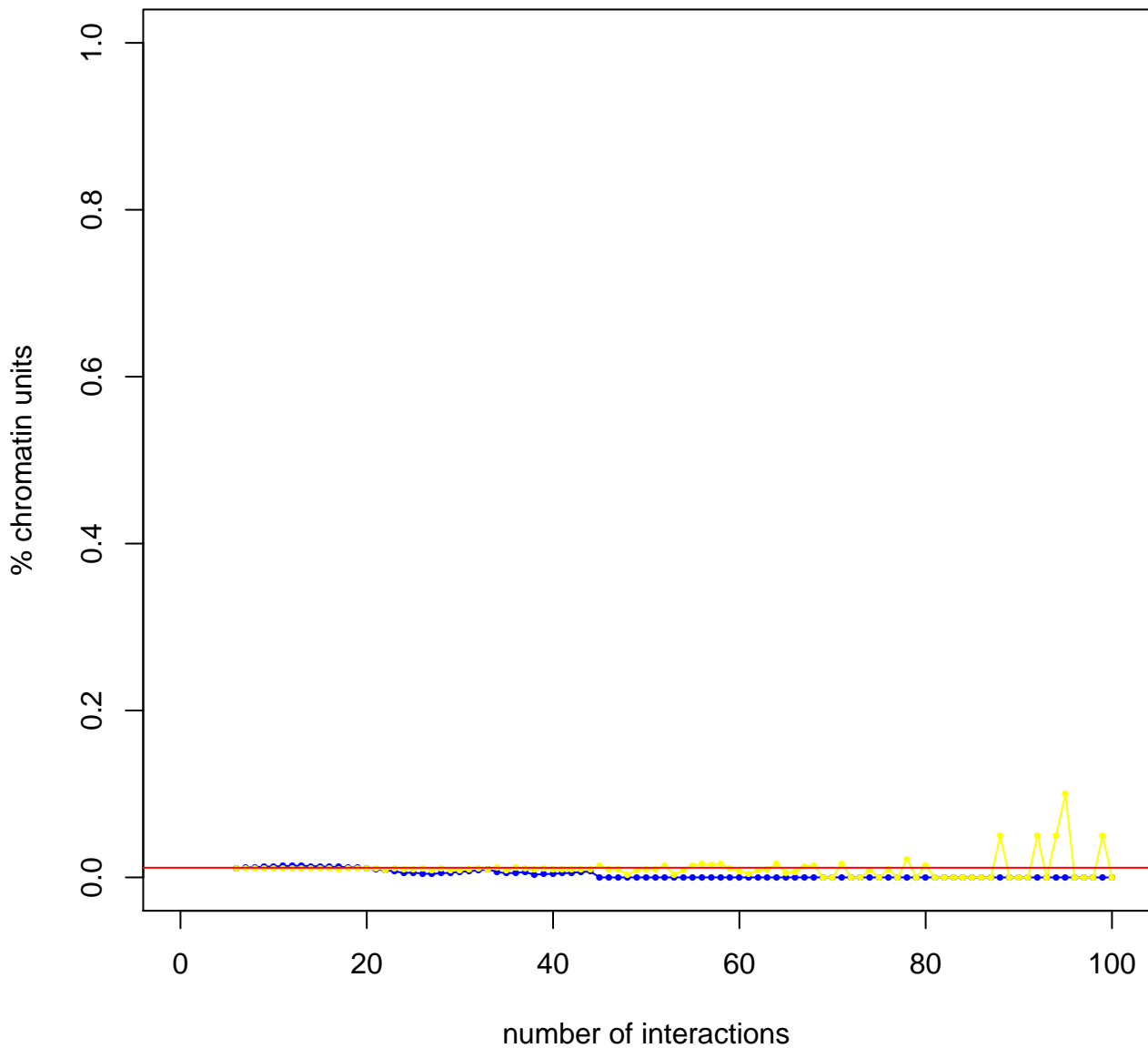

Supplement: Supplementary file 3 — A folder named SB-06-S3 contains 105 overlapping plot for each TF. (ZIP 624 kb) [file 12918_2018_643_MOESM3_ESM.zip › SB-06-S3/MAC1.pdf]

# MBP1

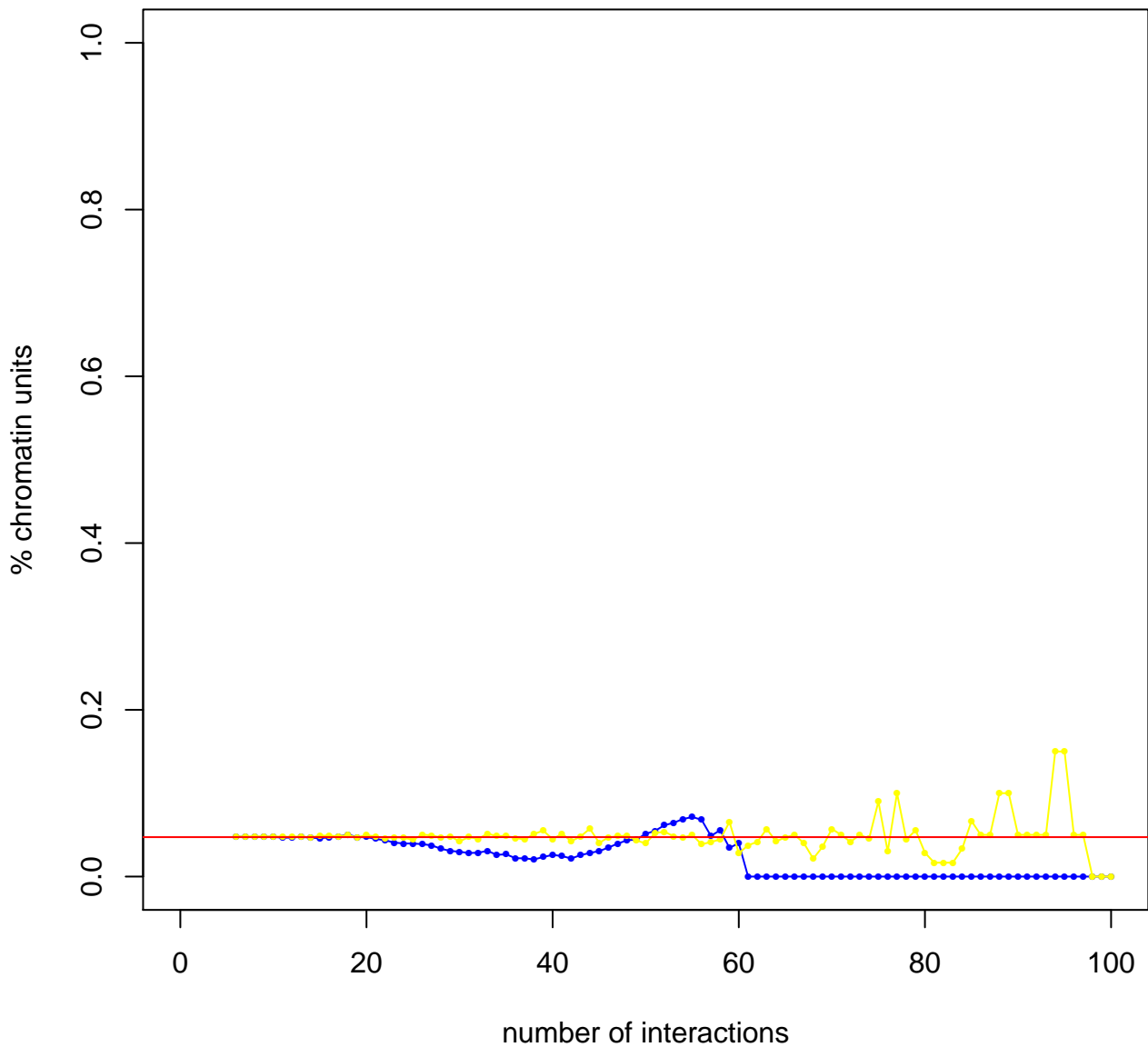

Supplement: Supplementary file 3 — A folder named SB-06-S3 contains 105 overlapping plot for each TF. (ZIP 624 kb) [file 12918_2018_643_MOESM3_ESM.zip › SB-06-S3/MBP1.pdf]

# MCM1

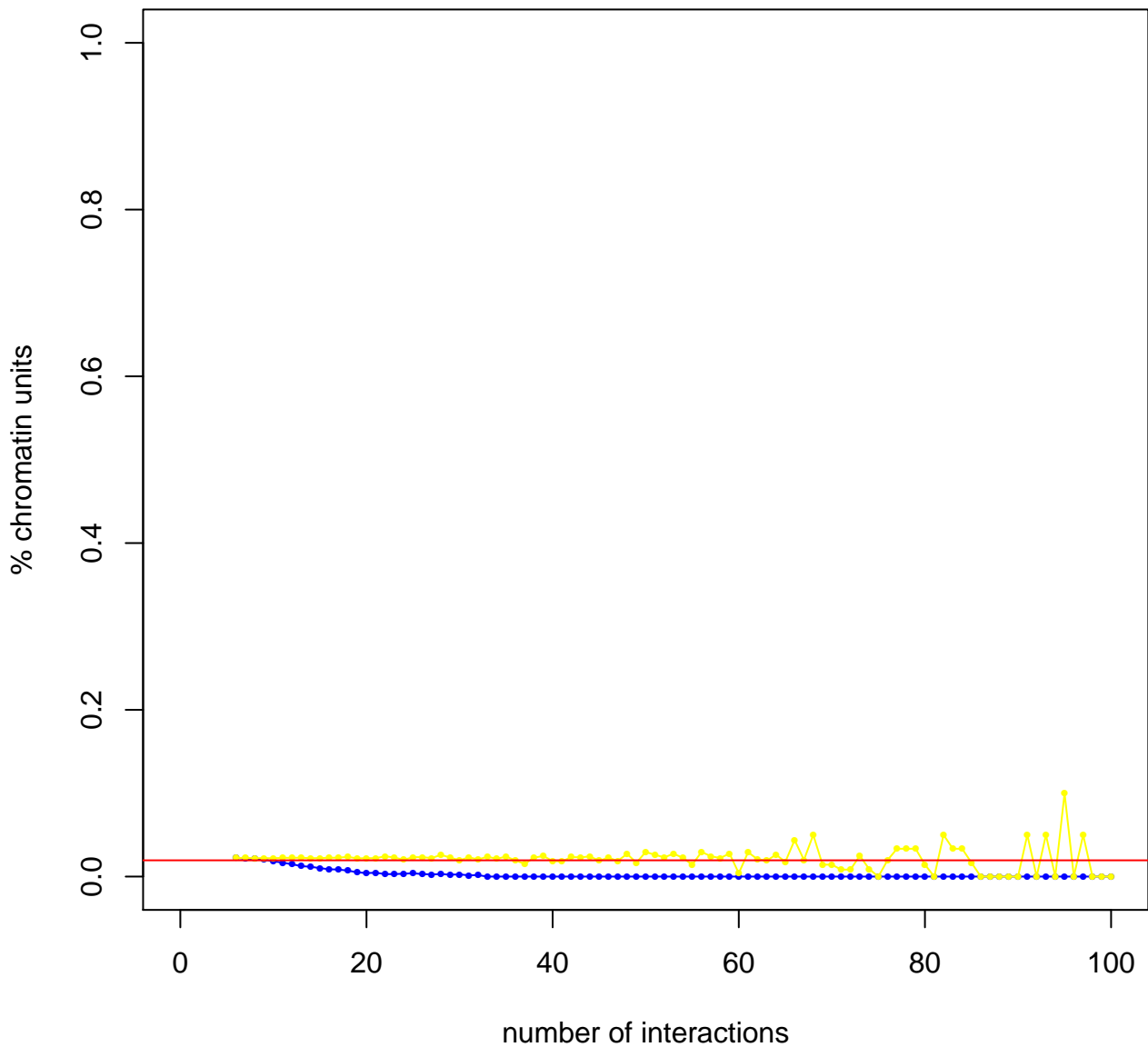

Supplement: Supplementary file 3 — A folder named SB-06-S3 contains 105 overlapping plot for each TF. (ZIP 624 kb) [file 12918_2018_643_MOESM3_ESM.zip › SB-06-S3/MCM1.pdf]

# MET31

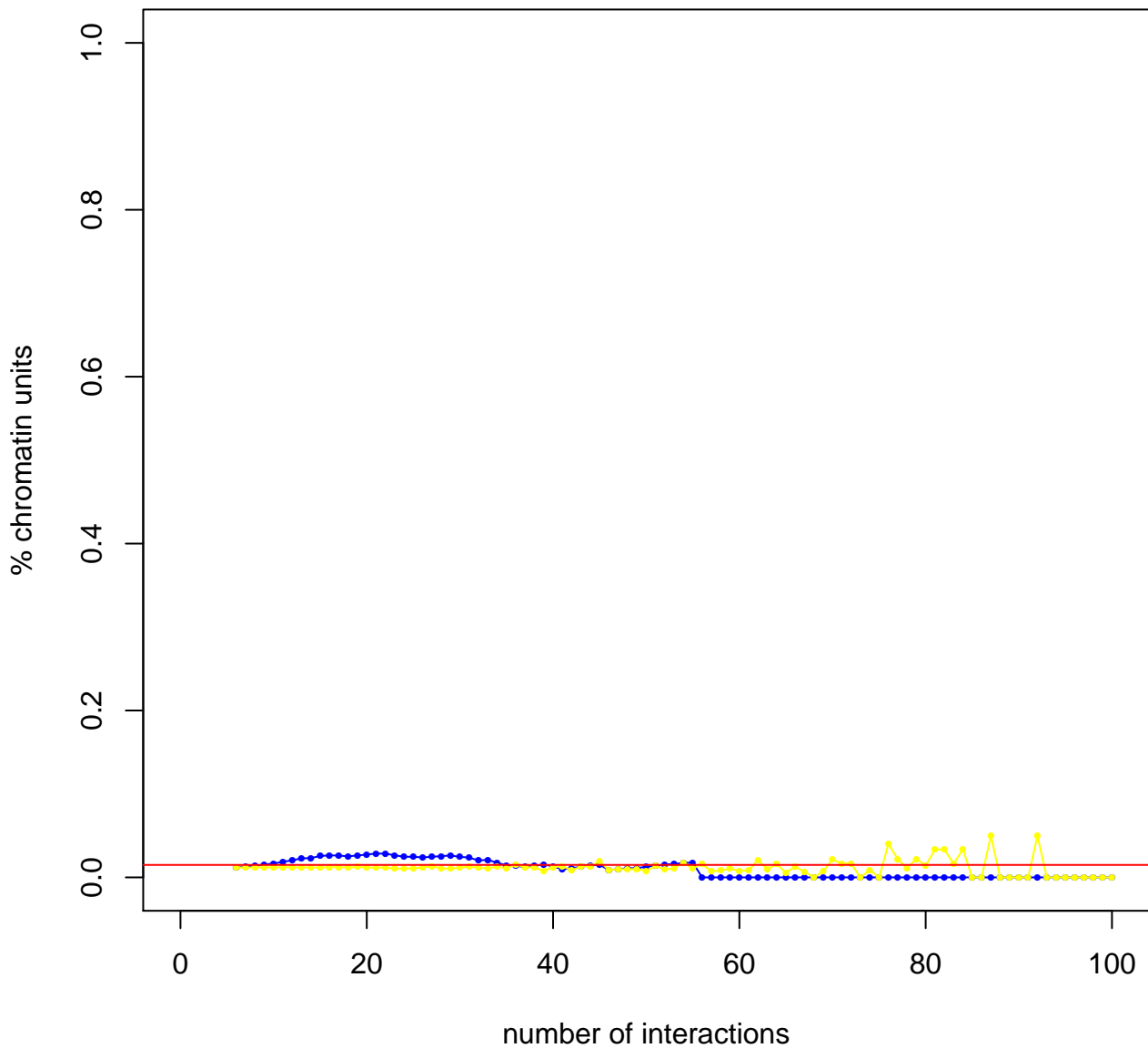

Supplement: Supplementary file 3 — A folder named SB-06-S3 contains 105 overlapping plot for each TF. (ZIP 624 kb) [file 12918_2018_643_MOESM3_ESM.zip › SB-06-S3/MET31.pdf]

# MET32

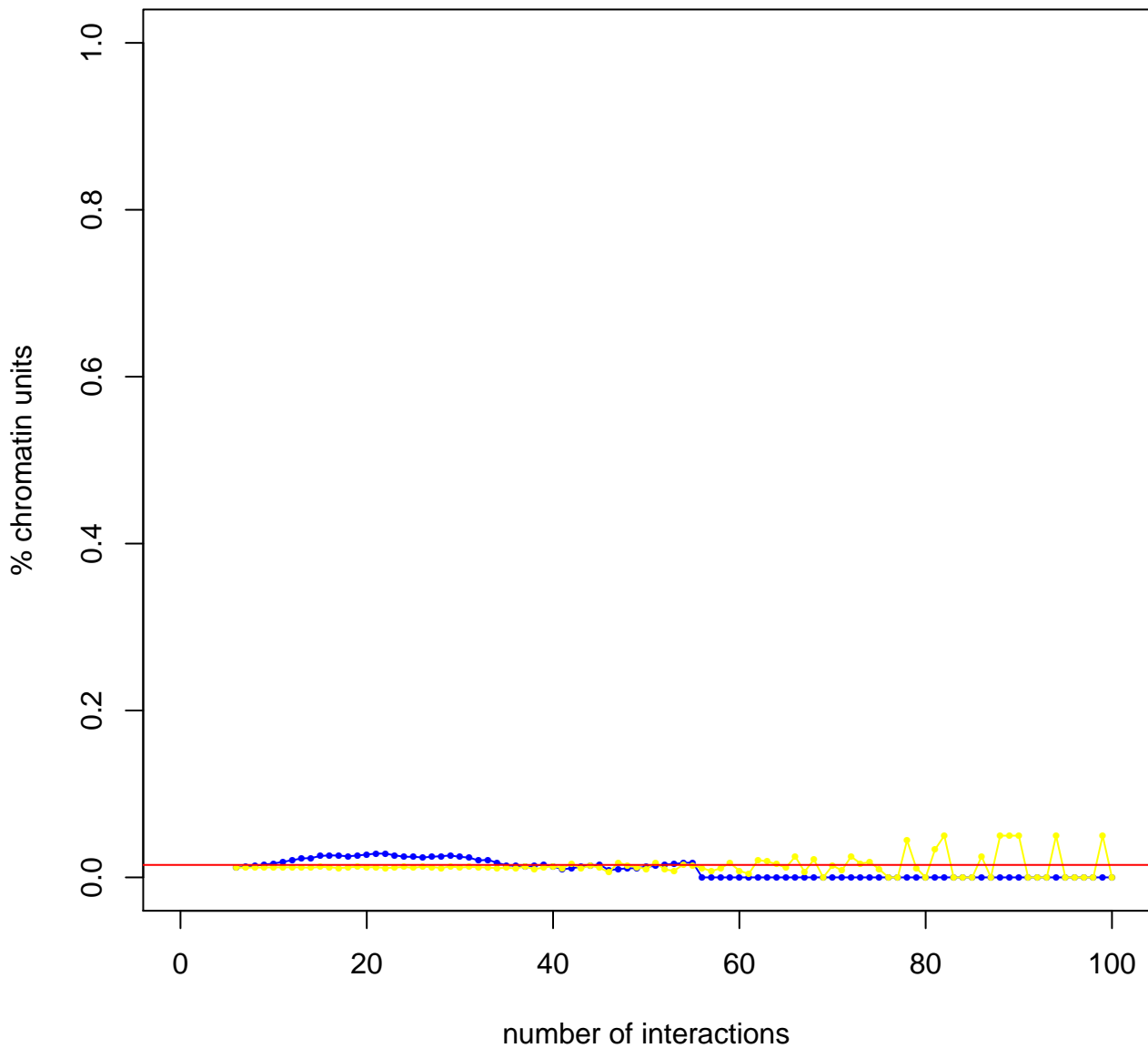

Supplement: Supplementary file 3 — A folder named SB-06-S3 contains 105 overlapping plot for each TF. (ZIP 624 kb) [file 12918_2018_643_MOESM3_ESM.zip › SB-06-S3/MET32.pdf]

# MET4

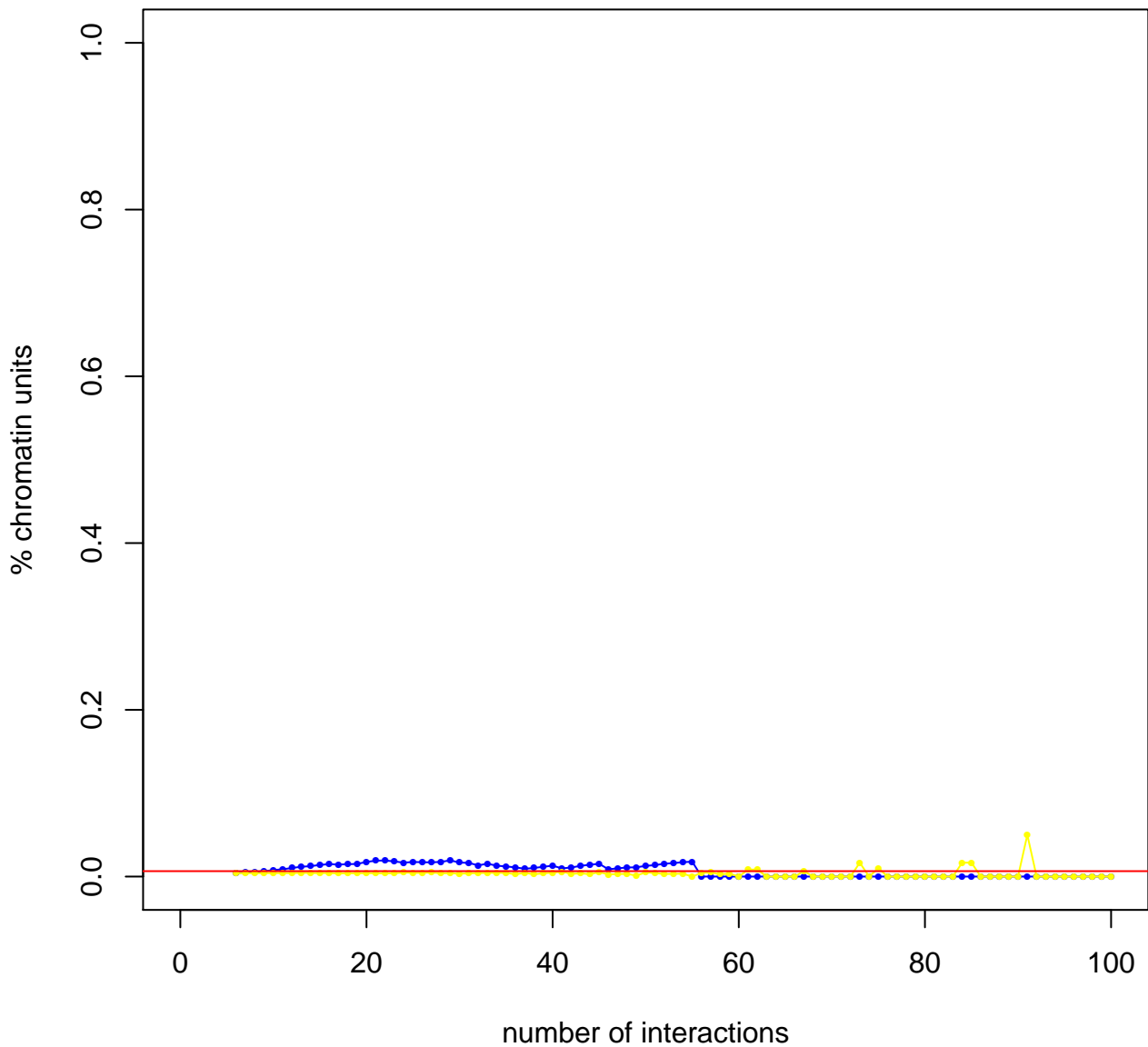

Supplement: Supplementary file 3 — A folder named SB-06-S3 contains 105 overlapping plot for each TF. (ZIP 624 kb) [file 12918_2018_643_MOESM3_ESM.zip › SB-06-S3/MET4.pdf]

# MOT3

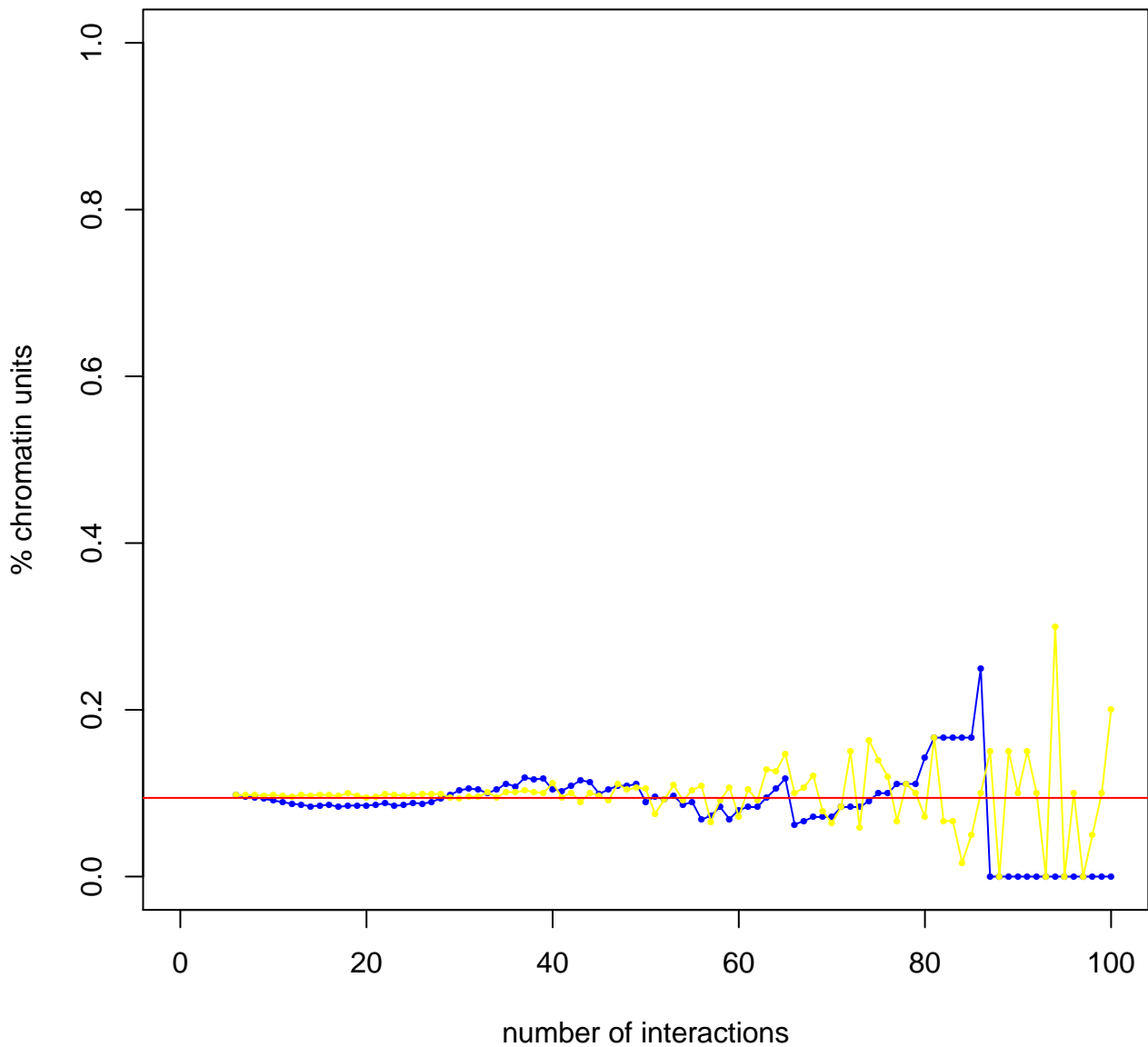

Supplement: Supplementary file 3 — A folder named SB-06-S3 contains 105 overlapping plot for each TF. (ZIP 624 kb) [file 12918_2018_643_MOESM3_ESM.zip › SB-06-S3/MOT3.pdf]

# MSN2

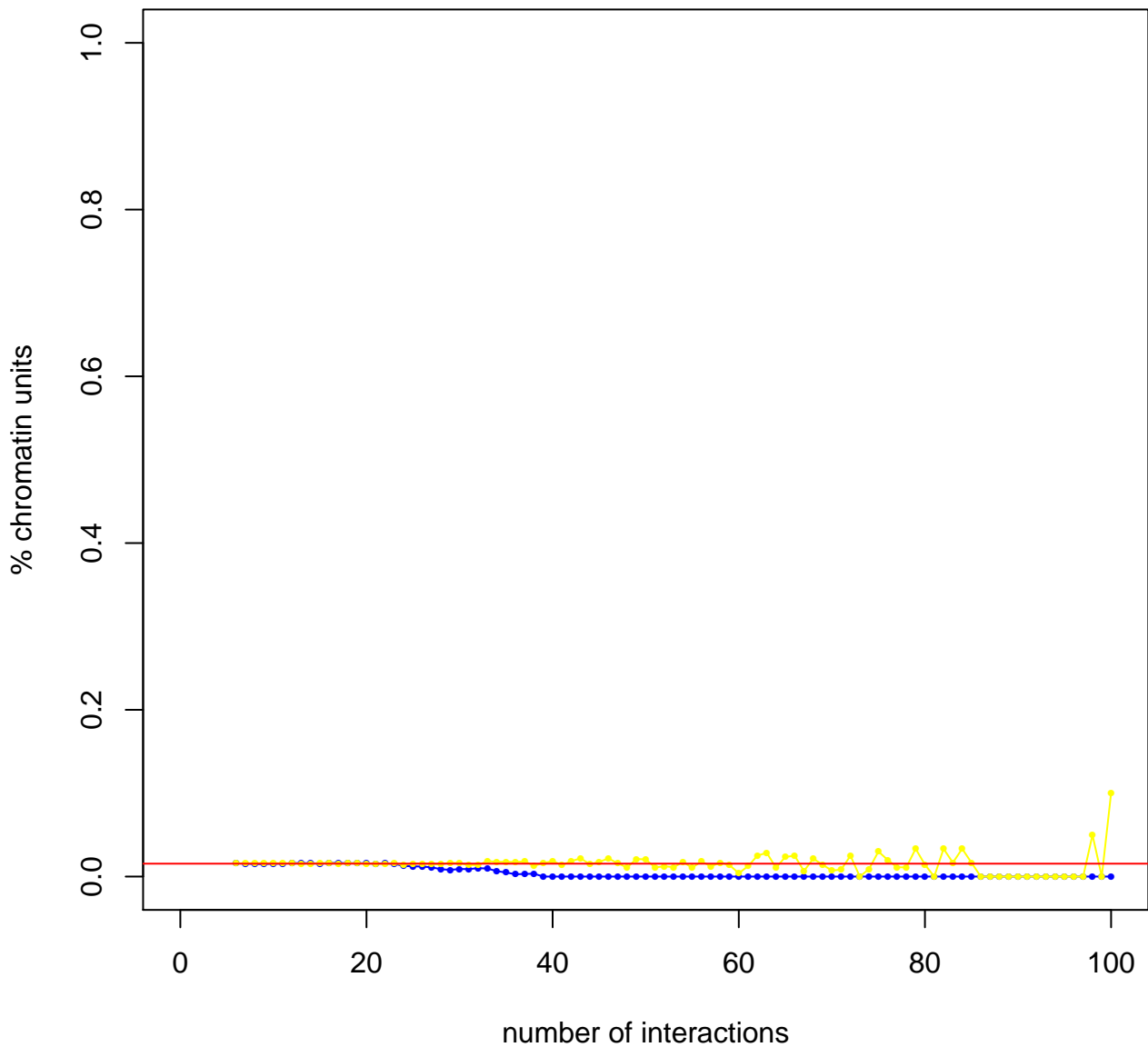

Supplement: Supplementary file 3 — A folder named SB-06-S3 contains 105 overlapping plot for each TF. (ZIP 624 kb) [file 12918_2018_643_MOESM3_ESM.zip › SB-06-S3/MSN2.pdf]

# MSN4

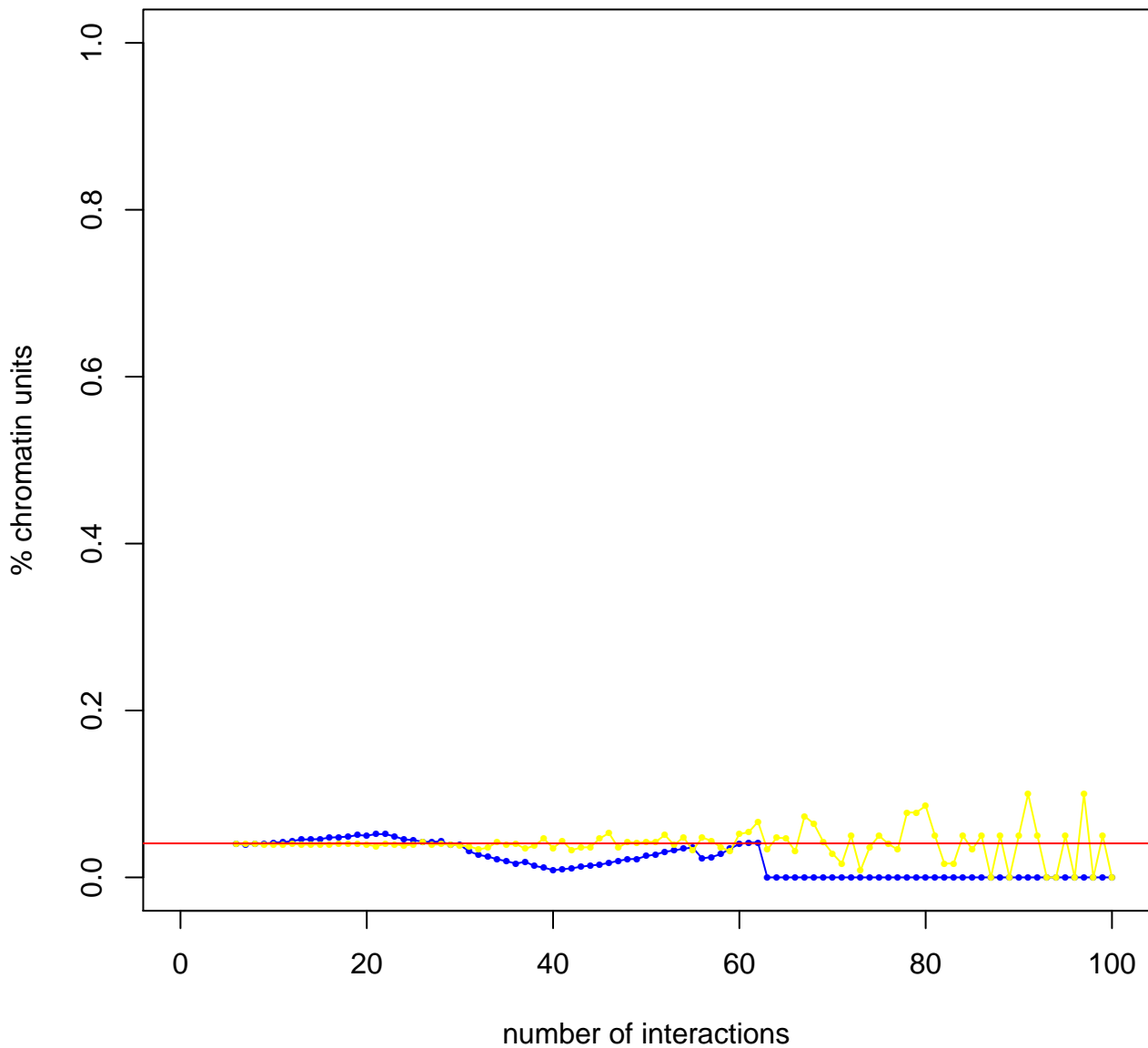

Supplement: Supplementary file 3 — A folder named SB-06-S3 contains 105 overlapping plot for each TF. (ZIP 624 kb) [file 12918_2018_643_MOESM3_ESM.zip › SB-06-S3/MSN4.pdf]

# NDD1

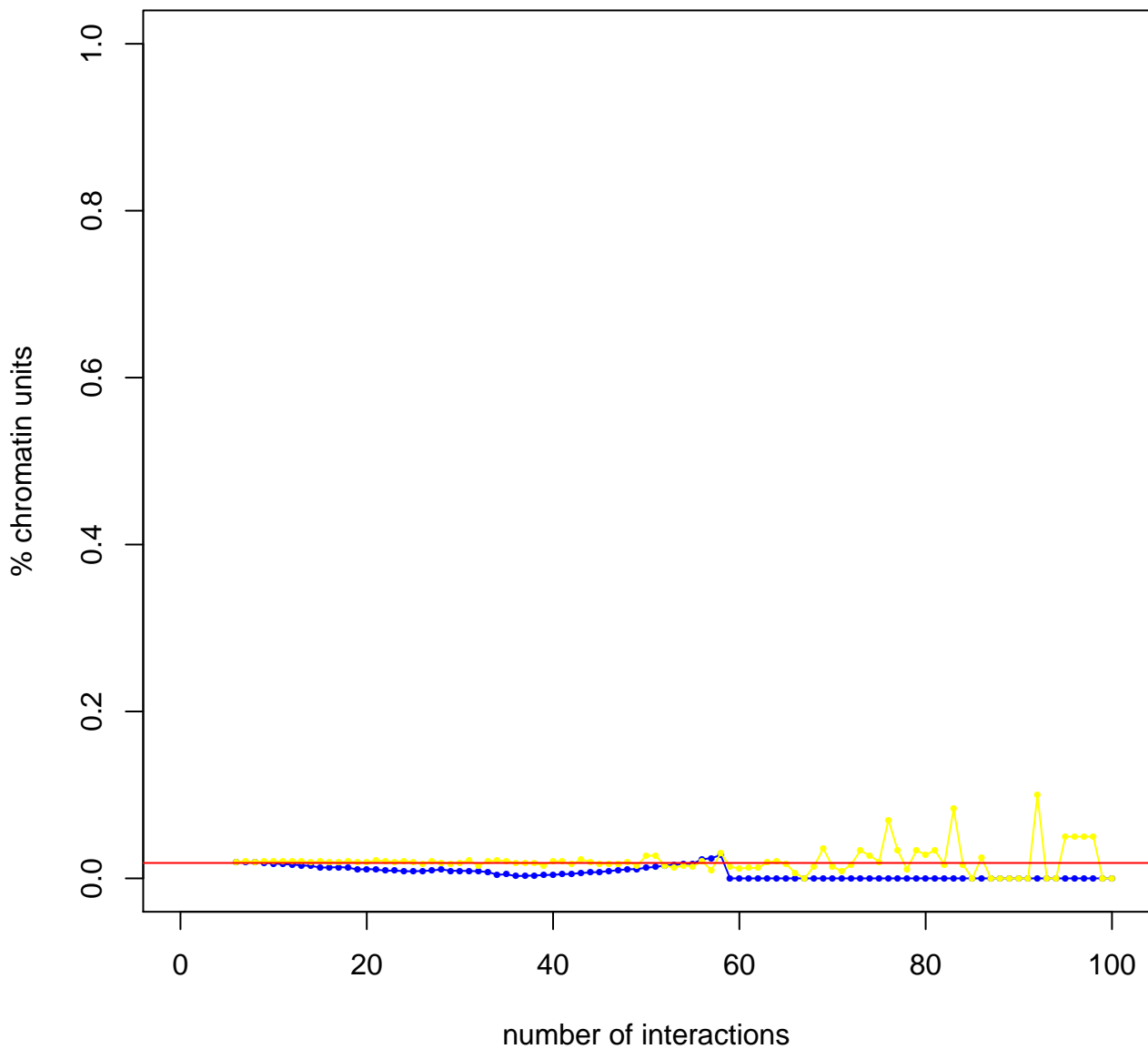

Supplement: Supplementary file 3 — A folder named SB-06-S3 contains 105 overlapping plot for each TF. (ZIP 624 kb) [file 12918_2018_643_MOESM3_ESM.zip › SB-06-S3/NDD1.pdf]

# NRG1

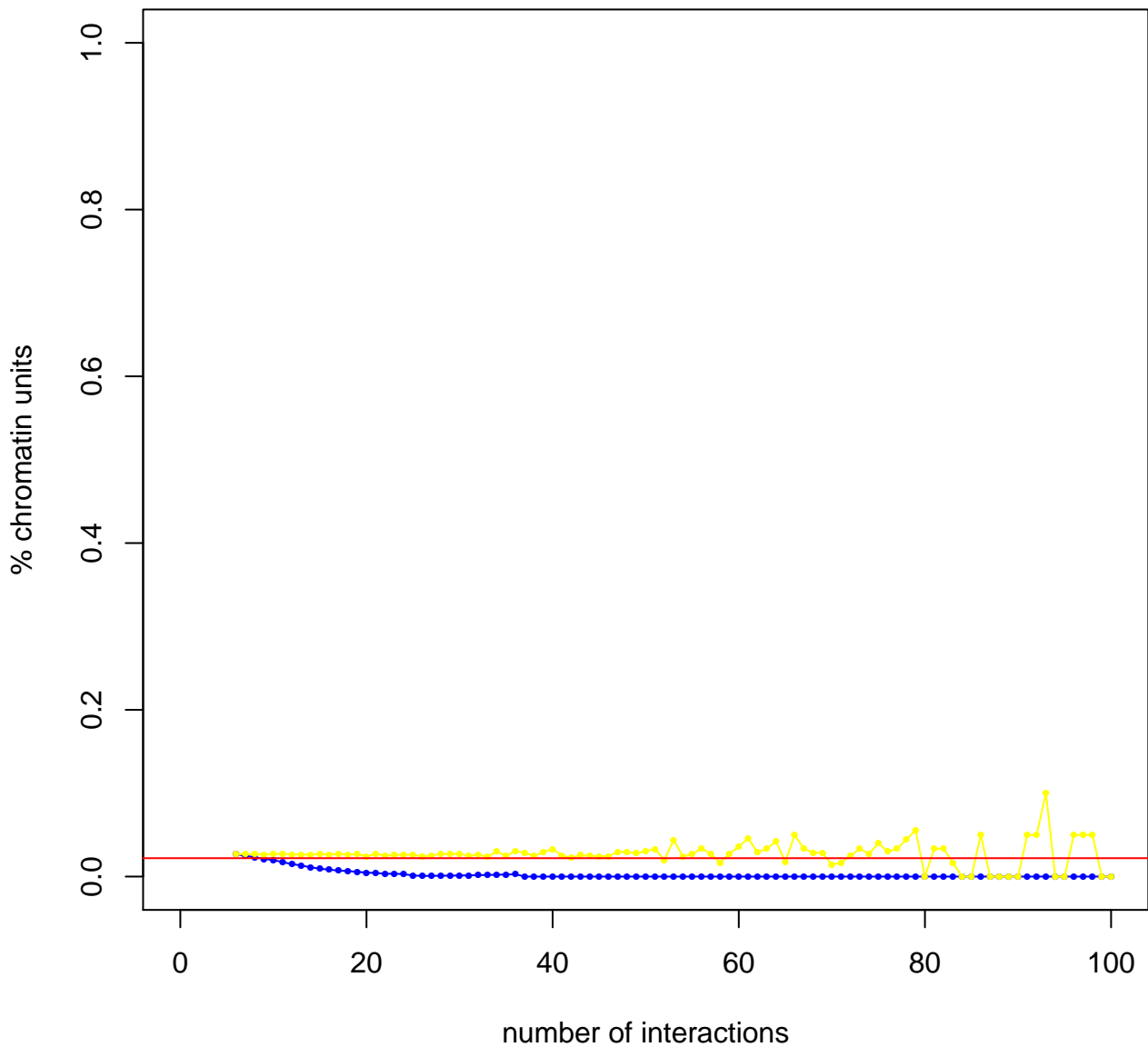

Supplement: Supplementary file 3 — A folder named SB-06-S3 contains 105 overlapping plot for each TF. (ZIP 624 kb) [file 12918_2018_643_MOESM3_ESM.zip › SB-06-S3/NRG1.pdf]

# OPI1

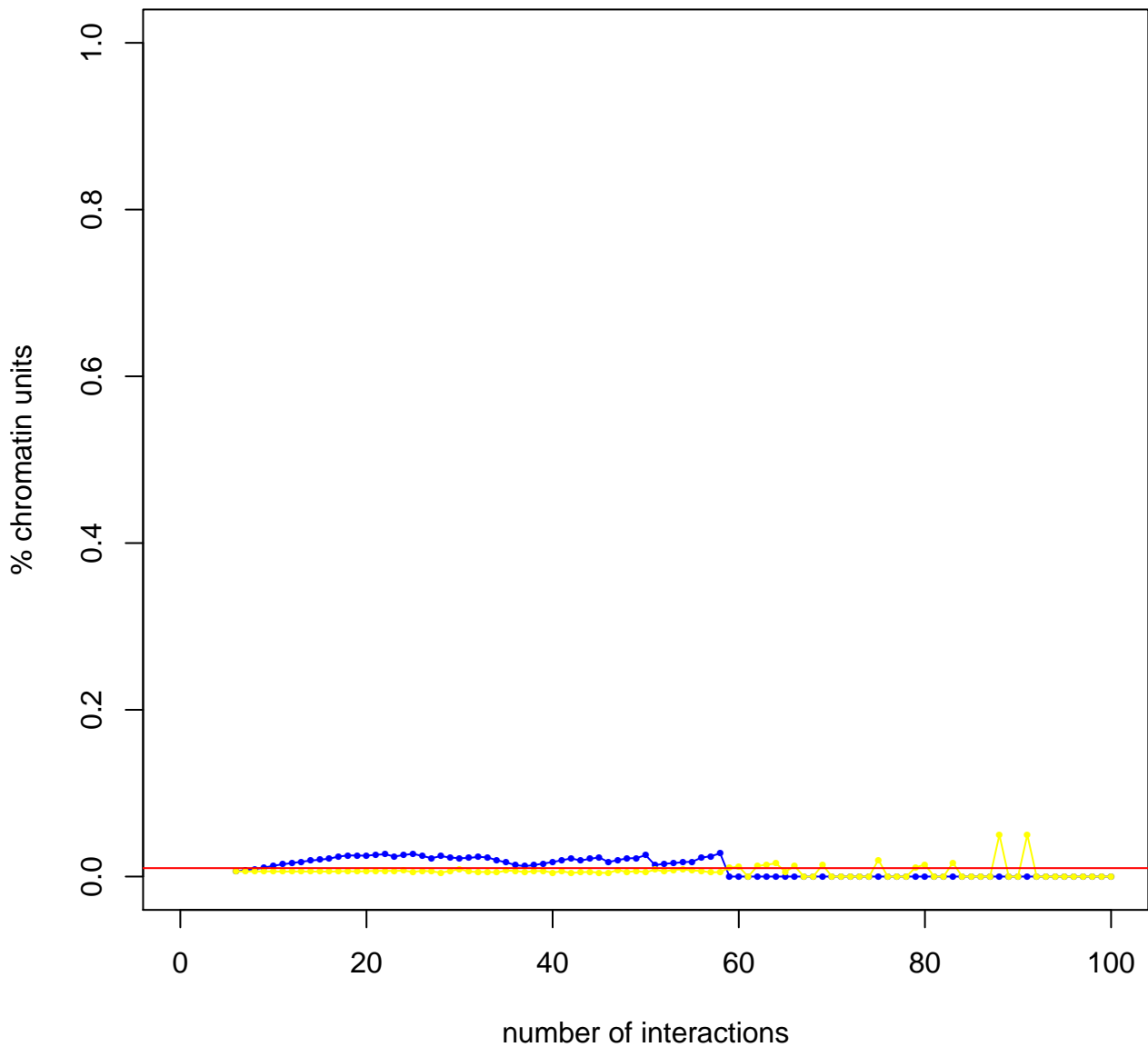

Supplement: Supplementary file 3 — A folder named SB-06-S3 contains 105 overlapping plot for each TF. (ZIP 624 kb) [file 12918_2018_643_MOESM3_ESM.zip › SB-06-S3/OPI1.pdf]

# PDR1

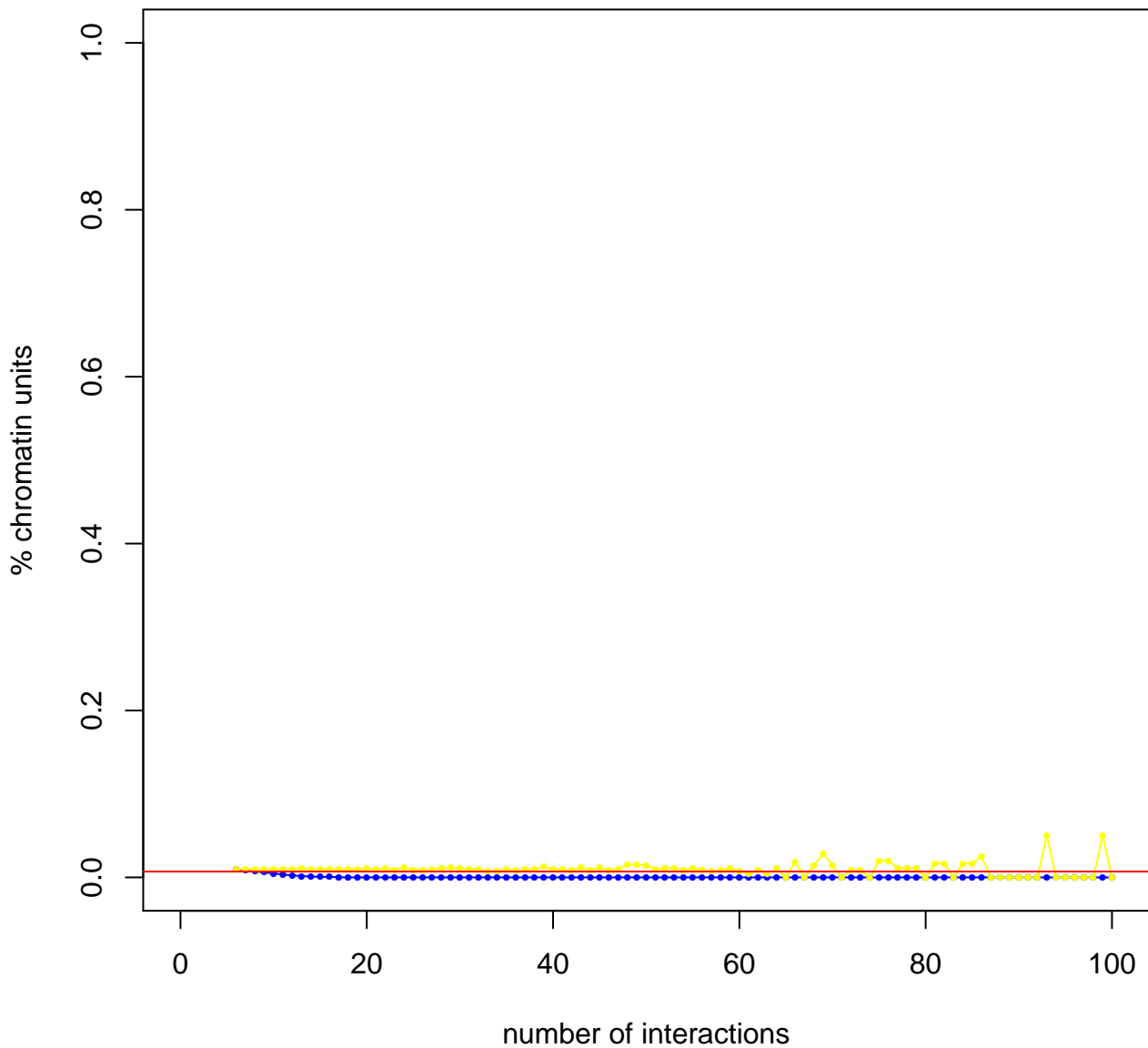

Supplement: Supplementary file 3 — A folder named SB-06-S3 contains 105 overlapping plot for each TF. (ZIP 624 kb) [file 12918_2018_643_MOESM3_ESM.zip › SB-06-S3/PDR1.pdf]

# PDR3

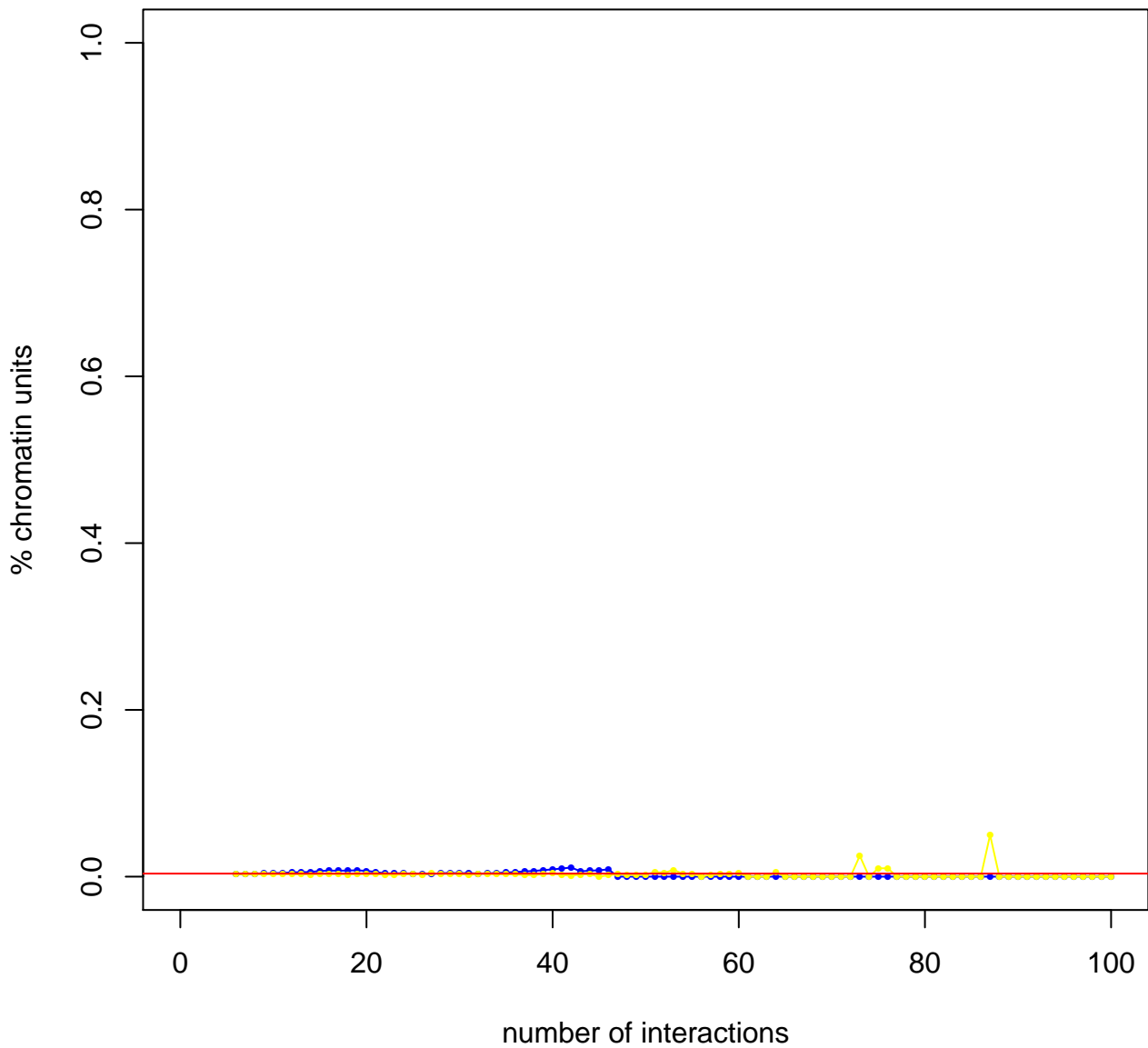

Supplement: Supplementary file 3 — A folder named SB-06-S3 contains 105 overlapping plot for each TF. (ZIP 624 kb) [file 12918_2018_643_MOESM3_ESM.zip › SB-06-S3/PDR3.pdf]

# PHD1

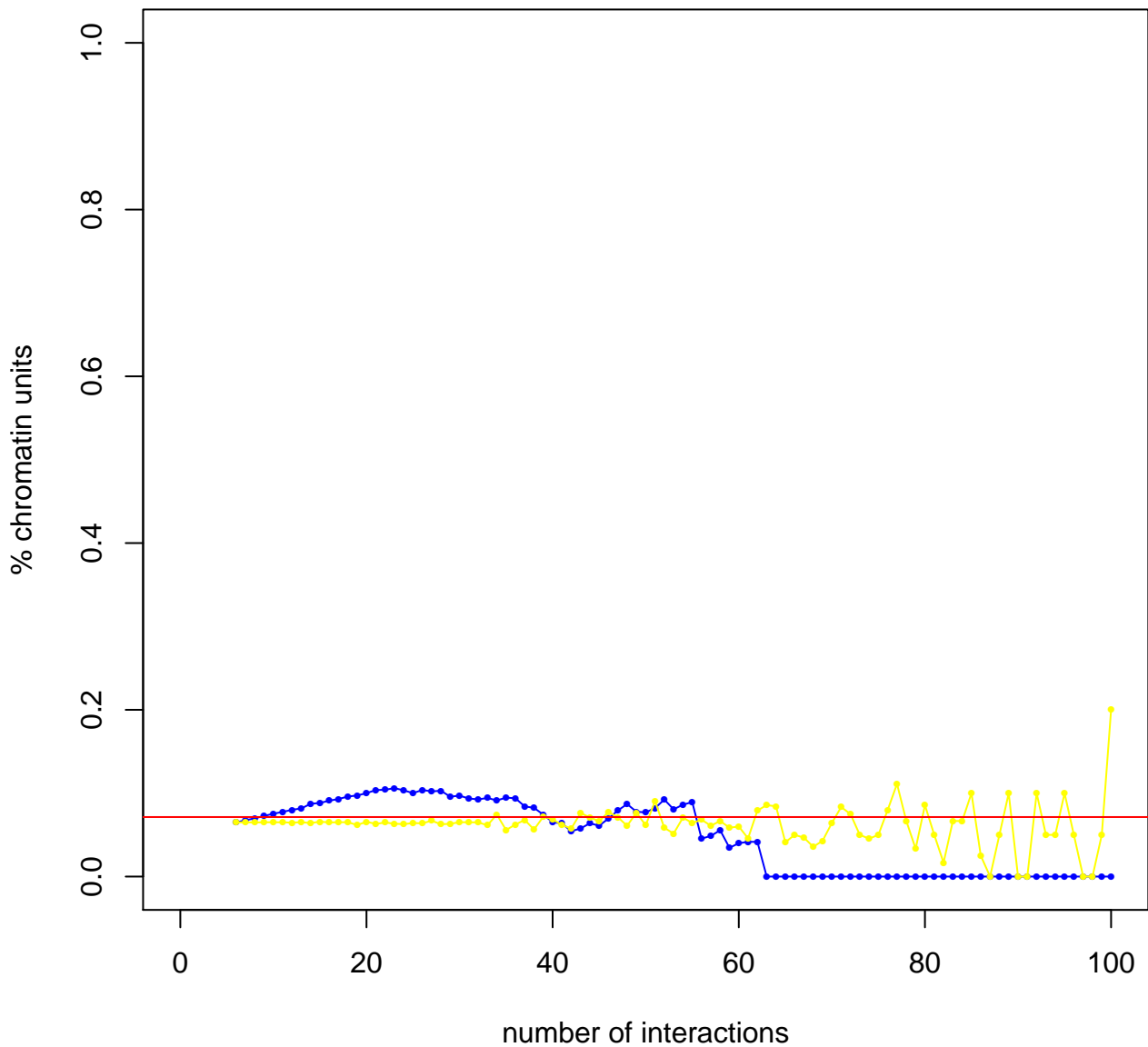

Supplement: Supplementary file 3 — A folder named SB-06-S3 contains 105 overlapping plot for each TF. (ZIP 624 kb) [file 12918_2018_643_MOESM3_ESM.zip › SB-06-S3/PHD1.pdf]

# PHO2

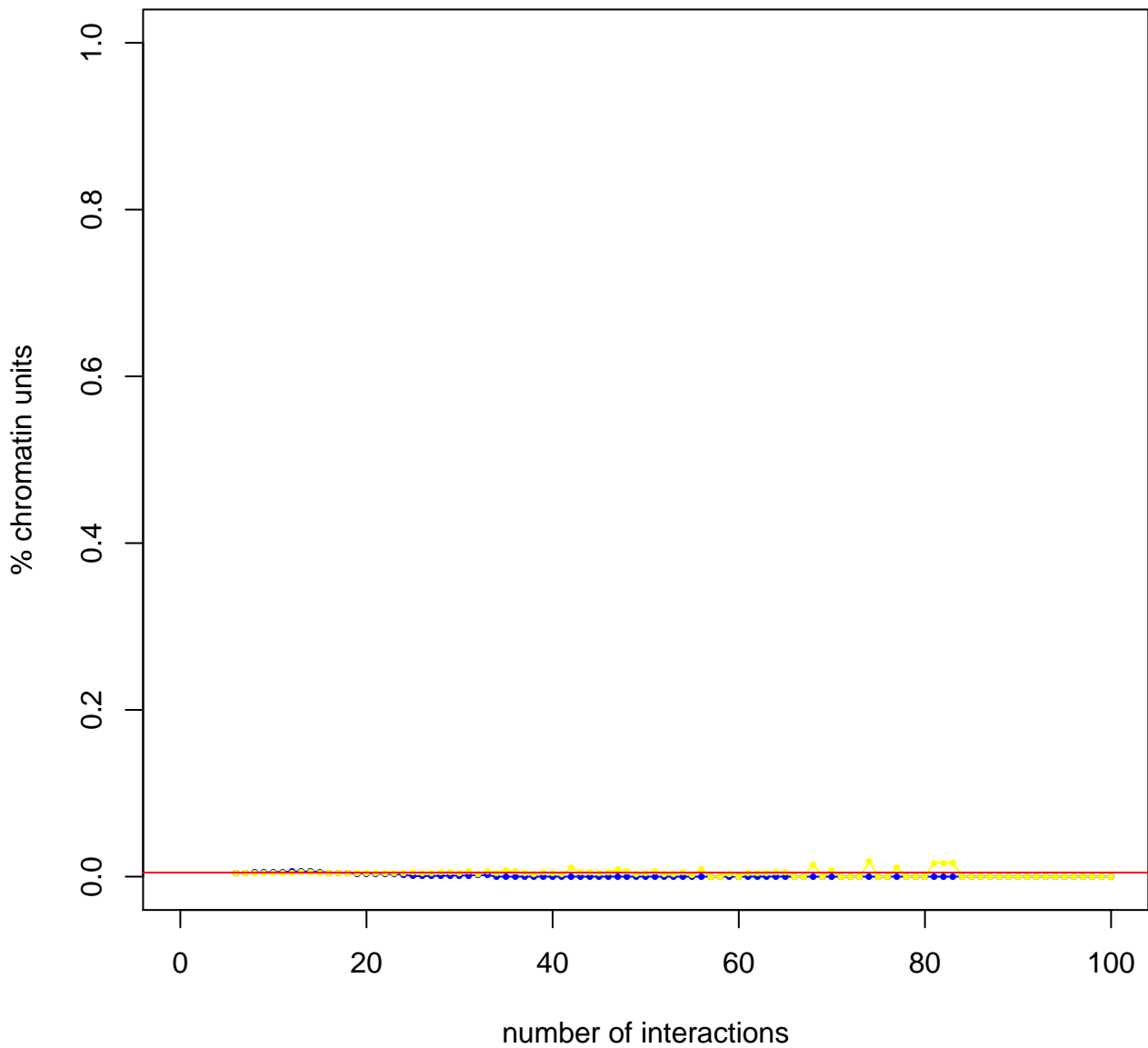

Supplement: Supplementary file 3 — A folder named SB-06-S3 contains 105 overlapping plot for each TF. (ZIP 624 kb) [file 12918_2018_643_MOESM3_ESM.zip › SB-06-S3/PHO2.pdf]

# PHO4

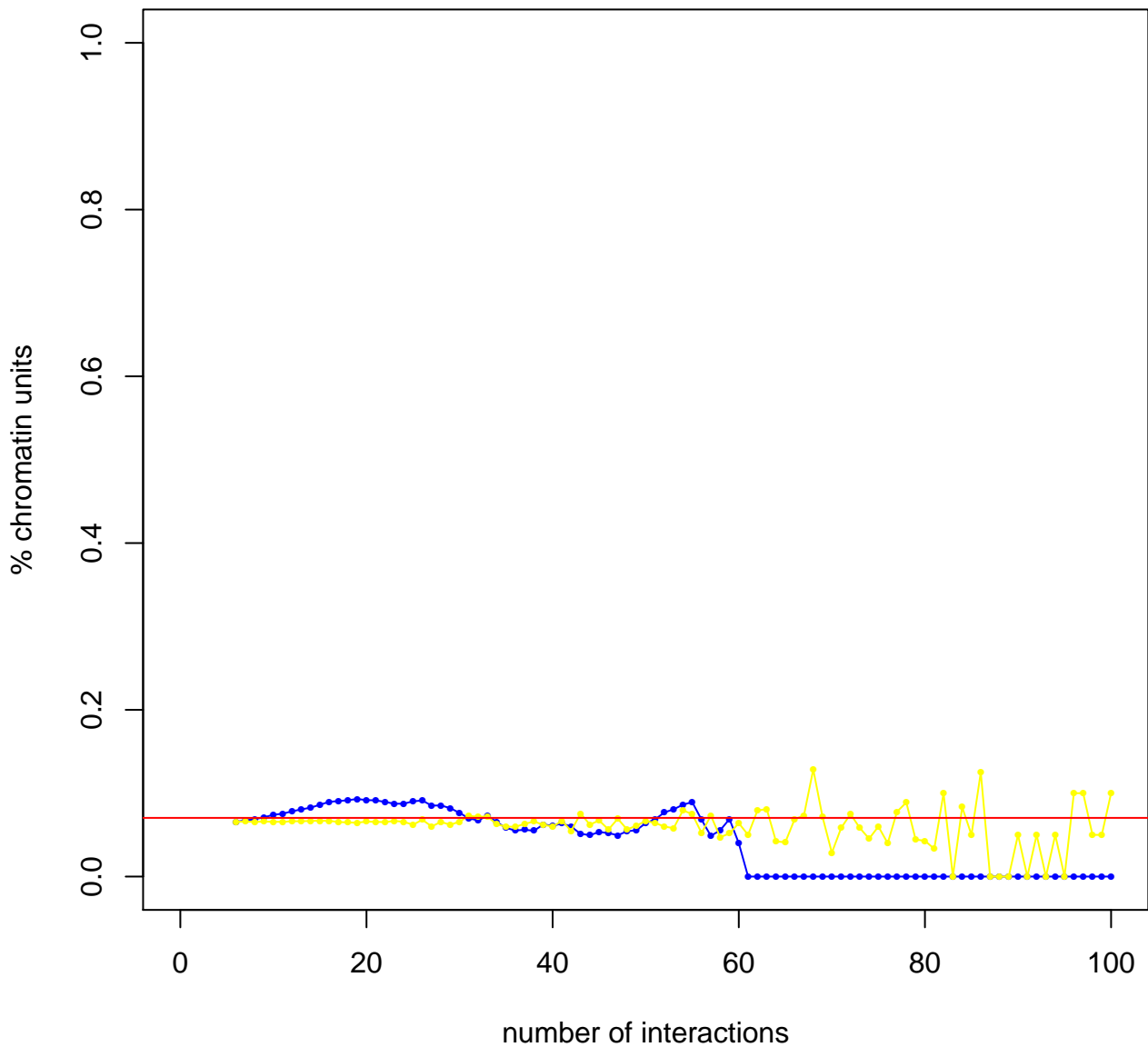

Supplement: Supplementary file 3 — A folder named SB-06-S3 contains 105 overlapping plot for each TF. (ZIP 624 kb) [file 12918_2018_643_MOESM3_ESM.zip › SB-06-S3/PHO4.pdf]

# PUT3

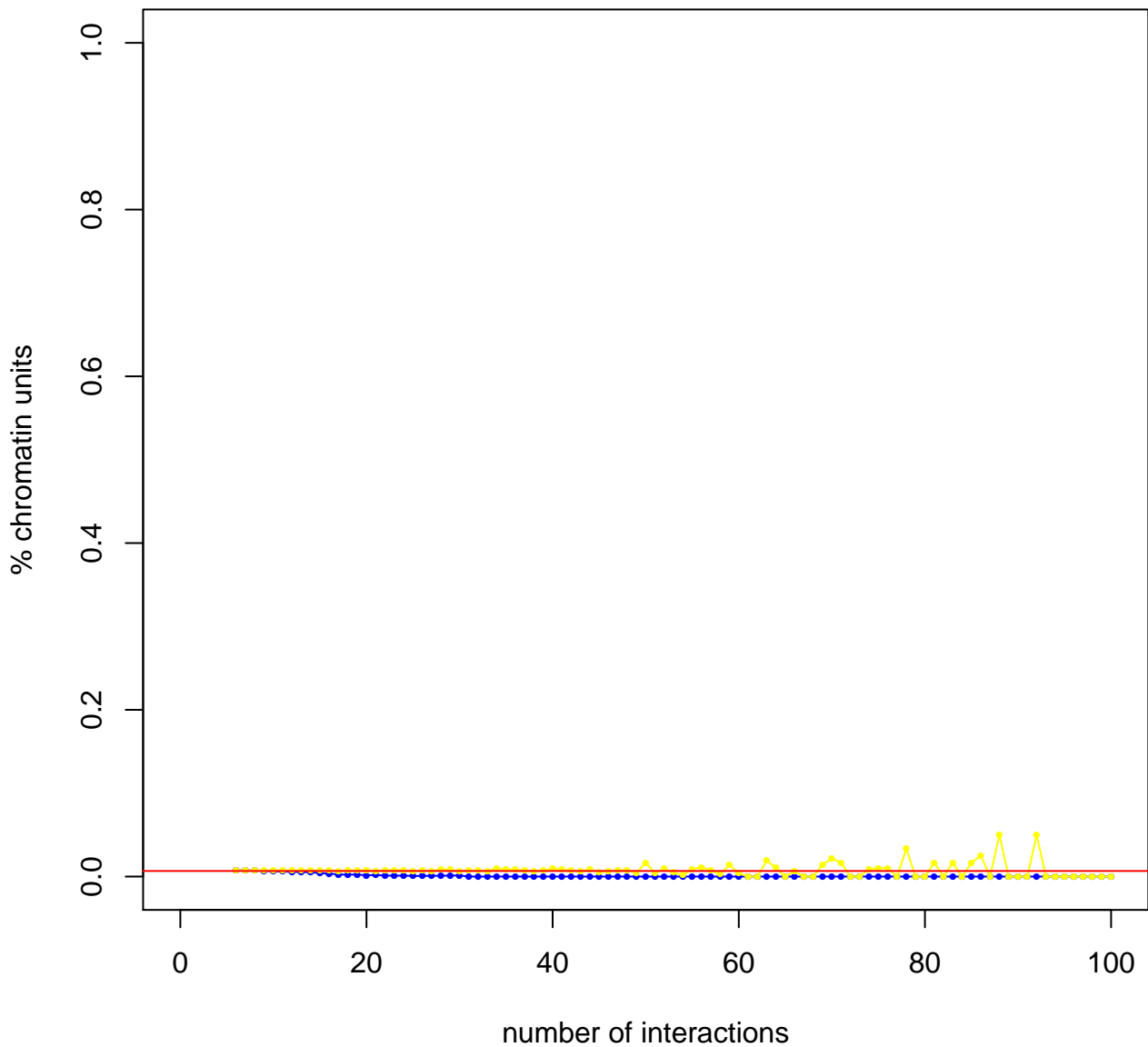

Supplement: Supplementary file 3 — A folder named SB-06-S3 contains 105 overlapping plot for each TF. (ZIP 624 kb) [file 12918_2018_643_MOESM3_ESM.zip › SB-06-S3/PUT3.pdf]

# RAP1

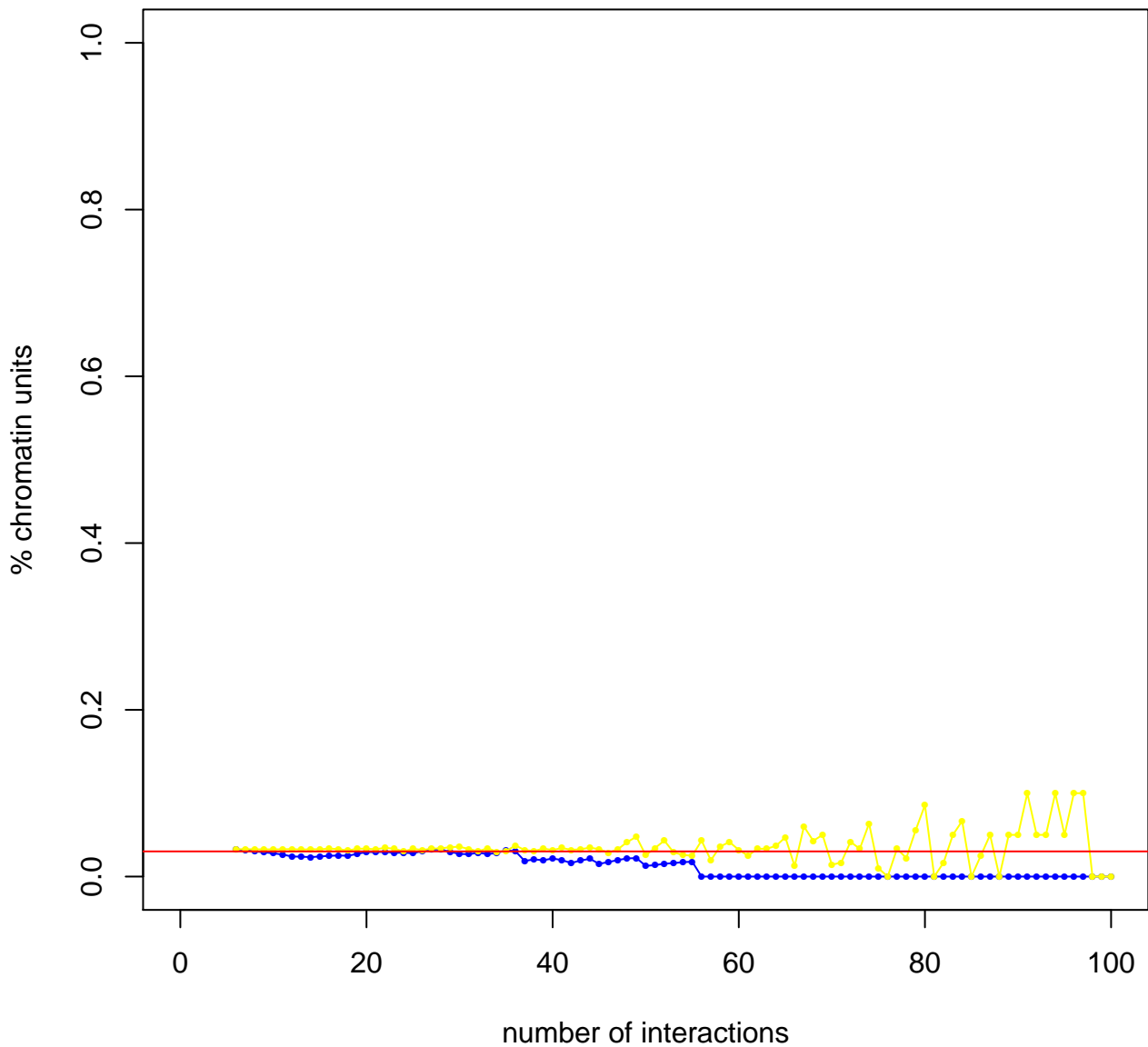

Supplement: Supplementary file 3 — A folder named SB-06-S3 contains 105 overlapping plot for each TF. (ZIP 624 kb) [file 12918_2018_643_MOESM3_ESM.zip › SB-06-S3/RAP1.pdf]

# RCS1

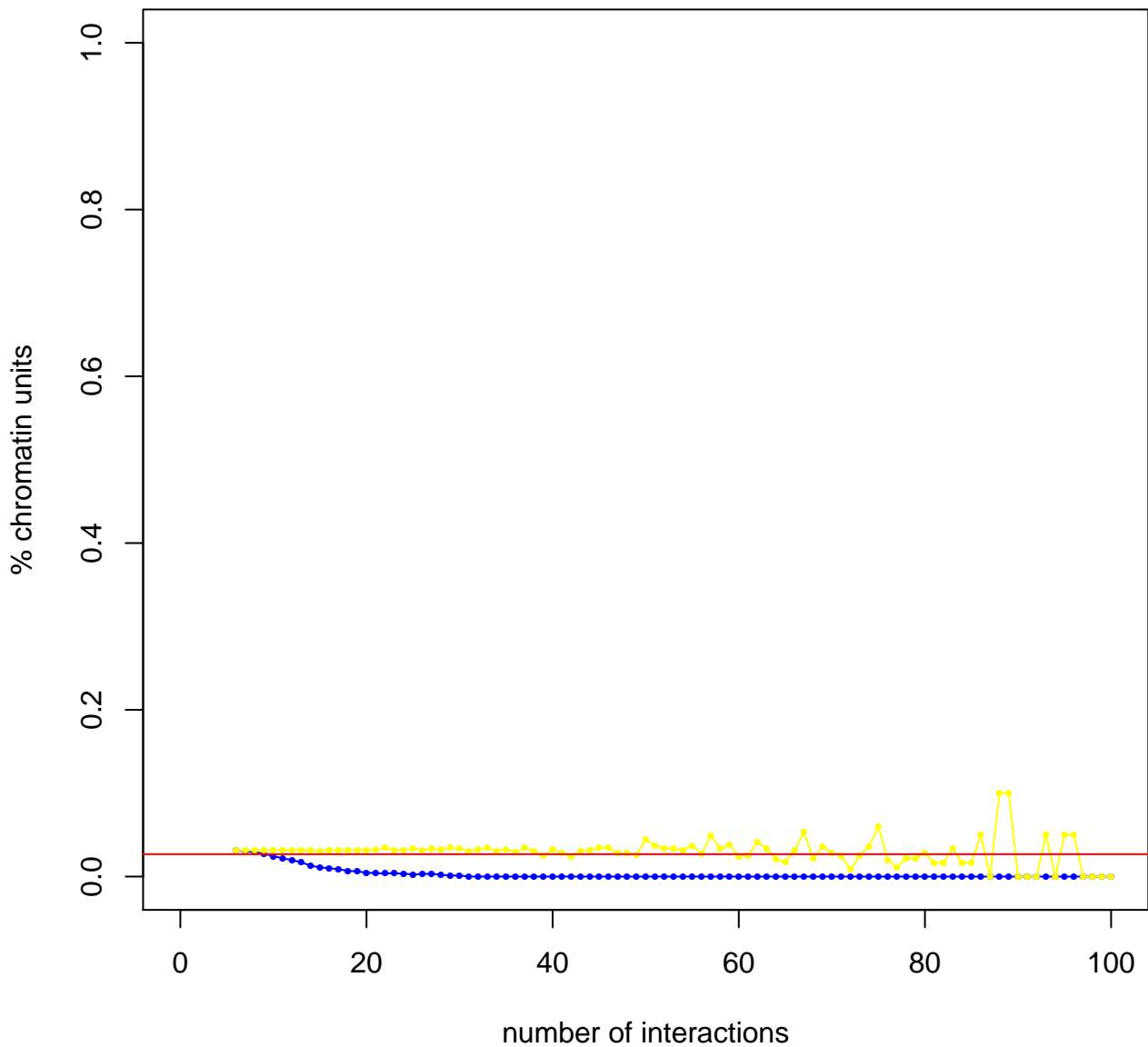

Supplement: Supplementary file 3 — A folder named SB-06-S3 contains 105 overlapping plot for each TF. (ZIP 624 kb) [file 12918_2018_643_MOESM3_ESM.zip › SB-06-S3/RCS1.pdf]

# RDS1

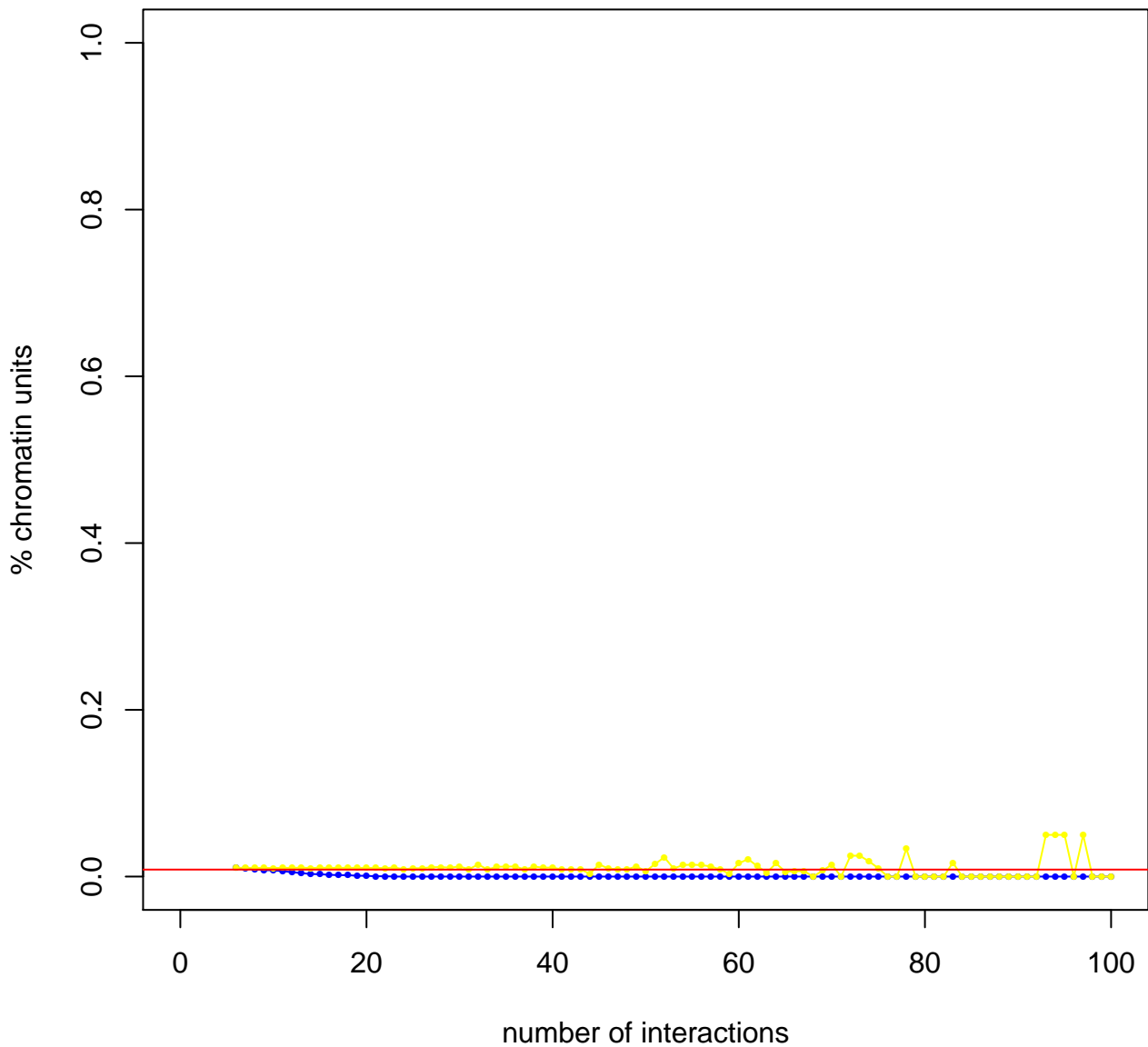

Supplement: Supplementary file 3 — A folder named SB-06-S3 contains 105 overlapping plot for each TF. (ZIP 624 kb) [file 12918_2018_643_MOESM3_ESM.zip › SB-06-S3/RDS1.pdf]

# REB1

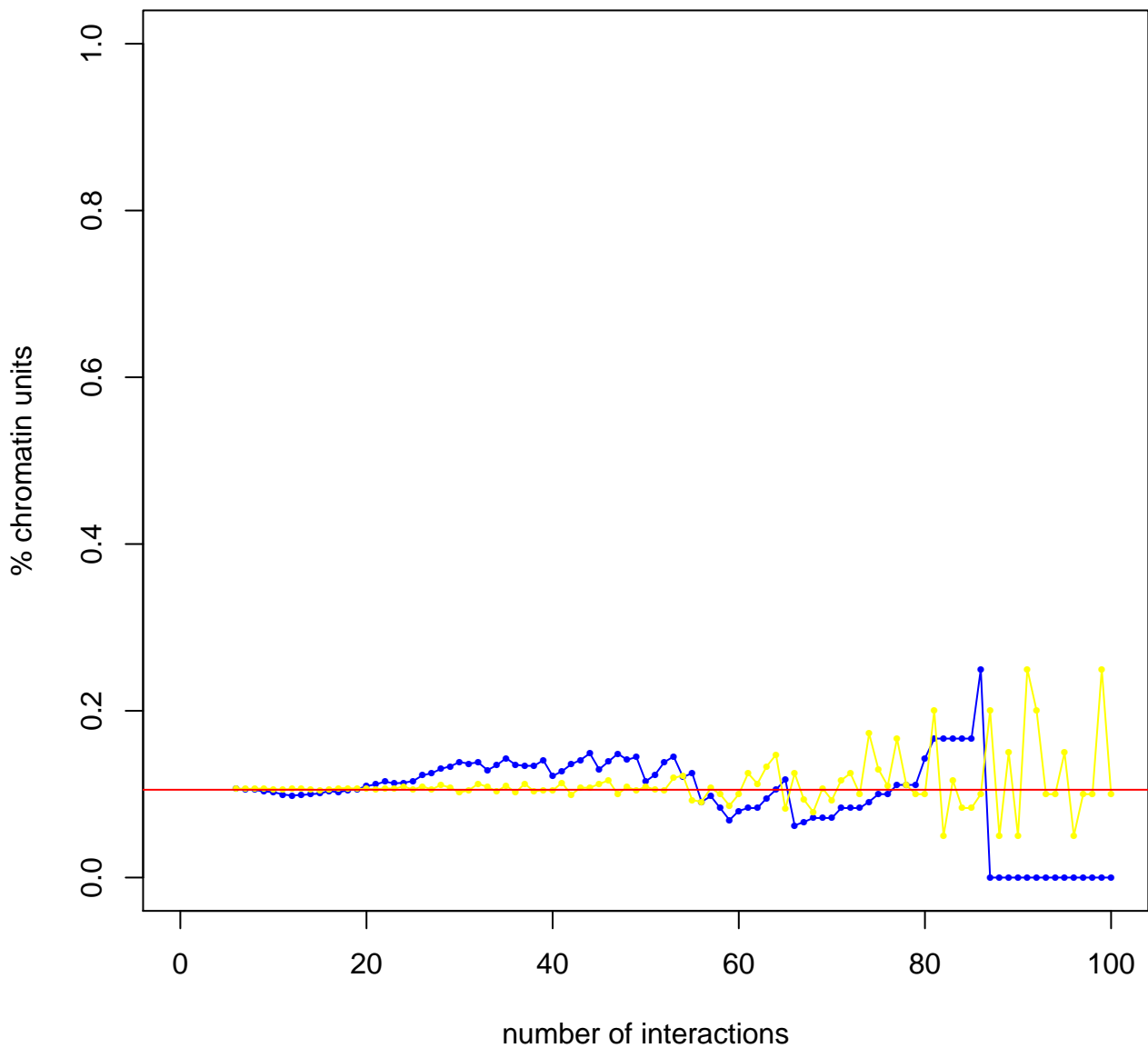

Supplement: Supplementary file 3 — A folder named SB-06-S3 contains 105 overlapping plot for each TF. (ZIP 624 kb) [file 12918_2018_643_MOESM3_ESM.zip › SB-06-S3/REB1.pdf]

# RFX1

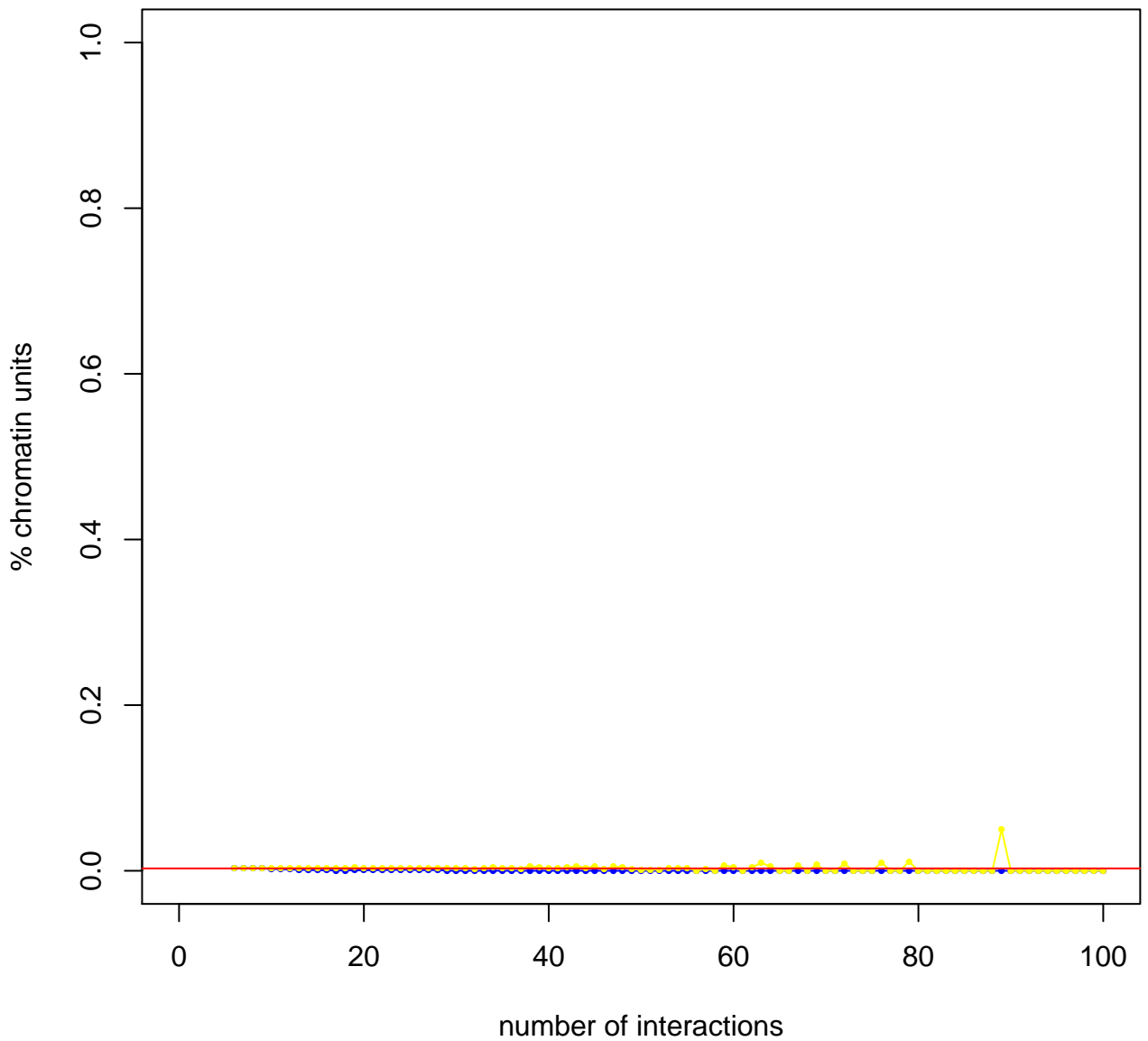

Supplement: Supplementary file 3 — A folder named SB-06-S3 contains 105 overlapping plot for each TF. (ZIP 624 kb) [file 12918_2018_643_MOESM3_ESM.zip › SB-06-S3/RFX1.pdf]

# RGT1

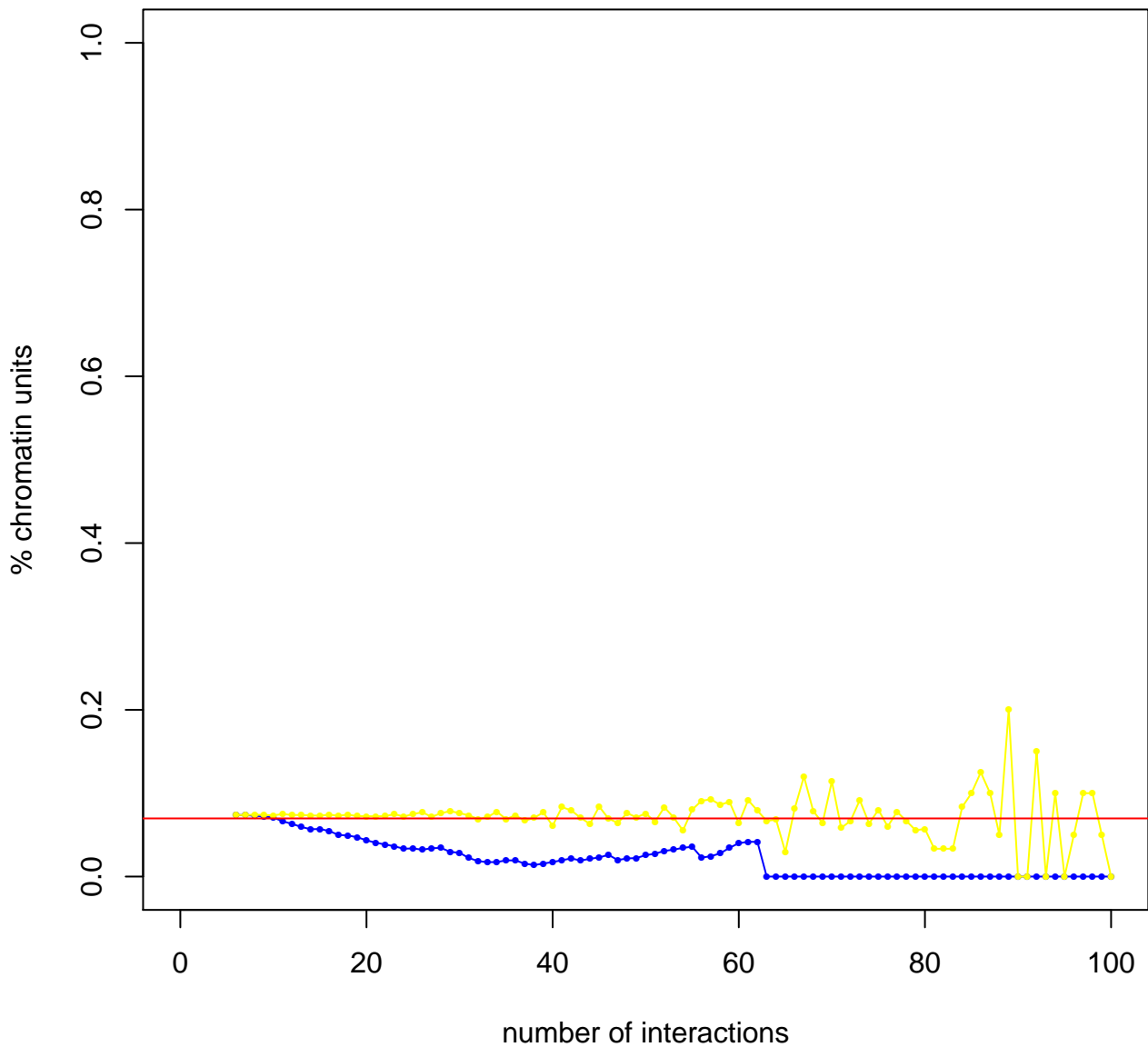

Supplement: Supplementary file 3 — A folder named SB-06-S3 contains 105 overlapping plot for each TF. (ZIP 624 kb) [file 12918_2018_643_MOESM3_ESM.zip › SB-06-S3/RGT1.pdf]

# RIM101

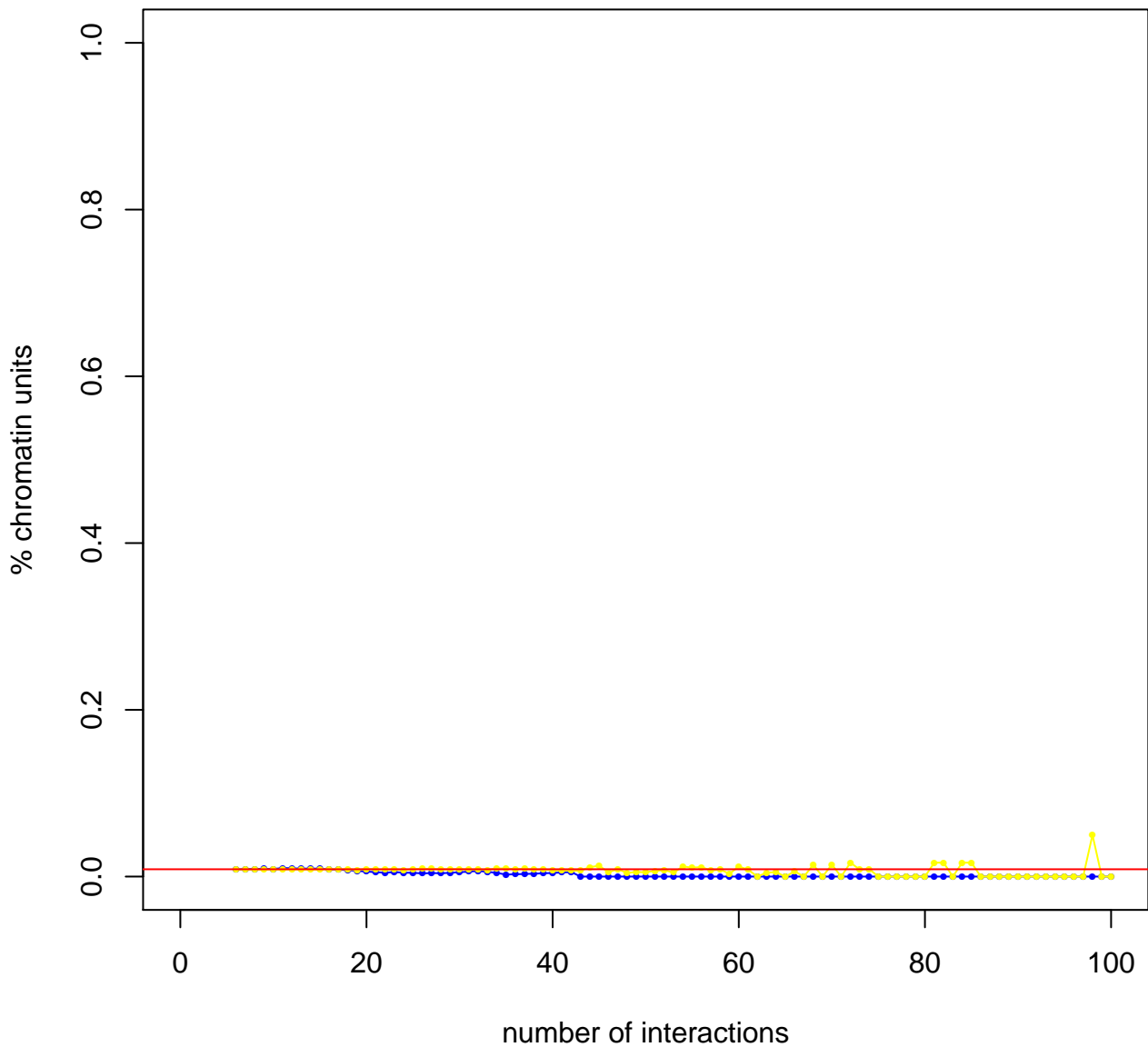

Supplement: Supplementary file 3 — A folder named SB-06-S3 contains 105 overlapping plot for each TF. (ZIP 624 kb) [file 12918_2018_643_MOESM3_ESM.zip › SB-06-S3/RIM101.pdf]

# RLM1

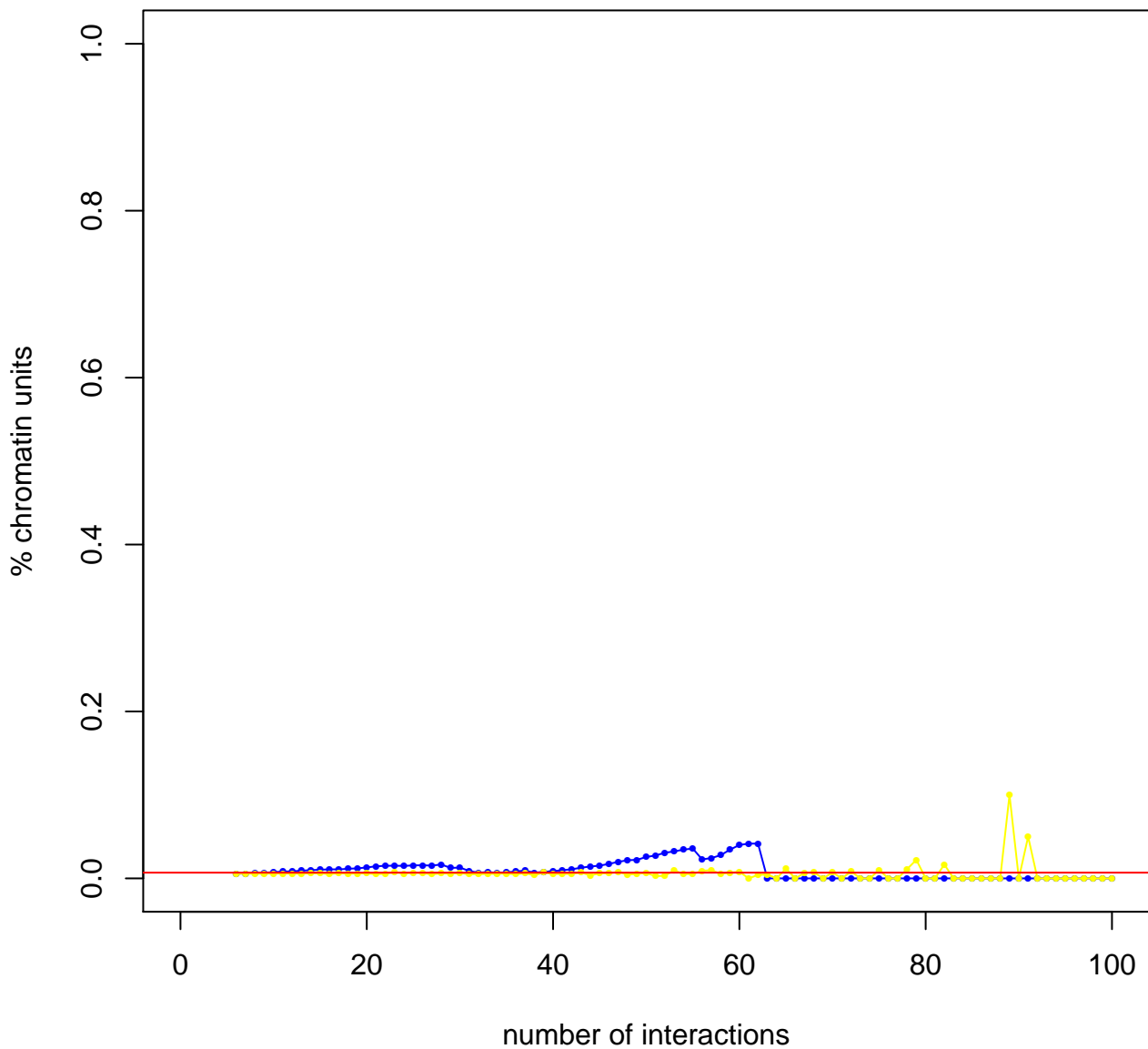

Supplement: Supplementary file 3 — A folder named SB-06-S3 contains 105 overlapping plot for each TF. (ZIP 624 kb) [file 12918_2018_643_MOESM3_ESM.zip › SB-06-S3/RLM1.pdf]

# RLR1

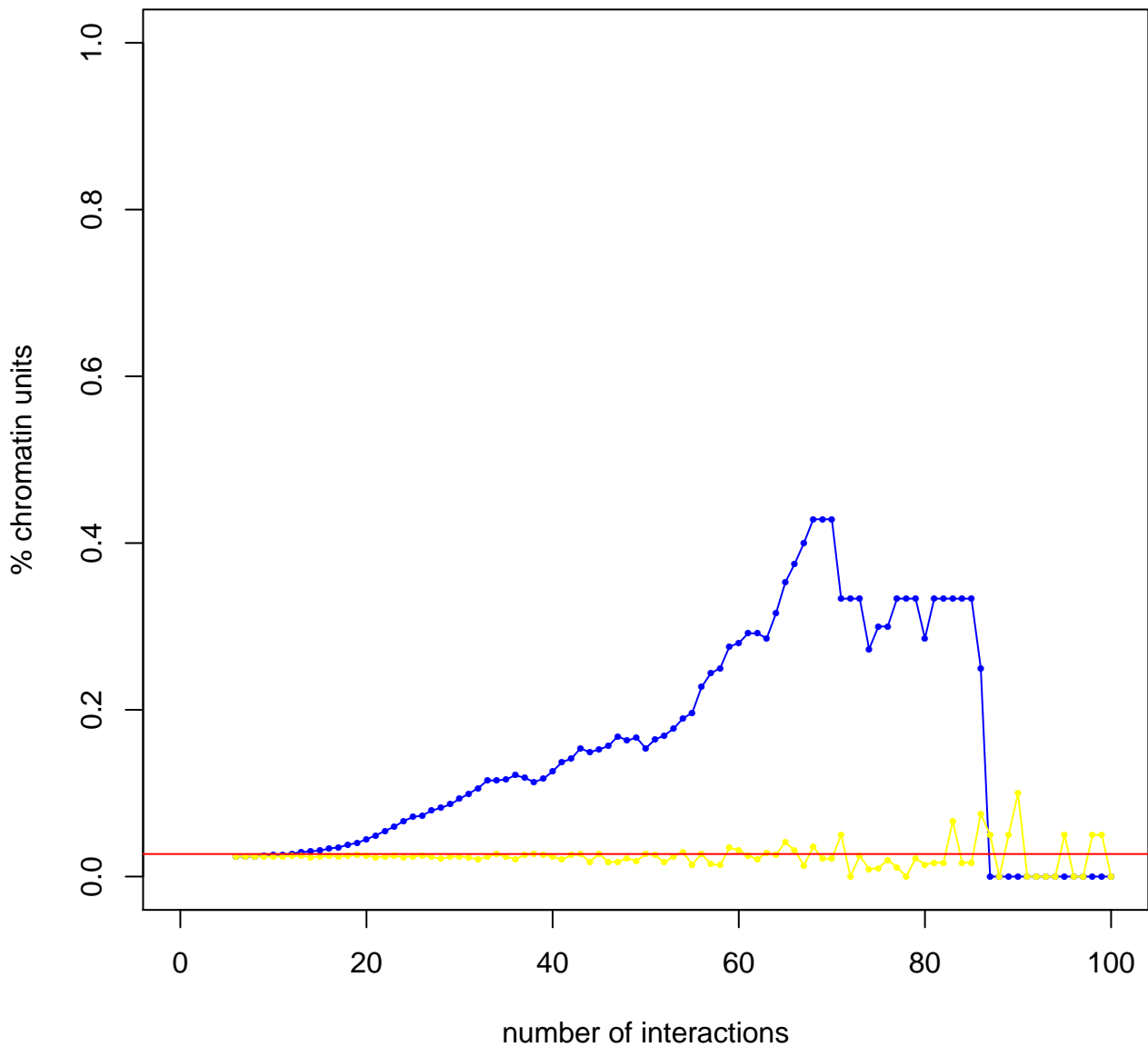

Supplement: Supplementary file 3 — A folder named SB-06-S3 contains 105 overlapping plot for each TF. (ZIP 624 kb) [file 12918_2018_643_MOESM3_ESM.zip › SB-06-S3/RLR1.pdf]

# ROX1

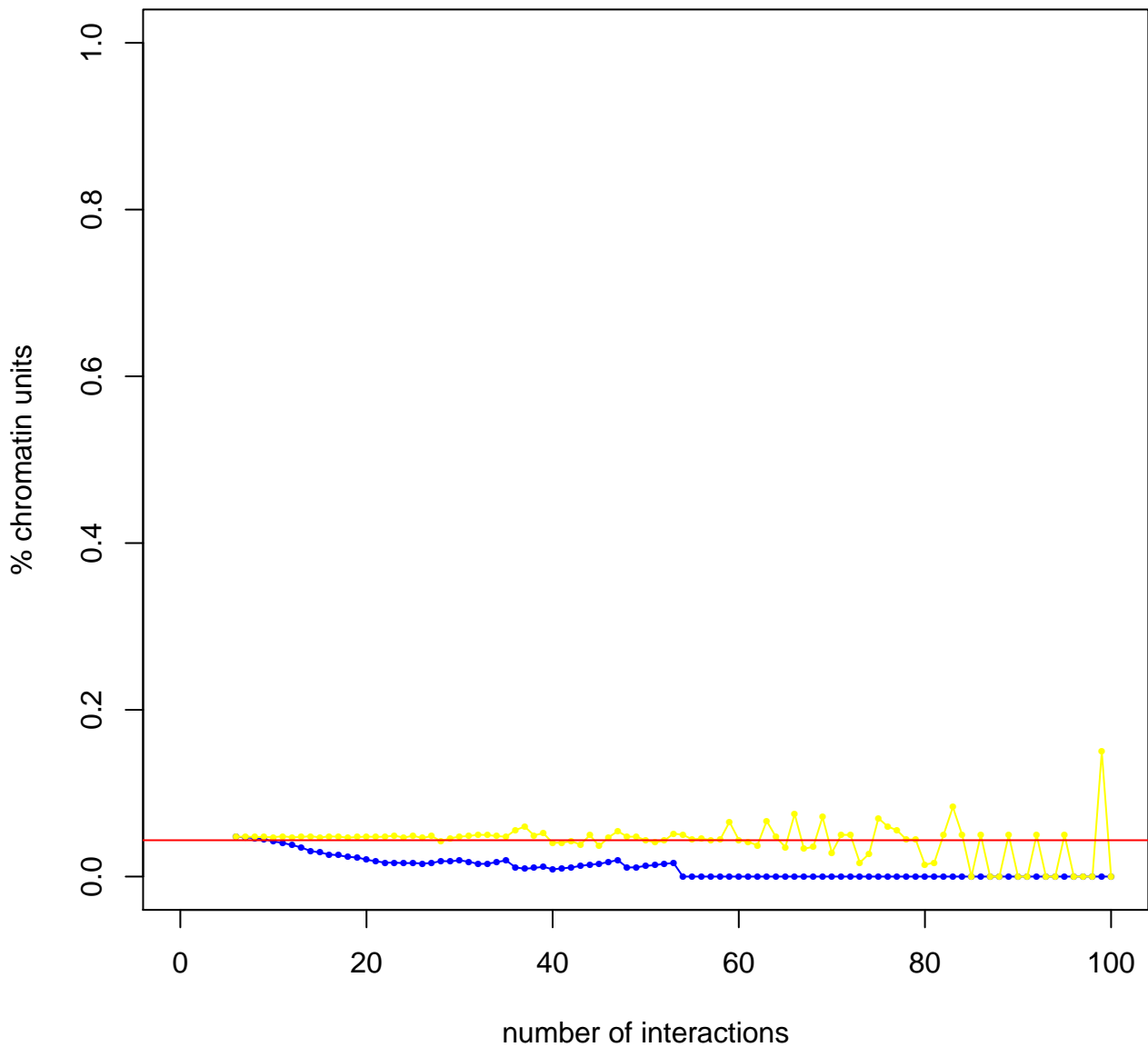

Supplement: Supplementary file 3 — A folder named SB-06-S3 contains 105 overlapping plot for each TF. (ZIP 624 kb) [file 12918_2018_643_MOESM3_ESM.zip › SB-06-S3/ROX1.pdf]

# RPH1

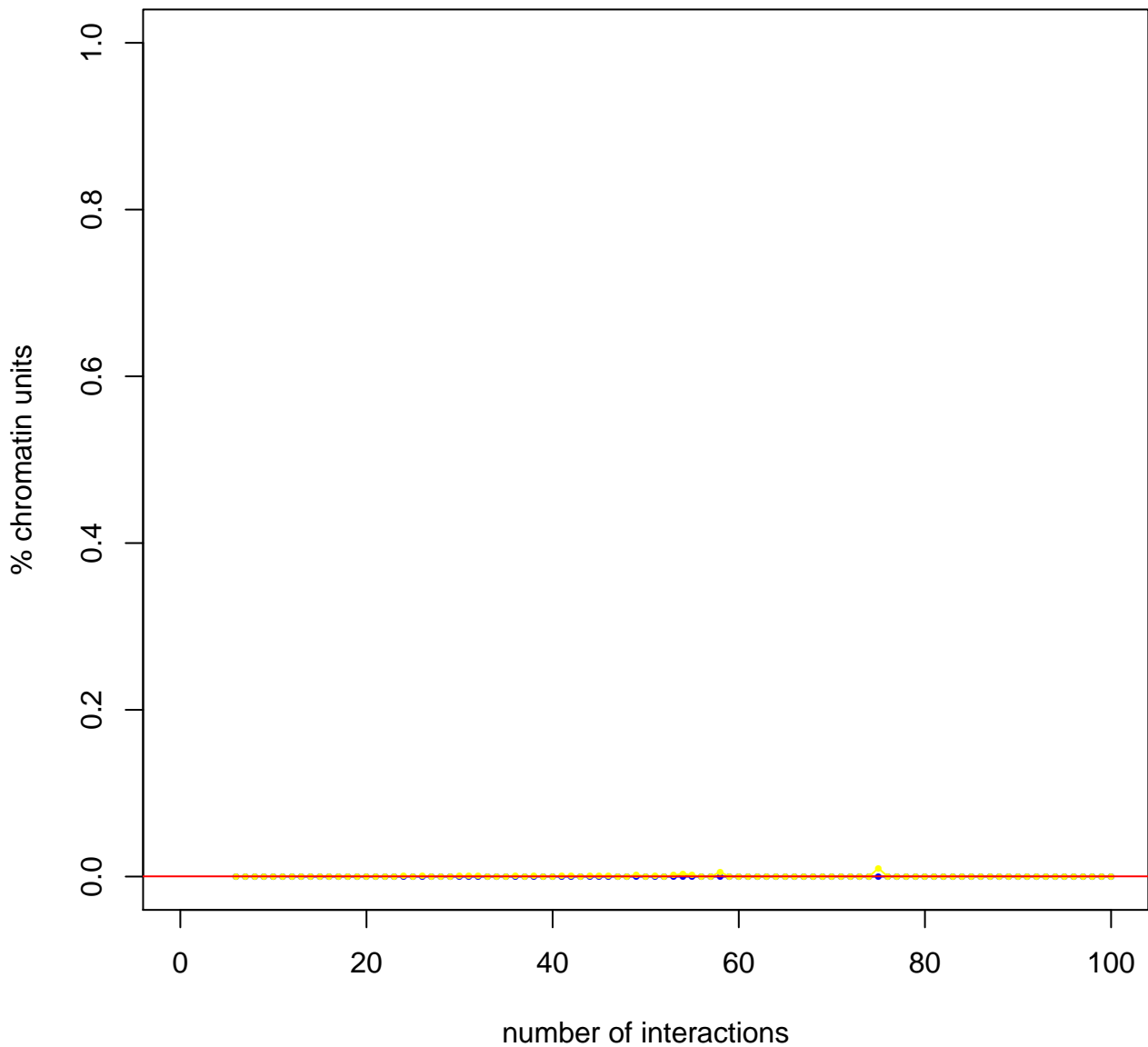

Supplement: Supplementary file 3 — A folder named SB-06-S3 contains 105 overlapping plot for each TF. (ZIP 624 kb) [file 12918_2018_643_MOESM3_ESM.zip › SB-06-S3/RPH1.pdf]

# RPN4

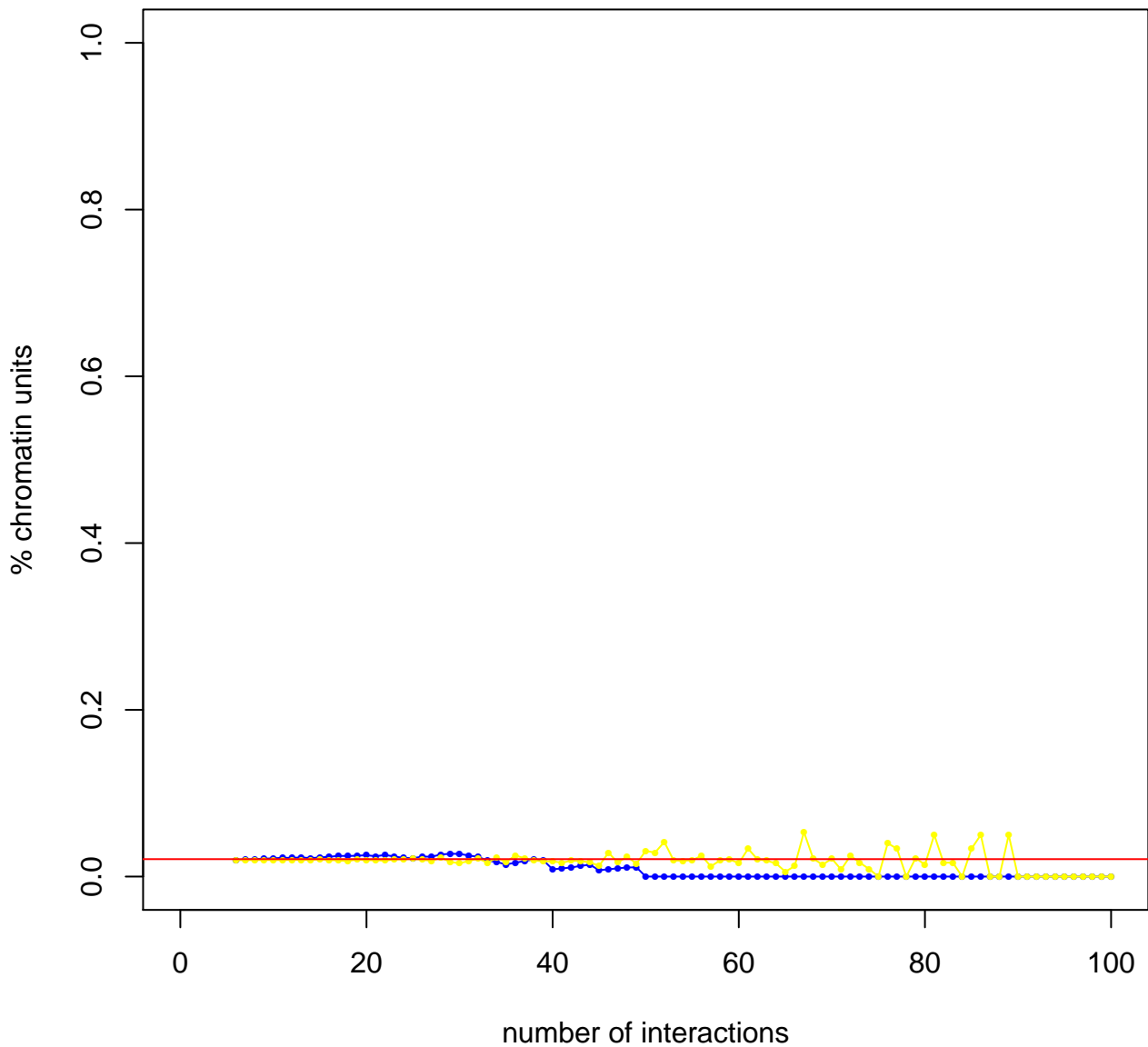

Supplement: Supplementary file 3 — A folder named SB-06-S3 contains 105 overlapping plot for each TF. (ZIP 624 kb) [file 12918_2018_643_MOESM3_ESM.zip › SB-06-S3/RPN4.pdf]

# RTG3

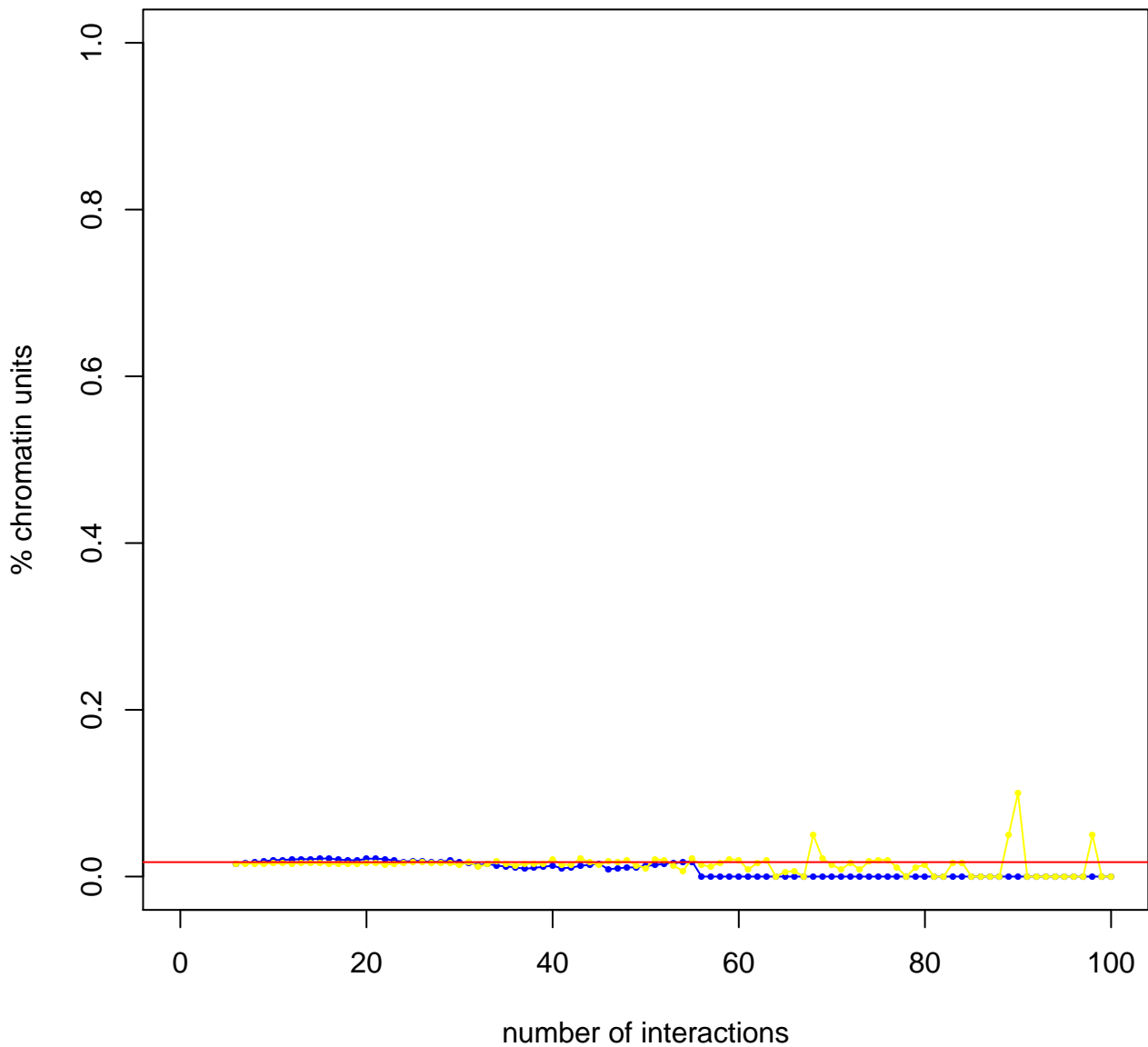

Supplement: Supplementary file 3 — A folder named SB-06-S3 contains 105 overlapping plot for each TF. (ZIP 624 kb) [file 12918_2018_643_MOESM3_ESM.zip › SB-06-S3/RTG3.pdf]

# SFL1

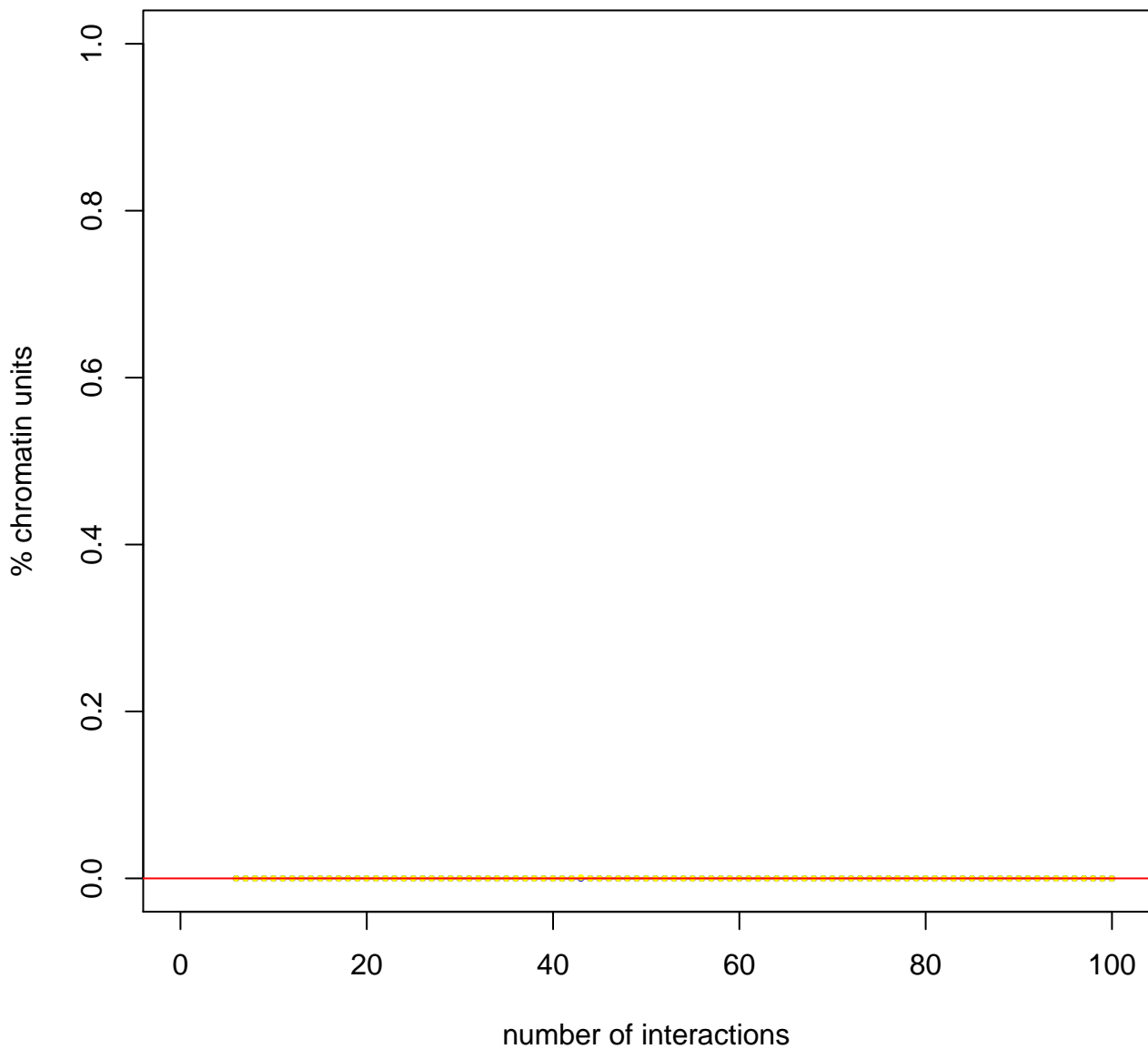

Supplement: Supplementary file 3 — A folder named SB-06-S3 contains 105 overlapping plot for each TF. (ZIP 624 kb) [file 12918_2018_643_MOESM3_ESM.zip › SB-06-S3/SFL1.pdf]

# SFP1

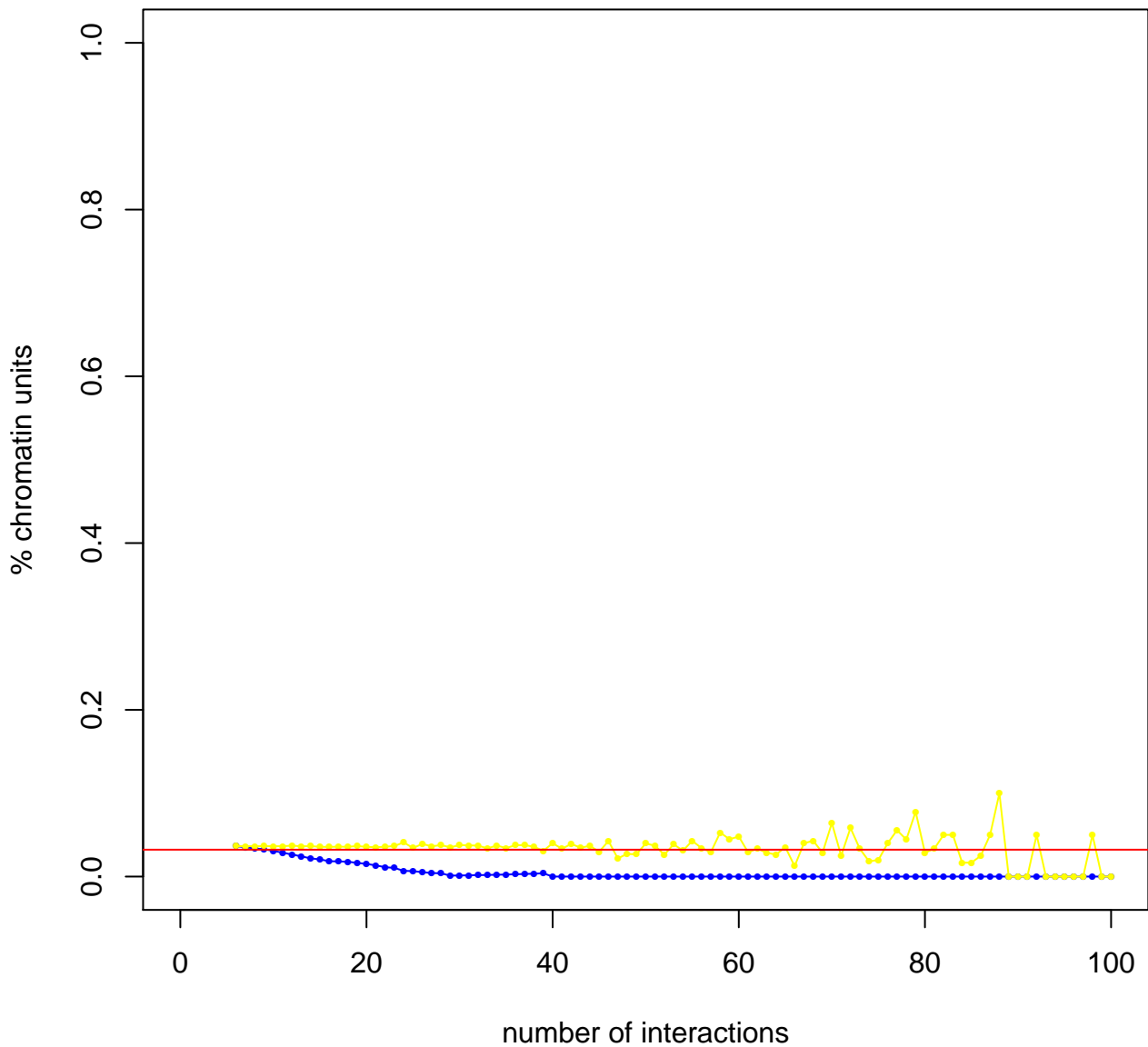

Supplement: Supplementary file 3 — A folder named SB-06-S3 contains 105 overlapping plot for each TF. (ZIP 624 kb) [file 12918_2018_643_MOESM3_ESM.zip › SB-06-S3/SFP1.pdf]

# SIG1

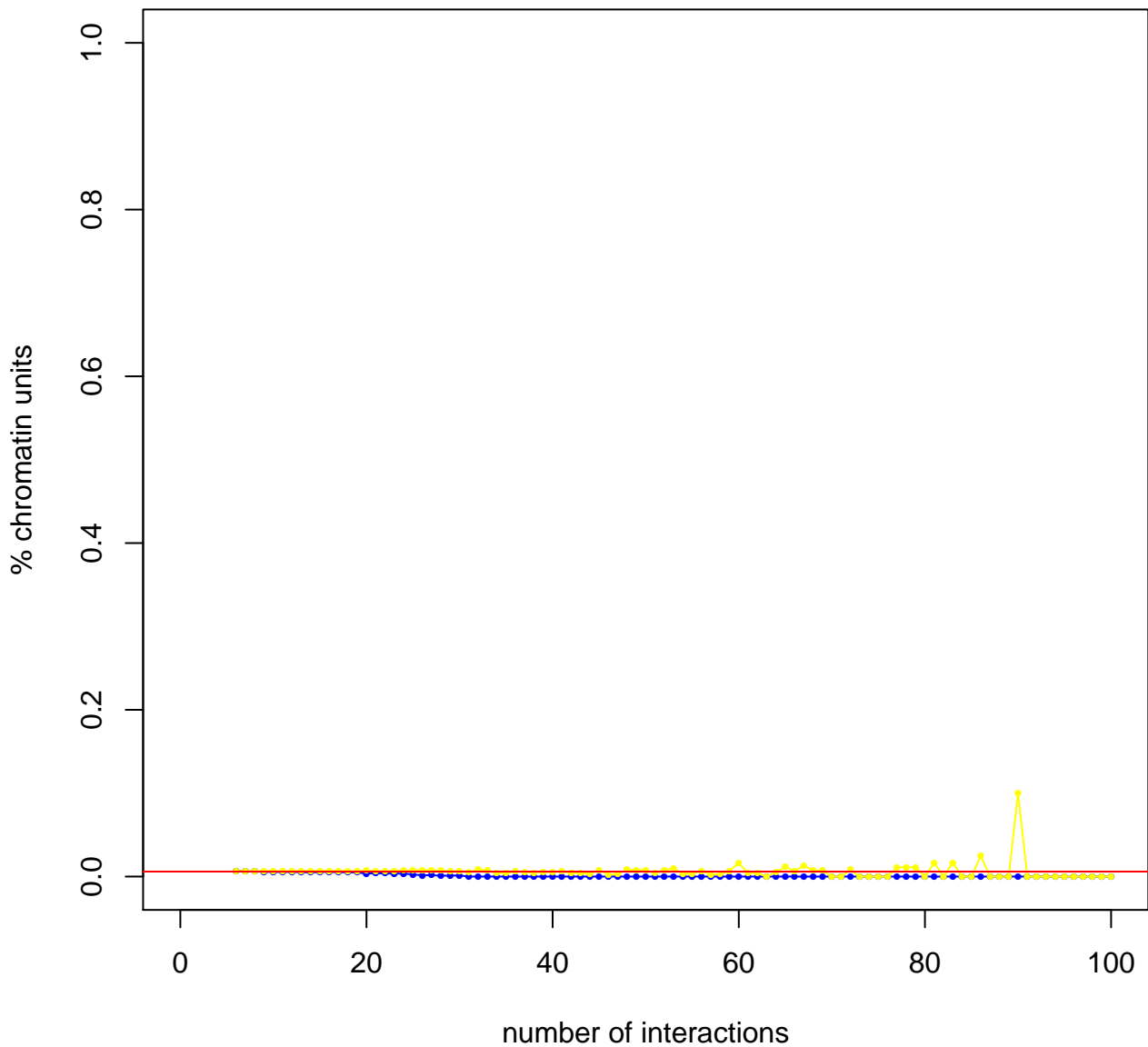

Supplement: Supplementary file 3 — A folder named SB-06-S3 contains 105 overlapping plot for each TF. (ZIP 624 kb) [file 12918_2018_643_MOESM3_ESM.zip › SB-06-S3/SIG1.pdf]

# SIP4

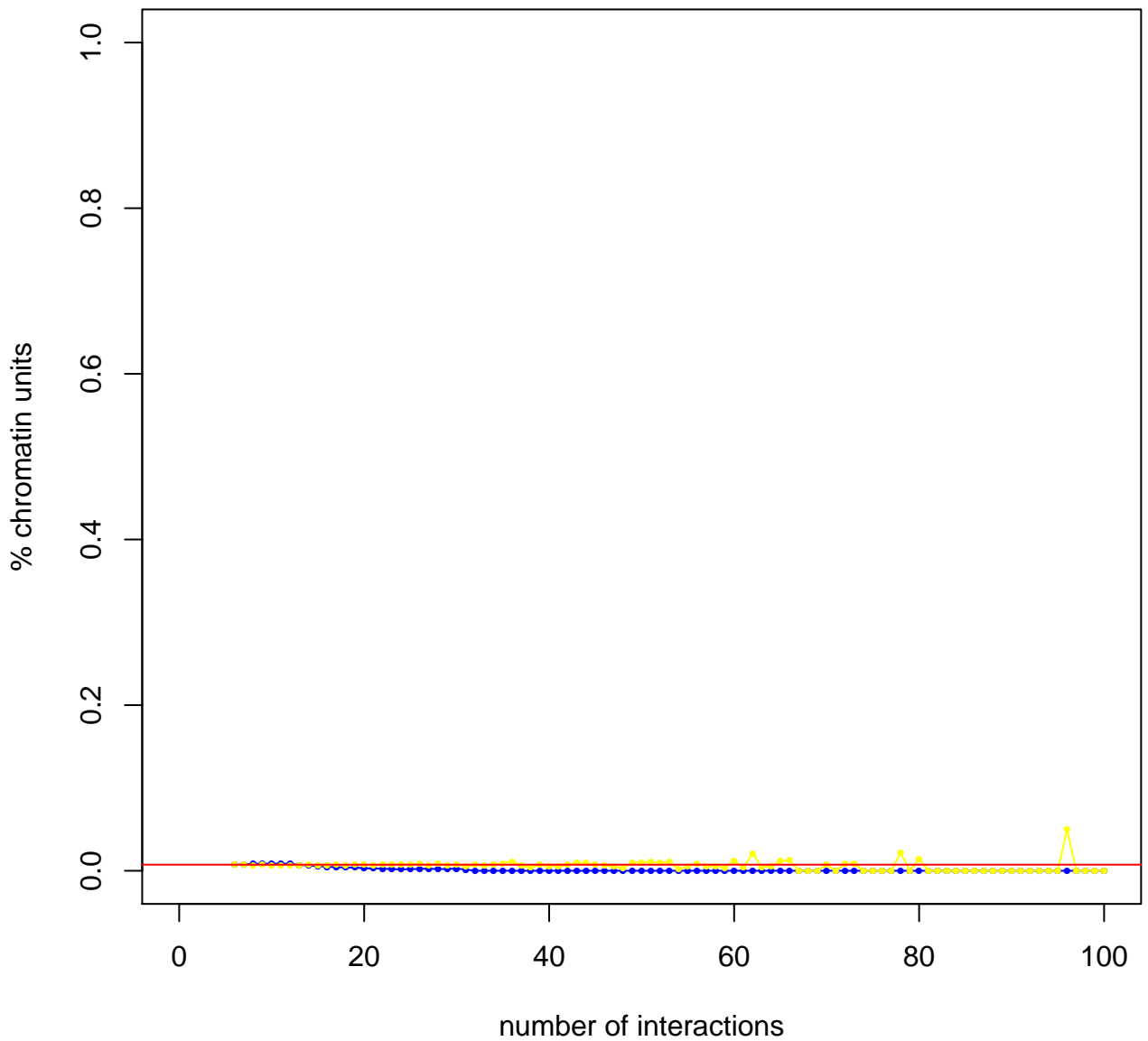

Supplement: Supplementary file 3 — A folder named SB-06-S3 contains 105 overlapping plot for each TF. (ZIP 624 kb) [file 12918_2018_643_MOESM3_ESM.zip › SB-06-S3/SIP4.pdf]

# SKN7

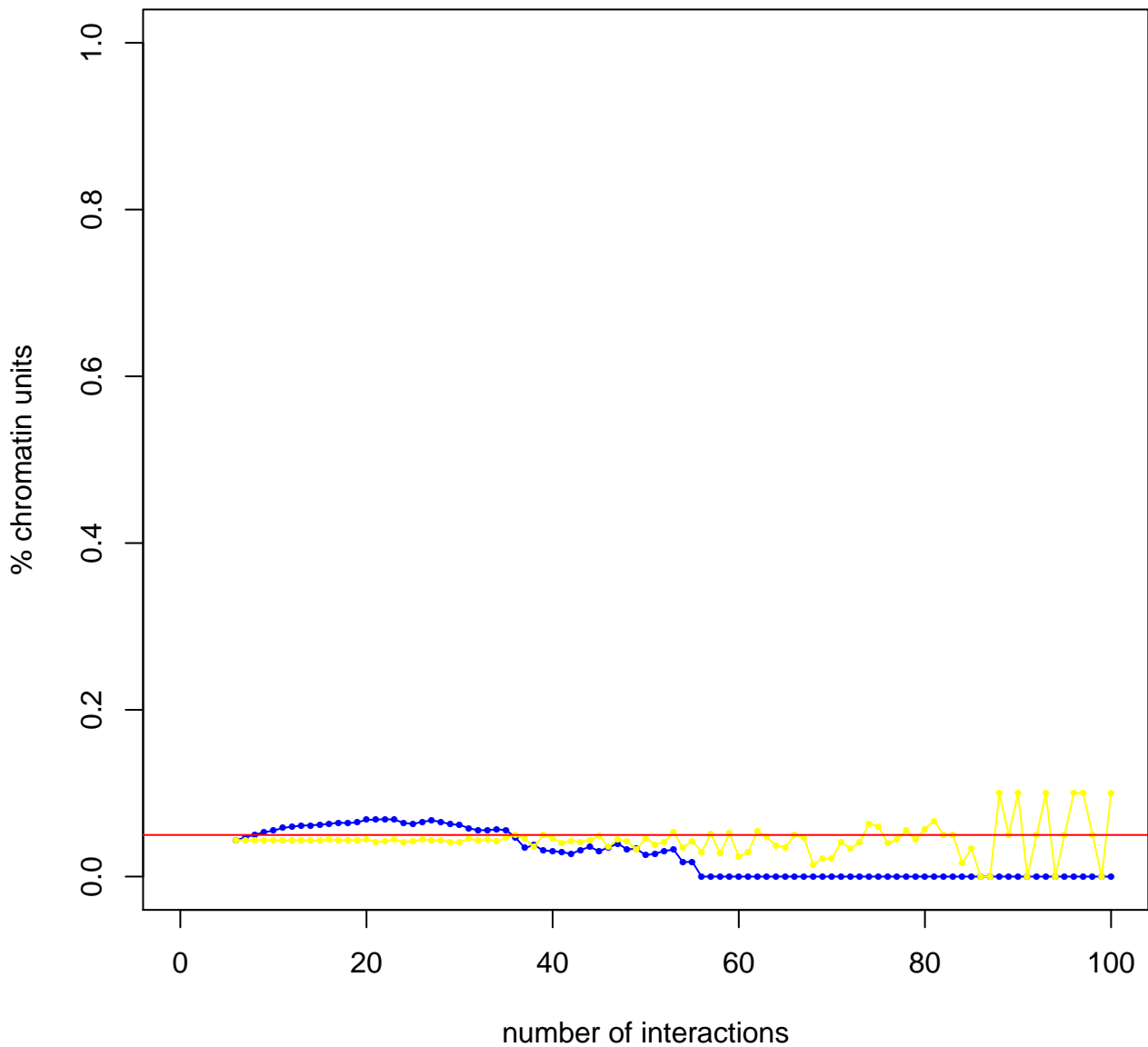

Supplement: Supplementary file 3 — A folder named SB-06-S3 contains 105 overlapping plot for each TF. (ZIP 624 kb) [file 12918_2018_643_MOESM3_ESM.zip › SB-06-S3/SKN7.pdf]

# SKO1

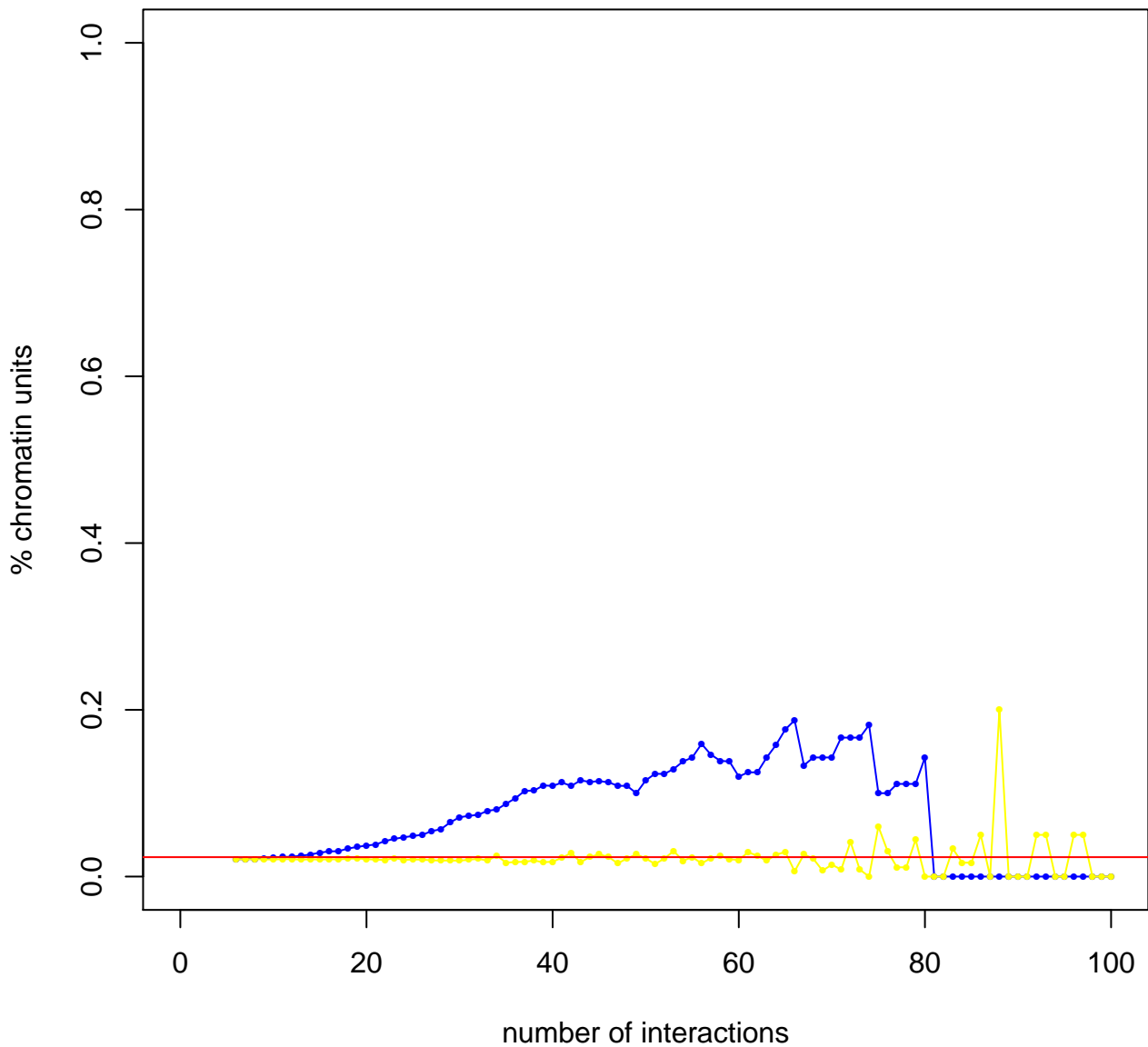

Supplement: Supplementary file 3 — A folder named SB-06-S3 contains 105 overlapping plot for each TF. (ZIP 624 kb) [file 12918_2018_643_MOESM3_ESM.zip › SB-06-S3/SKO1.pdf]

# SMP1

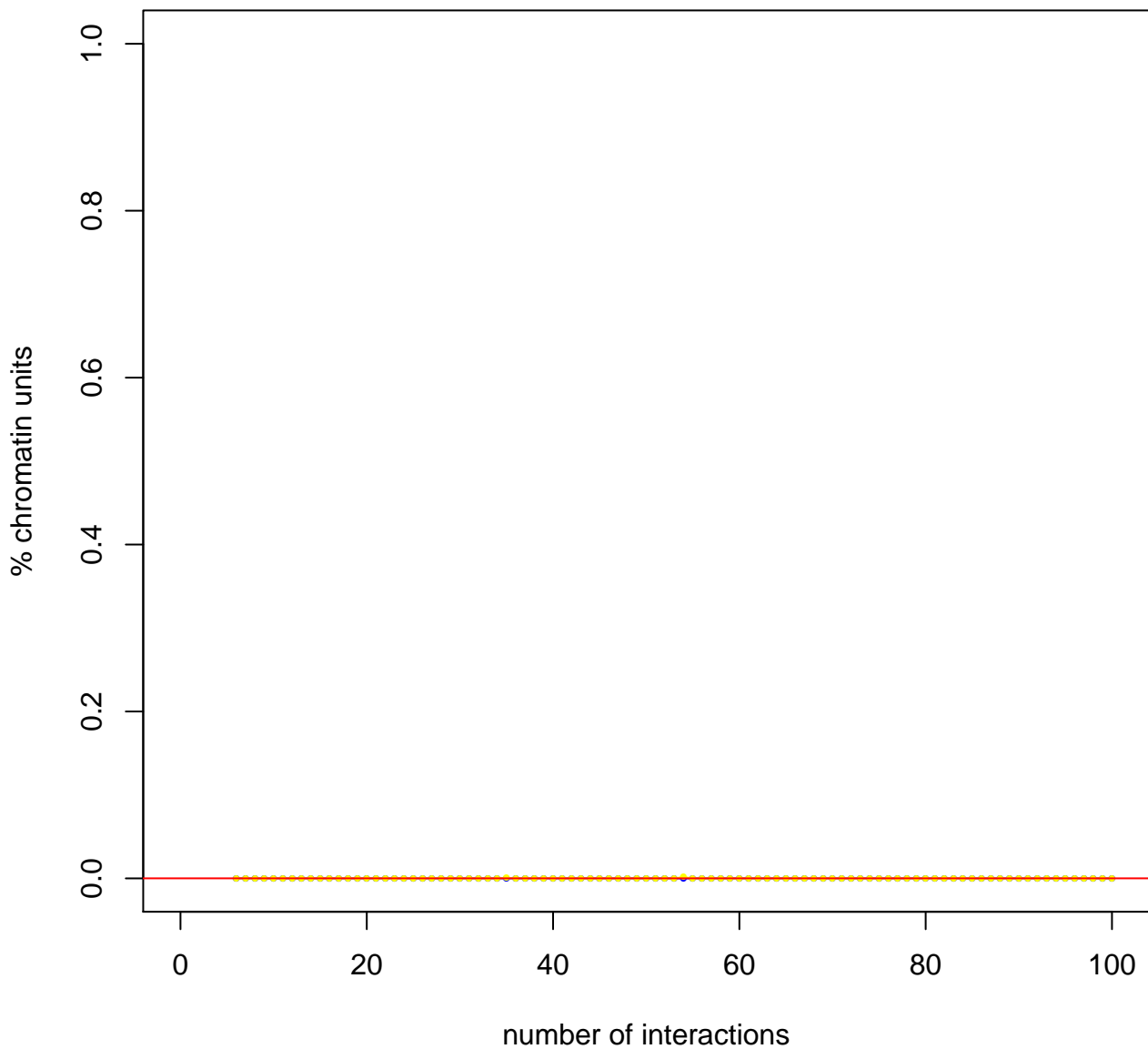

Supplement: Supplementary file 3 — A folder named SB-06-S3 contains 105 overlapping plot for each TF. (ZIP 624 kb) [file 12918_2018_643_MOESM3_ESM.zip › SB-06-S3/SMP1.pdf]

# SNT2

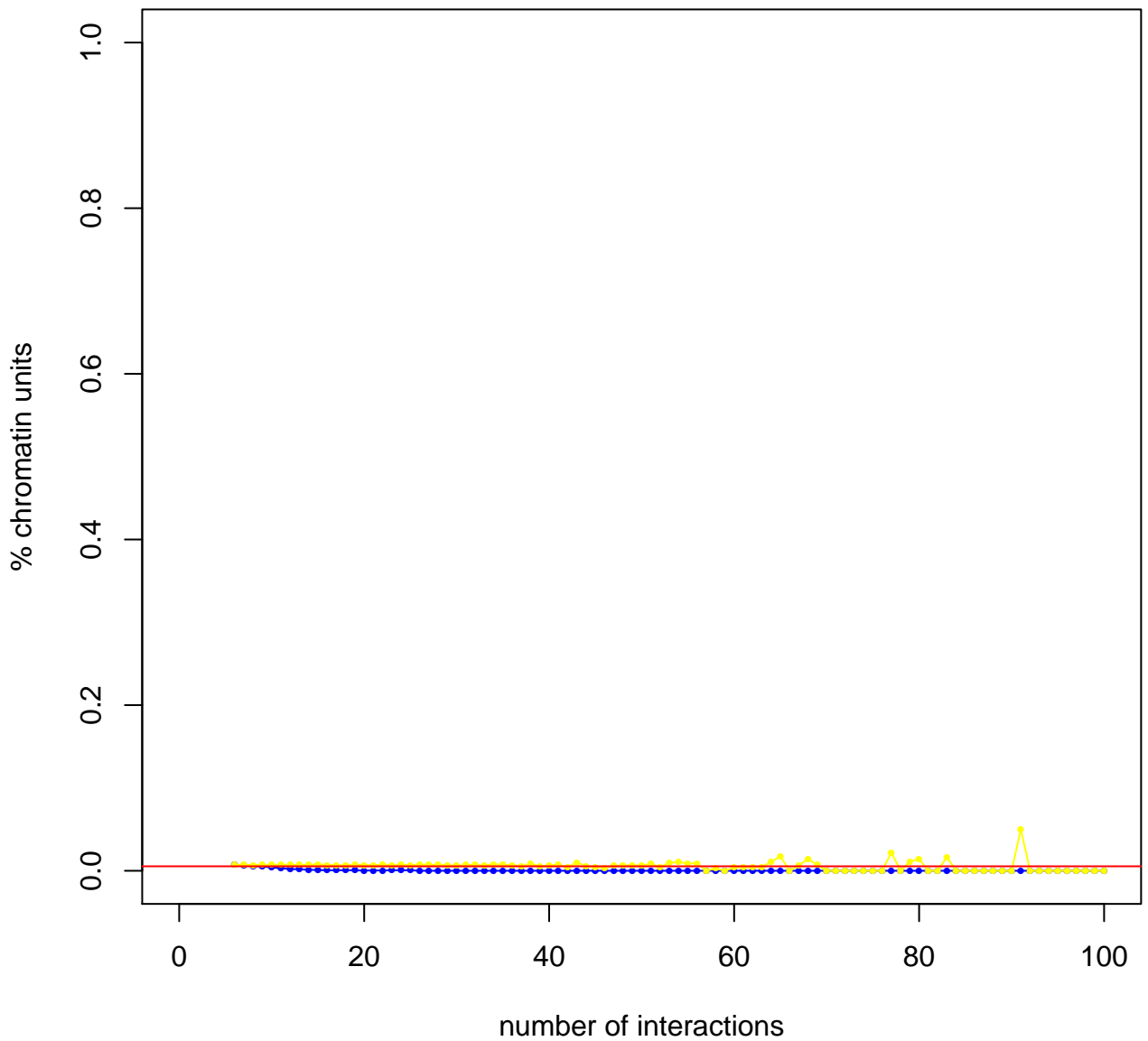

Supplement: Supplementary file 3 — A folder named SB-06-S3 contains 105 overlapping plot for each TF. (ZIP 624 kb) [file 12918_2018_643_MOESM3_ESM.zip › SB-06-S3/SNT2.pdf]

# SOK2

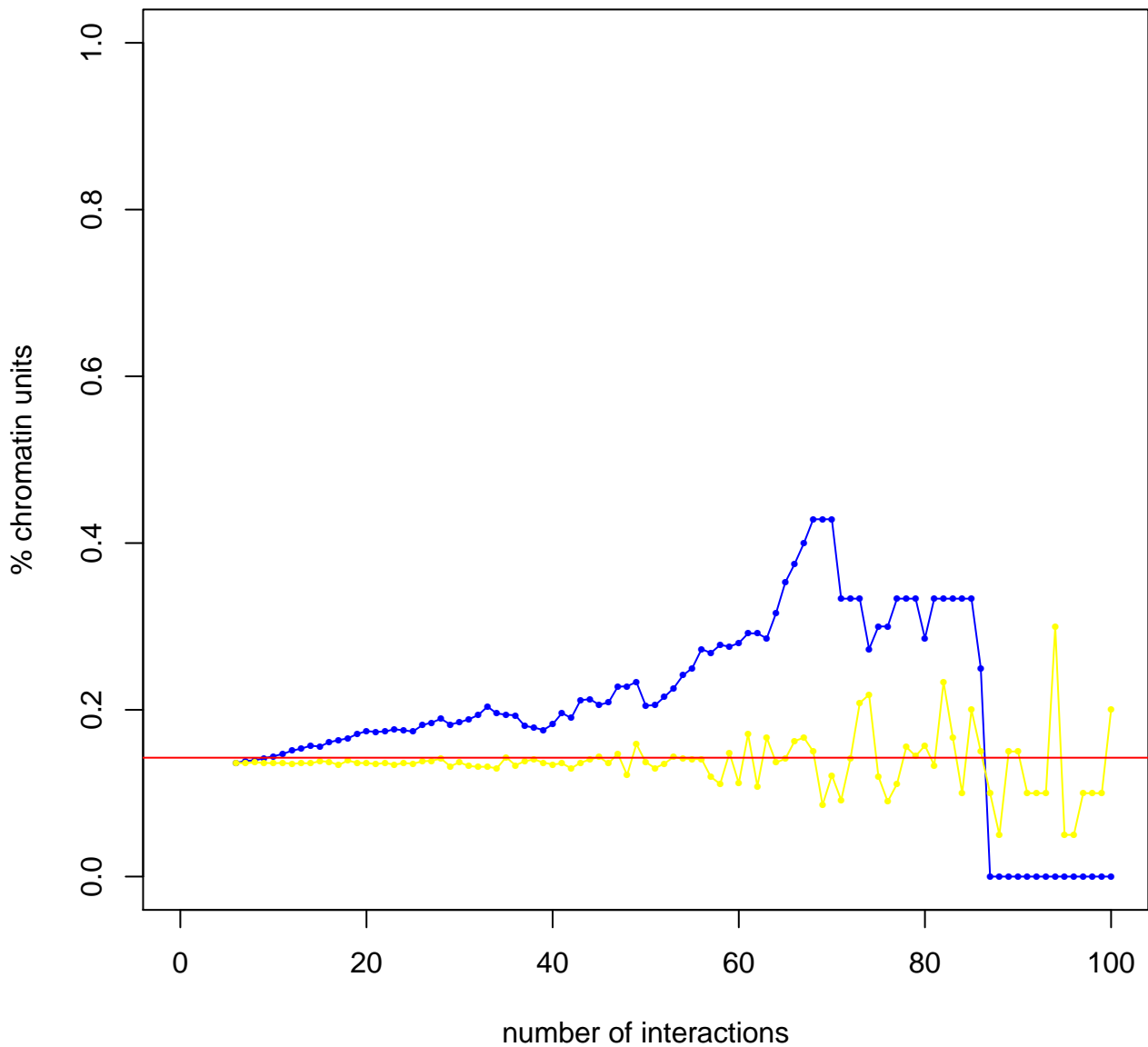

Supplement: Supplementary file 3 — A folder named SB-06-S3 contains 105 overlapping plot for each TF. (ZIP 624 kb) [file 12918_2018_643_MOESM3_ESM.zip › SB-06-S3/SOK2.pdf]

## SPT2

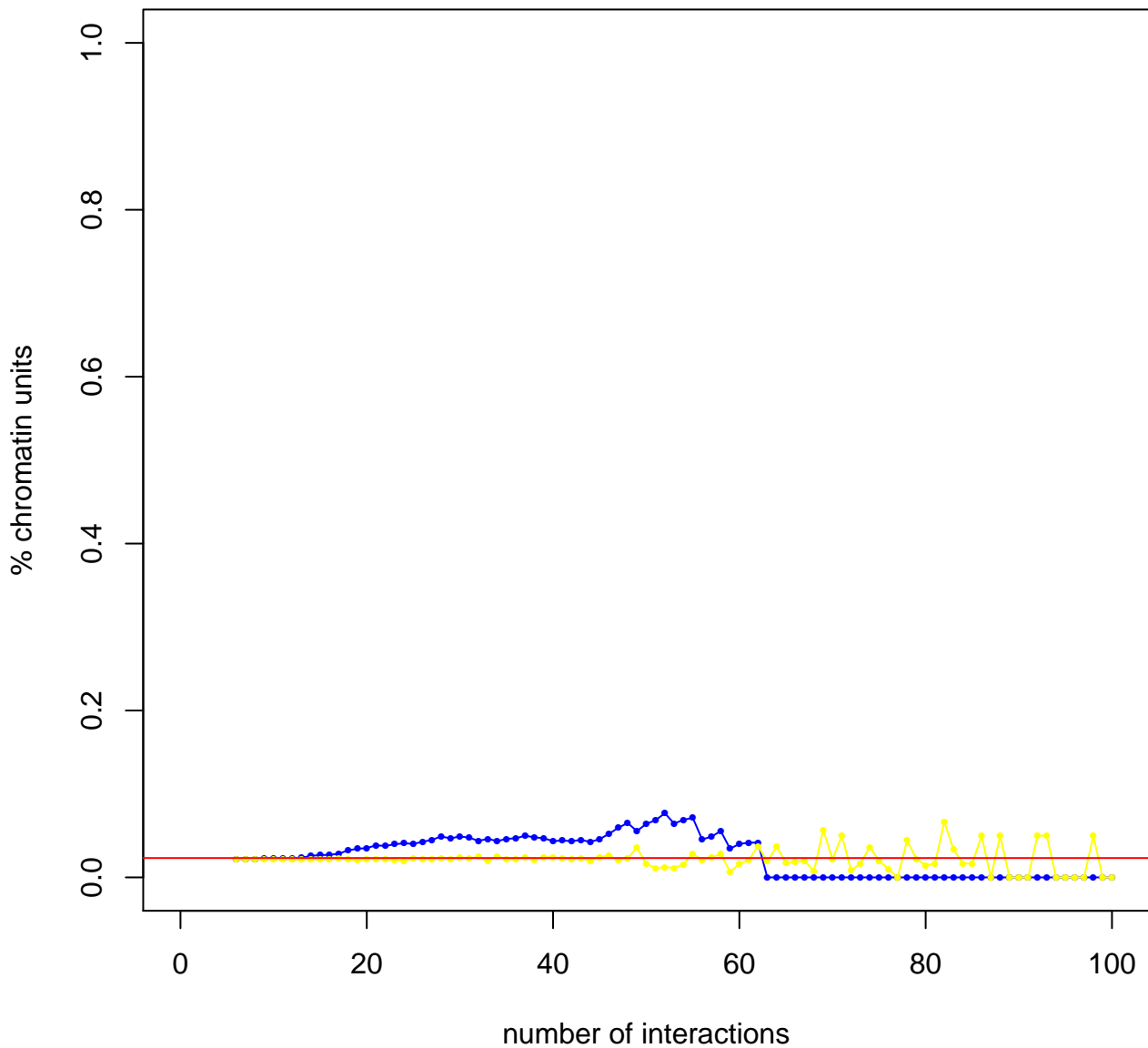

Supplement: Supplementary file 3 — A folder named SB-06-S3 contains 105 overlapping plot for each TF. (ZIP 624 kb) [file 12918_2018_643_MOESM3_ESM.zip › SB-06-S3/SPT2.pdf]

# SPT23

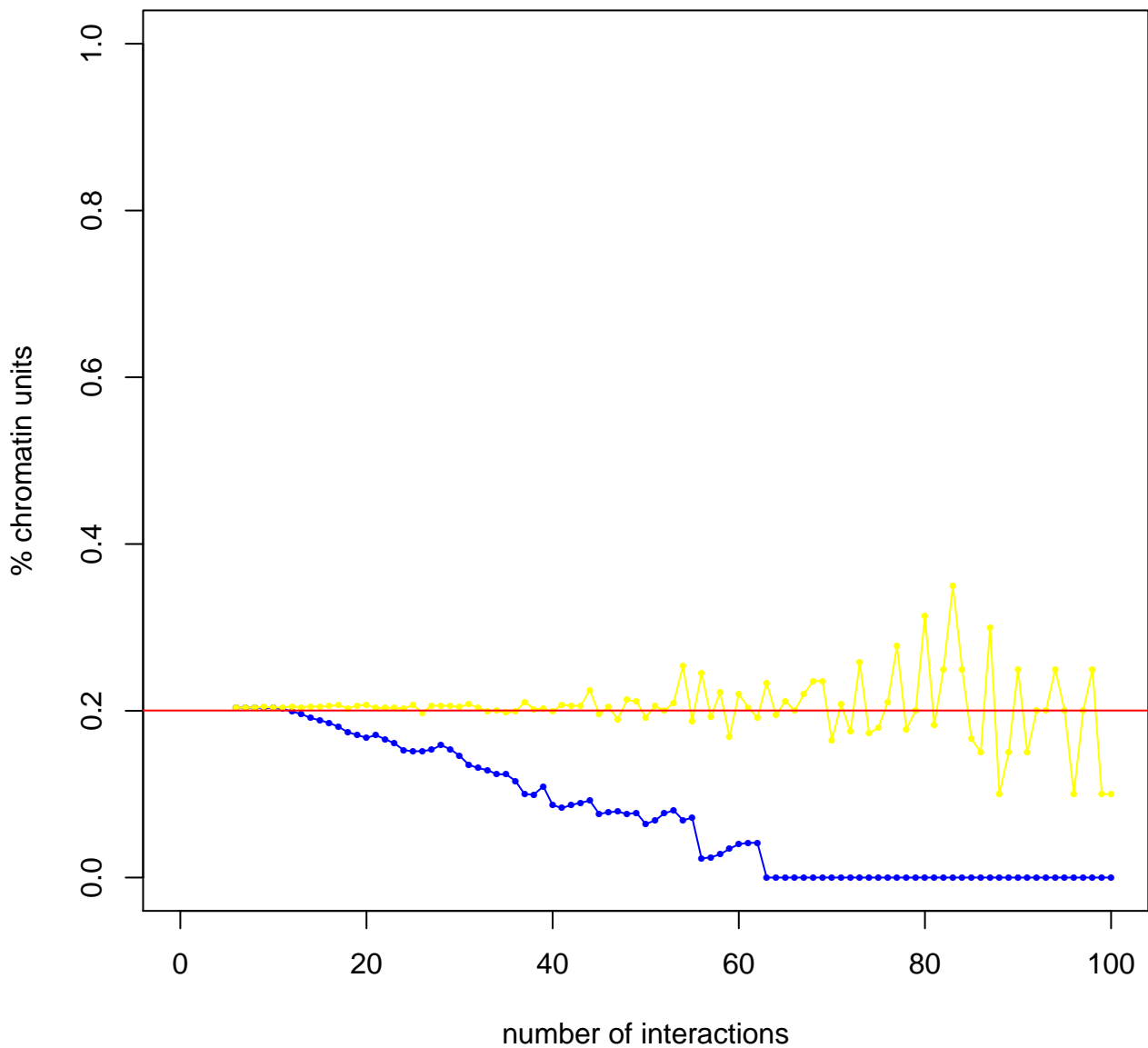

Supplement: Supplementary file 3 — A folder named SB-06-S3 contains 105 overlapping plot for each TF. (ZIP 624 kb) [file 12918_2018_643_MOESM3_ESM.zip › SB-06-S3/SPT23.pdf]

# STB1

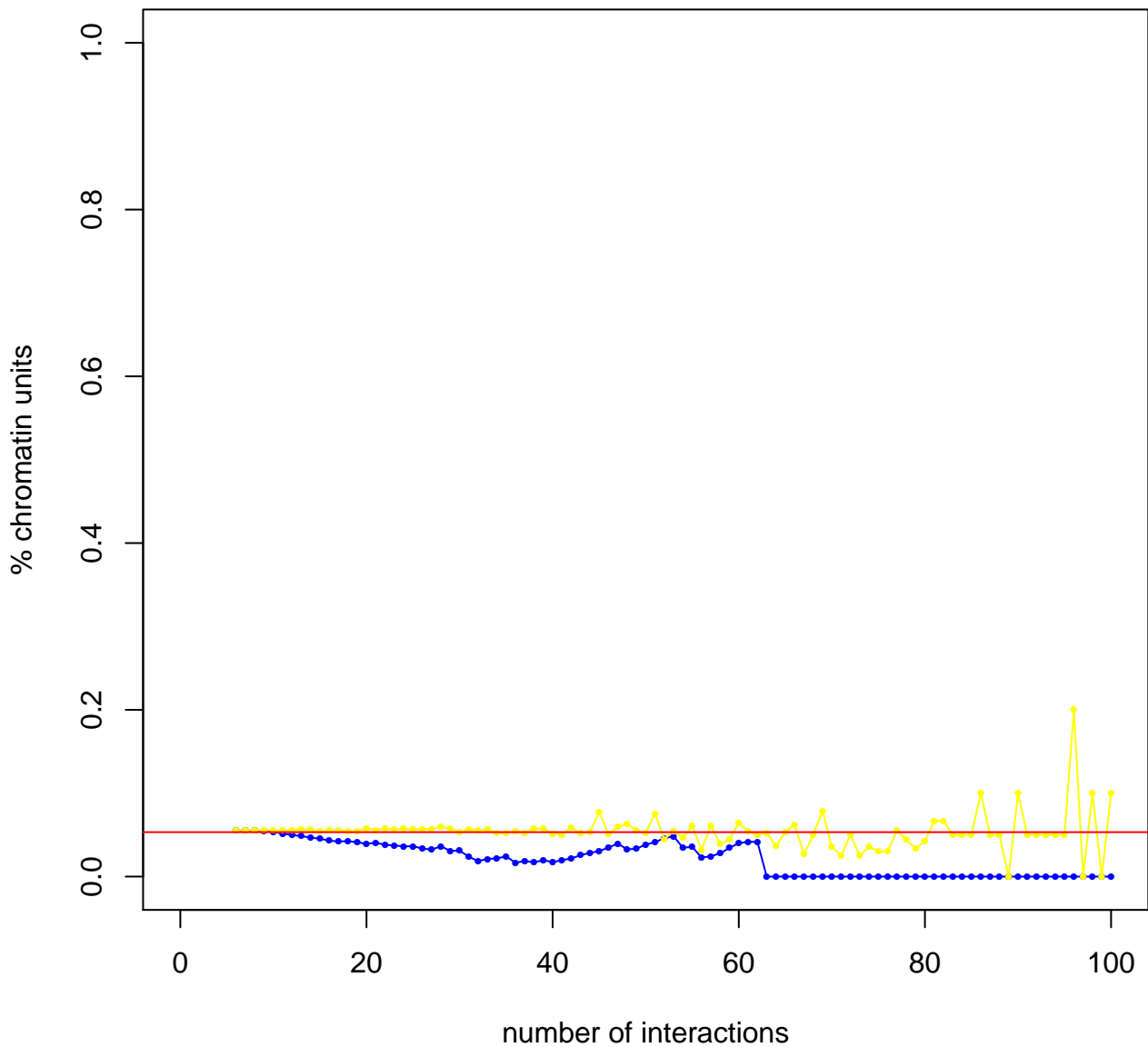

Supplement: Supplementary file 3 — A folder named SB-06-S3 contains 105 overlapping plot for each TF. (ZIP 624 kb) [file 12918_2018_643_MOESM3_ESM.zip › SB-06-S3/STB1.pdf]

# STB4

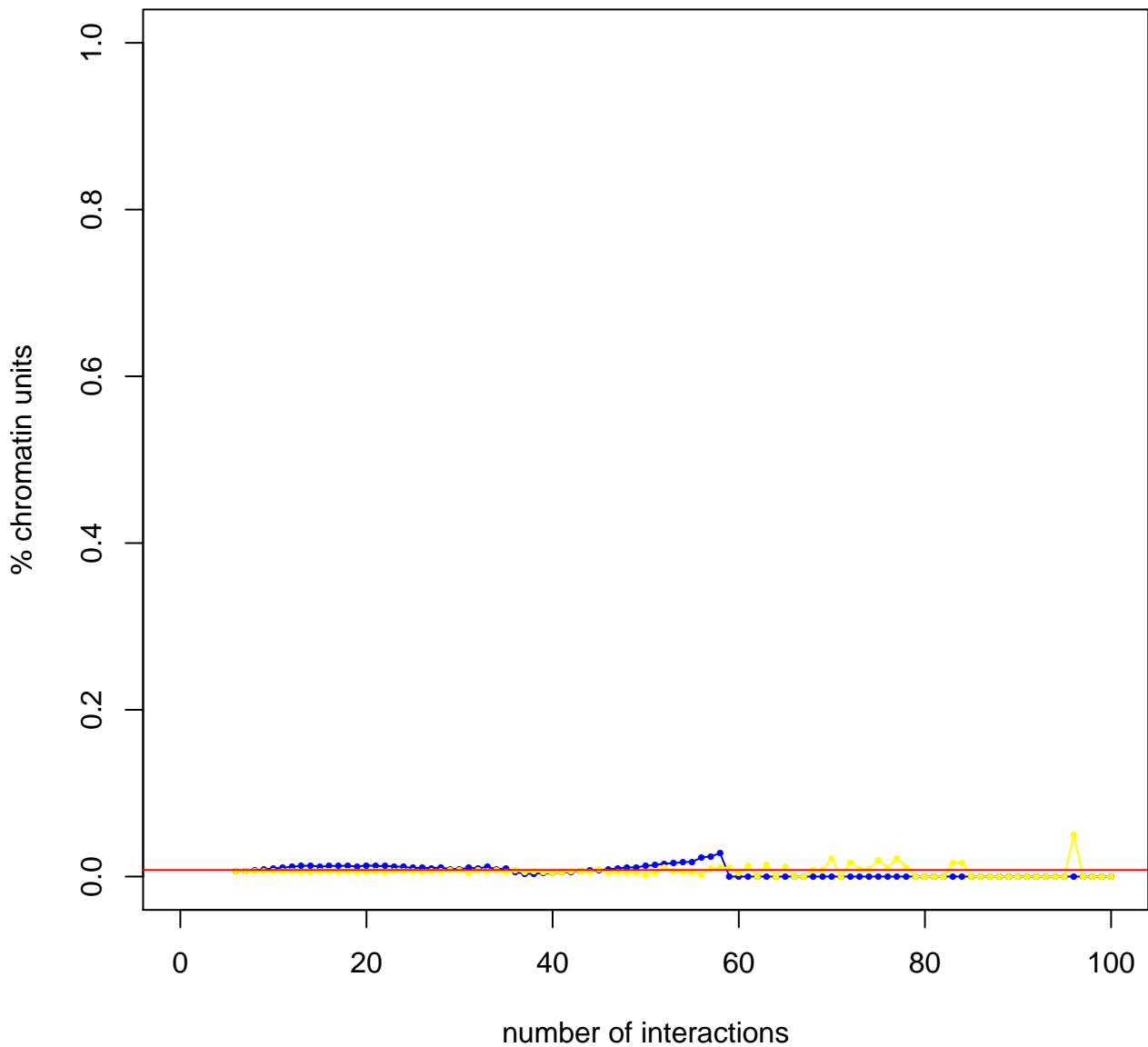

Supplement: Supplementary file 3 — A folder named SB-06-S3 contains 105 overlapping plot for each TF. (ZIP 624 kb) [file 12918_2018_643_MOESM3_ESM.zip › SB-06-S3/STB4.pdf]

# STB5

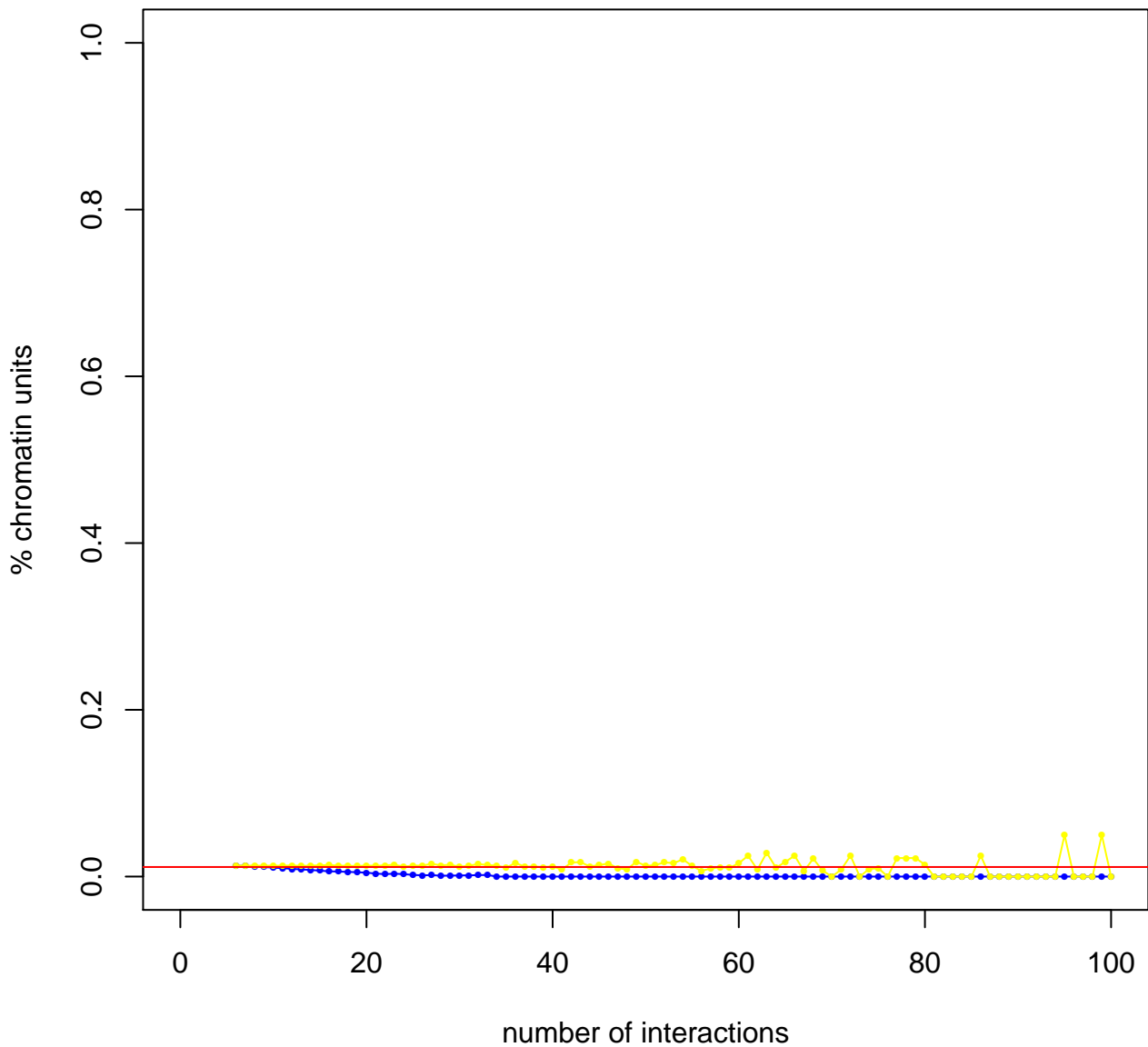

Supplement: Supplementary file 3 — A folder named SB-06-S3 contains 105 overlapping plot for each TF. (ZIP 624 kb) [file 12918_2018_643_MOESM3_ESM.zip › SB-06-S3/STB5.pdf]

# STE12

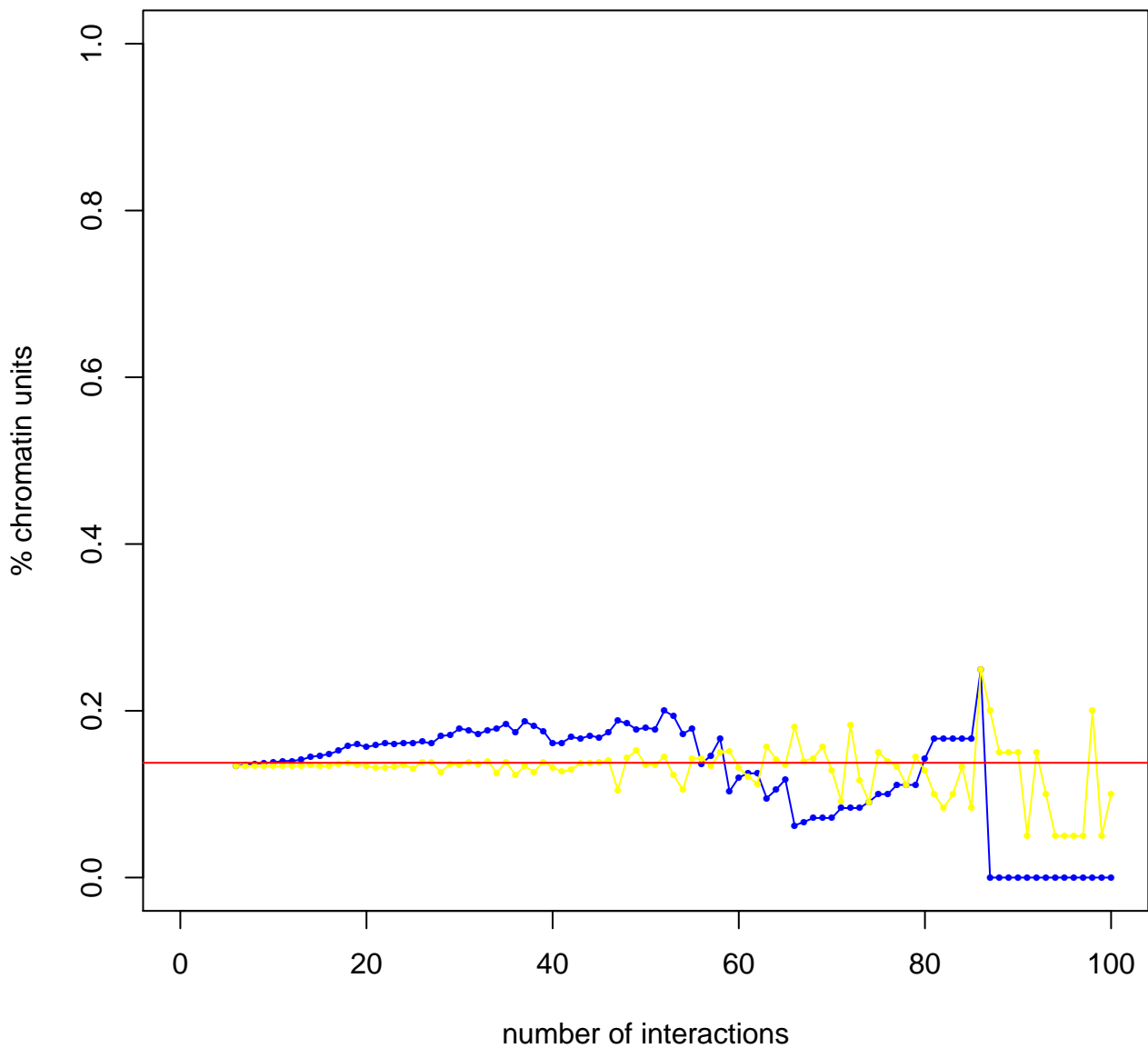

Supplement: Supplementary file 3 — A folder named SB-06-S3 contains 105 overlapping plot for each TF. (ZIP 624 kb) [file 12918_2018_643_MOESM3_ESM.zip › SB-06-S3/STE12.pdf]

# STP1

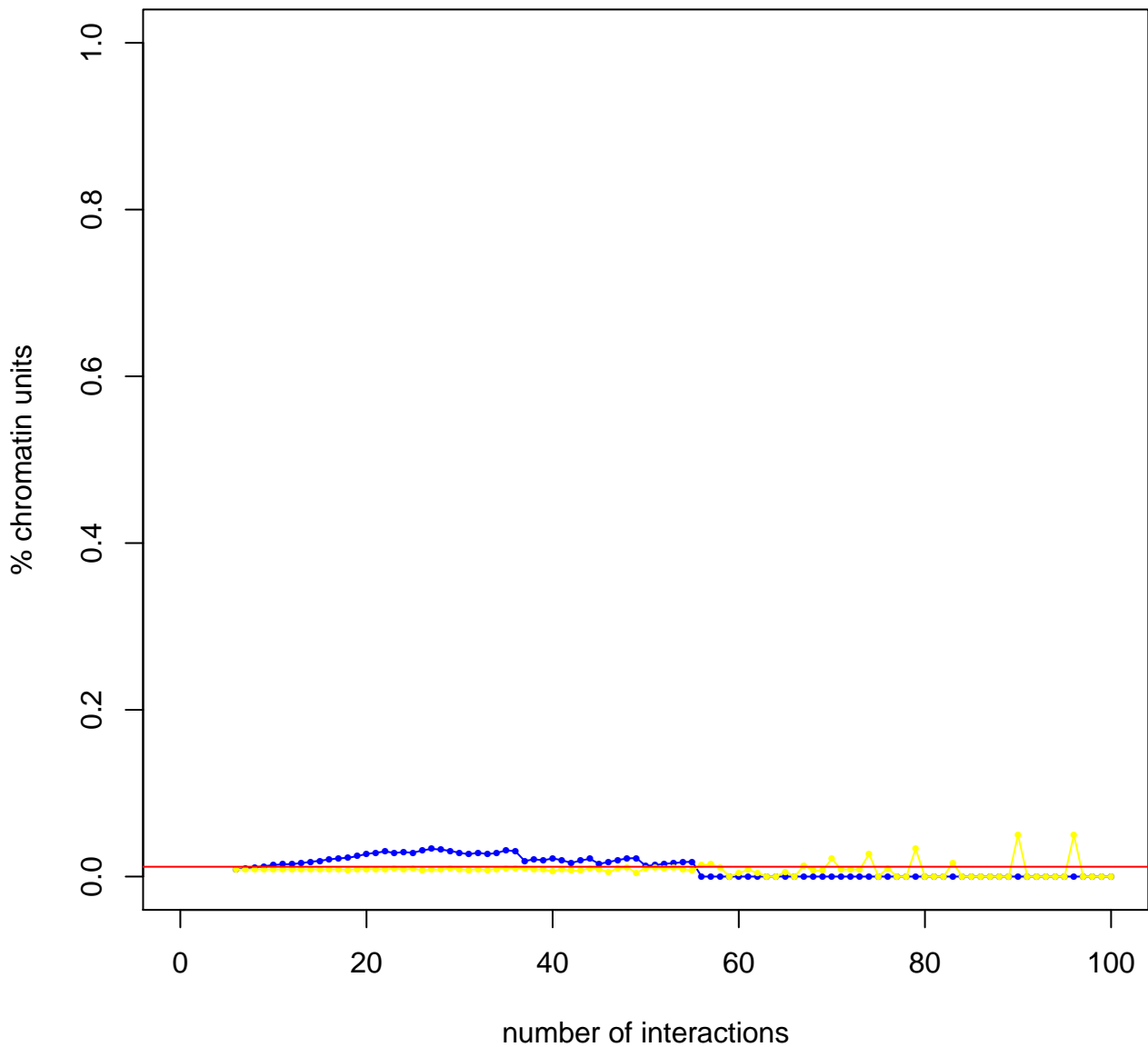

Supplement: Supplementary file 3 — A folder named SB-06-S3 contains 105 overlapping plot for each TF. (ZIP 624 kb) [file 12918_2018_643_MOESM3_ESM.zip › SB-06-S3/STP1.pdf]

# SUM1

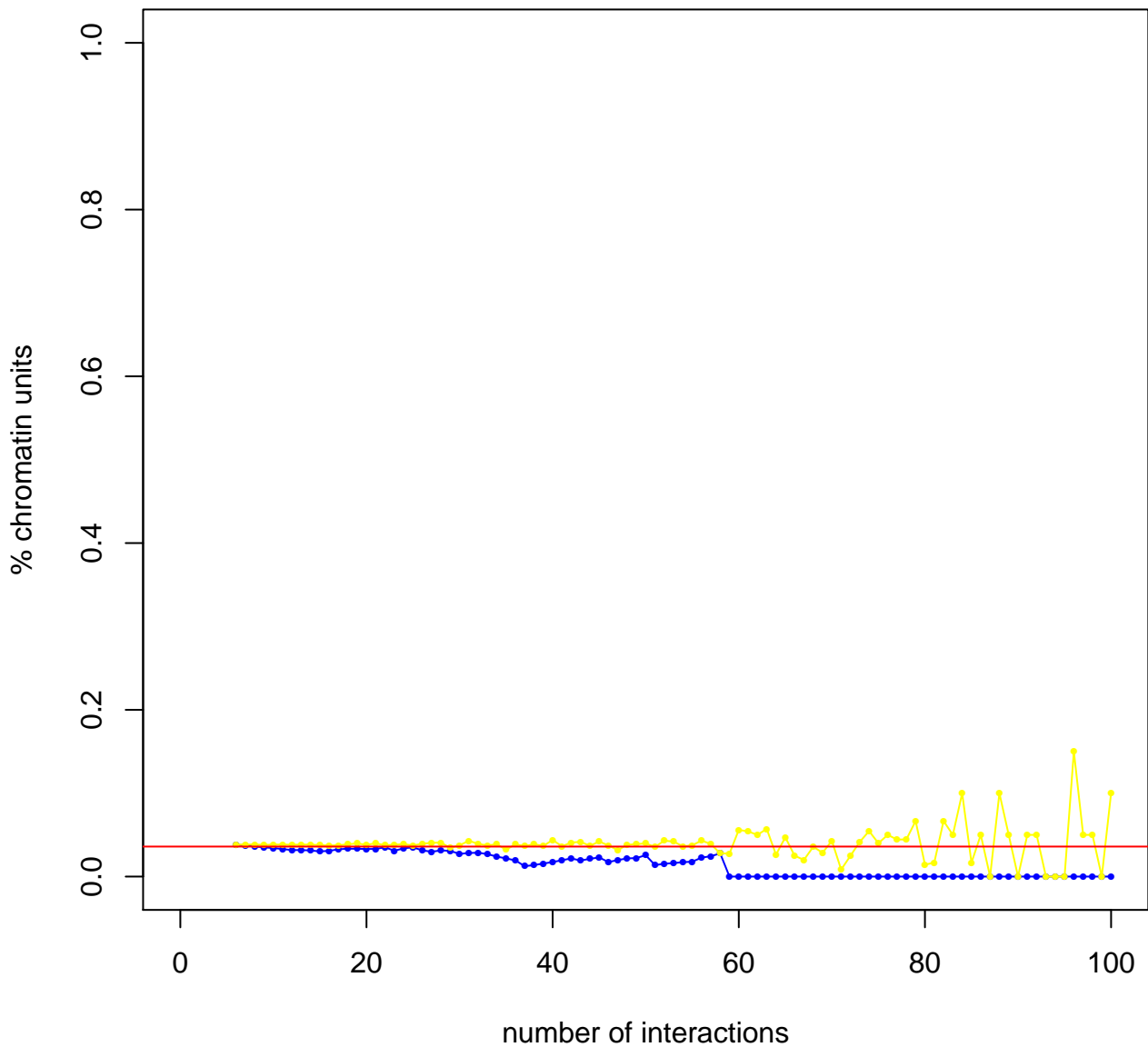

Supplement: Supplementary file 3 — A folder named SB-06-S3 contains 105 overlapping plot for each TF. (ZIP 624 kb) [file 12918_2018_643_MOESM3_ESM.zip › SB-06-S3/SUM1.pdf]

# SUT1

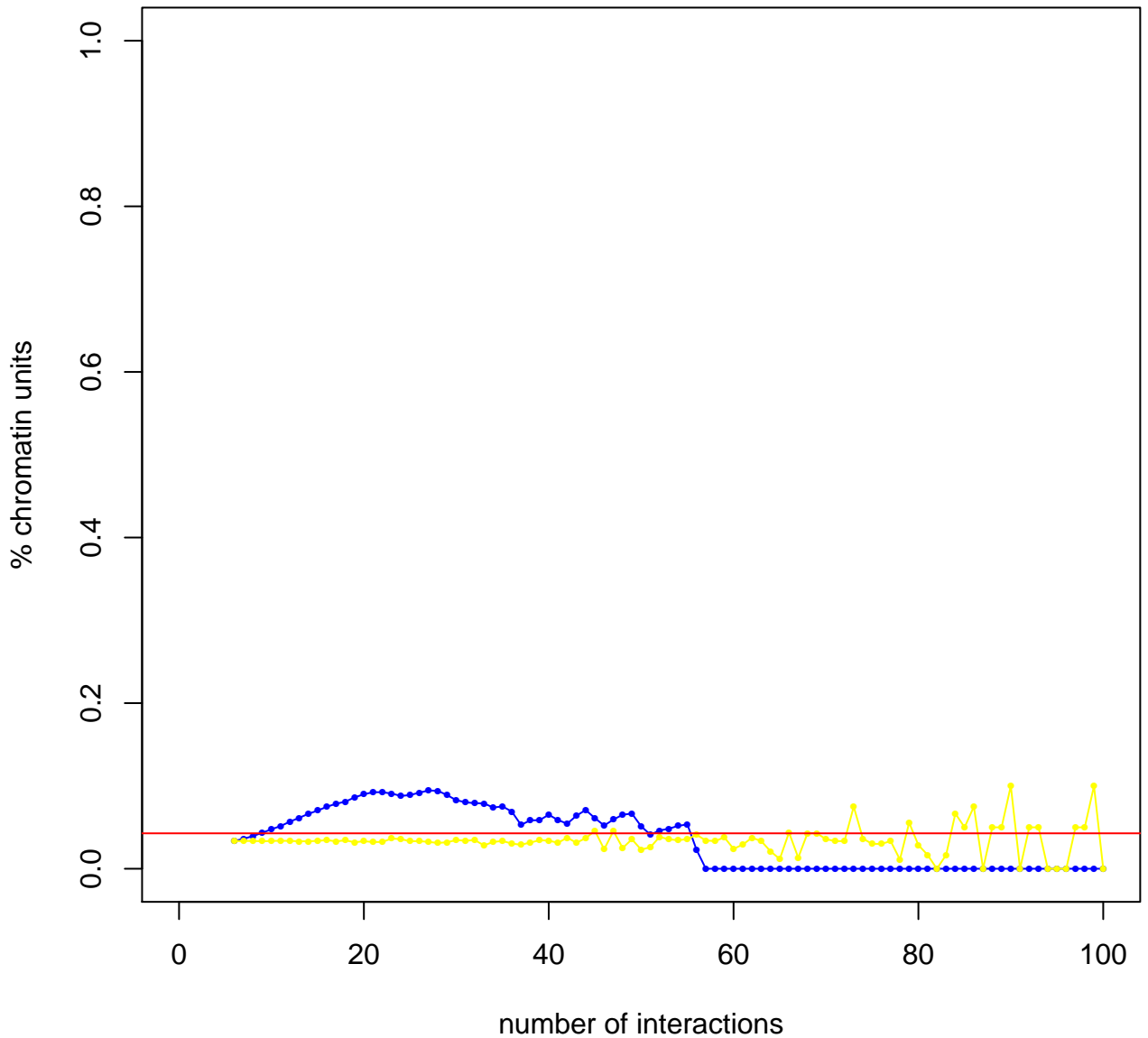

Supplement: Supplementary file 3 — A folder named SB-06-S3 contains 105 overlapping plot for each TF. (ZIP 624 kb) [file 12918_2018_643_MOESM3_ESM.zip › SB-06-S3/SUT1.pdf]

# SWI4

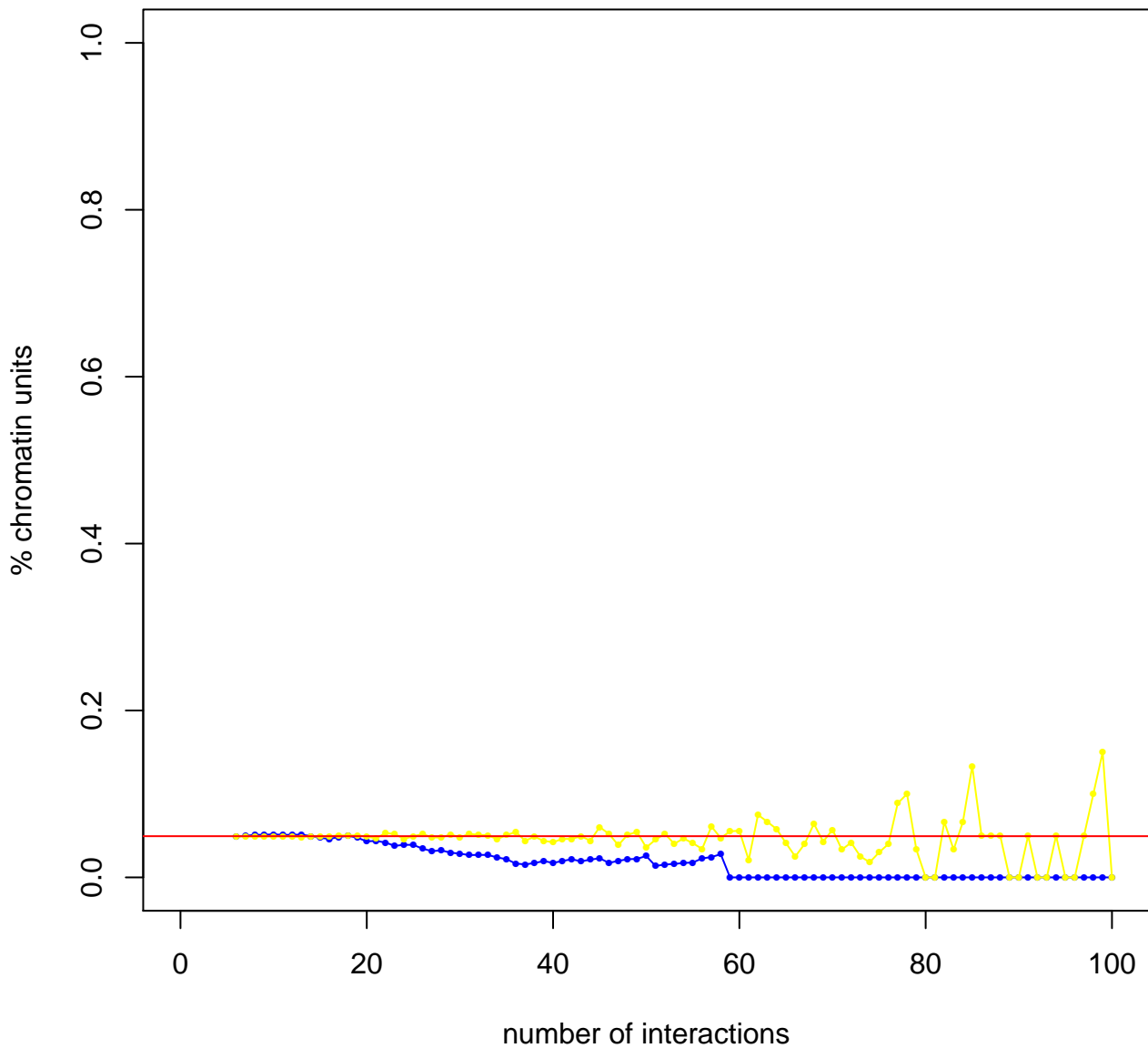

Supplement: Supplementary file 3 — A folder named SB-06-S3 contains 105 overlapping plot for each TF. (ZIP 624 kb) [file 12918_2018_643_MOESM3_ESM.zip › SB-06-S3/SWI4.pdf]

# SWI5

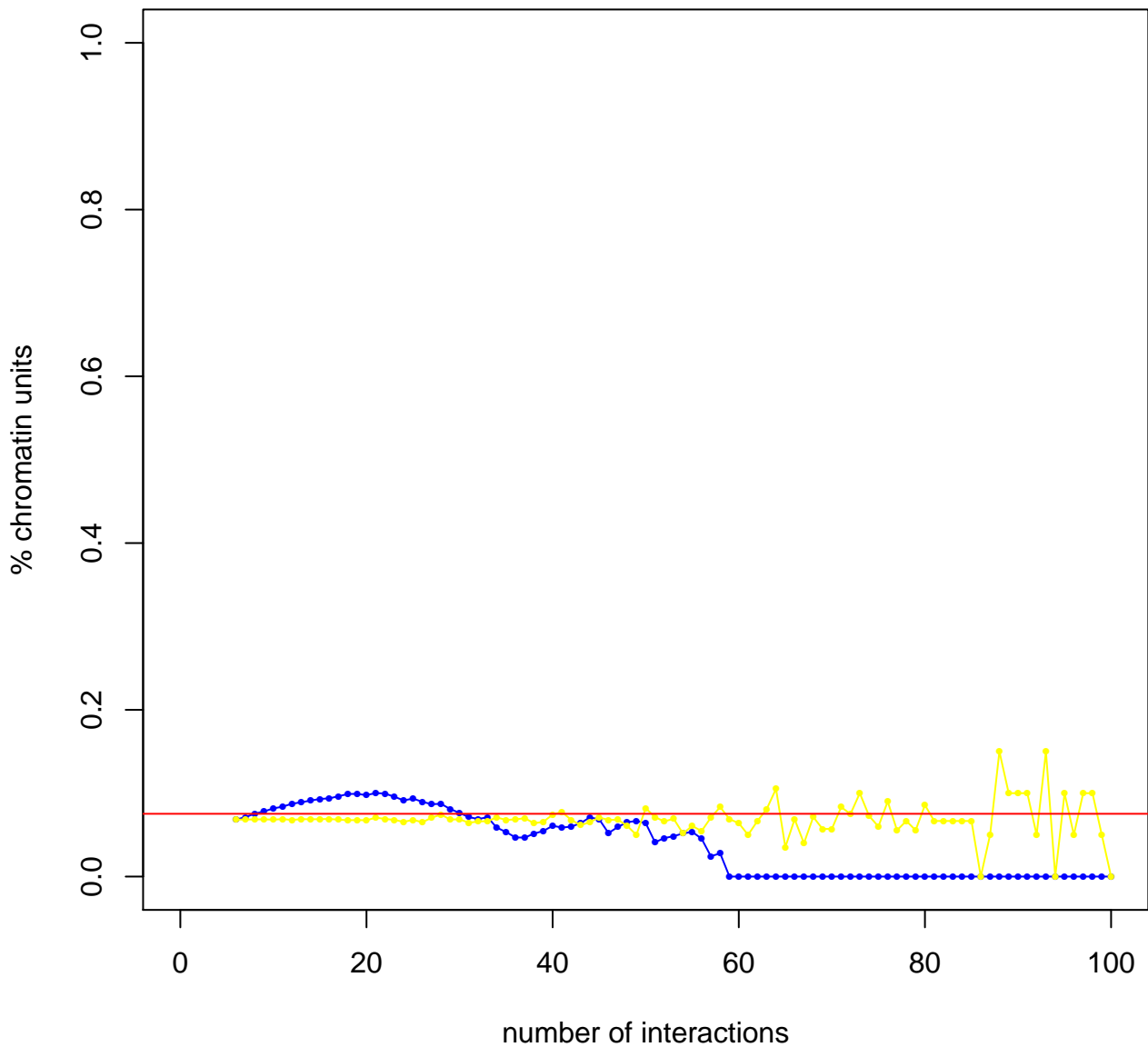

Supplement: Supplementary file 3 — A folder named SB-06-S3 contains 105 overlapping plot for each TF. (ZIP 624 kb) [file 12918_2018_643_MOESM3_ESM.zip › SB-06-S3/SWI5.pdf]

# SWI6

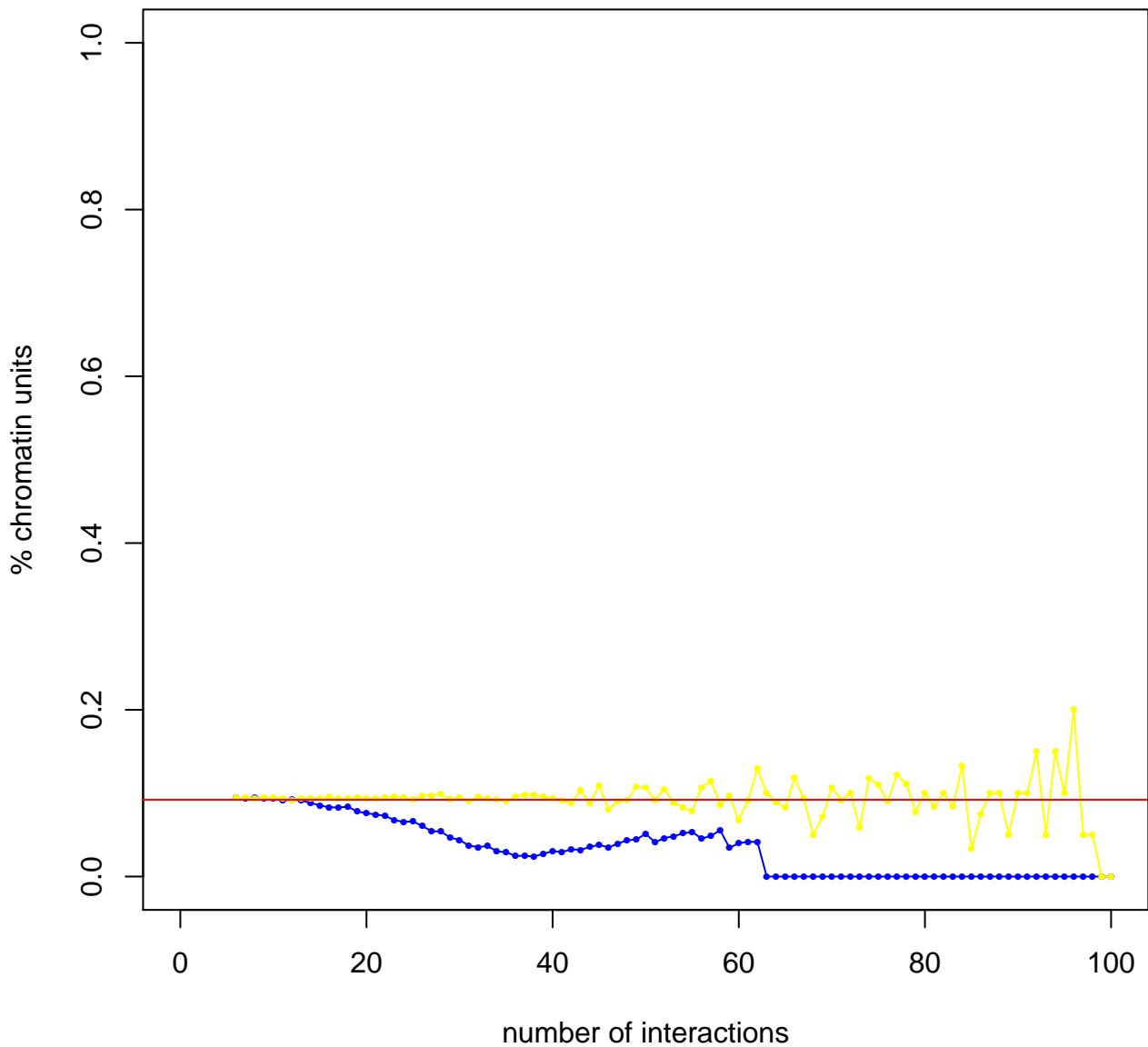

Supplement: Supplementary file 3 — A folder named SB-06-S3 contains 105 overlapping plot for each TF. (ZIP 624 kb) [file 12918_2018_643_MOESM3_ESM.zip › SB-06-S3/SWI6.pdf]

# TEC1

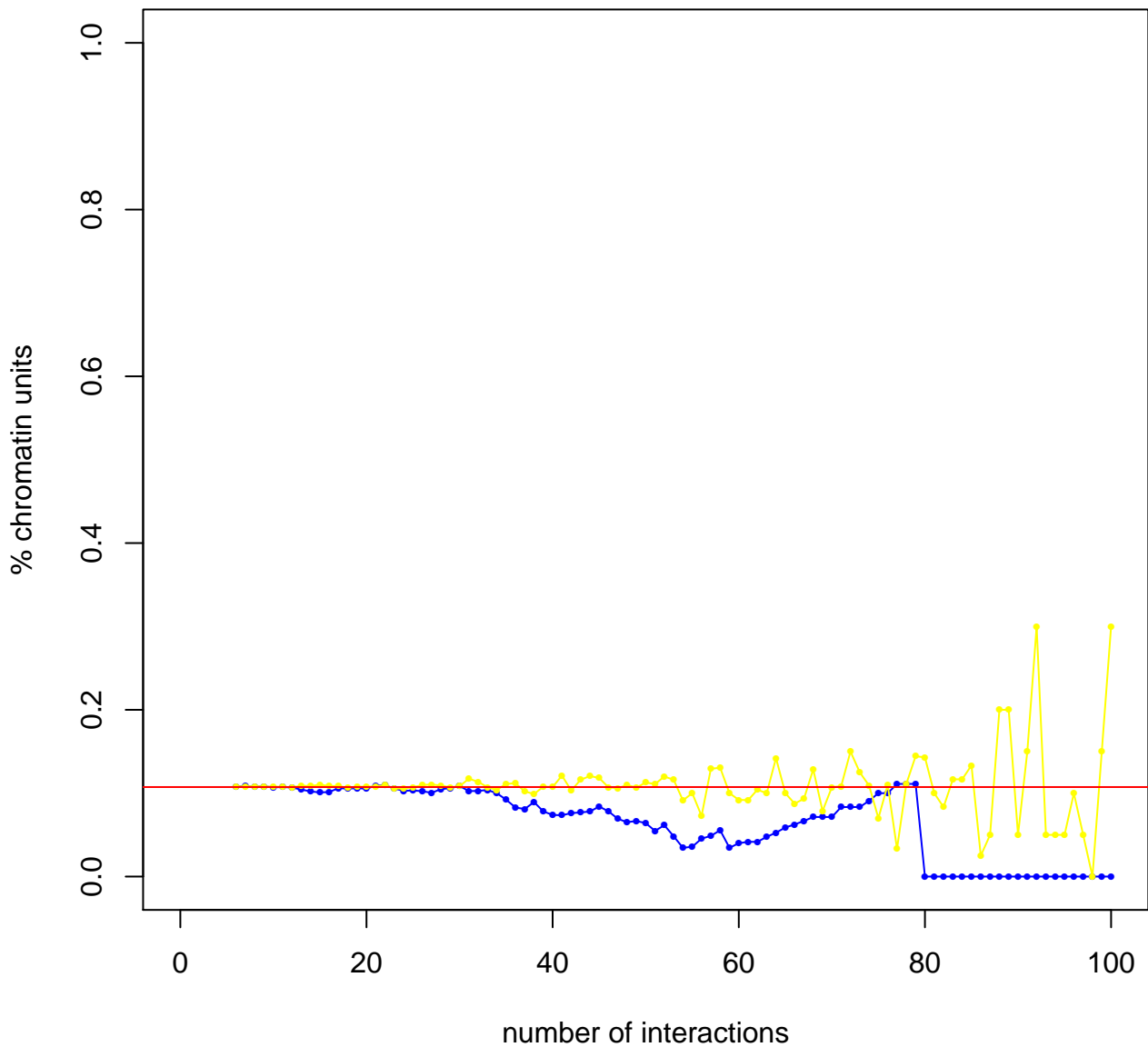

Supplement: Supplementary file 3 — A folder named SB-06-S3 contains 105 overlapping plot for each TF. (ZIP 624 kb) [file 12918_2018_643_MOESM3_ESM.zip › SB-06-S3/TEC1.pdf]

# THI2

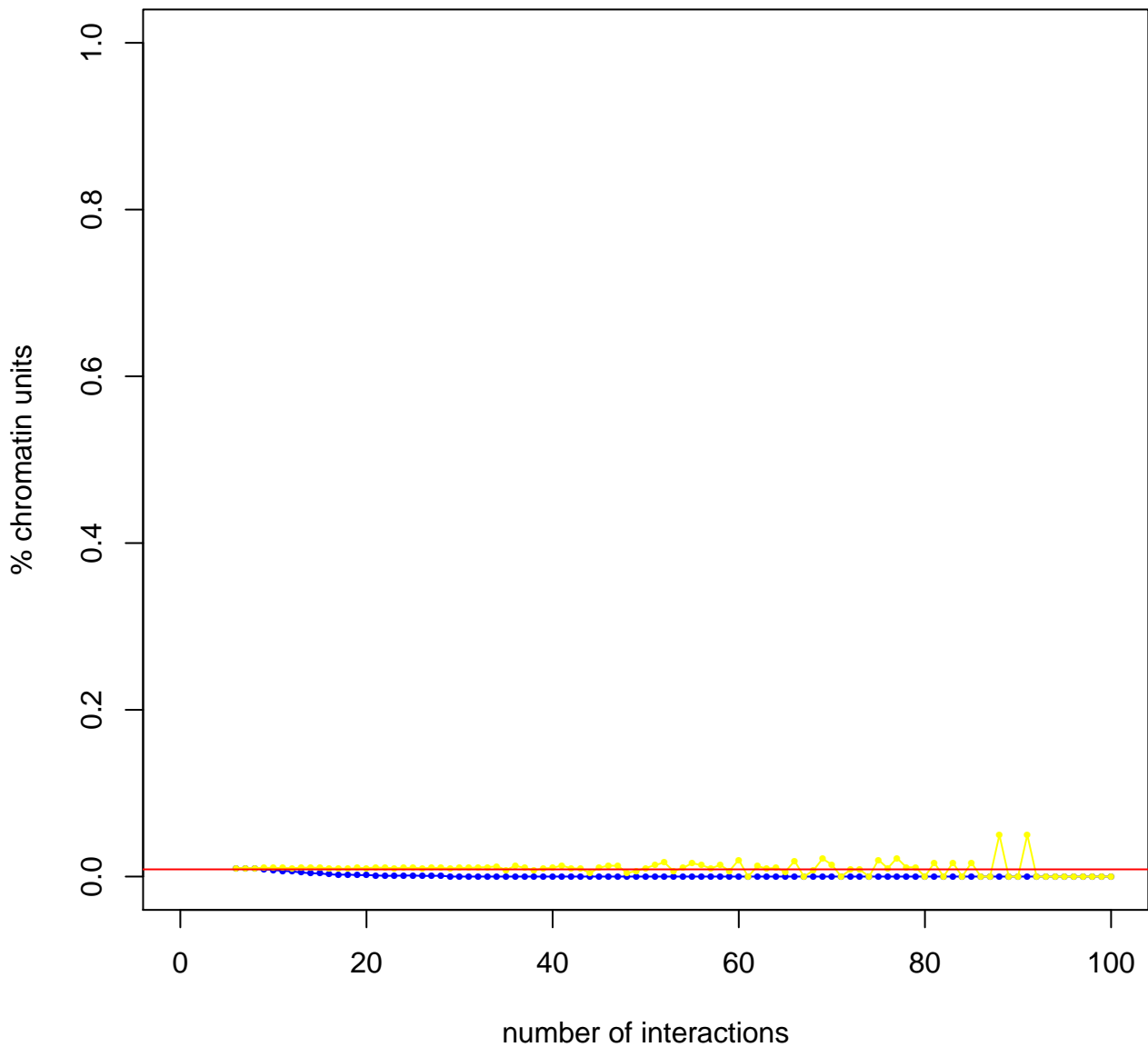

Supplement: Supplementary file 3 — A folder named SB-06-S3 contains 105 overlapping plot for each TF. (ZIP 624 kb) [file 12918_2018_643_MOESM3_ESM.zip › SB-06-S3/THI2.pdf]

# TYE7

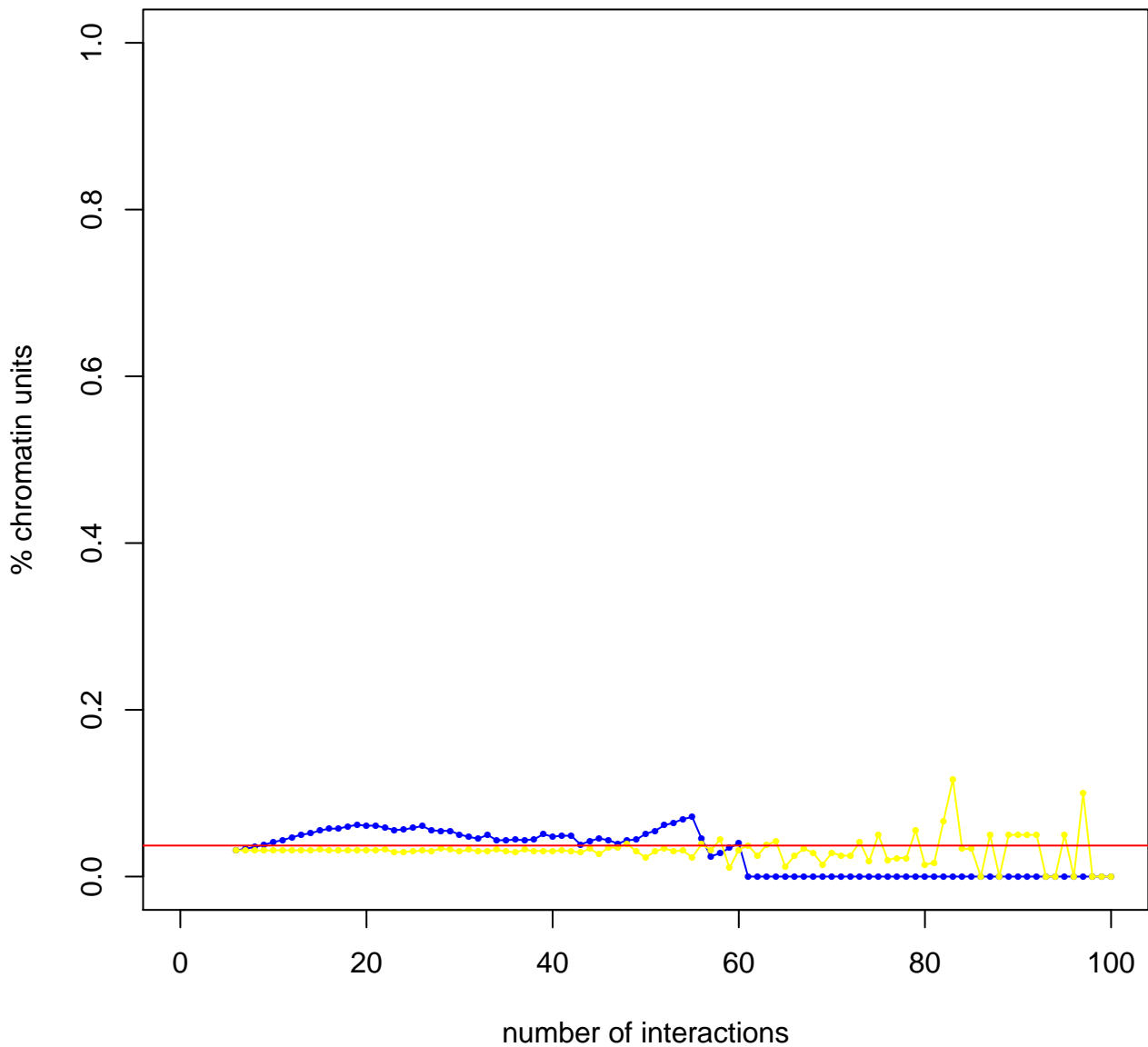

Supplement: Supplementary file 3 — A folder named SB-06-S3 contains 105 overlapping plot for each TF. (ZIP 624 kb) [file 12918_2018_643_MOESM3_ESM.zip › SB-06-S3/TYE7.pdf]

# UGA3

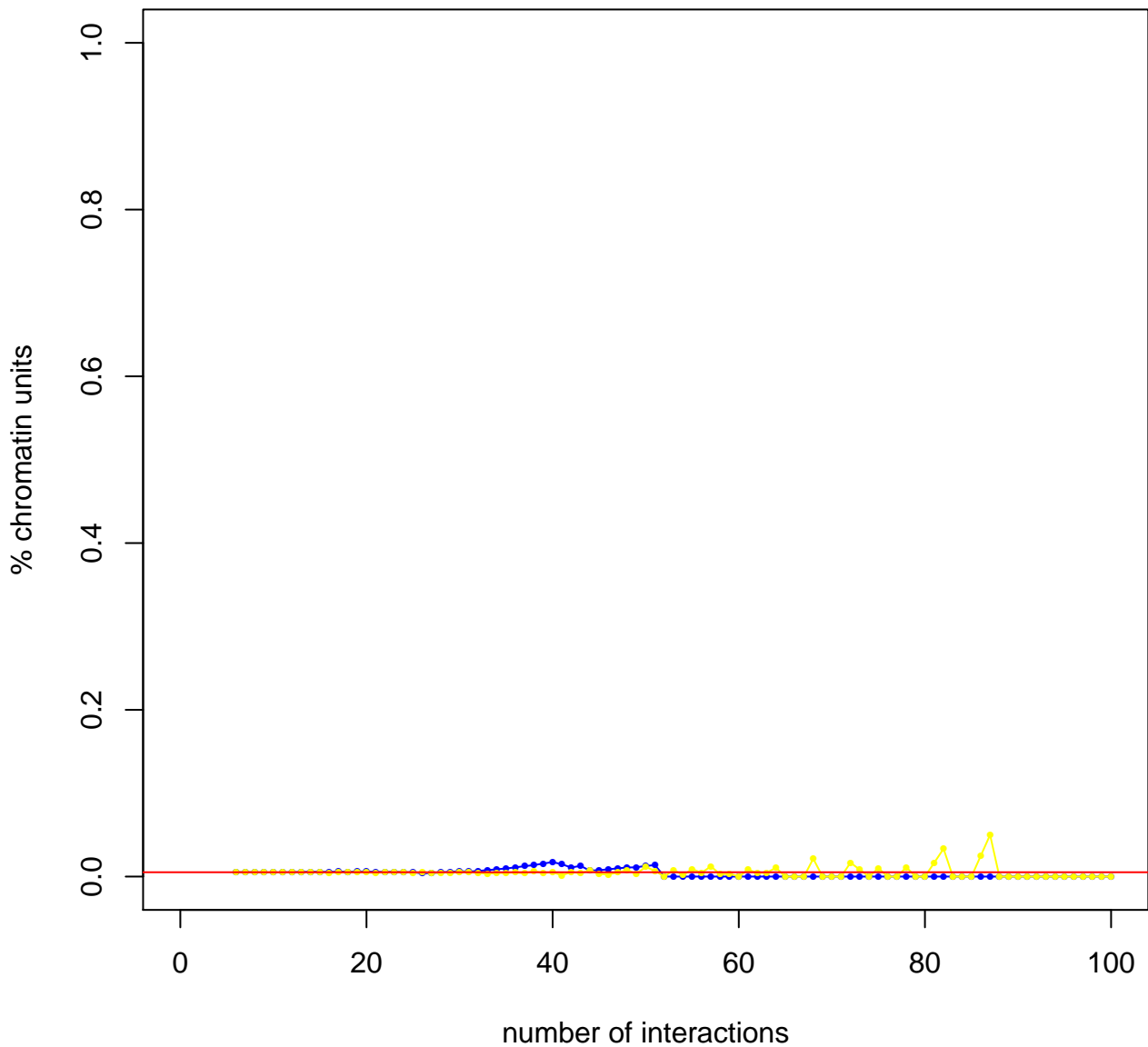

Supplement: Supplementary file 3 — A folder named SB-06-S3 contains 105 overlapping plot for each TF. (ZIP 624 kb) [file 12918_2018_643_MOESM3_ESM.zip › SB-06-S3/UGA3.pdf]

# UME1

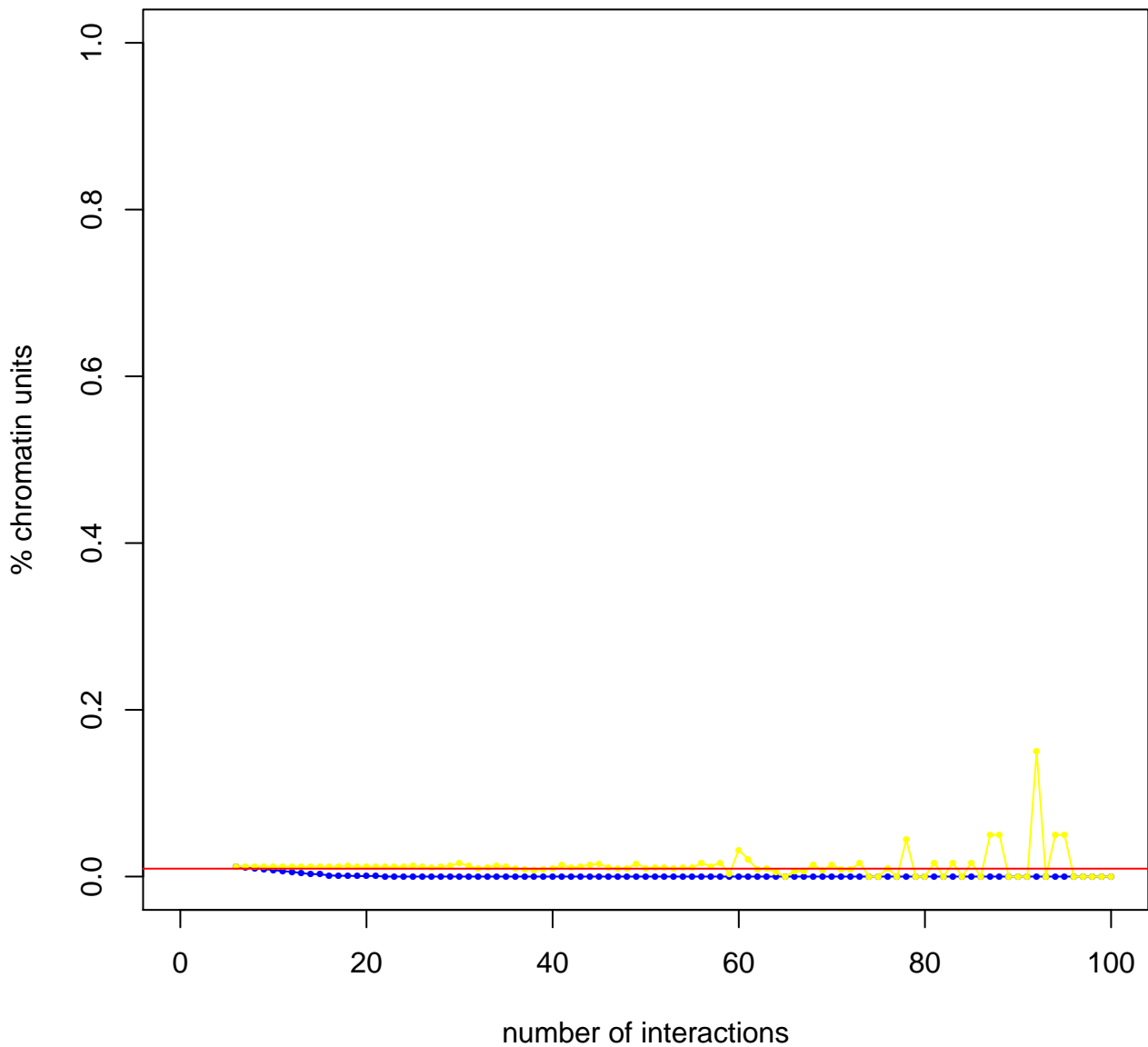

Supplement: Supplementary file 3 — A folder named SB-06-S3 contains 105 overlapping plot for each TF. (ZIP 624 kb) [file 12918_2018_643_MOESM3_ESM.zip › SB-06-S3/UME1.pdf]

# UME6

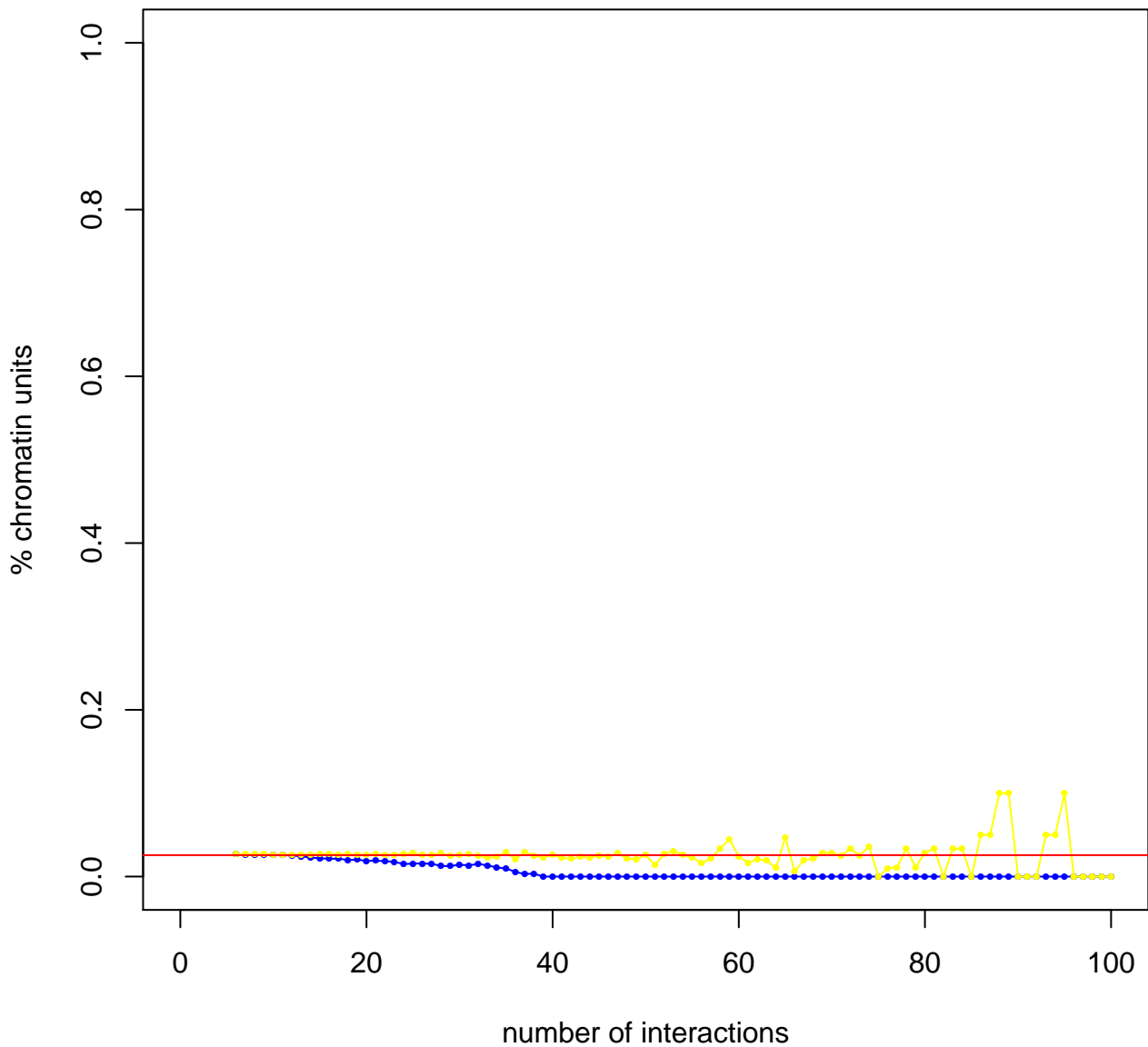

Supplement: Supplementary file 3 — A folder named SB-06-S3 contains 105 overlapping plot for each TF. (ZIP 624 kb) [file 12918_2018_643_MOESM3_ESM.zip › SB-06-S3/UME6.pdf]

# XBP1

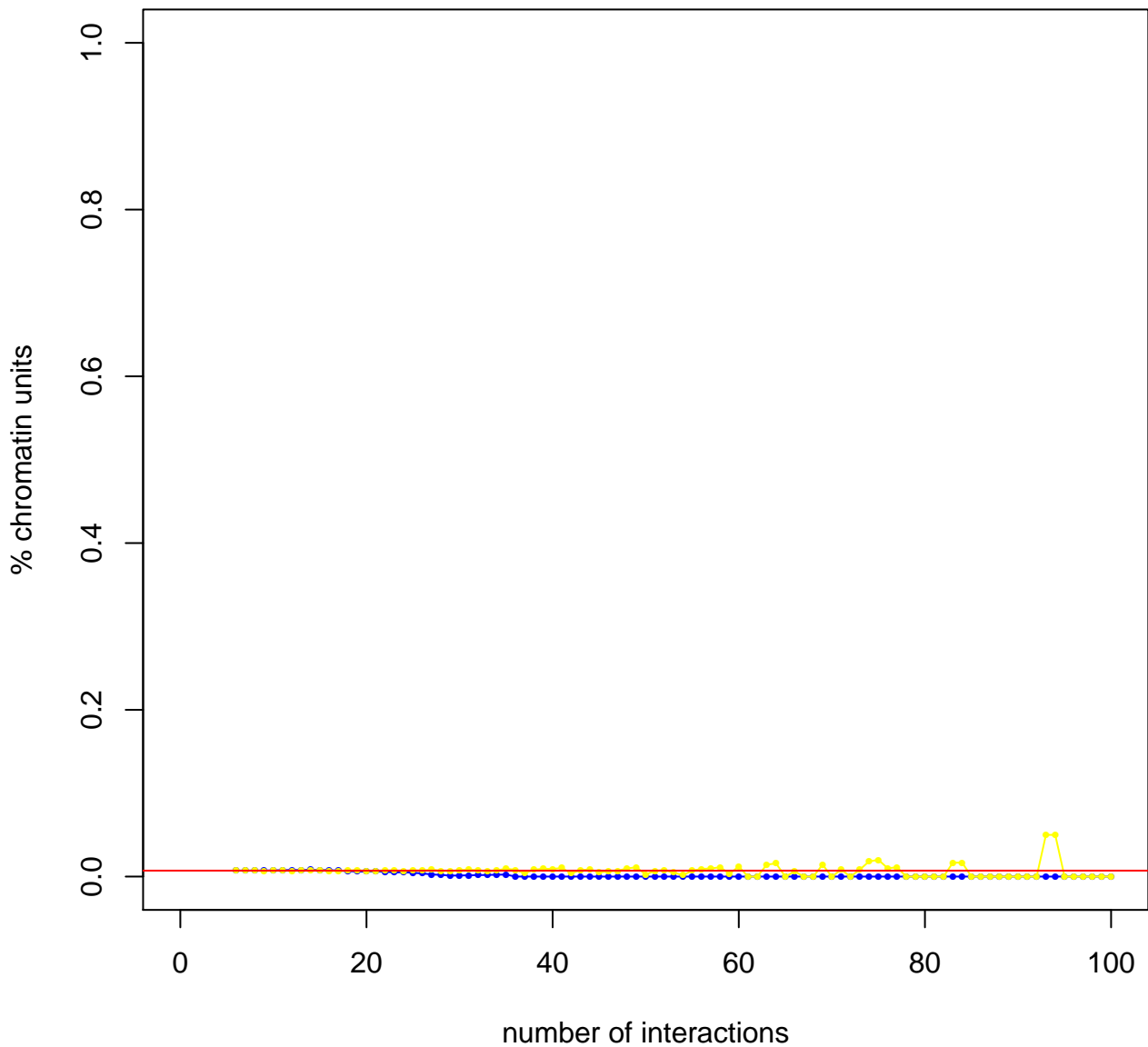

Supplement: Supplementary file 3 — A folder named SB-06-S3 contains 105 overlapping plot for each TF. (ZIP 624 kb) [file 12918_2018_643_MOESM3_ESM.zip › SB-06-S3/XBP1.pdf]

# YAP1

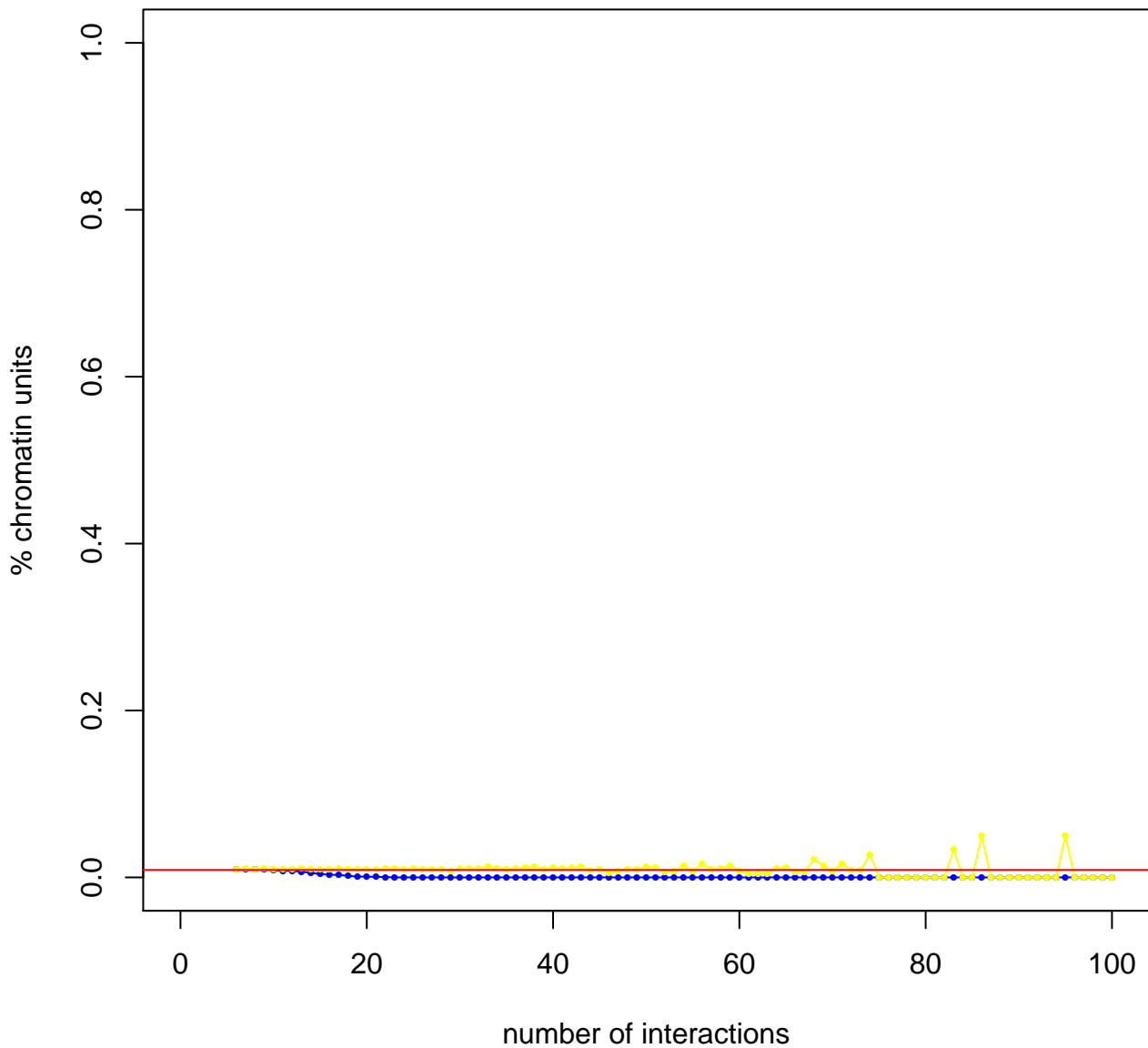

Supplement: Supplementary file 3 — A folder named SB-06-S3 contains 105 overlapping plot for each TF. (ZIP 624 kb) [file 12918_2018_643_MOESM3_ESM.zip › SB-06-S3/YAP1.pdf]

# YAP3

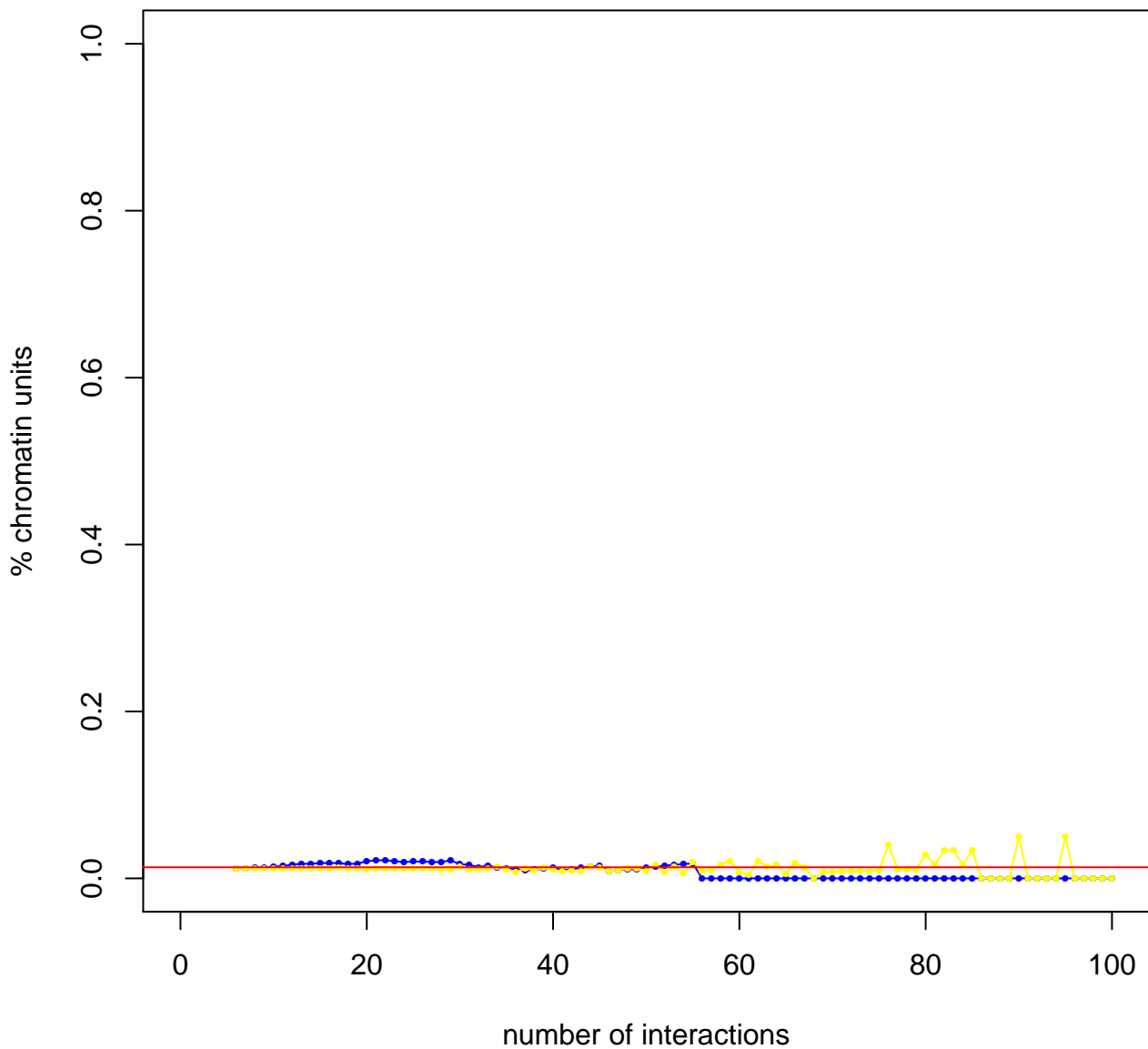

Supplement: Supplementary file 3 — A folder named SB-06-S3 contains 105 overlapping plot for each TF. (ZIP 624 kb) [file 12918_2018_643_MOESM3_ESM.zip › SB-06-S3/YAP3.pdf]

# YAP5

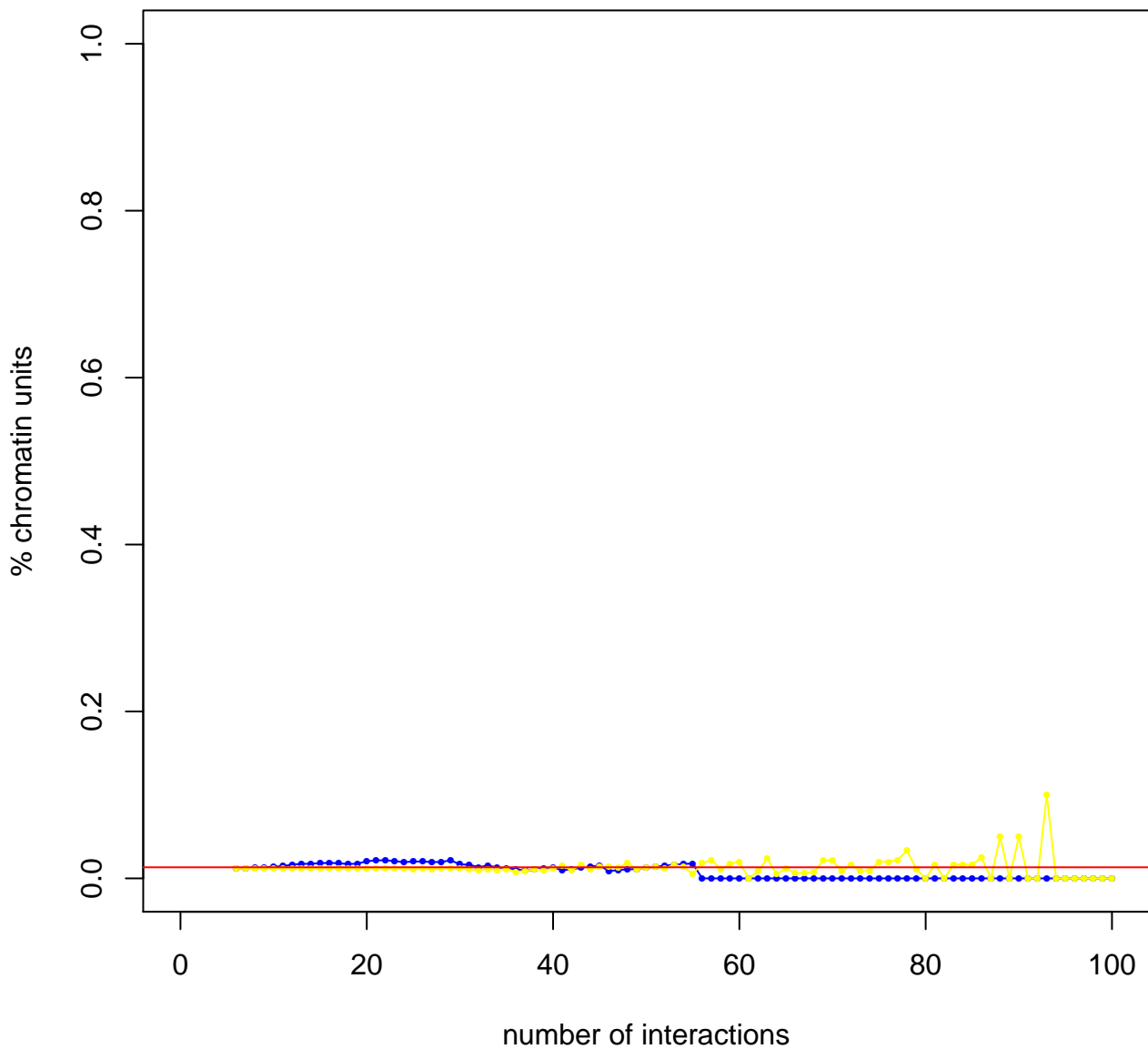

Supplement: Supplementary file 3 — A folder named SB-06-S3 contains 105 overlapping plot for each TF. (ZIP 624 kb) [file 12918_2018_643_MOESM3_ESM.zip › SB-06-S3/YAP5.pdf]

# YAP6

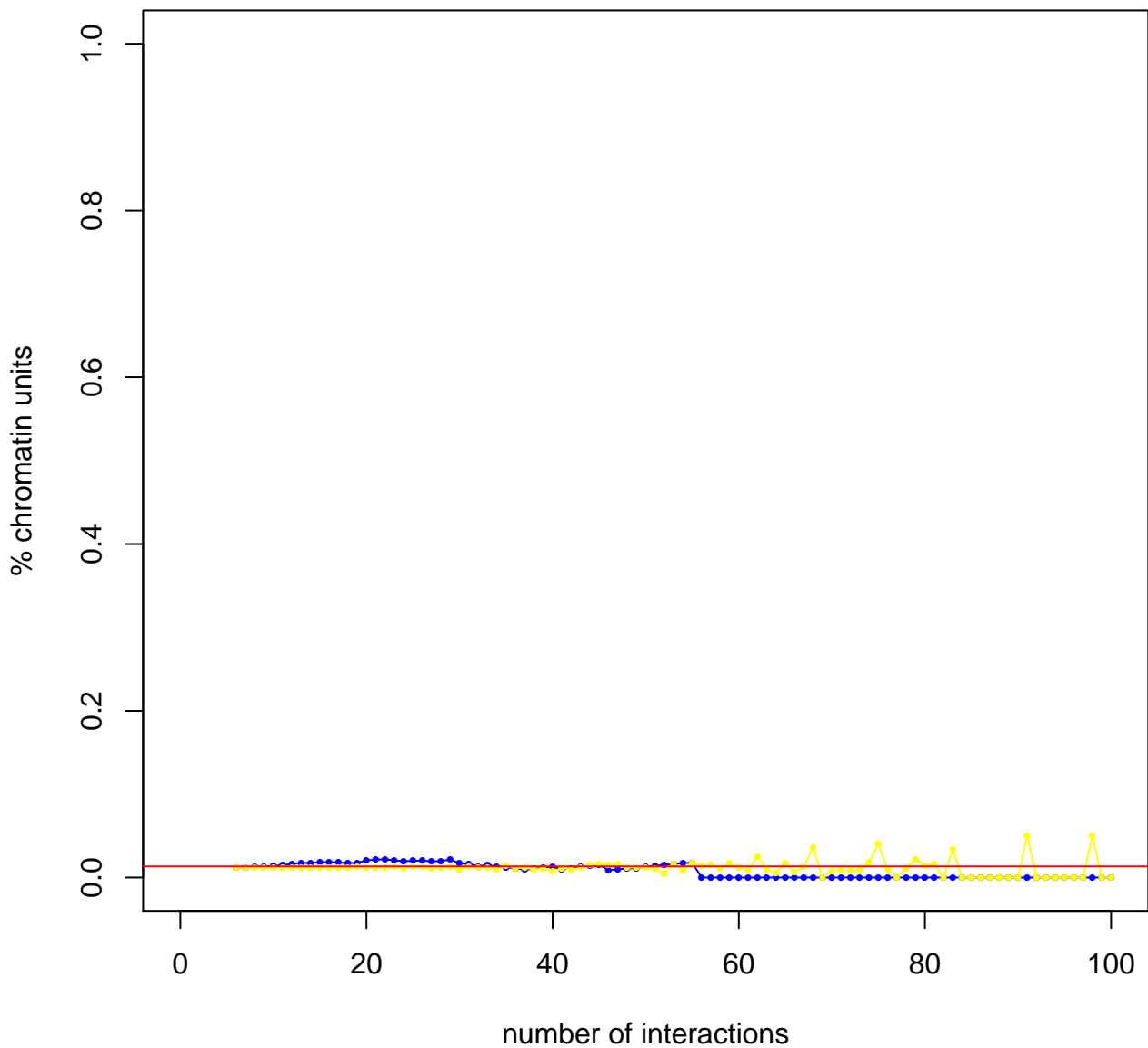

Supplement: Supplementary file 3 — A folder named SB-06-S3 contains 105 overlapping plot for each TF. (ZIP 624 kb) [file 12918_2018_643_MOESM3_ESM.zip › SB-06-S3/YAP6.pdf]
